# Novel Flavonol Glycosides from the Aerial Parts of Lentil (*Lens culinaris*)

**DOI:** 10.3390/molecules191118152

**Published:** 2014-11-06

**Authors:** Jerzy Żuchowski, Łukasz Pecio, Anna Stochmal

**Affiliations:** Department of Biochemistry, Institute of Soil Science and Plant Cultivation—State Research Institute, ul. Czartoryskich 8, Puławy 24-100, Poland; E-Mails: lpecio@iung.pulawy.pl (Ł.P.); asf@iung.pulawy.pl (A.S.)

**Keywords:** lentil, *Lens culinaris*, phenolic compounds, flavonoids, NMR

## Abstract

While the phytochemical composition of lentil (*Lens culinaris*) seeds is well described in scientific literature, there is very little available data about secondary metabolites from lentil leaves and stems. Our research reveals that the aerial parts of lentil are a rich source of flavonoids. Six kaempferol and twelve quercetin glycosides were isolated, their structures were elucidated using NMR spectroscopy and chemical methods. This group includes 16 compounds which have not been previously described in the scientific literature: quercetin 3-*O*-β-D-glucopyranosyl(1→2)-β-D-galactopyranoside-7-*O*-β-D-glucuropyranoside (**1**), kaempferol 3-*O*-β-D-glucopyranosyl(1→2)-β-D-galacto-pyranoside-7-*O*-β-D-glucuropyranoside (**3**), their derivatives **4**–**10**, **12**–**15**, **17**, **18** acylated with caffeic, *p*-coumaric, ferulic, or 3,4,5-trihydroxycinnamic acid and kaempferol 3-*O*-{[(6-*O*-*E*-*p*-coumaroyl)-β-D-glucopyranosyl(1→2)]-α-L-rhamnopyranosyl(1→6)}-β-D-galactopyranoside-7-*O*-α-L-rhamnopyranoside (**11**). Their DPPH scavenging activity was also evaluated. This is probably the first detailed description of flavonoids from the aerial parts of lentil.

## 1. Introduction

Lentil (*Lens culinaris* Medik) whose cultivation started in the Near East in the Neolithic period, is one of the earliest domesticated plants. Nowadays, lentil is a crop of high importance in many countries of Northern Africa, Western and Southern Asia, as well as in Canada, which is the world leader in its production [1]. The nutritional value of legumes is commonly known, especially their high content of good quality protein. Lentil grain contains, on average, about 28% of protein, and is rich in lysine and several other essential amino acids. It is also a good source of minerals (Ca, Fe, K, Mg, P, Zn), some B-group vitamins and pantothenic acid [2]. Lentil straw finds use as a valued fodder in many parts of the Near East [3].

There is a broad literature on basic nutrients and raffinose family oligosaccharides of lentil grain, but the number of publications about lentil secondary metabolites is much more limited. The seeds of this plant are known to contain phytosterols, phytic acid, saponins and phenolic compounds. The seed phenolics are represented by condensed tannins (present in significant amounts, especially in the seed coat), phenolic acids, lignans, stilbens, and flavonoids. The reported lentil flavonoids comprise mainly catechin and and glycosidic derivatives of kaempferol, quercetin, myricetin, apigenin and luteolin [2,4,5,6,7,8,9]. The marked differences in flavonoid profiles among individual studies can be explained by the use of different cultivars and plant growth conditions. While secondary metabolites of lentil seeds are well characterized, it seems there are hardly any data available about secondary metabolites in other organs of this plant.

Flavonoids are a group of phenolic compounds very widely distributed in the plant kingdom [10]. They are common food constituents, extensively investigated due to their antioxidant properties, diverse biological activities and role in prevention of cardiovascular diseases and cancer [11,12]. Leaves and stems of numerous legumes are known to be a rich source of various types of flavonoids, but there is extremely little information about phenolic compounds from the aerial parts of lentil. A few articles about lentil sprouts provide some more detailed data about phenolics, though flavonoids were only preliminarily identified. However, sprouts of the lentil were reported to contain acylated glycosides of kaempferol and quercetin [13,14,15]. Because of the broad bioactive potential of flavonoids, finding new sources and new types of these compounds still remains an important task, and the aim of our study was to isolate and identify flavonoids from aerial parts of the lentil cultivar Tina.

## 2. Results and Discussion

**Figure 1:**
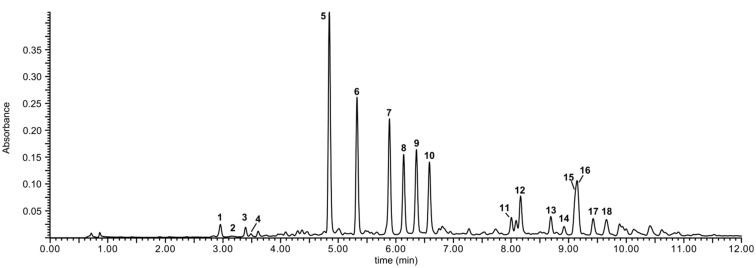
UPLC-UV (330 nm) chromatogram of the crude extract from the aerial parts of lentil *(Lens culinaris*).

**Figure 2:**
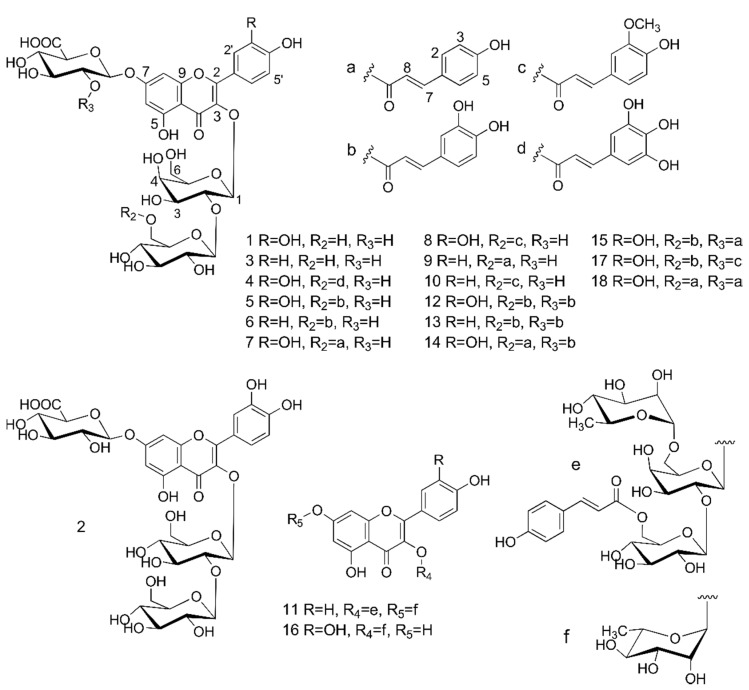
Structures of flavonol glycosides isolated from the aerial parts of lentil (*Lens culinaris*).

A preliminary UHPLC-MS/MS analysis of methanol extract from lentil aerial parts revealed the presence of numerous phenolic compounds (Figure 1) having flavonoid-like UV spectra. They were tentatively identified as kaempferol and quercetin glycosides, most of them acylated with phenolic acids. Three-step chromatographic separation of the extract led to the isolation of 18 flavonoids, including all major and several minor compounds (Figure 2). Their structures were determined on the basis of UV-VIS, ESI-MS/MS, HRESI-MS, and NMR analyses.

NMR spectroscopy data included various 1D [^1^H, proton-decoupled ^13^C and DEPT-135, selective excitation 1D-TOCSY and 1D-ROESY (mixing times of 120 and 250 ms respectively)] and 2D [^1^H-^1^H gCOSY (magnitude mode), ^1^H-^1^H TOCSY, ^1^H-^1^H ROESY, ^1^H-^13^C *g*HSQC, ^1^H-^13^C *g*HSQC-TOCSY (mixing time of 80 ms) and ^1^H-^13^C *g*HMBC (^n^*J*_CH_ = 8 Hz)] spectra (Figures S1–S193). Hydrolysis of the purified flavonoids, followed by the determination of absolute configuration of monosaccharides showed that these were D-Glc, D-Gal, and D-GlcA for compounds **1**, **3**–**10**, **12**–**15**, **17** and **18**, while **11** contained D-Glc, D-Gal, and L-Rha (see Supplementary Figures S211–S226). Analyses of the remaining products of alkaline and acid hydrolyses of these flavonoids confirmed the identity of their aglycones and constituent phenolic acids (see Supplementary Figures S195–S209). In the case of the 3,4,5-trihydroxycinnamic acid, present in the compound **4**, an analytical standard was not available and the compound was tentatively identified on the base of its molecular mass. We could not find any precise literature data about its UV maxima, but the UV spectrum we obtained (see Supplementary Figure S196) was similar to one presented in the work of Kopycki *et al.* [16].

Compounds **1**–**3** had UV spectra typical for flavonol 3-*O*-glycosides, and negative ESI-MS analyses showed that their deprotonated molecules had *m/z* values of 801, 801, and 785, respectively. The collision-induced dissociation (CID) of these precursor ions revealed that they share the same fragmentation pattern. In the case of compounds **1** and **2**, MS/MS spectra showed the presence of fragment ions of *m/z* 625 [(M−H)−176]^−^, suggesting the loss of a hexuronic acid, and *m/z* 300 [(M−H)−176−325]^−^, formed after the additional loss of the pair of hexoses and corresponding to the quercetin radical ion [Y_0_−H]^−^·. Similarly, compound **3** fragmented to ions of *m/z* 609 [(M−H)−176]^−^ (the loss of an hexuronic acid), and *m/z* 285 [(M−H)−176−324]^−^ (the additional loss of a dihexose moiety), corresponding to the kaempferol ion Y_0_^−^. A precise structure elucidation was possible after 1D and 2D-NMR analyses of these compounds. The ^13^C-NMR spectrum of **1** showed 33 signals, sorted by ^13^C and DEPT-135 experiments into 2 CH_2_, 20 CH and 11 quaternary carbon atoms. The aromatic region of the ^1^H and COSY spectra of **1** exhibited the presence of two sets of aromatic protons, characteristic for the quercetin aglycone (Table 1). One set corresponded to a tetrasubstituted aromatic ring with two meta-coupling protons and appeared at δ_H_ 6.77 (*d*, *J* = 1.6 Hz, H-8) and 6.50 (*d*, *J* = 1.5 Hz, H-6), which were correlated in the HSQC spectrum with their aromatic carbon atoms at δ_C_ 95.8 and 100.7 ppm, respectively. The other set corresponded to 3,4-dihydroxyphenyl group at δ_H_ 7.80 (*d*, *J* = 1.9 Hz, H-2'), 7.62 (*dd*, *J* = 8.4, 1.8 Hz, H-6'), and 6.91 (*d*, *J* = 8.4 Hz, H-5'), in accordance with AMX system of ring B of the aglycone. The assignments of all carbons of the flavonol moiety were accomplished by interpretation of the HSQC and HMBC spectra. Observed heteronuclear multiple bond connectivity (HMBC) correlations from H-2' and H-6’ to C-2, *^4^J* correlation from H-8 to C-4 and downfield shifted resonance at δ_C_ 159.4 for C-2, indicated that the compound **1** contained 3-O substituted quercetin. This was further supported by the result of UHPLC analysis of non-polar products of acid hydrolysis of **1**. The carbohydrate region of ^1^H NMR spectrum showed the presence of the oxymethine protons in the range δ 3.36–4.14. Moreover, three anomeric proton signals at δ_H_ 5.35 (*d*, *J* = 7.6 Hz, H-1_Gal_), 4.79 (*d*, *J* = 7.1 Hz, H-1_Glc_) and 5.20 (*m*, H-1_GlcA_) were also observed, indicating the presence of three sugar units. Based on the values of coupling constants (*J* > 7 Hz), and the analysis of ^1^H-, ^13^C-NMR spectra, and 1D TOCSY, COSY, TOCSY, HSQC, HSQC-TOCSY and HMBC data, the three sugar units were elucidated as β-galactopyranoside δ_H/C_ 5.35 (H-1_Gal_)/101.6 (C-1_Gal_), β-glucopyranoside δ_H/C_ 4.79 (H-1Glc)/105.1 (C-1_Glc_) and β-glucuropyranoside δ_H/C_ 5.20 (H-1_GlcA_)/101.4 (C-1_GlcA_) (Table 2). It was observed that the glucuropyranosyl moiety demonstrated non-first order ^1^H-NMR spectrum, which was not the result of impurities or other physical factors. In our opinion it was caused by the NMR phenomenon called virtual coupling [17].

**Table 1:** NMR spectroscopic data (methanol-*d*_4_, 500 MHz) for the aglycones of compounds **1**, **3**–**15**, **17** and **18**.

	1		3		4		5		6		7		8		9	
	δ_H_ (*J* in Hz)	δ_C_	δ_H_ (*J* in Hz)	δ_C_	δ_H_ (*J* in Hz)	δ_C_	δ_H_ (*J* in Hz)	δ_C_	δ_H_ (*J* in Hz)	δ_C_	δ_H_ (*J* in Hz)	δ_C_	δ_H_ (*J* in Hz)	δ_C_	δ_H_ (*J* in Hz)	δ_C_
2		159.4		159.4		158.4		158.6		158.8		158.6		158.6		158.9
3		135.4		135.2		135.4		135.4		135.3		135.4		135.4		135.3
4		180.0		180.0		180.1		180.1		180.2		180.1		180.1		180.3
5		162.8		162.9		162.4		162.6		162.6		162.6		162.6		162.6
6	6.50 d (1.5)	100.7	6.51 d (2.4)	100.8	6.36 d (2.1)	100.7	6.41d(2.2)	100.5	6.40d(2.2)	100.6	6.41 d (2.3)	100.6	6.39 d (2.1)	100.5	6.37 d (2.1)	100.6
7		164.3		164.4		164.4	-	164.1		164.1		164.0		164.0		164.0
8	6.77 d (1.6)	95.8	6.80 d (2.5)	95.7	6.47 d (2.1)	95.5	6.48d(2.2)	95.7	6.51d(2.1)	95.8	6.48 d (2.3)	95.7	6.42 d (2.1)	95.6	6.49 d (2.1)	95.7
9		157.9		158.0		157.6		157.6		157.6		157.6		157.5		157.6
10		107.6		107.7		107.4		107.5		107.5		107.5		107.4		107.5
1'		122.7		122.6		122.6		122.6		122.2		122.5		122.5		122.2
2'	7.80 d (1.9)	117.9	8.16 d (8.3)	132.6	7.69 ^a^	117.5	7.72 d(1.4)	117.5	8.15d (8.8)	132.8	7.73 d (2.3)	117.5	7.69 d (2.2)	117.5	8.12 d (8.9)	132.8
3'		145.9	6.94 d (8.3)	116.3		145.9		146.0	6.91d(8.7)	116.4		145.9		146.0	6.88 d (8.9)	116.4
4'		150.0		161.8		149.9		149.9	-	161.7		149.9		150.0		161.8
5'	6.91 d (8.4)	116.2			6.87 d (8.6)	116.4	6.91d(8.3)	116.3			6.91 d (8.5)	116.3	6.91 d (8.4)	116.3		
6'	7.62 dd(8.4, 1.8)	123.3			7.70 dd(9.1, 2.2)	124.1	7.68dd(8.8, 2.2)	124.0			7.72 dd(8.4, 2.3)	124.0	7.72dd(8.4, 2.1)	123.8		
2		158.8		158.9		158.6		158.8		158.6		158.6		158.6		158.6
3		135.3		135.3		135.4		135.3		135.4		135.4		135.4		135.4
4		180.3		180.2		180.0		180.2		180.1		180.0		180.0		180.1
5		162.5		162.6		162.6		162.6		162.7		162.6		162.6		162.7
6	6.35 d (2.1)	100.5	6.39 d (2.1)	100.7	6.28 d (1.6)	100.7	6.28 d (1.9)	100.7	6.28 d (1.9)	100.6	6.28 d (1.6)	100.6	6.28 d (1.2)	100.6	6.29 d (2.1)	100.6
7		164.1	-	163.5		163.7		163.7		163.7		163.6		163.7		163.7
8	6.43, d (2.1)	95.6	6.51 d (2.1)	95.6	6.42 d (1.5)	95.7	6.44 d (1.9)	95.8	6.39 d (1.8)	95.7	6.41 d (1.6)	95.7	6.42 d (1.6)	95.7	6.39 d (2.1)	95.7
9		157.5		157.7		157.5		157.6		157.6		157.5		157.5		157.5
10		107.4		107.2		107.7		107.7		107.7		107.7		107.7		107.7
1'		122.1		122.1		122.6		122.2		122.5		122.5		122.6		122.5
2'	8.11 d (8.9)	132.9	8.12 d (8.9)	132.9	7.69 d (2.1)	117.6	8.09 d (8.8)	132.8	7.68 d (1.5)	117.5	7.69 d (2.3)	117.6	7.69 d (2.1)	117.5	7.68 ^a^	117.5
3'	6.89 d (8.9)	116.4	6.89 d (8.8)	116.4		145.9	6.86 d (8.8)	116.4		146.0		145.9		145.9		146.0
4'		161.8		161.8		150.0		161.7		150.0		149.9		150.0		150.0
5'					6.86 d (8.4)	116.4			6.86 d (9.0)	116.4	6.86 d (8.4)	116.3	6.86 d (8.4)	116.3	6.86 d (9.0)	116.4
6'					7.66 dd (8.4, 2.1)	124.0			7.69 dd (9.3,2.4)	124.1	7.65 dd (8.4,2.3)	124.0	7.66 dd (8.5, 2.0)	124.0	7.69 ^a^	124.1

^a^: overlapping signals.

**Table 2:** NMR spectroscopic data (methanol-*d*_4_, 500 MHz) for the sugar units of compounds **1** and **3**.

	1		3	
	δ_H_ (*J* in Hz)	δ_C_	δ_H_ (*J* in Hz)	δ_C_
	7-*O*-β-GlcA		7-*O*-β-GlcA	
1	5.20 m	101.4	5.20 m	101.4
2	3.56 ^a^	74.4	3.56 ^a^	74.4
3	3.57 ^a^	77.1	3.57 ^a^	77.2
4	3.67 m	72.8	3.67 ^a^	72.9
5	4.14 d (9.6)	76.6	4.14 d (9.6)	76.6
6		172.0		172.1
	3-*O*-β-Gal		3-*O*-β-Gal	
1	5.35 d (7.6)	101.6	5.44 d (7.3)	101.3
2	4.09 dd (9.0, 8.0)	80.8	4.07 dd (9.4, 7.7)	80.5
3	3.73 dd (8.5, 4.0)	74.9	3.75 dd (9.7, 3.4)	74.9
4	3.88 d (2.4)	70.1	3.87 d (3.3)	70.1
5	3.47 ^a^	77.1	3.47 t (6.2)	77.1
6	3.63 m	62.0	3.62 m3.56 m	62.1
	3.58 m		3.56 m	
	2Gal-*O*-β-Glc		2Gal-*O*-β-Glc	
1'	4.79 d (7.1)	105.1	4.78 d (6.9)	104.9
2'	3.44 m	75.5	3.39 ^a^	75.6
3'	3.44 t (8.0)	77.9	3.42 ^a^	77.9
4'	3.45 ^a^	71.0	3.42 ^a^	71.3
5'	3.36 ddd (11.2, 4.8, 2.5)	78.0	3.33 m	78.2
6'	3.83 dd (11.7, 1.9)	62.3	3.81 dd (11.9, 2.0)	62.6
	3.74 dd (11.8, 4.5)		3.71 dd (11.8, 4.5)	

^a^: overlapping signals.

Typically it occurs when the chemical shift difference between two *J* coupled nuclei is of the same order as the coupling constant. It is said that this type of coupling shows dependence on solvent and field strength. Other factors influencing the complexity of the spectrum are both steric and electronic contributions. As it is shown later in this paper, other isolated compounds, substituted in either C-2_GlcA_ or C-6_Glc_ (or both) do not show the phenomenon of virtual coupling, but it is still visible in the GlcA of compound **3**. The long range correlations observed in the HMBC spectrum between the anomeric proton of the glucose (δ_H_ 4.79, H-1_Glc_) and C-2 of galactose (δ_C_ 80.8) indicated the presence of interglycosidic linkage between these hexosyl units (1→2). This was further supported by the NOE effect detected in the rotating frame nuclear Overhauser effect spectroscopy spectrum (ROESY) between H-1_Glc_ and H-2_Gal_. The 3-*O* glycosidation site was determined mainly by NOE effect in ROESY spectrum between the anomeric proton of galactopyranoside (δ_H_ 5.35, H-1_Gal_) and ring B (δ_H_ 7.80, H-2' and δ_H_ 7.62, H-6') of the quercetin moiety. The other, indirect evidence, was the downfield shifted resonance at δ_C_ 159.4 for C-2, as mentioned earlier. The correlation observed in the HMBC spectrum from anomeric proton at δ_H_ 5.20 (H-1_GlcA_) to carbon C-7 (δ_C_ 164.3) and the NOE effect visible in the ROESY spectrum between H-1_GlcA_ and H-6/8 indicated that the point of attachment of β-glucuropyranosyl unit to quercetin was at C-7 position. Therefore, the compound **1** was identified as quercetin 3-*O*-β-D-glucopyranosyl(1→2)-β-D-galactopyranoside-7-*O*-β-D-glucuropyranoside. Since the compound has not been reported before, we propose to name it lensoside A.

This scheme of glycosidation, along with the type of sugar moieties, was observed in all isolated compounds, except for molecules **2**, **11** and **16**. Furthermore, on the basis of NMR spectra and the results of UHPLC analyses of non-polar products of acid hydrolysis, we established that the quercetin nucleus was also present in compounds **2**, **4**, **5**, **7**, **8**, **12**, **14**, **15**, **16**, **17** and **18**, which will be discussed later.

Compound **2** was identified, on the base of its fragmentation pattern and NMR spectrum, as quercetin 3-*O*-β-sophoroside-7-*O*-β-glucuronide, previously isolated from epidermis of onion (*Allium cepa*) [18].

The ^13^C-NMR spectrum of **3**, like **1**, showed 33 signals, sorted by ^13^C and DEPT-135 experiments into two CH_2_, 21 CH and 10 quaternary carbon atoms. The aromatic region of the ^1^H and COSY spectra of **3** exhibited the presence of two sets of aromatic protons. One set corresponded to a tetrasubstituted aromatic ring with two *meta*-coupling protons and appeared at δ_H_ 6.80 (*d*, *J* = 2.5 Hz, H-8) and 6.51 (*d*, *J* = 2.4 Hz, H-6), which were correlated in the HSQC spectrum with their aromatic carbon atoms at δ_C_ 95.7 and 100.8 ppm, respectively. The other set, characteristic for AA’XX’ system, corresponded to *p*-hydroxyphenyl group at δ_H_ 8.16 (*d*, *J* = 8.3 Hz, H-2'/6') and 6.94 (*d*, *J* = 8.3 Hz, H-3'/5'), in accordance with the kaempferol nucleus (Table 1). The result of UHPLC analysis of non-polar products of acid hydrolysis of **3** confirmed this assignment. The carbohydrate region of ^1^H-NMR spectrum showed the presence of the oxymethine protons in the range δ 3.33–4.14. Moreover, three anomeric proton signals at δ_H_ 5.44 (*d*, *J* = 7.3 Hz, H-1_Gal_), 4.78 (*d*, *J* = 7.1 Hz, H-1_Glc_) and 5.20 (*m*, H-1_GlcA_) were also observed, thus indicating the presence of three sugar units. On the base of the values of coupling constants (*J* > 7 Hz), the analysis of ^1^H-, ^13^C-NMR spectra, and 1D TOCSY, COSY, TOCSY, HSQC, HSQC-TOCSY and HMBC data, the three sugar units were identified as β-galactopyranoside δ_H/C_ 5.44 (H-1_Gal_)/101.3 (C-1_Gal_), β-glucopyranoside δ_H/C_ 4.78 (H-1_Glc_)/104.9 (C-1_Glc_) and β-glucuropyranoside δ_H/C_ 5.20 (H-1_GlcA_)/101.4 (C-1_GlcA_) (Table 2). As in the case of the compound **1**, the glucuropyranosyl moiety demonstrated non-first order ^1^H NMR spectrum caused by virtual coupling. The long range correlations observed in the HMBC spectrum between the anomeric proton of the glucose (δ_H_ 4.78, H-1_Glc_) and C-2 of galactose (δ_C_ 80.5) indicated the interglycosidic linkage between these hexosyl units (1→2). This, along with the correlations observed in the HMBC spectrum from anomeric protons at δ_H_ 5.44 (H-1_Gal_) to carbon C-3 (δ_C_ 135.2), δ_H_ 5.20 (H-1_GlcA_) to carbon C-7 (δ_C_ 164.3) and the NOE effect visible in the ROESY spectrum between H-1_Gal_ and H-2'/6' together with NOE effect between H-1_GlcA_ and H-6/8 indicated that the points of glycosidation were identical as in **1**. Therefore, **3** was identified as kaempferol 3-*O*-β-D-glucopyranosyl(1→2)-β-D-galactopyranoside-7-*O*-β-D-glucuropyranoside, named lensoside B. Furthermore, it was established on the basis of NMR spectra and the results of UHPLC analysis of non-polar products of acid hydrolysis that the kaempferol nucleus was also present in compounds **6**, **9**, **10**, **11**, and **13**, which will be discussed later.

Compounds **4**–**10** form another group with a common structural scheme. Their UV spectra were characterized by a distinct (18–36 nm) hypsochromic shift in Band I, as compared to compounds **1**–**3**, indicating that they might be acylated with phenolic acids. The following [M−H]^−^ ions were detected during ESI-MS analyses of these flavonoids: *m/z* 979, 963, 947, 947, 977, 931 and 961, respectively. The precursor ion of compound **4** (*m/z* 979) gave fragment ions of *m/z* 803 [(M−H)−176]^−^ after the loss of a hexuronic acid, *m/z* 625 [(M−H)−176−178]^−^, which could indicate the presence of trihydroxy-cinnamic acid, and *m/z* 300 [(M−H)−176−178−325]^−^, after the additional loss of dihexose, corresponding to the quercetin radical ion [Y_0_−H]^−^·.

Compounds **5**, **7**, and **8** also seemed to be acylated quercetin derivatives, with the same fragmentation pattern (the loss of 176 mu at the first stage of fragmentation and the presence of the ions of *m/z* 625 and *m/z* 300 were always observed), differing only in their putative phenolic acid moieties: caffeic (**5**), *p*-coumaric (**7**) and ferulic acid (**8**). The ESI-MS/MS analysis of the compounds **6**, **9**, and **10** showed that they were acylated derivatives of kaempferol. The precursor ion of compound **6** (*m/z* 947) fragmented into ions of *m/z* 771 [(M−H)−176]^−^ (the loss of a hexuronic acid), *m/z* 609 [(M−H)−176−162]^−^ (indicating a putative loss of caffeic acid), and *m/z* 284 [(M−H)−176−162−325]^−^ (the additional loss of dihexose), corresponding to the kaempferol radical ion [Y_0_−H]^−^·. Compounds **9** and **10** had the same fragmentation scheme, and differed only in their putative phenolic acid groups: *p*-coumaric and ferulic acid, respectively.

The UV and MS spectra, as well as the higher retention times of these flavonoids clearly suggested that they might be acylated forms of compounds **1**–**3**. NMR analyses confirmed this conclusion. The ^13^C-NMR spectrum of the compound **4** showed 42 signals, sorted by ^13^C and DEPT-135 experiments into two CH_2_, 24 CH and 16 quaternary carbon atoms. Assignment of glucosidic protons systems and sites of glycosylation was achieved by analysis of 1D TOCSY, 1D ROESY, COSY, HSQC and HMBC experiments. The ^1^H-NMR spectrum of **4** contained resonances typical for the quercetin nucleus as expected (Table 1), but also a set of coupled doublets *E*-α-H and β-H at δ_H_ 7.13 and 5.85 corresponding to *E*-(*J*_α,β_ = 15.8 Hz) olefinic moiety and a downfield shifted aromatic singlet at δ_H_ 6.23 (2H) correlated in the HSQC spectrum with its aromatic carbon atom at δ_C_ 108.5. The long-range correlations observed in HMBC spectrum suggested that this is a *E*-3,4,5-trihydroxycinnamoyl moiety. For the Glc residue H-6 protons and C-6 carbon were downfield shifted to δ_H_ 4.41 (*m*, 2H) and δ_C_ 64.8, espectively (Table 3). Additionally, protons H-6_Glc_ exhibited ^3^*J* correlation in the HMBC spectrum with a carbonyl group resonated at δ_C_ 167.5, corresponding to C-9_triOHCin_ (COO^−^). Therefore, **4** was a monoacylated derivative of **1**, established as quercetin 3-*O*-[(6-*O*-*E*-3,4,5-trihydroxycinnamoyl)-β-D-glucopyranosyl(1→2)]-β-D-galactopyranoside-7-*O*-β-D-glucuropyranoside, named lensoside Aα.

Compounds **5**, **7** and **8**, similarly to **4**, were monoacylated with phenolic acids. All of them shared the same basic skeleton, identical with **1**, which was confirmed with COSY, ROESY, HSQC and HMBC spectra along with selective experiments (1D TOCSY and 1D ROESY) used for the determination of nature of sugar moieties. The aromatic region of the ^1^H and COSY spectra of **5** contained sets of resonances characteristic for *E*-(*J*_α,β_ = 15.9 Hz) olefinic moiety and ABX system corresponding to a 3,4-dihydroxyphenyl group at δ_H_ 6.75 (*d*, *J* = 1.9 Hz, H-2_Caf_), 6.62 (*d*, *J* = 8.3 Hz, H-5_Caf_), and 6.59 (*dd*, *J* = 8.2, 1.9 Hz, H-6_Caf_) and it was identified as *E*-caffeoyl group. Likewise, the aromatic region of the ^1^H and COSY spectra of **7** contained one pair of *E*-α-H and β-H doublets (δ_H_ 6.02 and 7.35 with *J*_α,β_ = 15.9 Hz), but it also exhibited a AA’XX’ system corresponding to *p*-hydroxyphenyl group at δ_H_ 7.12 (*d*, *J* = 8.1 Hz, H-2/6_Cou_) and 6.67 (*d*, *J* = 8.1 Hz, H-3/5_Cou_), which was interpreted as *E-p*-coumaroyl moiety in turn.

**Table 3:** NMR spectroscopic data (methanol-*d*_4_, 500 MHz) for the sugar and phenolic acid units of compounds **4**–**10**.

	4		5		6		7			8		9		10	
	δ_H_ (*J* in Hz)	δ_C_	δ_H_ (*J* in Hz)	δ_C_	δ_H_ (*J* in Hz)	δ_C_	δ_H_ (*J* in Hz)	δ_C_		δ_H_ (*J* in Hz)	δ_C_	δ_H_ (*J* in Hz)	δ_C_	δ_H_ (*J* in Hz)	δ_C_
	7-*O*-β-GlcA		7-*O*-β-GlcA		7-*O*-β-GlcA		7-*O*-β-GlcA			7-*O*-β-GlcA		7-*O*-β-GlcA		7-*O*-β*-*GlcA	
1	5.12 d (7.6)	101.2	5.17 d (7.3)	101.3	5.19 d (7.2)	101.2	5.15 d (6.8)	101.2		5.14 d (7.4)	101.4	5.14 d (7.2)	101.2	5.13 d (7.4)	101.3
2	3.53 dd (9.0, 7.4)	74.5	3.57 t (7.5)	74.4	3.58 dd (9.1, 7.3)	74.5	3.57 dd (9.3, 6.8)	74.4		3.57 dd (9.2, 7.2)	74.4	3.55 t (9.3)	74.4	3.55 dd (9.2, 7.1)	74.4
3	3.59 t (9.1)	77.5	3.61 t (9.0)	77.1	3.62 t (9.0)	77.2	3.61 t (9.1)	77.1		3.62 t (9.0)	77.1	3.58 t (9.1)	77.1	3.59 t (8.6)	77.1
4	3.56 t (9.2)	73.4	3.66 t (9.3)	72.9	3.67 t (9.2)	73.0	3.66 t (8.0)	72.9		3.67 t (9.5)	72.9	3.65 t (9.5)	72.9	3.64 t (9.1)	72.9
5	3.94 d (8.9)	76.3	4.13 d (9.5)	76.5	4.13 d (9.4)	76.5	4.14 d (9.5)	76.5		4.13 d (9.5)	76.5	4.12 d (9.6)	76.4	4.12 d (9.5)	76.5
6		ND		172.3		172.6		172.3			172.3		172.3		172.3
	3*-O*-β-Gal		3-*O*-β-Gal		3-*O*-β-Gal		3-*O*-β-Gal			3-*O*-β-Gal		3-*O*-β-Gal		3-*O*-β-Gal	
1	5.10 d (8.1)	101.3	5.20 br d (7.6)	101.3	5.06 br d (7.4)	101.4	5.19 br d (7.2)	101.4		5.11 br d (7.4)	101.3	5.01 br d (7.5)	101.4	4.94 br d (7.4)	101.5
2	3.98 dd (9.6, 7.6)	83.5	4.01 dd (9.6, 7.5)	83.3	4.00 dd (9.5, 7.5)	83.4	4.03 dd (9.6, 7.5)	83.2		4.01 dd (9.6, 7.6)	83.5	3.98 dd (9.5, 7.6)	83.3	3.96 dd (9.5, 7.6)	83.5
3	3.66 dd (9.7, 3.4)	74.7	3.71 dd (9.6, 3.3)	74.7	3.68 dd (10.2, 3.8)	74.8	3.70 dd (9.3, 3.3)	74.7		3.69 dd (9.0, 3.6)	74.8	3.65 dd (9.7, 2.8)	74.8	3.63 dd (9.5, 3.8)	74.8
4	3.79 d (3.5)	70.1	3.83 d (3.3)	70.1	3.81 d (3.5)	70.1	3.84 d (3.2)	70.1		3.82 d (3.7)	70.1	3.78 d (3.3)	70.1	3.77 d (3.3)	70.1
5	3.34 ^a^	76.9	3.40 t (5.6)	76.9	3.37 t (6.1)	76.9	3.41 t (5.9)	76.9		3.38 t (6.0)	76.9	3.37 t (6.1)	76.9	3.32 t (6.1)	76.9
6	3.55 ^a^	61.7	3.59 dd (11.2, 5.7)	61.8	3.59 dd (11.2, 5.8)	61.8	3.60 dd (11.3, 5.7)	61.8		3.59 dd (11.0, 4.2)	61.8	3.57 dd (11.3, 5.8)	61.8	3.55 dd (11.2, 5.8)	61.8
	3.48 ^a^		3.52 dd (11.0, 6.2)		3.50 dd (11.3, 6.4)		3.53 dd (11.3, 6.3)			3.50 dd (11.0, 5.0)		3.48 dd (11.2, 6.4)		3.45 dd (11.5, 6.2)	
	2Gal-*O*-β-Glc		2Gal-*O*-β-Glc		2Gal-*O*-β-Glc		2Gal-*O*-β-Glc			2Gal-*O*-β-Glc		2Gal-*O*-β-Glc		2Gal-*O*-β-Glc	
1'	4.70 d (7.6)	106.8	4.75 d (7.5)	106.6	4.72 d (7.6)	106.7	4.77 d (7.4)		106.5	4.76 d (7.6)	106.7	4.70 d (7.7)	106.6	4.69 d (7.7)	106.8
2'	3.42 dd (9.4, 7.6)	76.3	3.46 dd (9.4, 7.4)	76.2	3.43 dd (9.3, 7.9)	76.3	3.48 dd (9.3, 7.6)		76.1	3.47 t (8.6)	76.3	3.41 dd (9.0, 8.0)	76.2	3.41 dd (9.2, 7.8)	76.3
3'	3.48 dd (9.3, 8.6)	77.8	3.51 t (9.2)	77.8	3.52 t (9.2)	77.8	3.53 t (9.2)		77.8	3.54 t (8.7)	77.8	3.49 t (9.1)	77.8	3.50 t (9.1)	77.8
4'	3.33 dd (9.7, 8.5)	72.4	3.39 t (8.8)	72.1	3.39, t (9.2)	72.2	3.40 dd (9.7, 8.4)		72.0	3.40 t (8.7)	72.2	3.39 t (9.4)	72.1	3.37 dd (9.5, 8.7)	72.3
5'	3.74 ddd(9.8, 6.3, 3.6)	75.6	3.75 ddd(9.7, 6.5, 3.0)	75.7	3.70 ddd(9.7, 6.6, 2.9)	75.6	3.74 ddd(9.8, 6.1, 3.5)	75.7		3.79 ddd(9.5, 7.6, 2.3)	75.6	3.68 ddd(9.5, 5.6, 3.8)	75.6	3.70 ddd(9.7, 7.2, 2.5)	75.5
6'	4.41 m (2H)	64.8	4.43 m (2H)	64.8	4.45 dd (11.9, 6.7))	64.9	4.44 m (2H)	64.8		4.49 dd (11.8, 7.1)	64.8	4.42 m (2H)	64.9	4.47 dd (11.8, 7.2)	64.9
					4.42 dd (11.9, 3.0)					4.44 dd (11.7, 2.4)				4.41 dd (11.7, 2.3)	
	6Glc-*O*-triOHCin		6Glc-*O*-Caf		6Glc-*O*-Caf		6Glc-*O*-Cou			6Glc-*O*-Fer		6Glc-*O*-Cou		6Glc-*O*-Fer	
1		126.3		127.4		127.3			126.8		127.3		126.7		127.2
2	6.23 s	108.5	6.75 d (1.9)	114.9	6.71 d (1.9)	114.8	7.12 d (8.1)		130.8	6.76 d (1.8)	110.8	7.06 d(8.6)	130.7	6.71 d (1.6)	110.7
3		147.1		146.4		146.4	6.67 d (8.1)		116.7		148.9	6.62 d (8.6)	116.7		148.9
4		137.7		149.2		149.2			160.8		150.1		160.8		150.1
5		147.1	6.62 d (8.3)	116.4	6.60 d (8.0)	116.4	6.67 d (8.1)		116.7	6.65 d (8.1)	116.3	6.62 d (8.6)	130.7	6.60 d (8.1)	116.3
6	6.23 s	108.5	6.59 dd (8.2, 1.9)	122.7	6.56 dd (8.0, 1.9)	122.6	7.12 d (8.1)		130.8	6.70 dd (8.2, 1.8)	124.1	7.06 d (8.6)	116.7	6.65 dd (8.2, 1.7)	123.8
7	7.13 d (15.8)	147.2	7.28 d (15.9)	146.8	7.25 d (15.9)	146.8	7.35 d (15.9)		146.4	7.31 d (15.9)	146.7	7.30 d (15.9)	146.4	7.27 d (15.9)	146.6
8	5.85 d (15.8)	114.5	5.95 d (15.9)	114.5	5.93 d (15.8)	114.5	6.02 d (15.9)		114.6	6.02 d (15.9)	114.8	5.98 d (15.9)	114.6	5.98 d (15.9)	114.8
9		167.5		168.9		168.9			168.9		168.9		168.9		168.8
OCH_3_										3.79 s	56.2			3.75 s	56.2

^a^: overlapping signals.

Compound **8** exhibited in the aromatic region of ^1^H and COSY spectra sets of resonances very similar to **5**. One set of resonances was characteristic for *E*- (*J*_α,β_ = 15.9 Hz) olefinic moiety and an ABX system corresponded to 3,4-dihydroxyphenyl group at δ_H_ 6.76 (*d*, *J* = 1.8 Hz, H-2_Fer_), 6.70 (*dd*, *J* = 8.2, 1.8 Hz, H-6_Fer_) and 6.65 (*d*, *J* = 8.1 Hz, H-5_Fer_). The feruloyl nature of the acyl group in **8** was confirmed by a long-range correlation in HMBC between the 3-OCH_3_ group at δ_H_ 3.79 (*s*, 3H) and an aromatic carbon C-3_Fer_ that resonated at δ_C_ 148.9. The site of methylation in fthe eruloyl group of **8** was further confirmed by the NOE effect visible in the ROESY spectrum between protons of the CH_3_ group and H-2_Fer_. Protons H-6_Glc_ in compounds **5**, **7**, **8** were downfield shifted to δ_H_ 4.43 (*m*, 2H), 4.44 (*m*, 2H) and 4.49 (*dd*, *J* = 11.8, 7.1 Hz) as well as 4.44 (*dd*, *J* = 11.7, 2.4 Hz) and exhibited correlations in the HMBC spectra with carbonyl carbons resonated at δ_C_ 168.9, 168.9 and 168.9 corresponding with C-9_Caf_, C-9_Cou_ and C-9_Fer_, respectively. Therefore, **5** was quercetin 3-*O*-[(6-*O-E*-caffeoyl)-β-D-glucopyranosyl(1→2)]-β-D-galactopyranoside-7-O-β-D-glucuropyranoside (named lensoside Aβ), **7** was quercetin 3-*O*-[(6-*O-E-p*-coumaroyl)-β-D-glucopyranosyl(1→2)]-β-D-galactopyranoside-7-*O*-β-D-glucuropyranoside(named lensoside Aγ) and compound **8** was quercetin 3-*O*-[(6-*O-E*-feruloyl)-β-D-glucopyranosyl(1→2)]-β-D-galactopyranoside-7-*O*-β-D-glucuropyranoside (named lensoside Aδ).

Compounds **6**, **9** and **10**, similarly to **4**, were monoacylated with phenolic acids. All of them shared the same basic skeleton, identical with **3**, which was confirmed with COSY, ROESY, HSQC and HMBC spectra along with selective experiments (1D TOCSY and 1D ROESY) used for the determination of nature of sugar moieties. Compounds **6**, **9** and **10** were kaempferol analogues of acylated quercetin glycosides **5**, **7** and **8**. The ^1^H-NMR spectra of **6**, **9** and **10** contained resonances for the protons of kaempferol, and ^1^H- and ^13^C-NMR chemical shift values for the glycosyl moieties were similar to those of **5**, **7** and **8**, respectively. The biggest differences, apart from the aglycone part, were noticed in Gal anomeric protons upfield shifted to δ_H_ 5.06, 5.01 and 4.94 in **6**, **9** and **10**, comparing to δ_H_ 5.20, 5.19 and 5.11 in **5**, **7** and **8**, respectively. Thus **6** was kaempferol 3-*O*-[(6-*O-E*-caffeoyl)-β-D-glucopyranosyl(1→2)]-β-D-galactopyranoside-7-*O*-β-D-glucuropyranoside (named lensoside Bα), **9** was kaempferol 3-*O*-[(6-*O-E-p*-coumaroyl)-β-D-glucopyranosyl(1→2)]-β-D-galactopyranoside-7-*O*-β-D-glucuropyranoside (named lensoside Bβ) and the compound **10** was kaempferol 3-*O*-[(6-*O-E*-feruloyl)-β-D-glucopyranosyl(1→2)]-β-D-galactopyranoside-7-*O*-β-D-glucuropyranoside (named lensoside Bγ).

The third group of structurally related flavonoids comprise compounds **12**–**15**, **17**, and **18**. Their UV spectra indicated that they were acylated with phenolic acids. [M−H]^−^ ions of these compounds were as follows: *m/z* 1125, 1109, 1109, 1109, 1139, 1093. The fragmentation of the [M−H]^−^ ion of flavonoid **12** comprised ions of *m/z* 963, indicating the loss of caffeic acid (a loss of hexose can be excluded, as the retention time of **12** is much higher than those of earlier described compounds), *m/z* 787 (the loss of a hexuronic acid), and the further fragmentation path was the same as for **5**. These facts indicate that **12** contains two caffeic acid moieties, and one of them is probably bound to the hexuronic acid. Similarly, the [M−H]^−^ ion of compound **13** fragmented to the ions of *m/z* 947, which indicated the loss of coumaric acid, *m/z* 771 (the loss of an hexuronic acid), and other fragments were the same as for the flavonoid **6**. Since the general pattern of fragmentation was similar for the remaining flavonoids of this group, it may be deduced that the compound **14** is a caffeoylated derivative of **7**, **15**—a coumaroylated derivative of **5**, **17**—a feruloylated derivative of **5**, and **18** is a coumaroylated derivative of **7**.

The ^13^C-NMR spectrum of compound **12** showed 51 signals, sorted by ^13^C and DEPT-135 experiments into two CH_2_, 30 CH and 19 quaternary carbon atoms. Assignment of glucosidic protons systems and sites of glycosylation was achieved by analysis of 1D TOCSY, 1D ROESY, COSY, HSQC and HMBC experiments (Table 4). The ^1^H-NMR spectrum of **12** contained resonances typical for the quercetin nucleus and the basic skeleton was identical with **1** (Table 1). The aromatic region of the ^1^H and COSY spectra of **12** contained two pairs of *E*- α-H and β-H doublets (δ_H_ 7.65 and 6.34 with *J*_α,β_ = 15.9 Hz; δ_H_ 7.23 and 5.90 with *J*_α,β_ = 15.9 Hz), and exhibited two separate AMX systems corresponded to 3,4-dihydroxyphenyl groups, which were interpreted as two *E*-caffeoyl moieties. Protons H-6_Glc_ were downfield shifted at δ_H_ 4.42 (*dd*, *J* = 12.1, 7.1 Hz) and 4.38 (*dd*, *J* = 12.0, 2.8 Hz) and exhibited correlations in the HMBC spectra with carbonyl carbon resonated at δ_C_ 169.0 corresponding with C-9_Caf_. The H-2_GlcA_ was downfield shifted to δ_H_ 5.16 (*dd*, *J* = 10.0, 7.5 Hz) and this resonance correlated to the carbonyl carbon C-9_Caf_ of the second *E*-caffeoyl moiety at δ_C_ 168.3 in the HMBC spectrum. Therefore, **12** was in fact acylated form of the compound **5**, quercetin 3-*O*-[(6-*O-E*-caffeoyl)-β-D-glucopyranosyl(1→2)]-β-D-galactopyranoside-7-*O*-(2-*O-E*-caffeoyl’)-β-D glucuropyranoside, named lensoside C.

Compound **13**, like **12**, was diacylated flavonol. It shared the same basic skeleton, identical with **3**, which was confirmed with COSY, ROESY, HSQC and HMBC spectra along with selective experiments (1D TOCSY and 1D ROESY) used for the determination of nature of sugar moieties. The compound **13** was kaempferol analogue of the acylated quercetin glycoside **12**. The ^1^H NMR spectrum of **13** contained resonances for the protons of kaempferol, and ^1^H and ^13^C-NMR chemical shift values for the glycosyl moieties were similar to those of **12**. Thus, **13** was kaempferol 3-*O*-[(6-*O-E*-caffeoyl)-β-D-glucopyranosyl(1→2)]-β-D-galactopyranoside-7-*O*-(2-*O-E*-caffeoyl’)-β-D-glucuropyranoside, named lensoside D.

Compounds **15** and **17**, were *p*-coumaroylated and feruloylated derivatives of compound **5**, respectively. The ^1^H-NMR spectrum of **15** exhibited a AA’XX’ system corresponding to *p*-hydroxyphenyl group at δ_H_ 7.44 (*d*, *J* = 8.5 Hz, H-2/6_Cou_) and 6.77 (*d*, *J* = 8.4 Hz, H-3/5_Cou_), which was interpreted as *E-p*-coumaroyl moiety. For the GlcA residue, H-2 was downfield shifted to δ_H_ 5.16 (*dd*, *J* = 9.2, 7.8 Hz), as in **12**, and this proton correlated to the carbonyl carbon C-9_Cou_ resonated at δ_C_ 168.2 in the HMBC spectrum. Likewise, in **17**, H-2_GlcA_ was downfield shifted to δ_H_ 5.16 (*t*, *J* = 8.7 Hz) and this proton correlated to the carbonyl carbon C-9_Fer_ resonated at δ_C_ 168.2 in the HMBC spectrum. Therefore, **15** was identified as quercetin 3-*O*-[(6-*O*-*E*-caffeoyl)-β-D-glucopyranosyl(1→2)]-β-D-galactopyranoside-7-*O*-(2-*O*-*E-p*-coumaroyl)-β-D-glucuropyranoside (named lensoside E) and **17** was quercetin 3-*O*-[(6-*O*-*E*-caffeoyl)-β-D-glucopyranosyl(1→2)]-β-D-galactopyranoside-7-*O*-(2-*O*-*E*-feruloyl)-β-D-glucuropyranoside (named lensoside F).

Compounds **14** and **18**, were caffeoylated and *p*-coumaroylated derivatives of compound **7**, respectively. In the ^1^H-NMR spectrum of 14, H-2_GlcA_ was downfield shifted to δ_H_ 5.16 (*dd*, *J* = 9.4, 7.7 Hz) and this proton correlated to the carbonyl carbon C-9_Caf_ resonated at δ_C_ 168.2 in the HMBC spectrum. The aromatic region of the ^1^H and COSY spectra of **18** exhibited two sets of a AA’XX’ system corresponding to *p*-hydroxyphenyl group at δ_H_ 7.04 (*d*, *J* = 8.6 Hz, H-2/6_Cou_) and 6.62 (*d*, *J* = 8.6 Hz, H-3/5_Cou_) as well as δ_H_ 7.45 (*d*, *J* = 8.7 Hz, H-2/6_Cou’_) and 6.77 (*d*, *J* = 8.7 Hz, H-3/5_Cou’_), which was interpreted as two *E-p*-coumaroyl moieties.

**Table 4:** NMR spectroscopic data (methanol-*d*_4_, 500 MHz) for the sugar and phenolic acid units of compounds **12**–**15**, **17** and **18**.

	12		13		14		15		17		18	
	δ_H_ (*J* in Hz)	δ_C_	δ_H_ (*J* in Hz)	δ_C_	δ_H_ (*J* in Hz)	δ_C_	δ_H_ (*J* in Hz)	δ_C_	δ_H_ (*J* in Hz)	δ_C_	δ_H_ (*J* in Hz)	δ_C_
	7-*O*-β-GlcA		7-*O*-β-GlcA		7-*O*-β-GlcA		7-*O*-β-GlcA		7-*O*-β-GlcA		7-*O*-β-GlcA	
1	5.38 d (7.8)	99.6	5.41 d (7.9)	99.6	5.37 d (7.9)	99.6	5.40 d (7.7)	99.6	5.38 d (7.8)	99.7	5.38 d (7.9)	99.6
2	5.16 dd (10.0, 7.5)	74.5	5.16 dd (9.4, 7.9)	74.5	5.16 dd (9.4, 7.7)	74.5	5.16 dd (9.2, 7.8	74.5	5.16 t (8.7)	74.6	5.16 dd (9.2, 8.1)	74.5
3	3.84 t (9.0)	75.5	3.83 t (9.7)	75.4	3.81 t (9.1)	75.6	3.85 t (9.2)	75.4	3.84 t (8.5)	75.5	3.82 t (9.3)	75.5
4	3.75 br s	73.2	3.76 t (9.4)	73.1	3.74 br t (8.7)	73.2	3.77 br t (8.6)	73.1	3.76 br s	73.2	3.75 t (9.0)	73.1
5	4.14 br s	76.6	4.17 d (9.4)	76.5	4.11 br d (7.7)	76.6	4.18 br d (6.0)	76.5	4.14 br s	76.7	4.13 d (9.3)	76.6
6		173.2		172.7		173.2		172.3		ND		172.7
	3-*O*-β-Gal		3-*O*-β-Gal		3-*O*-β-Gal		3-*O*-β-Gal		3-*O*-β-Gal		3-*O*-β-Gal	
1	5.14 d (7.7)	101.3	5.02 d (7.5)	101.3	5.09 d (7.4)	101.4	5.16 d (7.6)	101.3	5.14 d (7.7)	101.3	5.10 d (7.6)	101.4
2	3.97 dd (9.4, 7.7)	83.4	3.95 dd (9.5, 7.6)	83.4	3.98 dd (9.5, 7.6)	83.4	3.98 dd (9.5, 7.6)	83.2	3.97 dd (9.4, 7.7)	83.3	3.98 dd (9.5, 7.6)	83.4
3	3.66 dd (9.6, 3.2)	74.7	3.63 dd (9.6, 3.1)	74.8	3.65 dd (9.7, 3.4)	74.7	3.67 dd (9.8, 3.2)	74.7	3.66 dd (9.6, 3.2)	74.7	3.66 dd (9.6, 3.4)	74.7
4	3.79 d (3.8)	70.1	3.77 d (3.2)	70.1	3.78 d (3.2)	70.1	3.80 d (2.2)	70.0	3.79 d (3.7)	70.1	3.78 d (3.2)	70.1
5	3.35 t (5.6)	76.9	3.32 t (5.7)	76.9	3.34 t (6.1)	76.9	3.36 t (5.9)	76.9	3.35 ^a^	76.9	3.34 t (6.0)	76.9
6	3.54 dd (11.3, 5.8)	61.8	3.54 dd (11.3, 5.7)	61.8	3.54 dd (11.2, 5.7)	61.8	3.55 dd (11.3, 5.7)	61.8	3.54 dd (11.4, 5.8)	61.8	3.54 dd (11.3, 5.8))	61.8
	3.46 dd (11.5, 6.6)		3.47 dd (10.7, 7.0)		3.45 dd (11.3, 6.5)		3.46 dd (11.3, 6.4)		3.46 dd (11.7, 6.5)		3.45 dd (11.1, 6.3)	
	2Gal-*O*-β-Glc		2Gal-*O*-β-Glc		2Gal-*O*-β-Glc		2Gal-*O*-β-Glc		2Gal-*O*-β-Glc		2Gal-*O*-β-Glc	
1'	4.71 d (7.5)	106.6	4.67 d (7.7)	106.7	4.72 d (7.6)	106.7	4.72 d (7.5)	106.5	4.71 d (7.5)	106.6	4.71 d (7.5)	106.6
2'	3.42 dd (9.6, 7.5)	76.2	3.39 dd (9.3, 7.8)	76.3	3.42 dd (9.5, 7.5)	76.2	3.43 dd (9.5, 7.4)	76.1	3.42 dd (9.6 ,7.5)	76.2	3.42 dd (9.6 ,7.5)	76.2
3'	3.48 t (9.0)	77.8	3.48 t (9.1)	77.8	3.48 t (9.0)	77.8	3.48 t (9.1)	77.7	3.47 t (9.1)	77.8	3.47 t (9.1)	77.8
4'	3.34 dd (9.9, 8.3)	72.2	3.35 dd (9.8, 8.5)	72.2	3.35 dd (9.6, 8.5)	72.2	3.35 t (8.7)	72.1	3.34 dd (9.8, 8.1)	72.2	3.34 dd (9.8, 8.1)	72.2
5'	3.72 ddd (9.6, 7.0, 3.2)	75.7	3.67 ddd (9.5, 6.9, 2.4)	75.6	3.72 td (9.5, 4.8)	75.7	3.72 ddd (9.4, 6.9, 2.4)	75.7	3.72 ddd (9.7, 6.5, 3.2)	75.7	3.72 ddd (9.7, 6.5, 3.2)	75.7
6'	4.42 dd (12.1, 7.1)	64.9	4.42 dd (11.8, 7.0)	64.9	4.40 d (4.8) (2H)	64.8	4.42 dd (12.0, 6.8)	64.8	4.40 m (2H)	64.8	4.40 m (2H)	64.8
	4.38 dd (12.0, 2.8)		4.38 dd (11.8, 2.4)				4.38 dd (11.8, 2.5)					
	6Glc-*O*-Caf		6Glc-*O*-Caf		6Glc-*O*-Cou		6Glc-*O*-Caf		6Glc-*O*-Caf		6Glc-*O*-Cou	
1		127.3		127.2		126.6		127.3		127.3		126.7
2	6.71 d (1.4)	114.8	6.69, d (1.7)	114.7	7.03 d (8.7)	130.7	6.73 d (2.3)	114.8	6.71 d (1.5)	114.8	7.04 d (8.6)	130.8
3		146.5		146.4	6.62 d (8.5)	116.7		146.4		146.5	6.62 d (8.6)	116.7
4		149.3		149.3		161.0		149.2		149.3		161.0
5	6.59 d (8.1)	116.4	6.57 d (8.1)	116.3	6.62 d (8.5)	116.7	6.60 d (8.2)	116.3	6.59 d (8.1)	116.4	6.62 d (8.6)	116.7
6	6.53 dd (8.2, 1.6)	122.8	6.51 dd (8.2, 1.7)	122.7	7.03 d (8.7)	130.7	6.55 dd (8.2, 1.5)	122.8	6.52 dd (8.1, 1.5)	122.7	7.04 d (8.6)	130.8
7	7.23 d (15.9)	146.9	7.21 d (15.8)	146.8	7.28 d (15.9)	146.4	7.24 d (15.7)	146.8	7.22 d (15.9)	146.9	7.28 d (15.9)	146.4
8	5.90 d (15.9)	114.4	5.89 d (15.9)	114.5	5.94 d (15.9)	114.5	5.91 d (15.9)	114.5	5.90 d (15.9)	114.4	5.95 d (15.9)	114.5
9		169.0		168.9		168.9		169.0		168.9		168.9
	2GlcA-*O*-Caf’		2GlcA-*O*-Caf’		2GlcA-*O*-Caf		2GlcA-*O*-Cou		2GlcA-*O*-Fer		2GlcA-*O*-Cou’	
1		127.7		127.7		127.7		127.1		127.7		127.1
2	7.05 d (1.5)	115.3	7.05 d (1.6)	115.2	7.04 d (2.4)	115.2	7.44 d (8.5)	131.3	7.17 d (1.6)	111.8	7.45 d (8.7)	131.3
3		146.8		146.8		146.8	6.77 d (8.4)	116.8		149.3	6.77 d (8.7)	116.9
4		149.7		149.7		149.7		161.3		150.7		161.4
5	6.75 d (8.1)	116.5	6.75d (8.2)	116.5	6.75 d (8.2)	116.5	6.77 d (8.4)	116.8	6.78 d (8.2)	116.5	6.77 d (8.7)	116.9
6	6.95 dd (8.2, 1.6)	123.1	6.95 dd (8.2, 1.7)	123.1	6.94 dd (8.3, 1.9)	123.1	7.44 d (8.5)	131.3	7.07 dd (8.2, 1.4)	124.2	7.45 d (8.7)	131.3
7	7.65 d (15.9)	147.7	7.65 d (15.8)	147.7	7.64 d (15.9)	147.7	7.71 d (15.8)	147.4	7.71 d (16.0)	147.6	7.71 d (15.9)	147.4
8	6.34 d (15.9)	114.8	6.34 d (15.9)	114.8	6.33 d (15.9)	114.8	6.40, d (15.6)	114.8	6.44 d (15.9)	115.2	6.39 d (15.9)	114.8
9		168.3		168.2		168.2		168.2		168.2		168.2
OCH_3_									3.84 s	56.4		

^a^: overlapping signals.

For the GlcA residue, H-2 was downfield shifted to δ_H_ 5.16 (*dd, J* = 9.2, 8.1 Hz), and this proton correlated to the carbonyl carbon C-9_Cou’_ resonated at δ_C_ 168.2 in the HMBC spectrum. Therefore, **14** was quercetin 3-*O*-[(6-*O*-*E-p*-coumaroyl)-β-D-glucopyranosyl(1→2)]-β-D-galactopyranoside-7-*O*-(2-*O*-*E*-caffeoyl)-β-D-glucuropyranoside (named lensoside G) and **18** was identified as quercetin 3-*O*-[(6-*O*-*E-p*-coumaroyl)-β-D-glucopyranosyl(1→2)]-β-D-galactopyranoside-7-*O*-(2-*O*-*E-p-*-coumaroyl’)-β-D-glucuropyranoside, named lensoside H.

Compounds **11** and **16** are significantly different from the above-described flavonoids. The MS/MS analysis of compound **11** gave a deprotonated molecule at *m/z* 1047, which fragmented into ions at *m/z* 901 [(M−H)−146]^−^, *m/z* 755 [(M−H)−146−146]^−^, indicating the loss of one and two deoxyhexose/coumaric acid units, respectively, and *m/z* 284 [(M−H)−146−146−471]^−^, formed after a putative loss of pair of hexoses bound to a deoxyhexose, and corresponding to kaempferol radical ion. Low value of UV absorption maximum for Band I (315 nm) and high retention time indicate the acylation of **11** with coumaric acid. The ^13^C-NMR spectrum of **11** showed 48 signals, sorted by ^13^C and DEPT-135 experiments into two CH_3_, two CH_2_, 32 CH and 12 quaternary carbon atoms. The aromatic region of the ^1^H and COSY spectra of **11** exhibited the presence of two sets of aromatic protons, characteristic for kaempferol aglycone (Table 1). The carbohydrate region of ^1^H-NMR spectrum showed the presence of the oxymethine protons in the range δ 3.24–4.47 and two methyl groups at δ_H_ 1.28 (*d*, *J* = 6.2 Hz, H-6_Rha_) and 1.15 (*d*, *J* = 6.2 Hz, H-6_Rha’_). Moreover, four anomeric proton signals at δ_H_ 5.53 (*d*, *J* = 1.3 Hz, H-1_Rha_), 4.99 (*d*, *J* = 7.5 Hz, H-1_Gal_), 4.71 (*d*, *J* = 7.7 Hz, H-1_Glc_) and 4.48 (*d*, *J* = 1.4 Hz, H-1_Rha’_) were also observed, thus indicating the presence of four sugar units. Assignment of glucosidic protons system and sites of glycosylation was achieved by analysis of 1D TOCSY, 1D ROESY, COSY, HSQC and HMBC experiments (Table 5). Therefore, **11** was kaempferol 3-*O*-{[(6-*O*-*E-p*-coumaroyl)-β-D-glucopyranosyl(1→2)]-α-L-rhamnopyranosyl(1→6)}-β-D-galactopyranoside-7-*O*-α-L-rhamnopyranoside.

Compound **16** had a UV spectrum typical for quercetin 3-*O*-glycosides. The MS analysis of this flavonoid gave a deprotonated ion at *m/z* 447, as well as a dimeric ion [2M−H]^−^ at *m/z* 895. The deprotonated ion fragmented to *m/z* 300 [(M−H)-147]^−^, corresponding to the quercetin radical ion, created after the loss of a deoxyhexose. On the base of its NMR spectra the compound **16** was identified as a widely occurring flavonoid, quercitrin, the quercetin 3-*O*-α-L-rhamnoside [19].

Many of the purified lentil flavonoids, including almost all monoacylated compounds, were readily soluble in water, which can be attributed to their high glycosylation level, the presence of the glucuronide moiety and bisdesmosidic character.

The ability of the purified flavonoids to scavenge DPPH radicals was assessed using a rapid TLC- DPPH test. Their antiradical activities were compared with the activity of rutin, and expressed as a sample activity/rutin activity ratio (Table 6). Compound **5** turned out to be a better radical scavenger than rutin, and the antiradical activities of **6**, **7**, **8**, **12**, **14**, **15** and **16** were also high. It should be noted that the scavenging effect of quercetin derivatives was in most cases much stronger than that of kaempferol glycosides. Moreover, acylation of flavonoid glycosides with caffeic acid significantly increased their antiradical properties, which is particularly visible when activities of the compounds **3** and **6**, or **1** and **5** are compared.

**Table 5:** NMR spectroscopic data (methanol-*d*_4_, 500 MHz) for the sugar and phenolic acid units of the compound **11**.

11														
δ_H_ (*J* in Hz)		δ_C_	δ_H_ (*J* in Hz)		δ_C_	δ_H_ (*J* in Hz)		δ_C_	δ_H_ (*J* in Hz)		δ_C_	δ_H_ (*J* in Hz)		δ_C_
7-*O*-*α*-Rha			3-*O*-β-Gal			2Gal-*O*-β-Glc			6Gal-*O*-α-Rha			6Glc-*O*-Cou		
1	5.53 d (1.3)	99.9	1	4.99 d (7.5)	101.6	1'	4.71 d (7.7)	106.5	1	4.48 d (1.4)	101.8	1		126.7
2	4.04 dd (3.1, 1.6)	71.7	2	3.98 dd (9.5, 7.6)	83.0	2'	3.41 dd (9.0, 7.6)	76.2	2	3.48 dd (3.8, 1.5)	72.0	2	7.07 d (8.6)	130.7
3	3.84 dd (9.5, 3.4)	72.1	3	3.65 dd (9.3, 4.1)	74.7	3'	3.49 t (9.2)	77.8	3	3.41 dd (9.3, 3.9)	72.3	3	6.60 d (8.6)	116.6
4	3.49 t (9.2)	73.7	4	3.75 d (3.4)	70.0	4'	3.38 t (8.7)	72.1	4	3.24 t (9.5)	73.9	4		161.0
5	3.64 qd (9.6, 6.2)	71.2	5	3.53 t (6.4)	75.0	5'	3.68 ddd (9.2, 6.5, 2.2)	75.6	5	3.48 qd (9.6, 6.1)	69.7	5	6.60 d (8.6)	116.6
6	1.28 d (6.2)	18.2	6	3.67 ^a^3.35 ^a^	66.8	6'	4.47 dd (11.7, 2.5)4.40 dd (11.8, 6.7)	64.9	6	1.15 d (6.2)	18.0	6	7.07 d (8.6)	130.7
												7	7.32 d (15.9)	146.4
												8	6.00 d (15.9)	114.6
												9		168.9

^a^: overlapping signals.

**Table 6:** The DPPH scavenging activity of isolated flavonoids, expressed in relation to the activity of rutin.

Compound	Activity in Relation to Rutin	SD
**1**	0.61	0.13
**3**	0.09	0.04
**5**	1.15	0.11
**6**	0.82	0.08
**7**	0.75	0.11
**8**	0.73	0.12
**9**	0.26	0.08
**10**	0.07	0.06
**11**	0.11	0.05
**12**	0.72	0.14
**14**	0.73	0.11
**15**	0.82	0.14
**16**	0.80	0.09
**17**	0.69	0.12
**18**	0.46	0.09
Rutin	1.00	

On the contrary, acylation with *p*-coumaric or ferulic acid had a small effect on the antiradical activity of the investigated flavonoids, which is especially visible in the case of the kaempferol derivatives (compounds **9**, **10**, **11**, **18**). These observations can be explained by the fact that quercetin and caffeic acid were reported to be more efficient DPPH scavengers (which can be attributed to the presence of catechol moiety in their molecules) than kaempferol, ferulic acid, and particularly *p*-coumaric acid, [20,21,22,23].

It seems that the majority of the isolated flavonoids, except for compounds **2** and **16** [18,19], have not been described before in the scientific literature. However, it is possible that the compound **11** was previously detected in lentil seeds by Zou *et al.*, who found two flavonoid derivatives with similar molecular masses (like **11**, they gave deprotonated ions at *m/z* 1047) and UV spectrum [6]. Compound **11** is an acylated form of kaempferol 3-*O*-[β-D-glucopyranosyl(1→2){α-L- rhamnopyranosyl(1→6)}-β-D-galactopyranoside]-7-*O*-α-L-rhamnopyranoside, a flavonoid occurring in lentil seeds, and found also in the legume *Ateleia chicoasensis* and the cactus *Cephalocereus senilis* [24,25,26]. Moreover, a non-acylated flavonoid (or more flavonoids with nearly identical retention times), giving the deprotonated ion at *m/z* 901 and showing the relevant fragmentation pattern, was found to be the main phenolic compound of the Tina lentil seeds (see Supplementary Figure S227)). 7-*O*-glucuronides of flavonols were rarely found. *Cichorium intybus* and some *Epilobium* sp. contain simple kaempferol and quercetin 7-*O*-glucuronides, while the more complex glycosides were found in tulip (*Tulipa gesneriana*) and onion (*A. cepa*) [18,27,28,29]. In contrast, 7-*O*-glucuronides of flavones are more widespread among plants, they can be found, e.g. in different plants belonging to the Lamiales order [30]. Among legumes, such compounds can be found in aerial parts of *Medicago* sp., known to contain different acylated and non-acylated 7-*O*-glucuronides of apigenin, luteolin, chrysoeriol, and tricin [31,32]. It seems the presence of the described 7-*O*-glucuronides of kaempferol and quercetin in aerial parts of lentil is interesting from chemotaxonoimic point of view, and these compounds can be used as molecular markers.

There are several articles describing phenolic compounds of lentil sprouts. Phenolic constituents of the lentil variety Aldona were analyzed, using LC-MS, by Troszyńska *et al*. [13]. They detected eight acylated and non-acylated glycosides of kaempferol and quercetin (only generally described), including an acylated kaempferol derivative showing a deprotonated ion at *m/z* 931. It seems this flavonoid may be identical to the compound **9** due to their similar fragment ions and UV spectra. Other publications present levels of some phenolic acids and flavonoid aglycones (luteolin, kaempferol, quercetin, daidzein, genistein, naringenin, catechin) but no information about flavonoid glycosides is available [14,15].

## 3. Experimental Section

### 3.1. Chemicals and Plant Material

Methanol (ACS), acetonitrile (HPLC gradient and isocratic grade) and ethyl acetate (ACS) were obtained from J.T. Baker (Deventer, The Netherlands). Formic acid puriss. p.a. and MS grade were from Sigma-Aldrich (St. Louis, MO, USA) and Fluka (St. Louis, MO, USA), respectively. d-Glucose, d-galactose, l-rhamnose, d-glucuronic acid, l-cysteine methyl ester hydrochloride, *o*-tolyl isothiocyanate, kaempferol, quercitin and ferulic acid were obtained from Sigma-Aldrich. Caffeic acid, *p*-coumaric acid, and NaOH were from Merck (Darmstadt, Germany). d-Cysteine methyl ester hydrochloride was from TCI (Tokyo Chemical Industry Co. Ltd., Tokyo, Japan). The other chemicals were obtained from POCH S.A. (Gliwice, Poland). Seeds of lentil (*Lens culinaris* Medik.) cultivar Tina were obtained from the Department of Agrotechnology and Crop Management, University of Warmia and Mazury, Olsztyn, Poland. Lentil was grown in the experimental field of the Institute of Soil Science and Plant Cultivation in Puławy, Poland, and harvested during the flowering period. The collected aerial parts of lentil were freeze-dried (Gamma 2-16 LSC, Christ, Osterode am Harz, Germany), milled, and defatted in a Soxhlet extractor with chloroform.

### 3.2. Preliminary Analyses

Crude extracts from aerial parts of lentil, and chromatographic fractions were analyzed by UHPLC-MS/MS. Chromatographic separations were performed on an ACQUITY UPLC System chromatograph (Waters, Milford, MA, USA), equipped with a PDA detector and a triple quadrupol mass detector (ACQUITY TQD, Waters). Samples were separated on a ACQUITY BEH C18 (100 × 2.1 mm, 1.7 µm; Waters) column, maintained at 40 °C. The elution (400 µL·min^−1^) was carried out with a gradient of solvent B (acetonitrile with 0.1% FA) in solvent A (water with 0.1% FA): 0–1 min, 5% B; 1–24 min 5%–50% B; 24–25 min 50%–95% B; 25–27 min 95% B; 27–27.1 min, 95%–5% B; 27.1–30 min, 5% B. Mass analyses were performed in the negative ionization mode, capillary voltage was 2.8 kV; cone voltage 40 V; source temperature 140 °C, desolvation temperature 350 °C, cone gas flow (nitrogen) 100 L·h^−1^, desolvation gas flow 800 L h^−1^, the collision gas (argon) flow was 0.1 mL·min^−1^.

### 3.3. Extraction and Fractionation of Crude Lentil Extract

A portion of the defatted plant material (250 g) was subjected to triple extraction with boiling 80% methanol aqueous solution (v/v; 2.5 L; 1 h) under reflux. The collected extract was filtered through a filter funnel, concentrated using a rotary evaporator (Heidolph, Schwabach, Germany), and freeze-dried. The resulting crude extract (45 g) was fractionated using vacuum liquid chromatography. A forty gram portion of the crude extract was dissolved in 1% aqueous methanol and loaded onto a C18 column (10 × 5.5 cm, i.d.; Lichroprep RP-18 40–63 μm, Merck), equilibrated with 1% methanol. The column was washed with the same solution to remove sugars and other highly polar compounds, the bound substances were subsequently eluted with methanol aqueous solutions of increasing concentration: 20%, 40%, 60%, 80%, 100% methanol (v/v). The obtained eluates were concentrated by rotary evaporation and freeze-dried. Masses of the dried fractions were as follows: 20% methanol (F1—3.08 g); 40% methanol (F2—4.34 g); 60% methanol (F3—2.43 g); 80% (F4—0.41 g); 100% methanol (F5—0.96 g). Fractions F1–F3 were used in the next step of the purification procedure, since they were shown by UHPLC-ESI-MS/MS analyses to contain numerous phenolic compounds, mainly flavonoids.

### 3.4. Purification of Phenolic Compounds

Fractions F1–F3 were subjected to low pressure reversed phase liquid chromatography on a C18 column (30 × 3.4 cm, i.d.; Lichroprep RP-18 40-63 μm, Merck). For separation of fraction F1, the column was equilibrated with 1.5% aqueous methanol with 0.1% formic acid. A 1.400 g portion of fraction F1 was dissolved in the same solution and loaded onto the column. The column was washed with the starting eluent, and the constituent phenolics were eluted with a stepwise gradient of: 5%–40% aqueous methanol containing 0.1% formic acid; 10 mL fractions were collected.

A similar experimental scheme was also used for fractions F2 and F3. Fraction F2 (1.400 g) was dissolved in 5% aqueous methanol with 0.1% formic acid and applied on the chromatographic column equilibrated with the same solvent. The column was subsequently washed with the starting eluent, followed by the step gradient of 10%–45% methanol containing 0.1% formic acid. Fraction F3 (1.443 g) was dissolved in 33% aqueous methanol with 0.1% formic acid and loaded onto the chromatographic column equilibrated with 25% methanol containing 0.1% formic acid. The column was washed with the starting solution and the sample constituents were eluted with a stepwise gradient of 30%–55% methanol.

The chromatographic separations were monitored by TLC on cellulose plates (15:85 acetic acid/water; preparation F1) or silica plates (60:20:5:5 MeCN/H_2_O/CHCl_3_/FA; preparations F2 and F3), spots were visualized under UV at 365 nm or were visible in daylight. Chromatographic fractions sharing the same phenolic compounds were combined, evaporated, dissolved in water and freeze-dried.

Lentil flavonoids were further purified by reversed-phase semi-preparative HPLC (Analitical to Semi-preparative HPLC system, Gilson Inc., Middleton, WI, USA) on a C18 column (Kromasil 100-5-C18, 250 × 10 mm, 5 μm). Preparations were separated isocratically, using aqueous acetonitrile solutions of different concentrations (from 6% to 19% MeCN), containing 0.2% FA. The mobile phase flow was 6.5 mL·min^−1^, the column temperature was maintained at 30 °C.

### 3.5. Mass Analyses of Purified Compounds

Exact masses of lentil flavonoids were determined by direct infusion electrospray high resolution (Q-TOF) mass spectrometry (HRESI-MS), using a SYNAPT G2-S HDMS mass spectrometer (Waters). Fragmentation analyses were performed by direct infusion electrospray mass spectrometry, using a ACQUITY TQD mass spectrometer (Waters).

### 3.6. NMR Analysis

NMR spectra were acquired in MeOH-*d*_4_ at 25 °C on an Avance III HD 500 MHz instrument (^1^H: 500.18 MHz; ^13^C 125.79 MHz; Bruker BioSpin, Rheinstetten, Germany). Standard pulse sequences and parameters were used to obtain 1D ^1^H, 1D selective TOCSY, 1D selective ROESY, 1D ^13^C and DEPT-135, gCOSY, TOCSY (mixing time 120 ms), ROESY (mixing time 250 ms), gHSQC, gHSQC-TOCSY (mixing time 80 ms), gHMBC spectra. Chemical shift referencing was carried out using the internal solvent resonances at δ_H_ 3.31 and δ_C_ 49.0 (calibrated to TMS at 0.00 ppm).

### 3.7. Alkaline and Acid Hydrolysis of Flavonoids

Flavonoids (0.25–0.30 mg) were subjected to alkaline hydrolysis (1 mL of 0.2 M NaOH + 0.05% ascorbic acid, 2 h), carried out at room temperature in the dark. Samples were subsequently acidified with 2 M HCl to achieve a pH value of about 2, and liberated phenolic acids were extracted with ethyl acetate (3 × 1 mL). The organic extracts were dried with a stream of nitrogen, dissolved in 25% methanol and analyzed to identify phenolic acids. UPLC-ESI-MS/MS analyses were performed using a Waters ACQUITY UPLC^®^ HSS C18 column (100 × 1 mm, 1.8 µm), maintained at 30 °C. The following gradient of solvent A (water with 0.1% FA, v/v) and solvent B (in acetonitrile with 0.1% FA, v/v) was used to elute analytes: 0–0.07 min, 5% B; 0.07–8.33 min, 5%–15% B; 8.33–8.67 min, 15%–60% B; 8.67–9.33 min 60% B; 9.33–9.40 min, 60%–5% B; 9.40–12.00 min, 5% B; the flow rate was 0.15 mL·min^−1^ ESI-MS/MS analyses were performed using negative ionization mode and the Selected Reaction Monitoring (SRM) detection method.

The water phases were dried and then desalted by SPE (Oasis HLB 30 mg). Methanol SPE eluates, containing deacylated flavonoids, were dried and subjected to acid hydrolysis (1 mL of 2M HCl, 2 h, 100 °C). Aglycones were then extracted with ethyl acetate (3 × 1 mL), dried with a stream of nitrogen, dissolved in 50% methanol, and identified by UPLC-ESI-MS. Chromatographic separations were performed on a ACQUITY BEH C18 (100 × 2.1 mm, 1.7 μm; Waters) column (40 °C). The mobile phases were water with 0.1% FA (A) and in acetonitrile with 0.1% FA (B). Samples were separated (400 μL·min^−1^) with the following gradient: 0–1 min, 15% B; 1–11 min, 15%–95% B; 11–13 min, 95% B; 13–13.1 min, 95%–15% B; 13.1–15 min, 15% B. Mass spectra were obtained in negative ionization mode, MS parameters were as follows: capillary voltage 2.8 kV; cone voltage 45 V; source temperature 140 °C, desolvation temperature 350 °C, cone gas flow (nitrogen) 100 L·h^−1^ desolvation gas flow 800 L·h^−1^. Sugar-containing aqueous layers were neutralized with Amberlite IRA-400 (OH^−^ form) [33]. After drying, the samples were used to determine the absolute configuration of the constituent monosaccharides.

### 3.8. Determining the Absolute Configuration of Sugars

The absolute configuration of sugars was determined according to the modified method of Tanaka, *et al*. [33] Samples of monosaccharides obtained after the acid hydrolysis of flavonoids were dissolved in anhydrous pyridine (100 μL) containing l-cysteine methyl ester hydrochloride (0.5 mg) and heated at 60 °C for 1 h. Then of solution of *o*-tolyl isothiocyanate (0.5 mg) in pyridine (100 μL) was added, and the mixture was heated for another hour, at 60 °C. After cooling, samples were analyzed by UPLC-ESI-MS/MS. Chromatographic separations were carried out on a Acquity BEH C18 column (100 × 2.1 mm, 1.7 μm; Waters). Mass spectrometry analyses were performed in positive ionization mode, using the SRM method. Details of the analysis can be found in the work of Pérez, *et al*. [34]. d-glucose (Glc), d-galactose (Gal), d-glucuronic acid (GlcA) and l-rhamnose (Rha) were identified on the base of retention time and *m/z* values of authentic standards, derivatized in the same way.

### 3.9. TLC–DPPH Test

The ability of lentil flavonoids to scavenge DPPH radicals was determined using a TLC rapid test [35]. Briefly, standard solutions (1 mg·mL^−1^) of the purified flavonoids and rutin (positive control) were prepared. Aliquots of the standard solutions (3 µL) were applied onto a silica TLC plates, and the plates were developed as described above. They were subsequently dried and immersed for 3 s in freshly prepared 0.2% (w/v) methanolic DPPH solution. The test was performed in triplicate. The developed plates were kept in the dark for 30 min and then scanned in a flat-bed scanner. The obtained scans were analyzed using ImageJ image processing program. The antiradical activity of lentil flavonoids was expressed in relation to activity of rutin.

### 3.10. Chemical Data of Lentil Flavonoids

*Quercetin 3-O-β-D-glucopyranosyl(1→2)-β-D-galactopyranoside-7-O-β-D-glucuropyranoside* (**1**); (11 mg), yellow solid; UV (UPLC-PDA) λ_max_ (nm): 255, 352; HRESI-MS (Q-TOF), *m/z*: 801.1713 [M−H]^−^ (calc. for C_33_H_37_O_23_^−^ = 801.1726); ESI-MS/MS (TQ), *m/z*: 801 [M−H]^−^, 625 [M−H−176]^−^, 300 [Y_0_−H]^−^· = [M−H−176−325 = quercetin-2H]^−^.

*Kaempferol 3-O-β-D-glucopyranosyl(1→2)-β-D-galactopyranoside-7-O-β-D-glucuropyranoside* (**3**): (6 mg), yellow solid, UV (UPLC-PDA) λ_max_ (nm): 265, 346; HRESI-MS (Q-TOF), *m/z*: 785.1769 [M−H]^−^ (calc. for C_33_H_37_O_22_^−^ = 785.1776); ESI-MS/MS (TQ), *m/z*: 785 [M−H]^−^, 609 [M−H−176]^−^, 285 [Y_0_]^−^ = [M−H−176−324 = kaempferol−H]^−^.

*Quercetin 3-O-[(6-O-E-3,4,5-trihydroxycinnamoyl)-β-D-glucopyranosyl(1→2)]-β-D-galacto- pyranoside-7-O-β-D-glucuropyranoside* (**4**): (1 mg), yellow solid, UV (UPLC-PDA) λ_max_ (nm): 255, 335; HRESI-MS (Q-TOF), *m/z*: 1003.1959 [M+Na]^+^ (calc. for C_42_H_44_O_27_Na^+^ = 1003.1968); ESI-MS/MS (TQ), *m/z*: 979 [M−H]^−^, 803 [M−H−176]^−^, 625 [M−H−176−178(triOHCin)]^−^, 300 [Y_0_−H]^−^· = [M−H−176−178−325 = quercetin−2H]^−^.

*Quercetin 3-O-[(6-O-E-caffeoyl)-β-D-glucopyranosyl(1→2)]-β-D-galactopyranoside-7-O-β-D-glucuro-pyranoside* (**5**) (30.1 mg), yellow solid, UV (UPLC-PDA) λ_max_ (nm): 253, 335; HRESI-MS (Q-TOF), *m/z*: 965.2171 [M+H]^+^ (calc. for C_42_H_45_O_26_^+^ = 965.2199); ESI-MS/MS (TQ), *m/z*: 963 [M−H]^−^, 787 [M−H−176]^−^, 625 [M−H−176−162]^−^, 300 [Y_0_−H]^−^· = [M−H−176−162−325 = quercetin−2H]^−^.

*Kaempferol 3-O-[(6-O-E-caffeoyl)-β-D-glucopyranosyl(1→2)]-β-D-galactopyranoside-7-O-β-D-glucuropyranoside* (**6**): (14.6 mg), yellow solid, UV (UPLC-PDA) λ_max_ (nm): 243, 266, 328; HRESI-MS (Q-TOF), *m/z*: 971.2070 [M+Na]^+^ (calc. for C_42_H_44_O_25_Na^+^ = 971.2069); ESI-MS/MS (TQ), *m/z*: 947 [M−H]^−^, 771 [M−H−176]^−^, 609 [M−H−176−162]^−^, 284 [Y_0_−H]^−^· = [M−H−176−162−325 = kaempferol−2H]^−^.

*Quercetin 3-O-[(6-O-E-p-coumaroyl)-β-D-glucopyranosyl-(1→2)]-β-D-galactopyranoside-7-O-β-D-glucuropyranoside* (**7**): (28.5 mg), yellow solid, UV (UPLC-PDA) λ_max_ (nm): 261sh, 268, 317; HRESI-MS (Q-TOF), *m/z*: 947.2078 [M−H]^−^ (calc. for C_42_H_43_O_25_^−^ = 947.2093); ESI-MS/MS (TQ), *m/z*: 947 [M−H]^−^, 771 [M−H−176]^−^, 625 [M−H−176−146]^−^, 300 [Y_0_−H]^−^· = [M−H−176−146−325= quercetin−2H]^−^.

*Quercetin 3-O-[(6-O-E-feruloyl)-β-D-glucopyranosyl(1→2)]-β-D-galactopyranoside-7-O-β-D-glucuro-pyranoside* (**8**): (18.2 mg), yellow solid, UV (UPLC-PDA) λ_max_ (nm): 253, 266sh, 332; HRESI-MS (Q-TOF), *m/z*: 977.200 [M−H]^−^ (calc. for C_43_H_45_O_26_^−^ = 977.2199); ESI-MS/MS (TQ), *m/z*: 977 [M−H]^−^, 801 [M−H−176]^−^, 625 [M−H−176−176]^−^, 300 [Y_0_−H]^−^· = [M−H−176−176−325 = quercetin−2H]^−^.

*Kaempferol 3-O-[(6-O-E-p-coumaroyl)-β-d-glucopyranosyl(1→2)]-β-d-galactopyranoside-7-O-β-D-glucuropyranoside* (**9**): (23.2 mg), yellow solid, UV (UPLC-PDA) λ_max_ (nm): 268, 315; HRESI-MS (Q-TOF), *m/z*: 931.2140 [M−H]^−^ (calc. for C_42_H_43_O_24_^−^ = 931.2144); ESI-MS/MS (TQ), *m/z*: 931 [M−H]^−^, 755 [M−H-176]^−^, 609 [M−H−176−146]^−^, 284 [Y_0_−H]^−^· = [M−H−176−146−325= kaempferol−2H]^−^.

*Kaempferol 3-O-[(6-O-E-feruloyl)-β-D-glucopyranosyl(1→2)]-β-D-galactopyranoside-7-O-β-D-glucuropyranoside* (**10**): (17.1 mg), yellow solid, UV (UPLC-PDA) λ_max_ (nm): 268, 327; HRESI-MS (Q-TOF), *m/z*: 961.2240 [M−H]^−^ (calc. for C_43_H_45_O_25_^−^ = 961.2250); ESI-MS/MS (TQ), *m/z*: 961 [M−H]^−^, 785 [M−H−176]^−^, 609 [M−H−176−176]^−^, 284 [Y_0_−H]^−^· = [M−H−176−176−325= kaempferol−2H]^−^.

*Kaempferol 3-O-{[(6-O-E-p-coumaroyl)-β-D-glucopyranosyl(1→2)]-α-L-rhamnopyranosyl(1→6)}-β-d-galactopyranoside-7-O-α-L-rhamnopyranoside* (**11**): (18.7 mg) yellow solid, UV (UPLC-PDA) λ_max_ (nm): 269, 315; HRESI-MS (Q-TOF), *m/z*: 1049.3115 [M+H]^+^ (calc. for C_48_H_57_O_26_^+^ = 1049.3138); ESI-MS/MS (TQ), *m/z*: 1047 [M−H]^−^, 901 [M−H−146]^−^, 755 [M−H−146−146]^−^, 284 [Y_0_−H]^−^· = [M−H−146−146−471= kaempferol−2H]^−^.

*Quercetin 3-O-[(6-O-E-caffeoyl)-β-D-glucopyranosyl(1→2)]-β-D-galactopyranoside-7-O-(2-O-E-caffeoyl')-β-D-glucuropyranoside* (**12**): (14 mg) yellow solid, UV (UPLC-PDA) λ_max_ (nm): 249, 266sh, 305sh, 332; HRESI-MS (Q-TOF), *m/z*: 1125.2423 [M−H]^−^ (calc. for C_51_H_49_O_29_ = 1125.2360); ESI-MS/MS (TQ), *m/z*: 1125 [M−H]^−^, 963 [M−H−162]^−^, 787 [M−H−162−176]^−^, 625 [M−H−162−176−162]^−^, 300 [Y_0_−H]^−^· = [M−H−162−176−162−325= quercetin−2H]^−^.

*Kaempferol 3-O-[(6-O-E-caffeoyl)-β-D-glucopyranosyl(1→2)]-β-D-galactopyranoside-7-O-(2-O-E-caffeoyl')-β-D-glucuropyranoside* (**13**): (9.2 mg) yellow solid, UV (UPLC-PDA) λ_max_ (nm): 243, 268, 328; HRESI-MS (Q-TOF), *m/z*: 1109.2390 [M−H]^−^ (calc. for C_51_H_49_O_28_^−^ = 1109.2410); ESI-MS/MS (TQ), *m/z*: 1109 [M−H]^−^, 947 [M−H−162]^−^, 771 [M−H−162−176]^−^, 609 [M−H−162−176−162]^−^, 284 [Y_0_−H]^−^· = [M−H−162−176−162−325= kaempferol−2H]^−^.

*Quercetin 3-O-[(6-O-E-p-coumaroyl)-β-D-glucopyranosyl(1→2)]-β-D-galactopyranoside-7-O-(2-O-E-caffeoyl)-β-D-glucuropyranoside* (**14**): (4.6 mg) yellow solid, UV (UPLC-PDA) λ_max_ (nm): 254, 269, 321; HRESI-MS (Q-TOF), *m/z*: 1111.2562 [M+H]^+^ (calc. for C_51_H_51_O_28_^+^ = 1111.2567); ESI-MS/MS (TQ), *m/z*: 1109 [M−H]^−^, 947 [M−H−162]^−^, 771 [M−H−162−176]^−^, 625 [M−H−162−176−146]^−^, 300 [Y_0_−H]^−^· = [M−H−162−176−146−325= quercetin−2H]^−^.

*Quercetin 3-O-[(6-O-E-caffeoyl)-β-D-glucopyranosyl(1→2)]-β-D-galactopyranoside-7-O-(2-O-E-p-coumaroyl)-β-D-glucuropyranoside* (**15**): (18 mg) yellow solid, UV (UPLC-PDA) λ_max_ (nm): 254, 269, 320; HRESI-MS (Q-TOF), *m/z*: 1111.2557 [M+H]^+^ (calc. for C_51_H_51_O_28_^+^ = 1111.2567); ESI-MS/MS (TQ), *m/z*: 1109 [M−H]^−^, 963 [M−H−146]^−^, 787 [M−H−146−176]^−^, 625 [M−H−146−176−162]^−^, 300 [Y_0_−H]^−^· = [M−H−146−176−162−325 = quercetin−2H]^−^.

*Quercetin 3-O-[(6-O-E-caffeoyl)-β-D-glucopyranosyl(1→2)]-β-D-galactopyranoside-7-O-(2-O-E-feruloyl)-β-D-glucuropyranoside* (**17**): (6.4 mg) yellow solid, UV (UPLC-PDA) λ_max_ (nm): 250, 266sh, 331; HRESI-MS (Q-TOF), *m/z*: 1141.2651 [M+H]^+^ (calc. for C_51_H_53_O_29_^+^ = 1141.2673); ESI-MS/MS (TQ), *m/z*: 1139 [M−H]^−^, 963 [M−H−176]^−^, 787 [M−H−176−176]^−^, 625 [M−H−176−176−162]^−^, 300 [Y_0_−H]^−^· = [M−H−176−176−162−325 = quercetin−2H]^−^.

*Quercetin 3-O-[(6-O-E-p-coumaroyl)-β-D-glucopyranosyl(1→2)]-β-D-galactopyranoside-7-O-(2-O-E-p-coumaroyl')-β-D-glucuropyranoside* (**18**): (5.2 mg) yellow solid, UV (UPLC-PDA) λ_max_ (nm): 268, 316; HRESI-MS (Q-TOF), *m/z*: 1095.2607 [M+H]^+^ (calc. for C_51_H_51_O_27_^+^ = 1095.2618); ESI-MS/MS (TQ), *m/z*: 1093 [M−H]^−^, 947 [M−H−146]^−^, 771 [M−H−146−176]^−^, 625 [M−H−146−176−146]^−^, 300 [Y_0_−H]^−^· = [M−H−146−176−146−325 = quercetin−2H]^−^.

## 4. Conclusions

As a concluding remark, it may be pointed out that aerial parts of lentil are a source of numerous flavonoids. In this study we presented the purification and structure elucidation of 18 acylated and non-acylated glycosides of kaempferol and quercetin, including 16 compounds which have not been reported previously in the scientific literature: quercetin 3-*O*-β-D-glucopyranosyl(1→2)-β-D-galactopyranoside-7-*O*-β-D-glucuropyranoside (**1**), its derivatives **4**, **5**, **7**, **8**, **12**, **14**, **15**, **17**, **18** acylated with 3,4,5-trihydroxycinnamic acid, kaempferol 3-*O*-β-D-glucopyranosyl(1→2)-β-D-galacto-pyranoside-7-*O*-β-D-glucuropyranoside (**3**), and its derivatives **6**, **9**, **10**, **13** acylated with caffeic, *p*-coumaric, or ferulic acid, as well as kaempferol 3-*O*-{[(6-*O*-*E*-*p*-coumaroyl)-β-D-glucopyranosyl(1→2)]-α-L-rhamno-pyranosyl(1→6)}-β-D-galactopyranoside-7-*O*-α-L-rhamnopyranoside (**11**). Kaempferol and quercetin are known as potent antioxidants with diverse biological activity and health-promoting properties. Aerial parts of lentil may find use as a source of water-soluble flavonol glycosides, which can be applied, e.g., as nutraceuticals.

## Supplementary Materials

NMR spectra of the new compounds, chromatograms showing the results of the analyses of phenolic acids and sugars, and a chromatogram of lentil seed extract are provided as Supplementary Information. Supplementary materials can be accessed athttp://www.mdpi.com/1420-3049/19/11/18152/s1.

## Supplementary Files

Supplementary File 1
