# Supplementary material for: Novel Flavonol Glycosides from the Aerial Parts of Lentil (Lens culinaris)
Source: Molecules. 2014 Nov 6;19(11):18152–78. doi: 10.3390/molecules191118152 (PMC6270693; doi:10.3390/molecules191118152)

Supplementary Materials

Compound 1

Figure S1. 1D ^{1}H-NMR spectrum of compound **1** (methanol-*d*4, 500.18 MHz).
Figure S2. 1D ^{13}C-NMR spectra (^{1}H decoupled and DEPT-135) (methanol-*d*4, 125.77 MHz).
Figure S3. 2D *g*-COSY NMR spectrum of compound **1** (methanol-*d*4, 500.18 MHz).
Figure S4. 2D TOCSY NMR spectrum of compound **1** (methanol-*d*4, 500.18 MHz).
Figure S5. 2D ROESY NMR spectrum of compound **1** (methanol-*d*4, 500.18 MHz).
Figure S6. 2D *g*-HSQC-NMR spectrum of compound **1** (methanol-*d*4, 500.18 MHz, 125.77 MHz).
Figure S7. 2D *g*-HSQC-TOCSY NMR spectrum of compound **1** (methanol-*d*4, 500.18 MHz, 125.77 MHz).
Figure S8. 2D *g*-HMBC-NMR spectrum of compound **1** (methanol-*d*4, 500.18 MHz, 125.77 MHz).
Figure S9. 1D TOCSY and 1D ROESY NMR subspectra of $H\text{-}1_{(7\text{-}O\text{-}\beta\text{-}GlcA)}$ in compound **1** (methanol-*d*4, 500.18 MHz).
Figure S10. 1D TOCSY and 1D ROESY NMR subspectra of $H\text{-}1_{(3\text{-}O\text{-}\beta\text{-}Gal)}$ in compound **1** (methanol-*d*4, 500.18 MHz).
Figure S11. 1D TOCSY and 1D ROESY NMR subspectra of $H\text{-}1_{(2^{Gal}\text{-}O\text{-}\beta\text{-}Glc)}$ in compound **1** (methanol-*d*4, 500.18 MHz).

Compound 2

Figure S12. 1D ^{1}H-NMR spectrum of compound **2** (methanol-*d*4, 500.18 MHz).
Figure S13. 1D ^{13}C-NMR spectrum (^{1}H decoupled) of compound **2** (methanol-*d*4, 125.77 MHz).
Figure S14. 2D *g*-COSY NMR spectrum of compound **2** (methanol-*d*4, 500.18 MHz).
Figure S15. 2D TOCSY NMR spectrum of compound **2** (methanol-*d*4, 500.18 MHz).
Figure S16. 2D ROESY NMR spectrum of compound **2** (methanol-*d*4, 500.18 MHz).
Figure S17. 2D *g*-HSQC-NMR spectrum of compound **2** (methanol-*d*4, 500.18 MHz, 125.77 MHz).
Figure S18. 2D *g*-HSQC-TOCSY NMR spectrum of compound **2** (methanol-*d*4, 500.18 MHz, 125.77 MHz).
Figure S19. 2D *g*-HMBC-NMR spectrum of compound **2** (methanol-*d*4, 500.18 MHz, 125.77 MHz).
Figure S20. 1D TOCSY and 1D ROESY NMR subspectra of $H\text{-}1_{(7\text{-}O\text{-}\beta\text{-}GlcA)}$ in compound **2** (methanol-*d*4, 500.18 MHz).
Figure S21. 1D TOCSY and 1D ROESY NMR subspectra of $H\text{-}1_{(3\text{-}O\text{-}\beta\text{-}Glc)}$ in compound **2** (methanol-*d*4, 500.18 MHz).
Figure S22. 1D TOCSY and 1D ROESY NMR subspectra of $H\text{-}1_{(2^{Glc}\text{-}O\text{-}\beta\text{-}Glc)}$ in compound **2** (methanol-*d*4, 500.18 MHz).

Compound 3

Figure S23. 1D ^{1}H-NMR spectrum of compound **3** (methanol-*d*4, 500.18 MHz).
Figure S24. 1D ^{13}C-NMR spectra (1H decoupled and DEPT-135) of compound **3** (methanol-*d*4, 125.77 MHz).
Figure S25. 2D *g*-COSY NMR spectrum of compound **3** (methanol-*d*4, 500.18 MHz).
Figure S26. 2D TOCSY NMR spectrum of compound **3** (methanol-*d*4, 500.18 MHz).

Figure S27. 2D ROESY NMR spectrum of compound **3** (methanol-d_4, 500.18 MHz).
Figure S28. 2D *g*-HSQC-NMR spectrum of compound **3** (methanol-d_4, 500.18 MHz, 125.77 MHz).
Figure S29. 2D *g*-HSQC-TOCSY NMR spectrum of compound **3** (methanol-d_4, 500.18 MHz, 125.77 MHz).
Figure S30. 2D *g*-HMBC-NMR spectrum of compound **3** (methanol-d_4, 500.18 MHz, 125.77 MHz).
Figure S31. 1D TOCSY and 1D ROESY NMR subspectra of H-1$_{(7\text{-}O\text{-}\beta\text{-GlcA})}$ in compound **3** (methanol-d_4, 500.18 MHz).
Figure S32. 1D TOCSY and 1D ROESY NMR subspectra of H-1$_{(3\text{-}O\text{-}\beta\text{-Gal})}$ in compound **3** (methanol-d_4, 500.18 MHz).
Figure S33. 1D TOCSY and 1D ROESY NMR subspectra of H-1$_{(2^{Gal}\text{-}O\text{-}\beta\text{-Glc})}$ in compound **3** (methanol-d_4, 500.18 MHz).

Compound 4

Figure S34. 1D ^{1}H-NMR spectrum of compound **4** (methanol-d_4, 500.18 MHz).
Figure S35. 1D ^{13}C-NMR spectra (1H decoupled and DEPT-135) of compound **4** (methanol-d_4, 125.77 MHz).
Figure S36. 2D *g*-COSY NMR spectrum of compound **4** (methanol-d_4, 500.18 MHz).
Figure S37. 2D TOCSY NMR spectrum of compound **4** (methanol-d_4, 500.18 MHz).
Figure S38. 2D ROESY NMR spectrum of compound **4** (methanol-d_4, 500.18 MHz).
Figure S39. 2D *g*-HSQC-NMR spectrum of compound **4** (methanol-d_4, 500.18 MHz, 125.77 MHz).
Figure S40. 2D *g*-HSQC-TOCSY NMR spectrum of compound **4** (methanol-d_4, 500.18 MHz, 125.77 MHz).
Figure S41. 2D *g*-HMBC-NMR spectrum of compound **4** (methanol-d_4, 500.18 MHz, 125.77 MHz).
Figure S42. 1D TOCSY and 1D ROESY NMR subspectra of H-1$_{(7\text{-}O\text{-}\beta\text{-GlcA})}$ and H-1$_{(3\text{-}O\text{-}\beta\text{-Gal})}$ in compound **4** (methanol-d_4, 500.18 MHz).
Figure S43. 1D TOCSY and 1D ROESY NMR subspectra of H-1$_{(2^{Gal}\text{-}O\text{-}\beta\text{-Glc})}$ in compound **4** (methanol-d_4, 500.18 MHz).

Compound 5

Figure S44. 1D ^{1}H-NMR spectrum of compound **5** (methanol-d_4, 500.18 MHz).
Figure S45. 1D ^{13}C-NMR spectra (1H decoupled and DEPT-135) of compound **5** (methanol-d_4, 125.77 MHz).
Figure S46. 2D *g*-COSY NMR spectrum of compound **5** (methanol-d_4, 500.18 MHz).
Figure S47. 2D TOCSY NMR spectrum of compound **5** (methanol-d_4, 500.18 MHz).
Figure S48. 2D ROESY NMR spectrum of compound **5** (methanol-d_4, 500.18 MHz).
Figure S49. 2D *g*-HSQC-NMR spectrum of compound **5** (methanol-d_4, 500.18 MHz, 125.77 MHz).
Figure S50. 2D *g*-HSQC-TOCSY NMR spectrum of compound **5** (methanol-d_4, 500.18 MHz, 125.77 MHz).
Figure S51. 2D *g*-HMBC-NMR spectrum of compound **5** (methanol-d_4, 500.18 MHz, 125.77 MHz).
Figure S52. 1D TOCSY and 1D ROESY NMR subspectra of H-1$_{(7\text{-}O\text{-}\beta\text{-GlcA})}$ and H-1$_{(3\text{-}O\text{-}\beta\text{-Gal})}$ in compound **5** (methanol-d_4, 500.18 MHz).

Figure S53. 1D TOCSY and 1D ROESY NMR subspectra of H-1$_{(2^{Gal}\text{-}O\text{-}\beta\text{-Glc})}$ in compound **5** (methanol-*d*4, 500.18 MHz).

Compound 6

Figure S54. 1D ^{1}H-NMR spectrum of compound **6** (methanol-*d*4, 500.18 MHz).
Figure S55. 1D ^{13}C-NMR spectra (1H decoupled and DEPT-135) of compound **6** (methanol-*d*4, 125.77 MHz).
Figure S56. 2D *g*-COSY NMR spectrum of compound **6** (methanol-*d*4, 500.18 MHz).
Figure S57. 2D TOCSY NMR spectrum of compound **6** (methanol-*d*4, 500.18 MHz).
Figure S58. 2D ROESY NMR spectrum of compound **6** (methanol-*d*4, 500.18 MHz).
Figure S59. 2D *g*-HSQC-NMR spectrum of compound **6** (methanol-*d*4, 500.18 MHz, 125.77 MHz).
Figure S60. 2D *g*-HSQC-TOCSY NMR spectrum of compound **6** (methanol-*d*4, 500.18 MHz, 125.77 MHz).
Figure S61. 2D *g*-HMBC-NMR spectrum of compound **6** (methanol-*d*4, 500.18 MHz, 125.77 MHz).
Figure S62. 1D TOCSY and 1D ROESY NMR subspectra of H-1$_{(7\text{-}O\text{-}\beta\text{-GlcA})}$ in compound **6** (methanol-*d*4, 500.18 MHz).
Figure S63. 1D TOCSY and 1D ROESY NMR subspectra of H-1$_{(3\text{-}O\text{-}\beta\text{-Gal})}$ in compound **6** (methanol-*d*4, 500.18 MHz).
Figure S64. 1D TOCSY and 1D ROESY NMR subspectra of H-1$_{(2^{Gal}\text{-}O\text{-}\beta\text{-Glc})}$ in compound **6** (methanol-*d*4, 500.18 MHz).

Compound 7

Figure S65. 1D ^{1}H-NMR spectrum of compound **7** (methanol-*d*4, 500.18 MHz).
Figure S66. 1D ^{13}C-NMR spectra (1H decoupled and DEPT-135) of compound **7** (methanol-*d*4, 125.77 MHz).
Figure S67. 2D *g*-COSY NMR spectrum of compound **7** (methanol-*d*4, 500.18 MHz).
Figure S68. 2D TOCSY NMR spectrum of compound **7** (methanol-*d*4, 500.18 MHz).
Figure S69. 2D ROESY NMR spectrum of compound **7** (methanol-*d*4, 500.18 MHz).
Figure S70. 2D *g*-HSQC-NMR spectrum of compound **7** (methanol-*d*4, 500.18 MHz, 125.77 MHz).
Figure S71. 2D *g*-HSQC-TOCSY NMR spectrum of compound **7** (methanol-*d*4, 500.18 MHz, 125.77 MHz).
Figure S72. 2D *g*-HMBC-NMR spectrum of compound **7** (methanol-*d*4, 500.18 MHz, 125.77 MHz).
Figure S73. 1D TOCSY and 1D ROESY NMR subspectra of H-1$_{(7\text{-}O\text{-}\beta\text{-GlcA})}$ in compound **7** (methanol-*d*4, 500.18 MHz).
Figure S74. 1D TOCSY and 1D ROESY NMR subspectra of H-1$_{(3\text{-}O\text{-}\beta\text{-Gal})}$ in compound **7** (methanol-*d*4, 500.18 MHz).
Figure S75. 1D TOCSY and 1D ROESY NMR subspectra of H-1$_{(2^{Gal}\text{-}O\text{-}\beta\text{-Glc})}$ in compound **7** (methanol-*d*4, 500.18 MHz).
Figure S76. 1D ^{1}H-NMR spectrum of compound **8** (methanol-*d*4, 500.18 MHz).

Compound 8

Figure S77. 1D ^{13}C-NMR spectra (1H decoupled and DEPT-135) of compound **8** (methanol-*d*4, 125.77 MHz).
Figure S78. 2D *g*-COSY NMR spectrum of compound **8** (methanol-*d*4, 500.18 MHz).
Figure S79. 2D TOCSY NMR spectrum of compound **8** (methanol-*d*4, 500.18 MHz).
Figure S80. 2D ROESY NMR spectrum of compound **8** (methanol-*d*4, 500.18 MHz).
Figure S81. 2D *g*-HSQC-NMR spectrum of compound **8** (methanol-*d*4, 500.18 MHz, 125.77 MHz).
Figure S82. 2D *g*-HSQC-TOCSY NMR spectrum of compound **8** (methanol-*d*4, 500.18 MHz, 125.77 MHz).
Figure S83. 2D *g*-HMBC-NMR spectrum of compound **8** (methanol-*d*4, 500.18 MHz, 125.77 MHz).
Figure S84. 1D TOCSY and 1D ROESY NMR subspectra of H-1$_{(7\text{-}O\text{-}\beta\text{-GlcA})}$ in compound **8** (methanol-*d*4, 500.18 MHz).
Figure S85. 1D TOCSY and 1D ROESY NMR subspectra of H-1$_{(3\text{-}O\text{-}\beta\text{-Gal})}$ in compound **8** (methanol-*d*4, 500.18 MHz).
Figure S86. 1D TOCSY and 1D ROESY NMR subspectra of H-1$_{(2^{Gal}\text{-}O\text{-}\beta\text{-Glc})}$ in compound **8** (methanol-*d*4, 500.18 MHz).

Compound 9

Figure S87. 1D ^{1}H-NMR spectrum of compound **9** (methanol-*d*4, 500.18 MHz).
Figure S88. 1D ^{13}C-NMR spectra (1H decoupled and DEPT-135) of compound **9** (methanol-*d*4, 125.77 MHz).
Figure S89. 2D *g*-COSY NMR spectrum of compound **9** (methanol-*d*4, 500.18 MHz).
Figure S90. 2D TOCSY NMR spectrum of compound **9** (methanol-*d*4, 500.18 MHz).
Figure S91. 2D ROESY NMR spectrum of compound **9** (methanol-*d*4, 500.18 MHz).
Figure S92. 2D *g*-HSQC-NMR spectrum of compound **9** (methanol-*d*4, 500.18 MHz, 125.77 MHz).
Figure S93. 2D *g*-HSQC-TOCSY NMR spectrum of compound **9** (methanol-*d*4, 500.18 MHz, 125.77 MHz).
Figure S94. 2D *g*-HMBC-NMR spectrum of compound **9** (methanol-*d*4, 500.18 MHz, 125.77 MHz).
Figure S95. 1D TOCSY and 1D ROESY NMR subspectra of H-1$_{(7\text{-}O\text{-}\beta\text{-GlcA})}$ in compound **9** (methanol-*d*4, 500.18 MHz).
Figure S96. 1D TOCSY and 1D ROESY NMR subspectra of H-1$_{(3\text{-}O\text{-}\beta\text{-Gal})}$ in compound **9** (methanol-*d*4, 500.18 MHz).
Figure S97. 1D TOCSY and 1D ROESY NMR subspectra of H-1$_{(2^{Gal}\text{-}O\text{-}\beta\text{-Glc})}$ in compound **9** (methanol-*d*4, 500.18 MHz).

Compound 10

Figure S98. 1D ^{1}H-NMR spectrum of compound **10** (methanol-*d*4, 500.18 MHz).
Figure S99. 1D ^{13}C-NMR spectra (1H decoupled and DEPT-135) of compound **10** (methanol-*d*4, 125.77 MHz).
Figure S100. 2D *g*-COSY NMR spectrum of compound **10** (methanol-*d*4, 500.18 MHz).

Figure S101. 2D TOCSY NMR spectrum of compound **10** (methanol-d_4, 500.18 MHz).
Figure S102. 2D ROESY NMR spectrum of compound **10** (methanol-d_4, 500.18 MHz).
Figure S103. 2D *g*-HSQC-NMR spectrum of compound **10** (methanol-d_4, 500.18 MHz, 125.77 MHz).
Figure S104. 2D *g*-HSQC-TOCSY NMR spectrum of compound **10** (methanol-d_4, 500.18 MHz, 125.77 MHz).
Figure S105. 2D *g*-HMBC-NMR spectrum of compound **10** (methanol-d_4, 500.18 MHz, 125.77 MHz).
Figure S106. 1D TOCSY and 1D ROESY NMR subspectra of H-$1_{(7\text{-}O\text{-}\beta\text{-GlcA})}$ in compound **10** (methanol-d_4, 500.18 MHz).
Figure S107. 1D TOCSY and 1D ROESY NMR subspectra of H-$1_{(3\text{-}O\text{-}\beta\text{-Gal})}$ in compound **10** (methanol-d_4, 500.18 MHz).
Figure S108. 1D TOCSY and 1D ROESY NMR subspectra of H-$1_{(2^{Gal}\text{-}O\text{-}\beta\text{-Glc})}$ in compound **10** (methanol-d_4, 500.18 MHz).

Compound 11

Figure S109. 1D ^{1}H-NMR spectrum of compound **11** (methanol-d_4, 500.18 MHz).
Figure S110. 1D ^{13}C-NMR spectra (1H decoupled and DEPT-135) of compound **11** (methanol-d_4, 125.77 MHz).
Figure S111. 2D *g*-COSY NMR spectrum of compound **11** (methanol-d_4, 500.18 MHz).
Figure S112. 2D TOCSY NMR spectrum of compound **11** (methanol-d_4, 500.18 MHz).
Figure S113. 2D ROESY NMR spectrum of compound **11** (methanol-d_4, 500.18 MHz).
Figure S114. 2D *g*-HSQC-NMR spectrum of compound **11** (methanol-d_4, 500.18 MHz, 125.77 MHz).
Figure S115. 2D *g*-HSQC-TOCSY NMR spectrum of compound **11** (methanol-d_4, 500.18 MHz, 125.77 MHz).
Figure S116. 2D *g*-HMBC-NMR spectrum of compound **11** (methanol-d_4, 500.18 MHz, 125.77 MHz).
Figure S117. 1D TOCSY and 1D ROESY NMR subspectra of H-$1_{(7\text{-}O\text{-}\alpha\text{-Rha})}$ and H-$6_{(7\text{-}O\text{-}\alpha\text{-Rha})}$ in compound **11** (methanol-d_4, 500.18 MHz).
Figure S118. 1D TOCSY and 1D ROESY NMR subspectra of H-$1_{(3\text{-}O\text{-}\beta\text{-Gal})}$ in compound **11** (methanol-d_4, 500.18 MHz).
Figure S119. 1D TOCSY and 1D ROESY NMR subspectra of H-$1_{(2^{Gal}\text{-}O\text{-}\beta\text{-Glc})}$ in compound **11** (methanol-d_4, 500.18 MHz).
Figure S120. 1D TOCSY and 1D ROESY NMR subspectra of H-$1_{(6^{Gal}\text{-}O\text{-}\alpha\text{-Rha})}$ and H-$6_{(6^{Gal}\text{-}O\text{-}\alpha\text{-Rha})}$ in compound **11** (methanol-d_4, 500.18 MHz).

Compound 12

Figure S121. 1D ^{1}H-NMR spectrum of compound **12** (methanol-d_4, 500.18 MHz).
Figure S122. 1D ^{13}C-NMR spectra (1H decoupled and DEPT-135) of compound **12** (methanol-d_4, 125.77 MHz).
Figure S123. 2D *g*-COSY NMR spectrum of compound **12** (methanol-d_4, 500.18 MHz).
Figure S124. 2D TOCSY NMR spectrum of compound **12** (methanol-d_4, 500.18 MHz).

Figure S125. 2D ROESY NMR spectrum of compound **12** (methanol-d_4, 500.18 MHz).
Figure S126. 2D *g*-HSQC-NMR spectrum of compound **12** (methanol-d_4, 500.18 MHz, 125.77 MHz).
Figure S127. 2D *g*-HSQC-TOCSY NMR spectrum of compound **12** (methanol-d_4, 500.18 MHz, 125.77 MHz).
Figure S128. 2D *g*-HMBC-NMR spectrum of compound **12** (methanol-d_4, 500.18 MHz, 125.77 MHz).
Figure S129. 1D TOCSY and 1D ROESY NMR subspectra of H-1$_{(7\text{-}O\text{-}\beta\text{-GlcA})}$ in compound **12** (methanol-d_4, 500.18 MHz).
Figure S130. 1D TOCSY NMR subspectrum of H-2$_{(3\text{-}O\text{-}\beta\text{-Gal})}$ in compound **12** (methanol-d_4, 500.18 MHz).
Figure S131. 1D TOCSY and 1D ROESY NMR subspectra of H-1$_{(2^{Gal}\text{-}O\text{-}\beta\text{-Glc})}$ in compound **12** (methanol-d_4, 500.18 MHz).

Compound 13

Figure S132. 1D ^{1}H-NMR spectrum of compound **13** (methanol-d_4, 500.18 MHz).
Figure S133. 1D ^{13}C-NMR spectra (1H decoupled and DEPT-135) of compound **13** (methanol-d_4, 125.77 MHz).
Figure S134. 2D *g*-COSY NMR spectrum of compound **13** (methanol-d_4, 500.18 MHz).
Figure S135. 2D TOCSY NMR spectrum of compound **13** (methanol-d_4, 500.18 MHz).
Figure S136. 2D ROESY NMR spectrum of compound **13** (methanol-d_4, 500.18 MHz).
Figure S137. 2D *g*-HSQC-NMR spectrum of compound **13** (methanol-d_4, 500.18 MHz, 125.77 MHz).
Figure S138. 2D *g*-HSQC-TOCSY NMR spectrum of compound **13** (methanol-d_4, 500.18 MHz, 125.77 MHz).
Figure S139. 2D *g*-HMBC-NMR spectrum of compound **13** (methanol-d_4, 500.18 MHz, 125.77 MHz).
Figure S140. 1D TOCSY and 1D ROESY NMR subspectra of H-1$_{(7\text{-}O\text{-}\beta\text{-GlcA})}$ in compound **13** (methanol-d_4, 500.18 MHz).
Figure S141. 1D TOCSY and 1D ROESY NMR subspectra of H-1$_{(3\text{-}O\text{-}\beta\text{-Gal})}$ in compound **13** (methanol-d_4, 500.18 MHz).
Figure S142. 1D TOCSY and 1D ROESY NMR subspectra of H-1$_{(2^{Gal}\text{-}O\text{-}\beta\text{-Glc})}$ in compound **13** (methanol-d_4, 500.18 MHz).

Compound 14

Figure S143. 1D ^{1}H-NMR spectrum of compound **14** (methanol-d_4, 500.18 MHz).
Figure S144. 1D ^{13}C-NMR spectra (1H decoupled and DEPT-135) of compound **14** (methanol-d_4, 125.77 MHz).
Figure S145. 2D *g*-COSY NMR spectrum of compound **14** (methanol-d_4, 500.18 MHz).
Figure S146. 2D TOCSY NMR spectrum of compound **14** (methanol-d_4, 500.18 MHz).
Figure S147. 2D ROESY NMR spectrum of compound **14** (methanol-d_4, 500.18 MHz).
Figure S148. 2D *g*-HSQC-NMR spectrum of compound **14** (methanol-d_4, 500.18 MHz, 125.77 MHz).

Figure S149. 2D *g*-HSQC-TOCSY NMR spectrum of compound **14** (methanol-d_4, 500.18 MHz, 125.77 MHz).
Figure S150. 2D *g*-HMBC-NMR spectrum of compound **14** (methanol-d_4, 500.18 MHz, 125.77 MHz).
Figure S151. 1D TOCSY and 1D ROESY NMR subspectra of H-$1_{(7\text{-}O\text{-}\beta\text{-GlcA})}$ in compound **14** (methanol-d_4, 500.18 MHz).
Figure S152. 1D TOCSY and 1D ROESY NMR subspectra of H-$1_{(3\text{-}O\text{-}\beta\text{-Gal})}$ in compound **14** (methanol-d_4, 500.18 MHz).
Figure S153. 1D TOCSY and 1D ROESY NMR subspectra of H-$1_{(2^{Gal}\text{-}O\text{-}\beta\text{-Glc})}$ in compound **14** (methanol-d_4, 500.18 MHz).

Compound 15

Figure S154. 1D ^{1}H-NMR spectrum of compound **15** (methanol-d_4, 500.18 MHz).
Figure S155. 1D ^{13}C-NMR spectra (1H decoupled and DEPT-135) of compound **15** (methanol-d_4, 125.77 MHz).
Figure S156. 2D *g*-COSY NMR spectrum of compound **15** (methanol-d_4, 500.18 MHz).
Figure S157. 2D TOCSY NMR spectrum of compound **15** (methanol-d_4, 500.18 MHz).
Figure S158. 2D ROESY NMR spectrum of compound **15** (methanol-d_4, 500.18 MHz).
Figure S159. 2D *g*-HSQC-NMR spectrum of compound **15** (methanol-d_4, 500.18 MHz, 125.77 MHz).
Figure S160. 2D *g*-HSQC-TOCSY NMR spectrum of compound **15** (methanol-d_4, 500.18 MHz, 125.77 MHz).
Figure S161. 2D *g*-HMBC-NMR spectrum of compound **15** (methanol-d_4, 500.18 MHz, 125.77 MHz).
Figure S162. 1D TOCSY and 1D ROESY NMR subspectra of H-$1_{(7\text{-}O\text{-}\beta\text{-GlcA})}$ in compound **15** (methanol-d_4, 500.18 MHz).
Figure S163. 1D TOCSY NMR subspectrum of H-$2_{(3\text{-}O\text{-}\beta\text{-Gal})}$ in compound **15** (methanol-d_4, 500.18 MHz).
Figure S164. 1D TOCSY and 1D ROESY NMR subspectra of H-$1_{(2^{Gal}\text{-}O\text{-}\beta\text{-Glc})}$ in compound **15** (methanol-d_4, 500.18 MHz).

Compound 16

Figure S165. 1D ^{1}H-NMR spectrum of compound **16** (methanol-d_4, 500.18 MHz).
Figure S166. 1D ^{13}C-NMR spectra (1H decoupled and DEPT-135) of compound **16** (methanol-d_4, 125.77 MHz).
Figure S167. 2D *g*-COSY NMR spectrum of compound **16** (methanol-d_4, 500.18 MHz).
Figure S168. 2D ROESY NMR spectrum of compound **16** (methanol-d_4, 500.18 MHz).
Figure S169. 2D *g*-HSQC-NMR spectrum of compound **16** (methanol-d_4, 500.18 MHz, 125.77 MHz).
Figure S170. 2D *g*-HMBC-NMR spectrum of compound **16** (methanol-d_4, 500.18 MHz, 125.77 MHz).
Figure S171. 1D TOCSY and 1D ROESY NMR subspectra of H-$1_{(3\text{-}O\text{-}\alpha\text{-Rha})}$ and H-$6_{(3\text{-}O\text{-}\alpha\text{-Rha})}$ in compound **16** (methanol-d_4, 500.18 MHz).

Compound 17

Figure S172. 1D ^{1}H-NMR spectrum of compound **17** (methanol-*d*4, 500.18 MHz).
Figure S173. 1D ^{13}C-NMR spectra (1H decoupled and DEPT-135) of compound **17** (methanol-*d*4, 125.77 MHz).
Figure S174. 2D *g*-COSY NMR spectrum of compound **17** (methanol-*d*4, 500.18 MHz).
Figure S175. 2D TOCSY NMR spectrum of compound **17** (methanol-*d*4, 500.18 MHz).
Figure S176. 2D ROESY NMR spectrum of compound **17** (methanol-*d*4, 500.18 MHz).
Figure S177. 2D *g*-HSQC-NMR spectrum of compound **17** (methanol-*d*4, 500.18 MHz, 125.77 MHz).
Figure S178. 2D *g*-HSQC-TOCSY NMR spectrum of compound **17** (methanol-*d*4, 500.18 MHz, 125.77 MHz).
Figure S179. 2D *g*-HMBC-NMR spectrum of compound **17** (methanol-*d*4, 500.18 MHz, 125.77 MHz).
Figure S180. 1D TOCSY and 1D ROESY NMR subspectra of H-1$_{(7\text{-}O\text{-}\beta\text{-GlcA})}$ in compound **17** (methanol-*d*4, 500.18 MHz).
Figure S181. 1D TOCSY subspectrum of H-2$_{(3\text{-}O\text{-}\beta\text{-Gal})}$ in compound **17** (methanol-*d*4, 500.18 MHz).
Figure S182. 1D TOCSY and 1D ROESY NMR subspectra of H-1$_{(2^{Gal}\text{-}O\text{-}\beta\text{-Glc})}$ in compound **17** (methanol-*d*4, 500.18 MHz).

Compound 18

Figure S183. 1D ^{1}H-NMR spectrum of compound **18** (methanol-*d*4, 500.18 MHz).
Figure S184. 1D ^{13}C-NMR spectra (1H decoupled and DEPT-135) of compound **18** (methanol-*d*4, 125.77 MHz).
Figure S185. 2D *g*-COSY NMR spectrum of compound **18** (methanol-*d*4, 500.18 MHz).
Figure S186. 2D TOCSY NMR spectrum of compound **18** (methanol-*d*4, 500.18 MHz).
Figure S187. 2D ROESY NMR spectrum of compound **18** (methanol-*d*4, 500.18 MHz).
Figure S188. 2D *g*-HSQC-NMR spectrum of compound **18** (methanol-*d*4, 500.18 MHz, 125.77 MHz).
Figure S189. 2D *g*-HSQC-TOCSY NMR spectrum of compound **18** (methanol-*d*4, 500.18 MHz, 125.77 MHz).
Figure S190. 2D *g*-HMBC-NMR spectrum of compound **18** (methanol-*d*4, 500.18 MHz, 125.77 MHz).
Figure S191. 1D TOCSY and 1D ROESY NMR subspectra of H-1$_{(7\text{-}O\text{-}\beta\text{-GlcA})}$ in compound **18** (methanol-*d*4, 500.18 MHz).
Figure S192. 1D TOCSY and 1D ROESY NMR subspectra of H-1$_{(3\text{-}O\text{-}\beta\text{-Gal})}$ in compound **18** (methanol-*d*4, 500.18 MHz).
Figure S193. 1D TOCSY and 1D ROESY NMR subspectra of H-1$_{(2^{Gal}\text{-}O\text{-}\beta\text{-Glc})}$ in compound **18** (methanol-*d*4, 500.18 MHz).
Figure S194. UPLC-MS chromatograms of phenolic acid reference standards.
Figure S195. UPLC-MS chromatograms of phenolic acids released during alkaline hydrolysis of the compound **4**.
Figure S196. The UV spectrum of the putative 3,4,5-trihydroxycinnamic acid.

Figure S197. UPLC-MS chromatograms of phenolic acids released during alkaline hydrolysis of the compound **5**.
Figure S198. UPLC-MS chromatograms of phenolic acids released during alkaline hydrolysis of the compound **6**.
Figure S199. UPLC-MS chromatograms of phenolic acids released during alkaline hydrolysis of the compound **7**.
Figure S200. UPLC-MS chromatograms of phenolic acids released during alkaline hydrolysis of the compound **8**.
Figure S201. UPLC-MS chromatograms of phenolic acids released during alkaline hydrolysis of the compound **9**.
Figure S202. UPLC-MS chromatograms of phenolic acids released during alkaline hydrolysis of the compound **10**.
Figure S203. UPLC-MS chromatograms of phenolic acids released during alkaline hydrolysis of the compound **11**.
Figure S204. UPLC-MS chromatograms of phenolic acids released during alkaline hydrolysis of the compound **12**.
Figure S205. UPLC-MS chromatograms of phenolic acids released during alkaline hydrolysis of the compound **13**.
Figure S206. UPLC-MS chromatograms of phenolic acids released during alkaline hydrolysis of the compound **14**.
Figure S207. UPLC-MS chromatograms of phenolic acids released during alkaline hydrolysis of the compound **15**.
Figure S208. UPLC-MS chromatograms of phenolic acids released during alkaline hydrolysis of the compound **17**.
Figure S209. UPLC-MS chromatograms of phenolic acids released during alkaline hydrolysis of the compound **18**.
Figure S210. UPLC-MS chromatograms of the sugar standards.
Figure S211. UPLC-MS chromatograms of sugars released during the acid hydrolysis of the compound **1**.
Figure S212. UPLC-MS chromatograms of sugars released during the acid hydrolysis of the compound **3**.
Figure S213. UPLC-MS chromatograms of sugars released during the acid hydrolysis of the compound **4**.
Figure S214. UPLC-MS chromatograms of sugars released during the acid hydrolysis of the compound **5**.
Figure S215. UPLC-MS chromatograms of sugars released during the acid hydrolysis of the compound **6**.
Figure S216. UPLC-MS chromatograms of sugars released during the acid hydrolysis of the compound **7**.
Figure S217. UPLC-MS chromatograms of sugars released during the acid hydrolysis of the compound **8**.

Figure S218. UPLC-MS chromatograms of sugars released during the acid hydrolysis of the compound **9**.
Figure S219. UPLC-MS chromatograms of sugars released during the acid hydrolysis of the compound **10.**
Figure S220. UPLC-MS chromatograms of sugars released during the acid hydrolysis of the compound **11**.
Figure S221. UPLC-MS chromatograms of sugars released during the acid hydrolysis of the compound **12**.
Figure S222. UPLC-MS chromatograms of sugars released during the acid hydrolysis of the compound **13**.
Figure S223. UPLC-MS chromatograms of sugars released during the acid hydrolysis of the compound **14**.
Figure S224. UPLC-MS chromatograms of sugars released during the acid hydrolysis of the compound **15**.
Figure S225. UPLC-MS chromatograms of sugars released during the acid hydrolysis of the compound **17**.
Figure S226. UPLC-MS chromatograms of sugars released during the acid hydrolysis of the compound **18.**
Figure S227. UPLC-MS chromatograms of the methanol extract of lentil seeds (var. Tina), and the UV spectrum of the dominant phenolic compound.

Compound 1

Figure S1. 1D ^{1}H-NMR spectrum of compound **1** (methanol-d_4, 500.18 MHz).

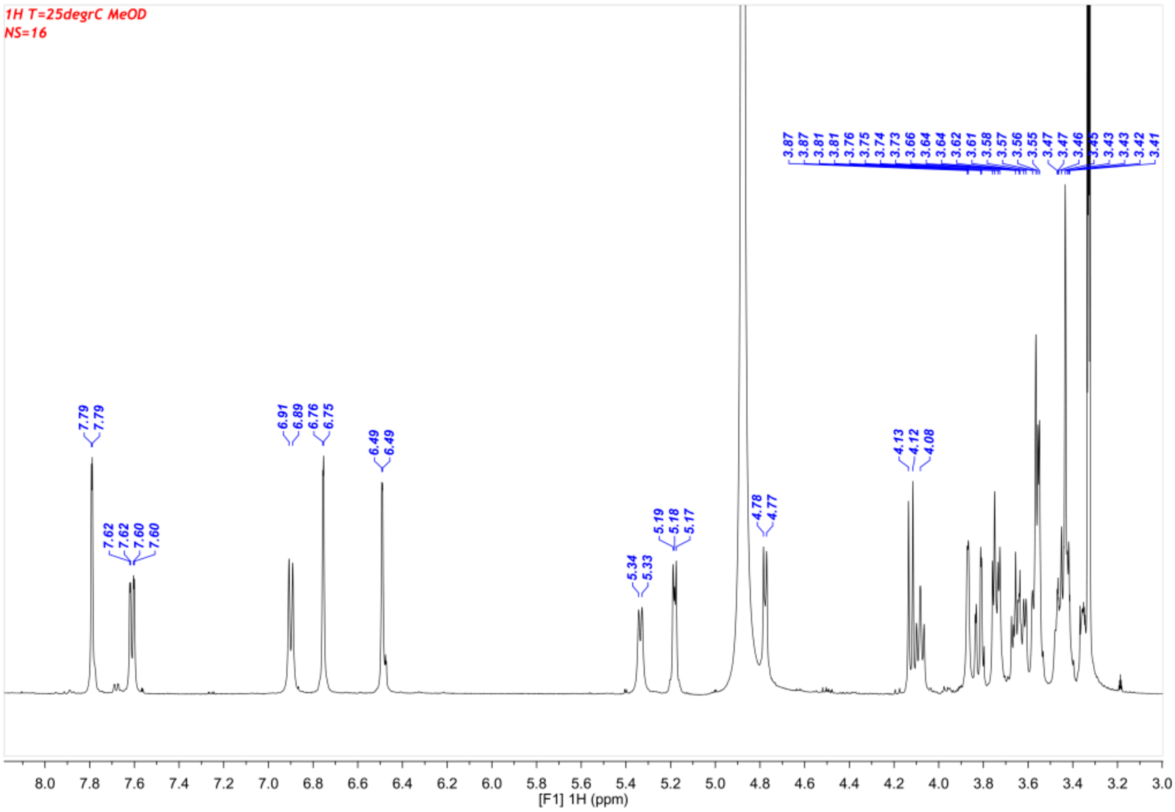

Figure S2. 1D ^{13}C-NMR spectra (^{1}H decoupled and DEPT-135) (methanol-d_4, 125.77 MHz).

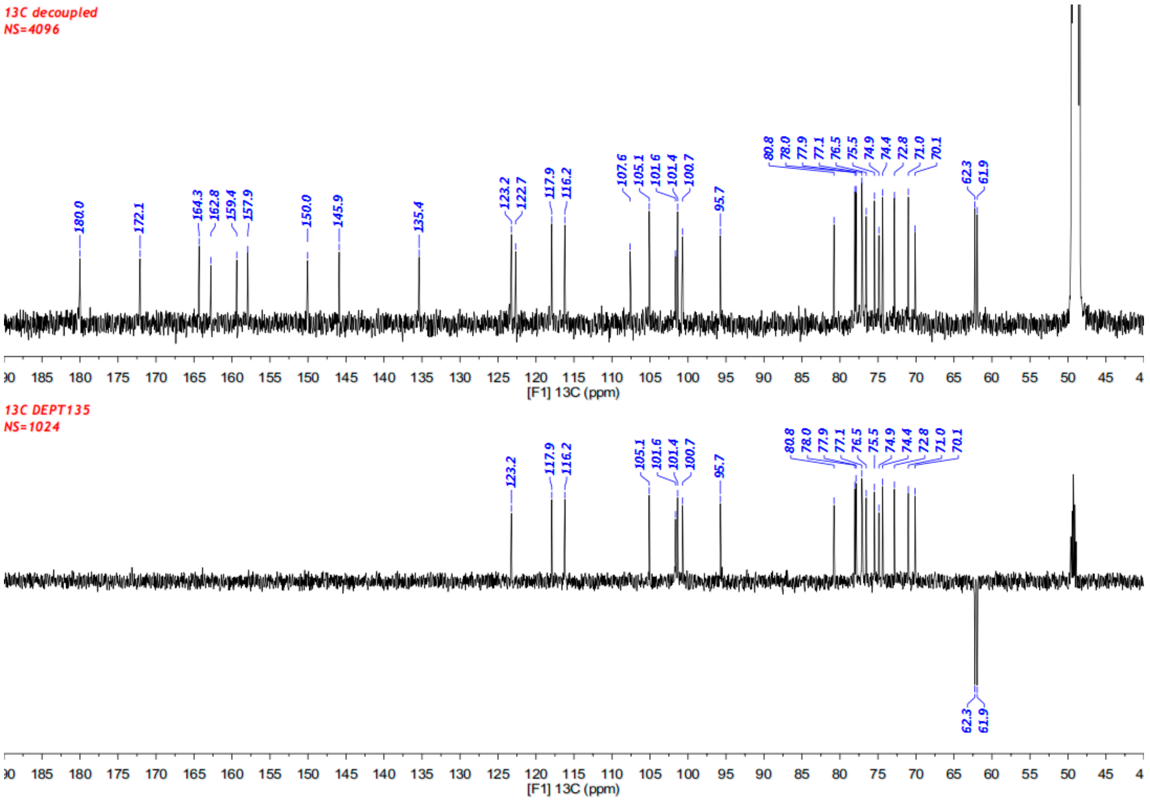

Figure S3. 2D *g*-COSY NMR spectrum of compound **1** (methanol-d_4, 500.18 MHz).

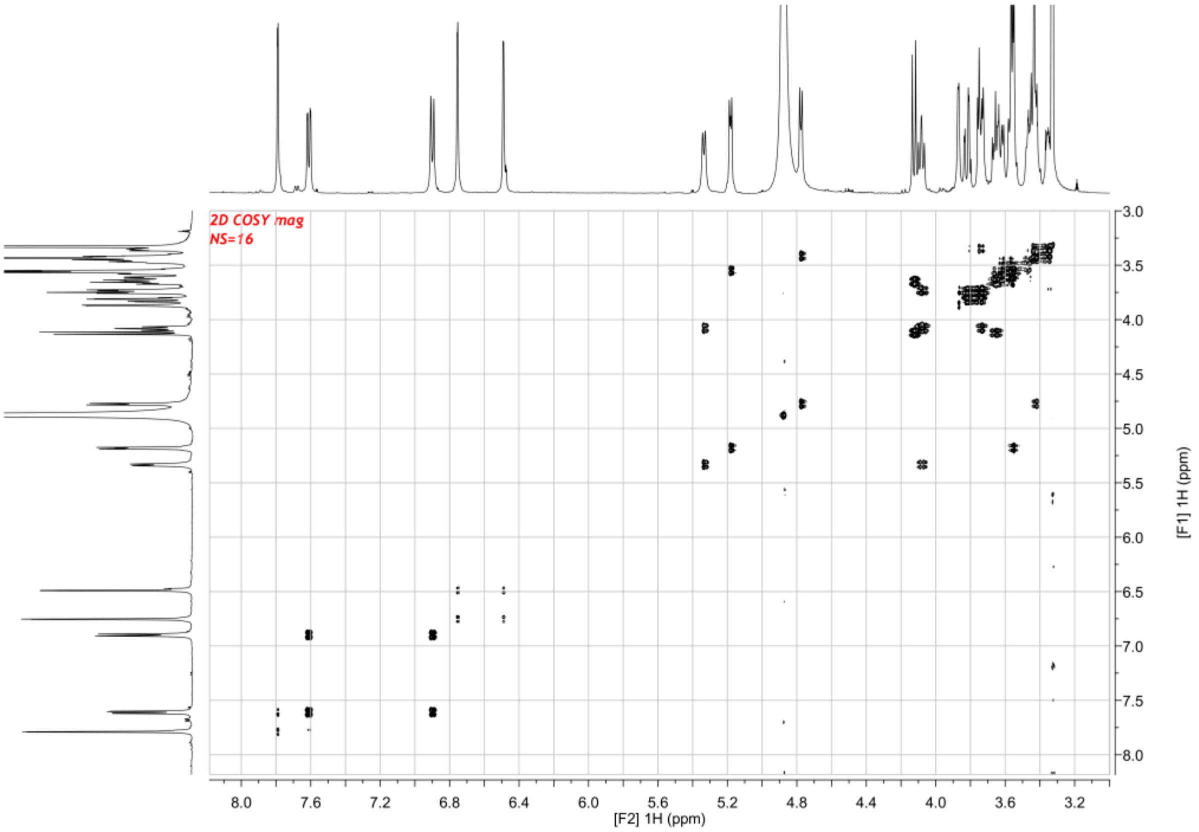

Figure S4. 2D TOCSY NMR spectrum of compound **1** (methanol-d_4, 500.18 MHz).

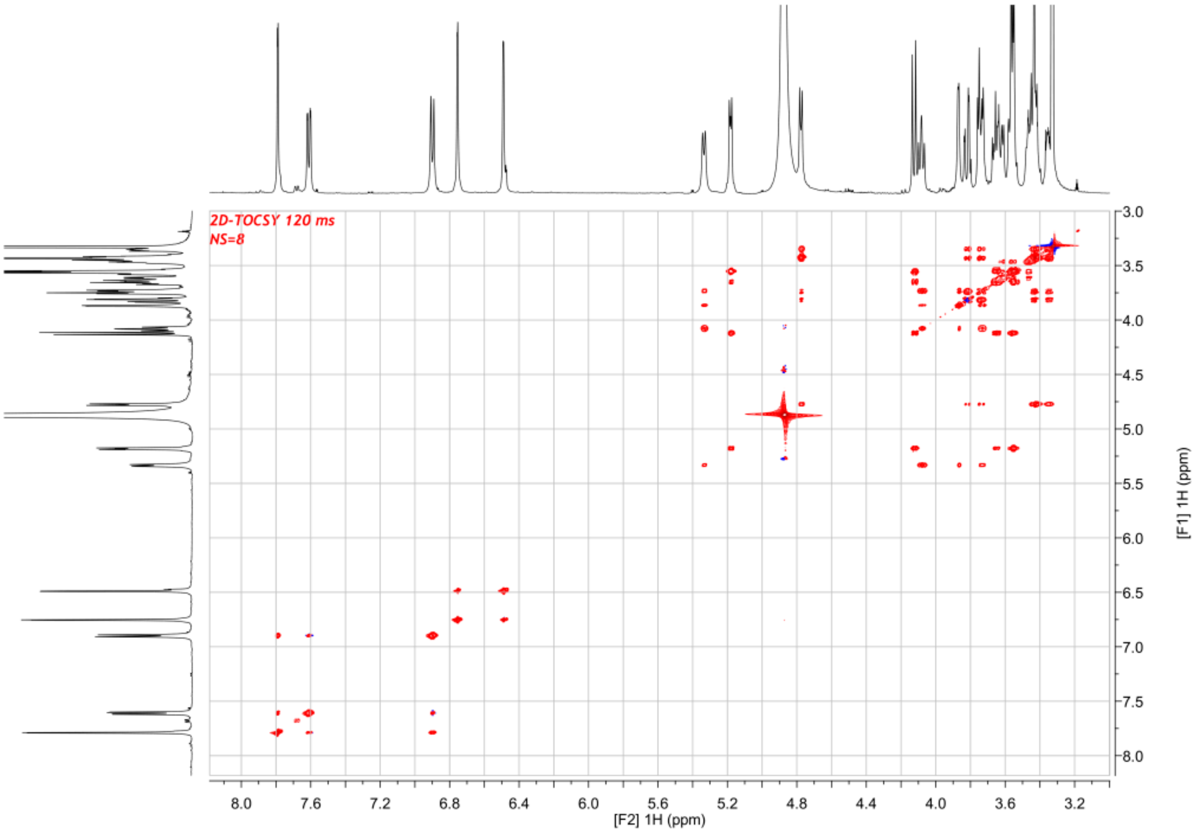

Figure S5. 2D ROESY NMR spectrum of compound **1** (methanol-*d*4, 500.18 MHz).

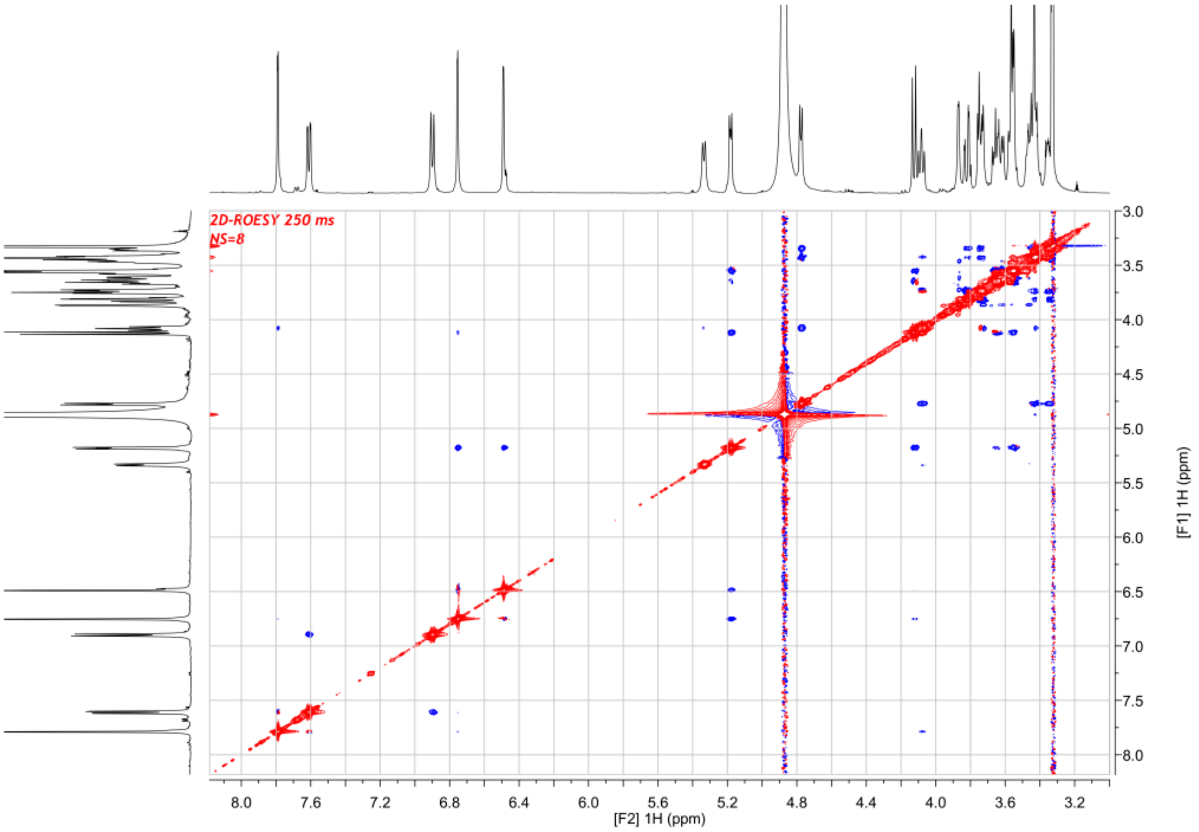

Figure S6. 2D *g*-HSQC-NMR spectrum of compound **1** (methanol-*d*4, 500.18 MHz, 125.77 MHz).

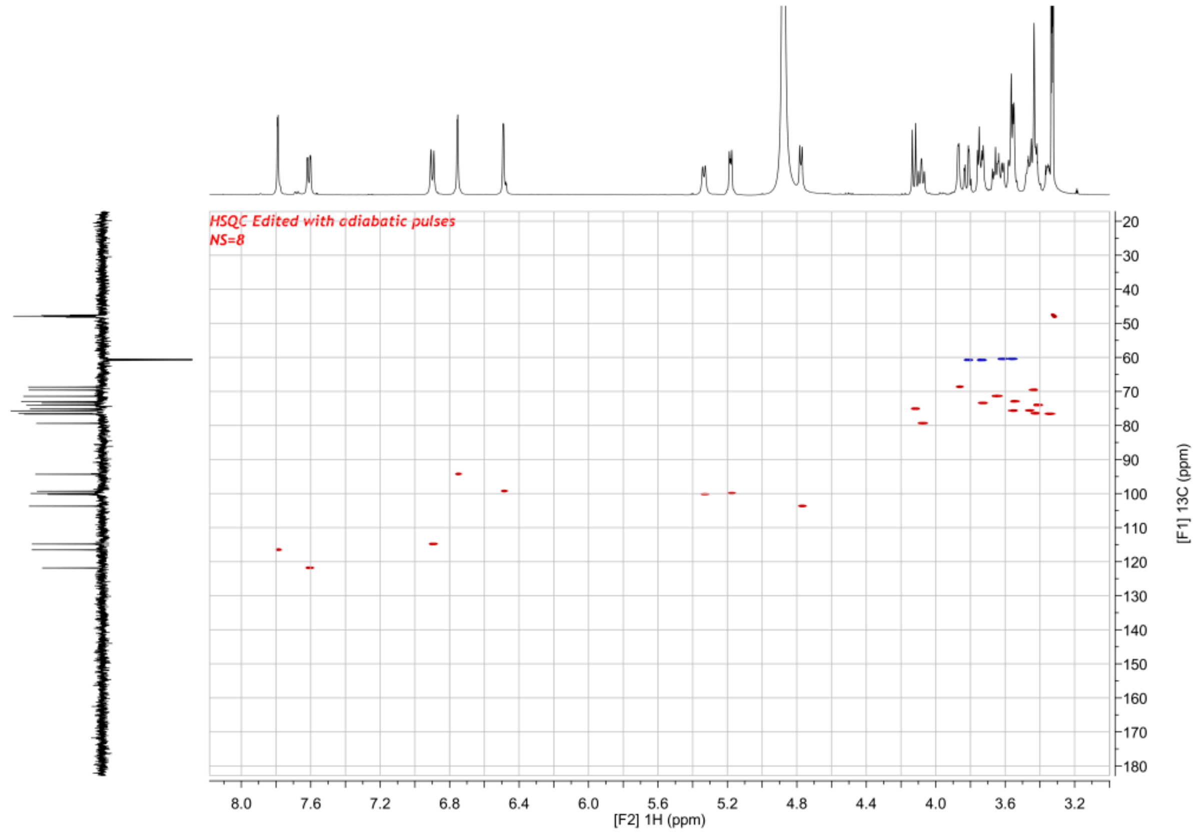

Figure S7. 2D *g*-HSQC-TOCSY NMR spectrum of compound **1** (methanol-*d*4, 500.18 MHz, 125.77 MHz).

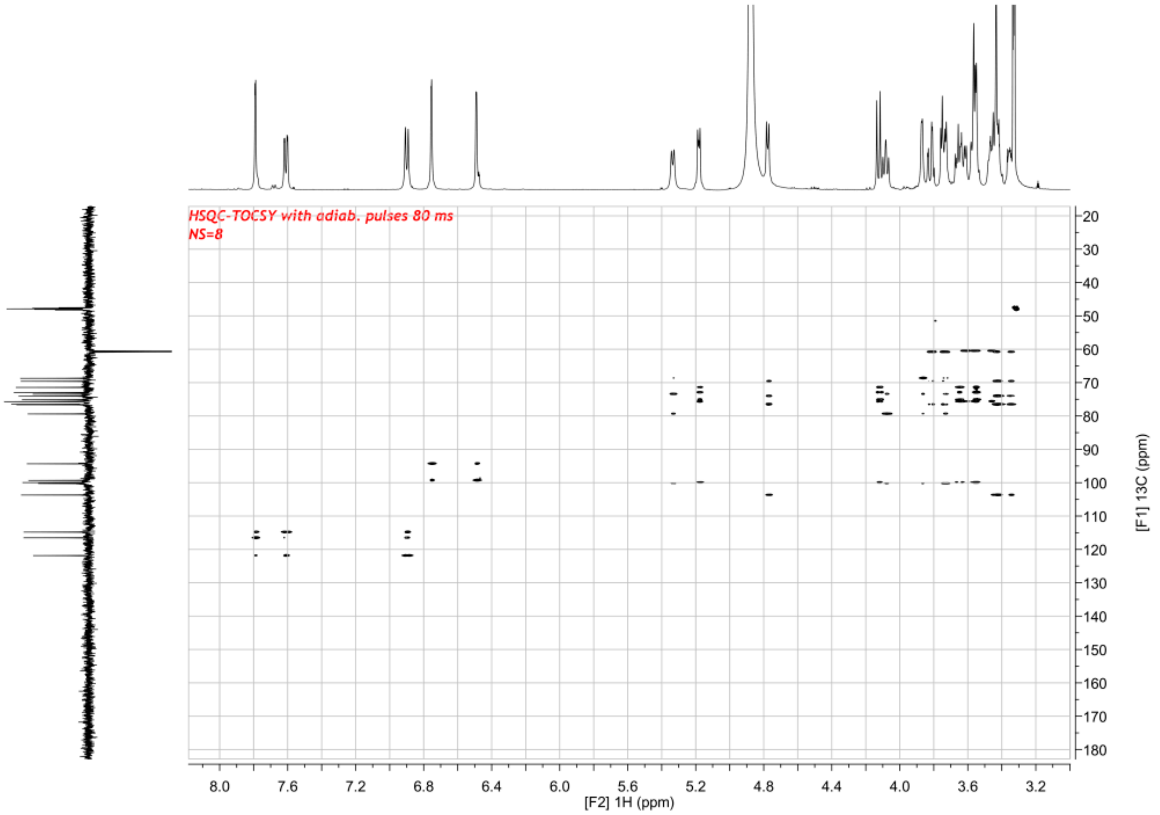

Figure S8. 2D *g*-HMBC-NMR spectrum of compound **1** (methanol-*d*4, 500.18 MHz, 125.77 MHz).

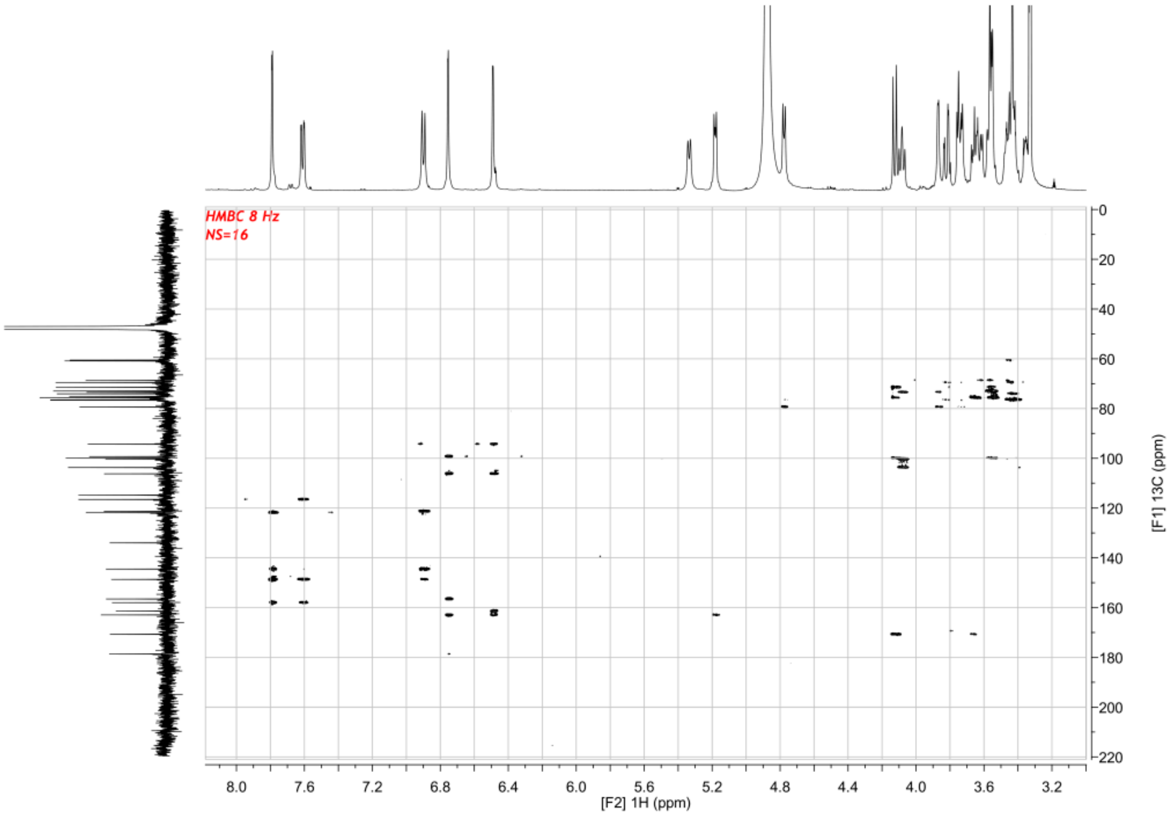

Figure S9. 1D TOCSY and 1D ROESY NMR subspectra of H-1$_{(7\text{-}O\text{-}\beta\text{-GlcA})}$ in compound **1** (methanol-d_4, 500.18 MHz).

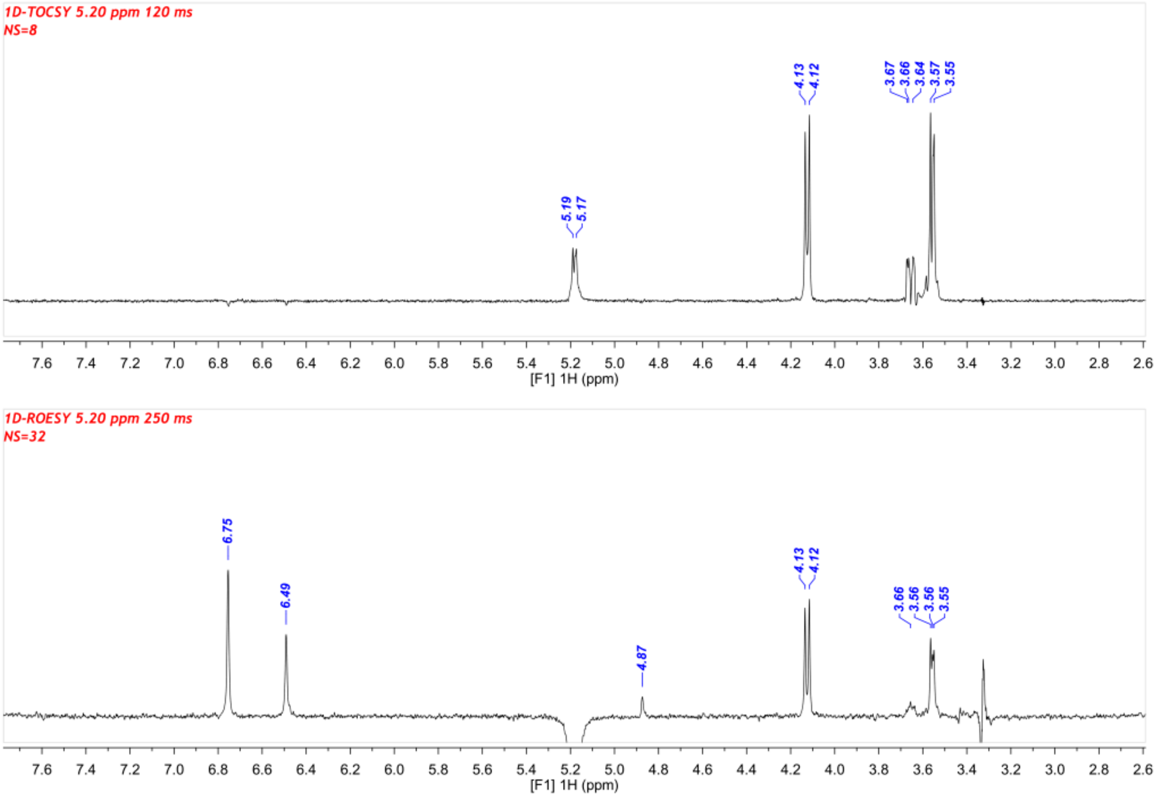

Figure S10. 1D TOCSY and 1D ROESY NMR subspectra of H-1$_{(3\text{-}O\text{-}\beta\text{-Gal})}$ in compound **1** (methanol-d_4, 500.18 MHz).

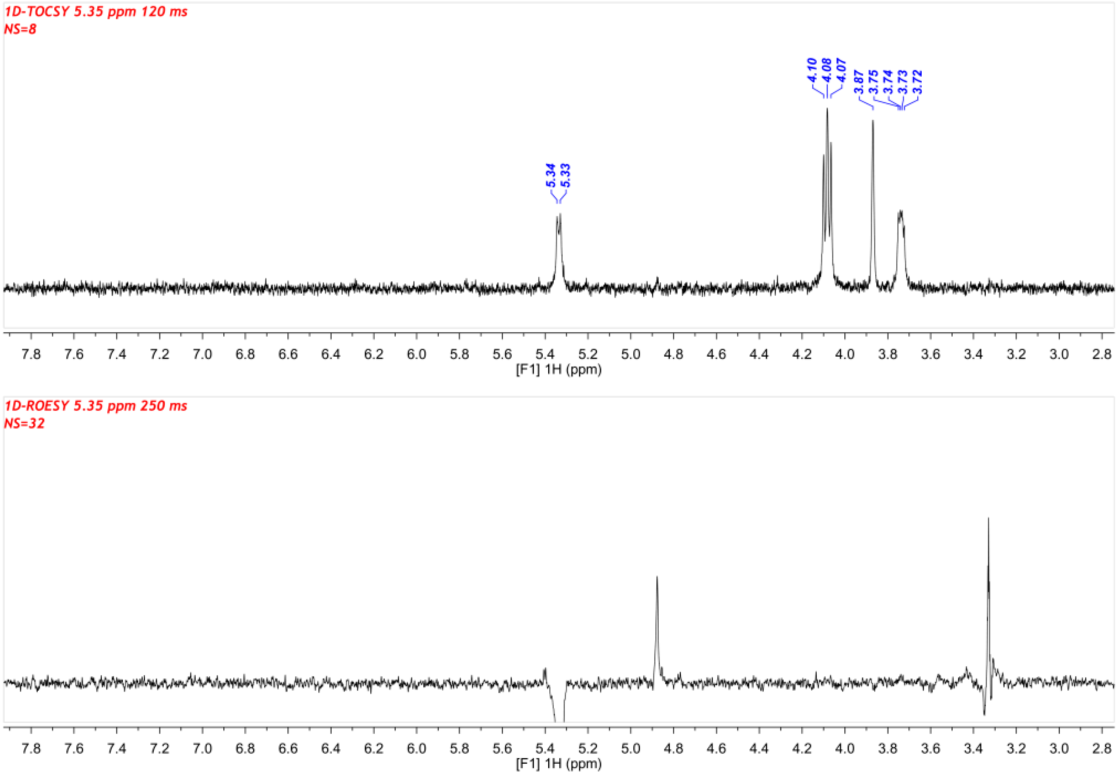

Figure S11. 1D TOCSY and 1D ROESY NMR subspectra of H-1$_{(2^{Gal}\text{-}O\text{-}\beta\text{-}Glc)}$ in compound **1** (methanol-d_4, 500.18 MHz).

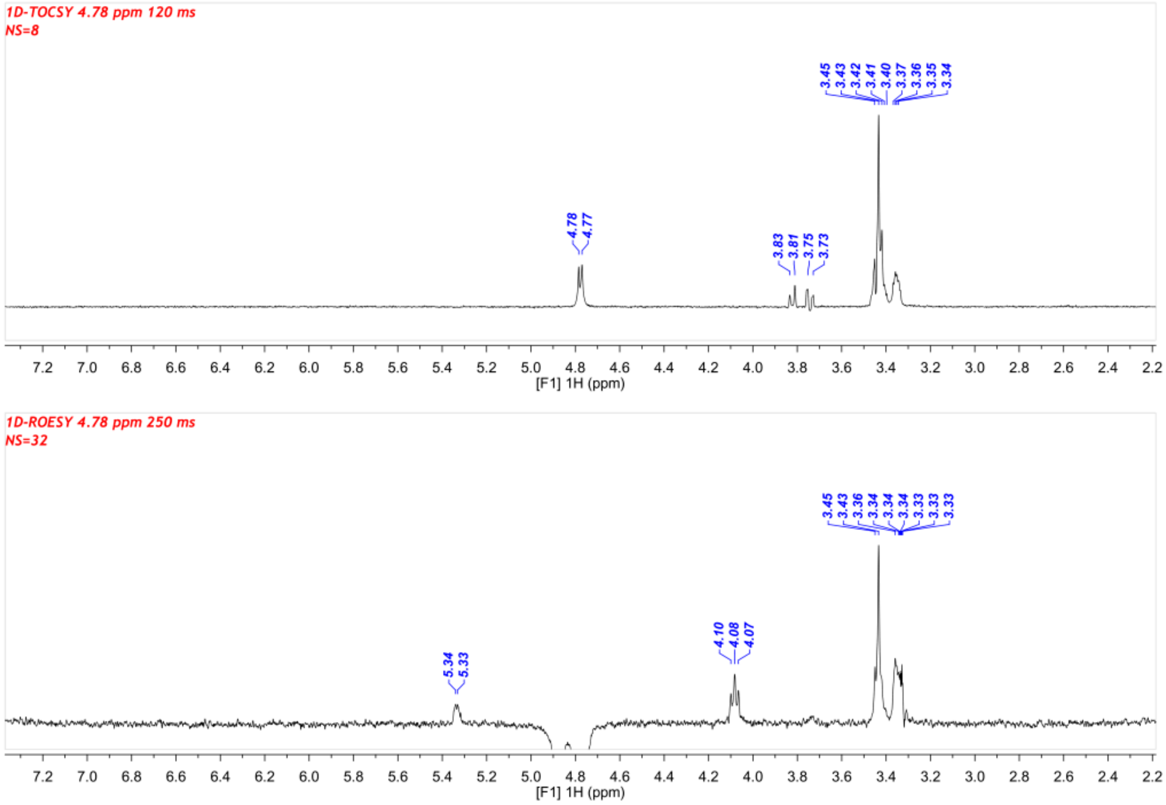

Compound 2

Figure S12. 1D ^{1}H-NMR spectrum of compound **2** (methanol-d_4, 500.18 MHz).

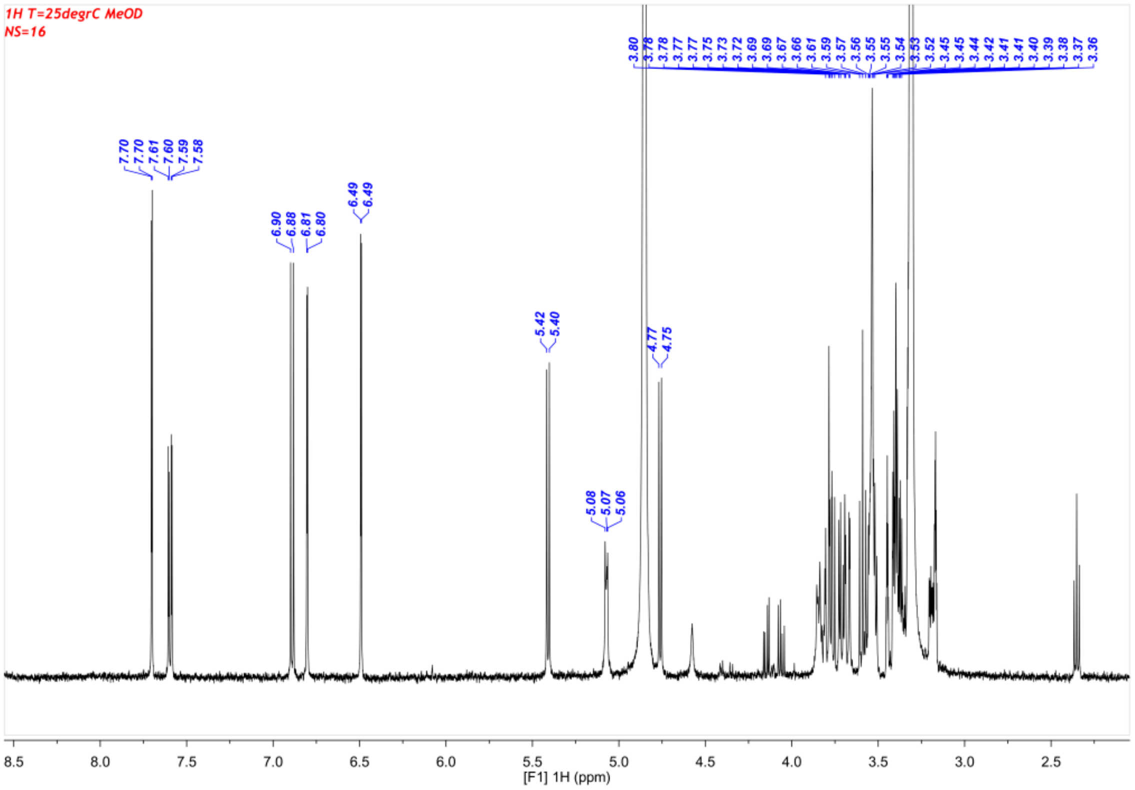

Figure S13. 1D ^{13}C-NMR spectrum (^{1}H decoupled) of compound **2** (methanol-*d*4, 125.77 MHz).

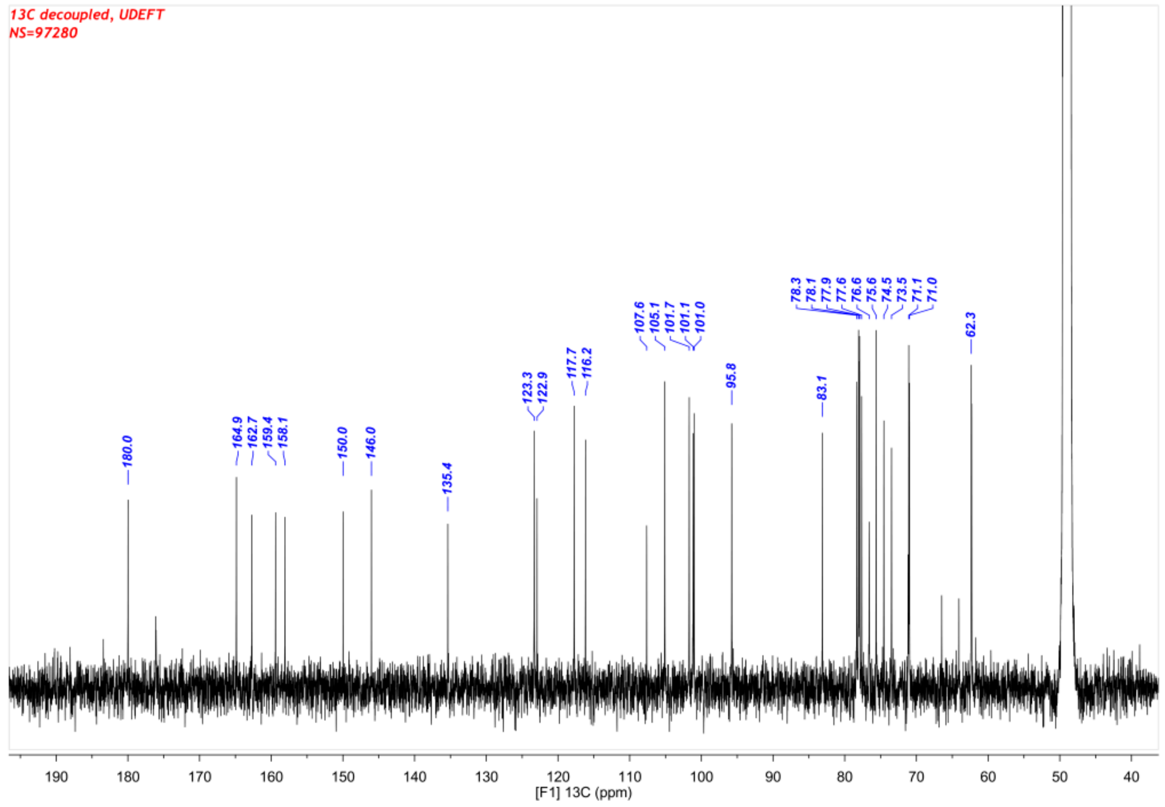

Figure S14. 2D *g*-COSY NMR spectrum of compound **2** (methanol-*d*4, 500.18 MHz).

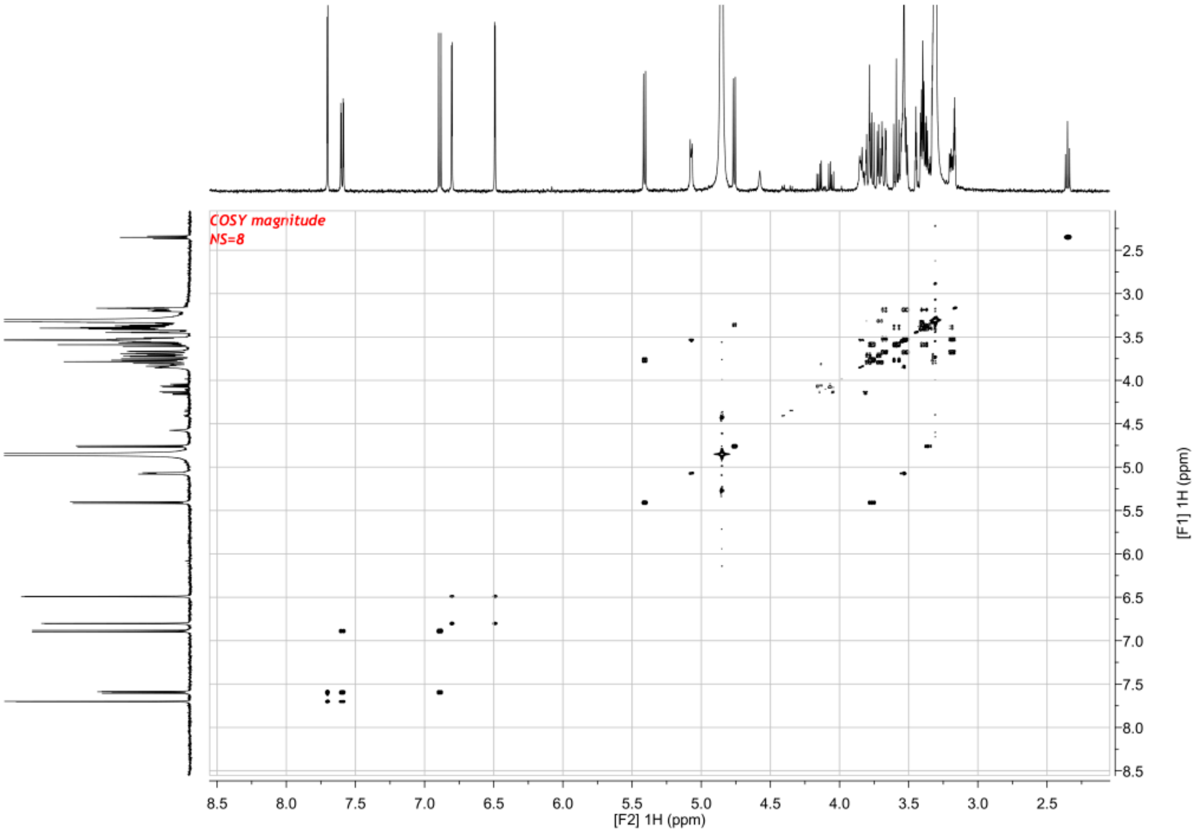

Figure S15. 2D TOCSY NMR spectrum of compound **2** (methanol-*d*4, 500.18 MHz).

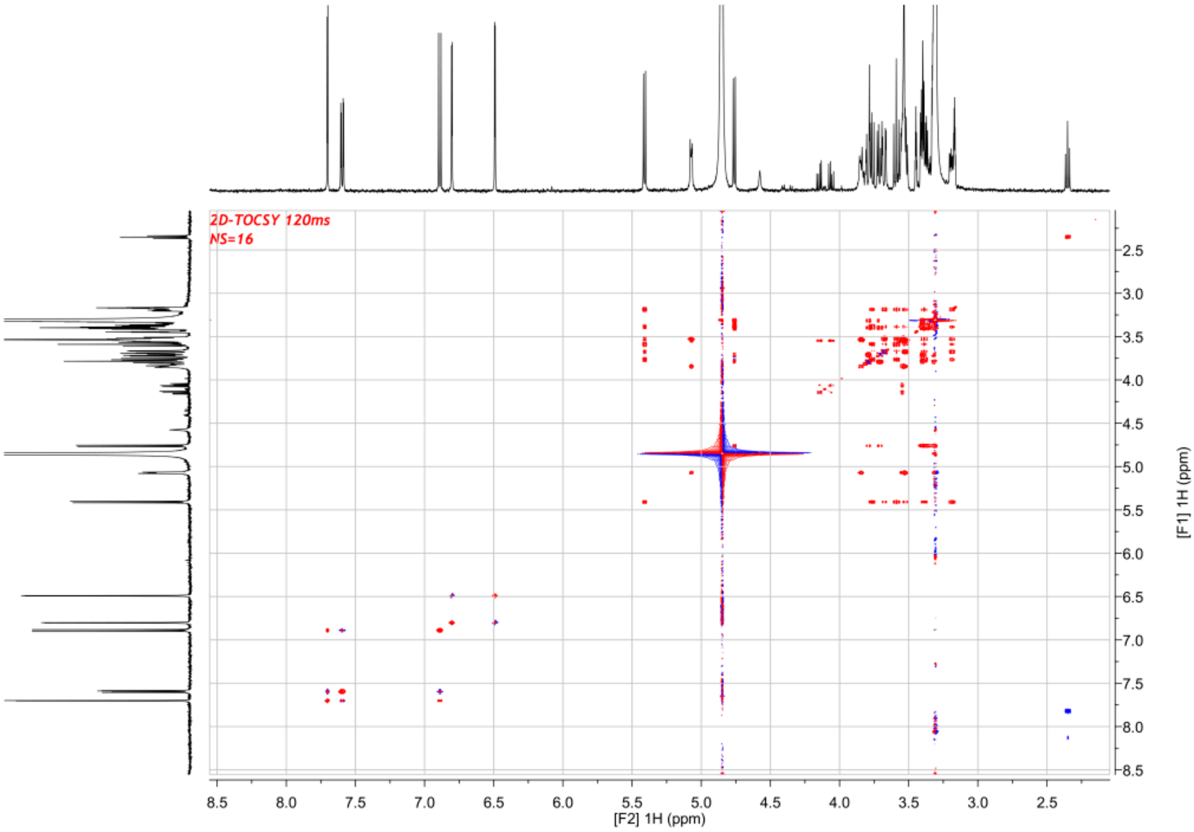

Figure S16. 2D ROESY NMR spectrum of compound **2** (methanol-*d*4, 500.18 MHz).

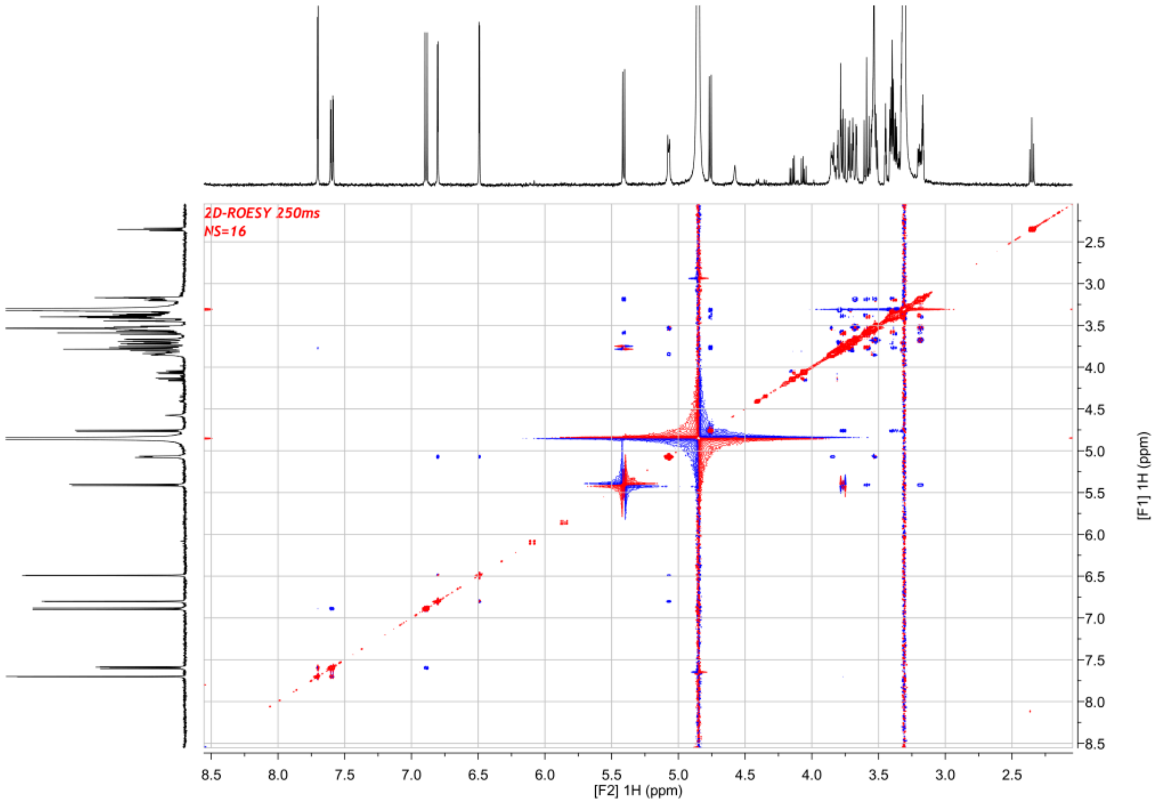

Figure S17. 2D *g*-HSQC-NMR spectrum of compound **2** (methanol-*d*4, 500.18 MHz, 125.77 MHz).

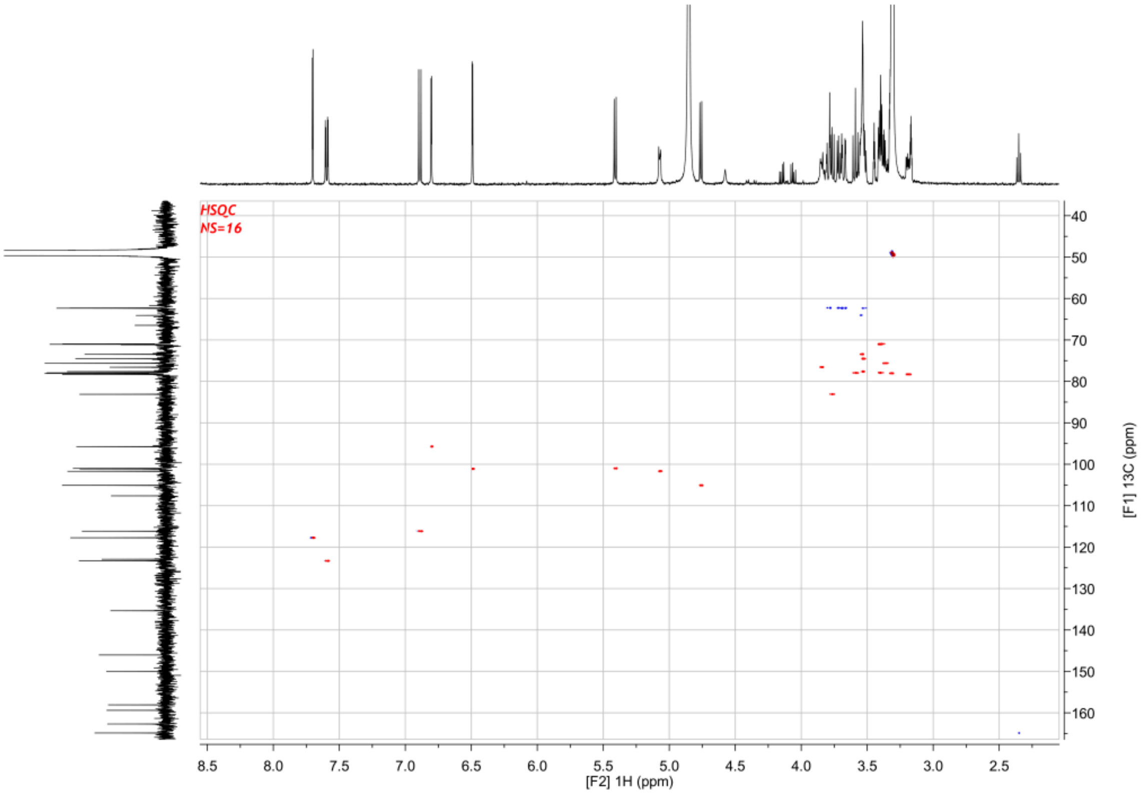

Figure S18. 2D *g*-HSQC-TOCSY NMR spectrum of compound **2** (methanol-*d*4, 500.18 MHz, 125.77 MHz).

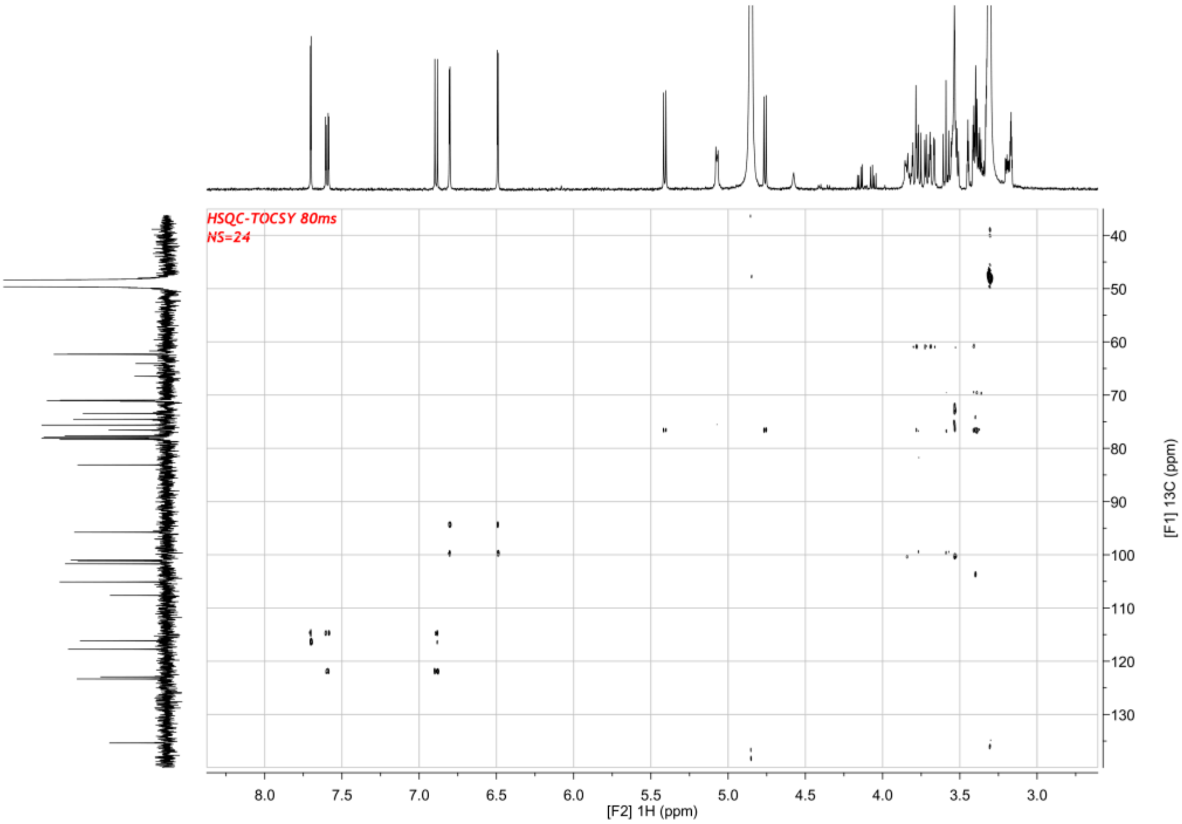

Figure S19. 2D *g*-HMBC-NMR spectrum of compound **2** (methanol-d_4, 500.18 MHz, 125.77 MHz).

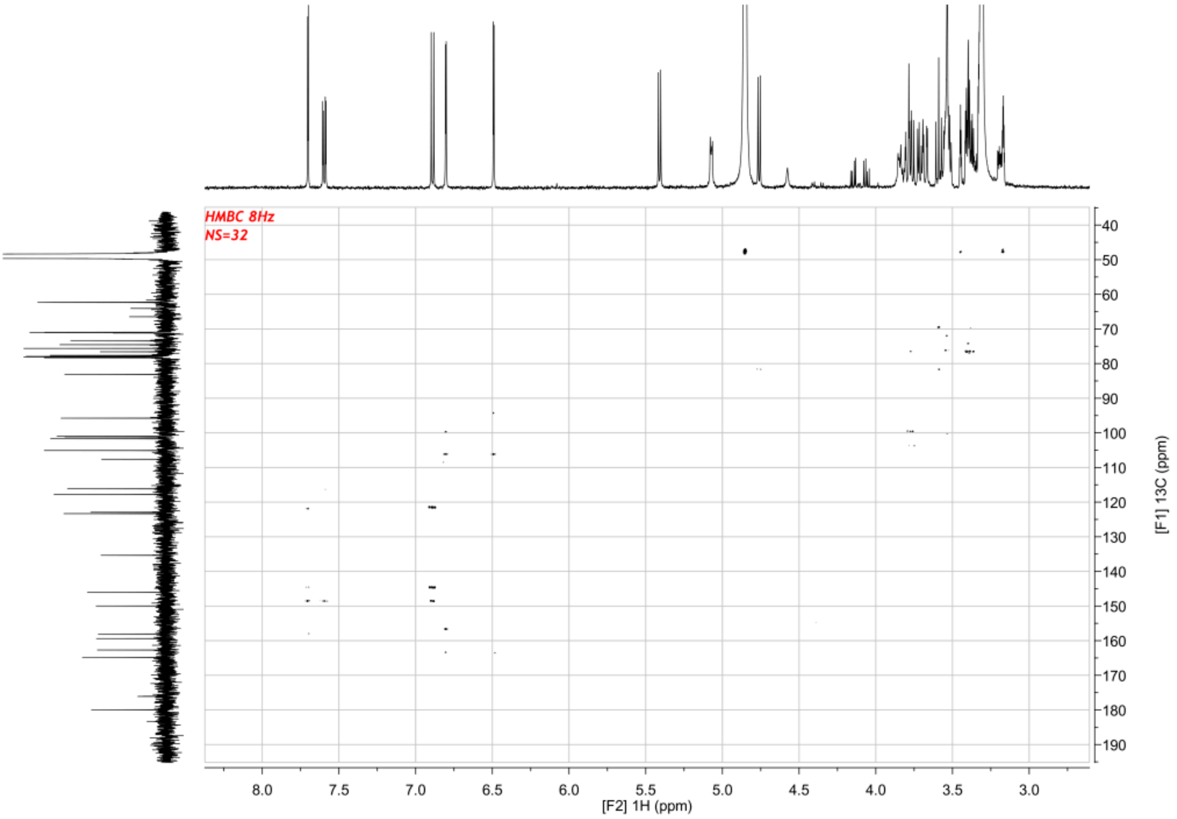

Figure S20. 1D TOCSY and 1D ROESY NMR subspectra of H-1$_{(7\text{-}O\text{-}\beta\text{-GlcA})}$ in compound **2** (methanol-d_4, 500.18 MHz).

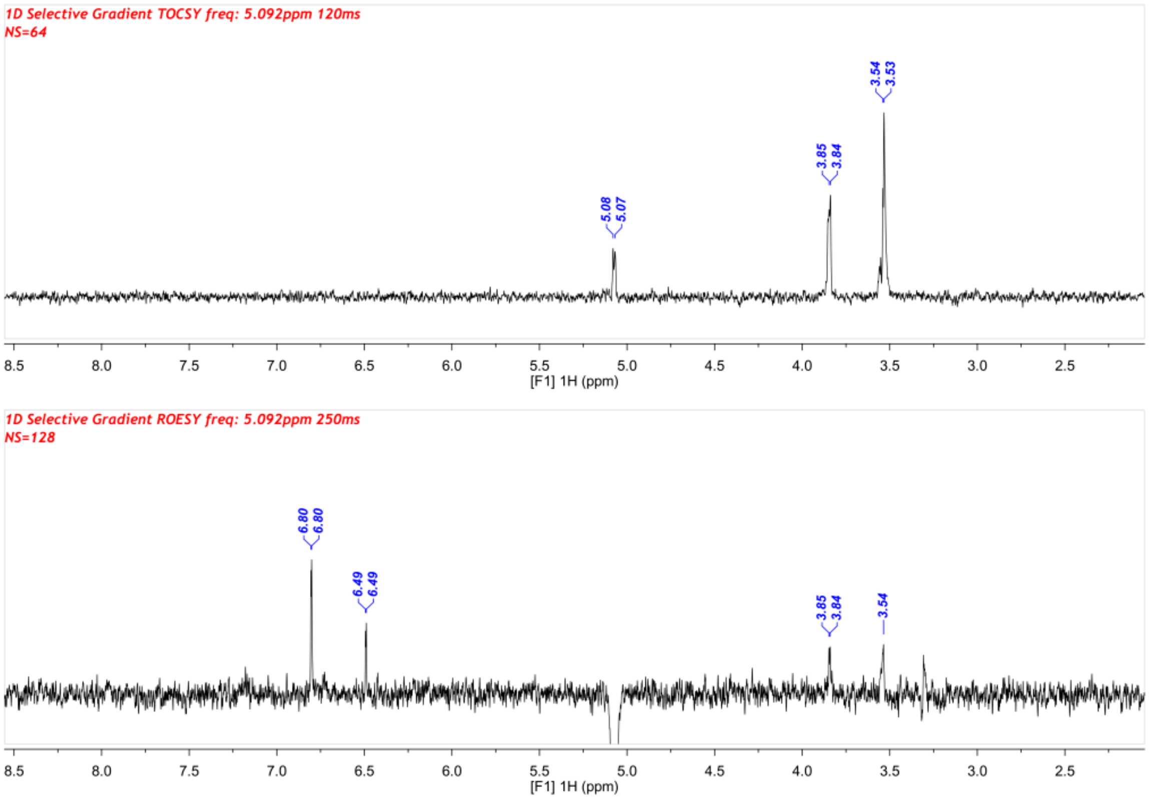

Figure S21. 1D TOCSY and 1D ROESY NMR subspectra of H-1(3-*O*-β-Glc) in compound **2** (methanol-d_4, 500.18 MHz).

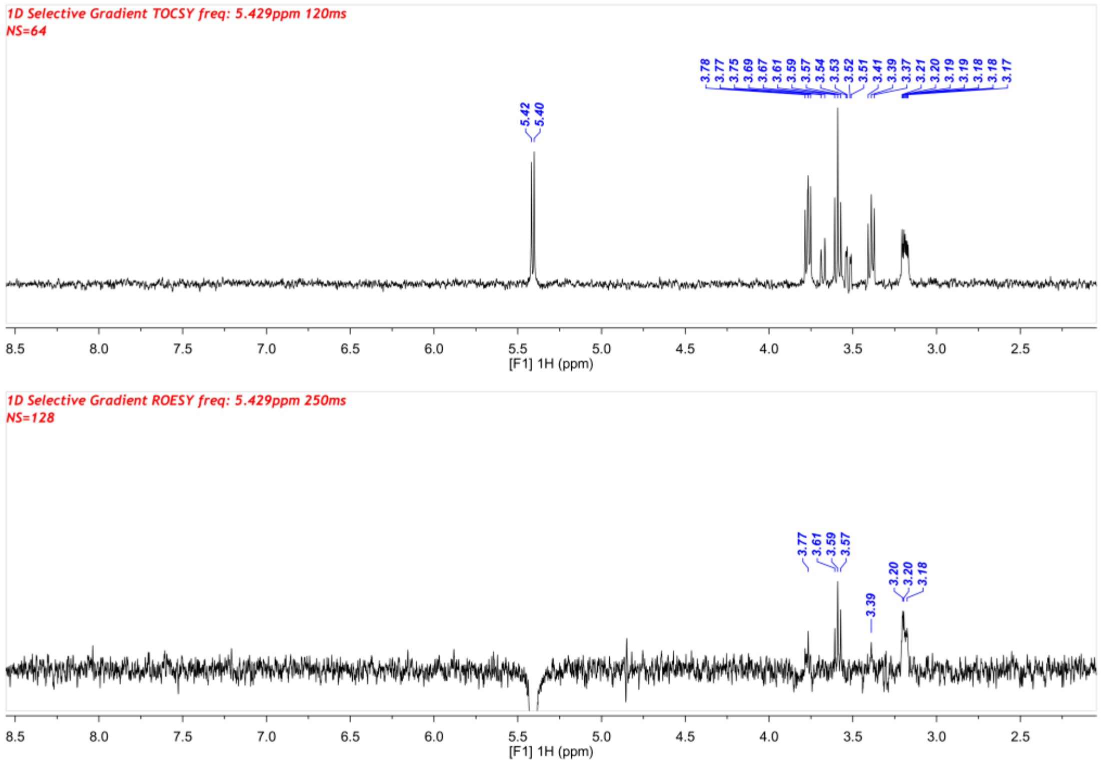

Figure S22. 1D TOCSY and 1D ROESY NMR subspectra of H-1(2^{Glc}-*O*-β-Glc) in compound **2** (methanol-d_4, 500.18 MHz).

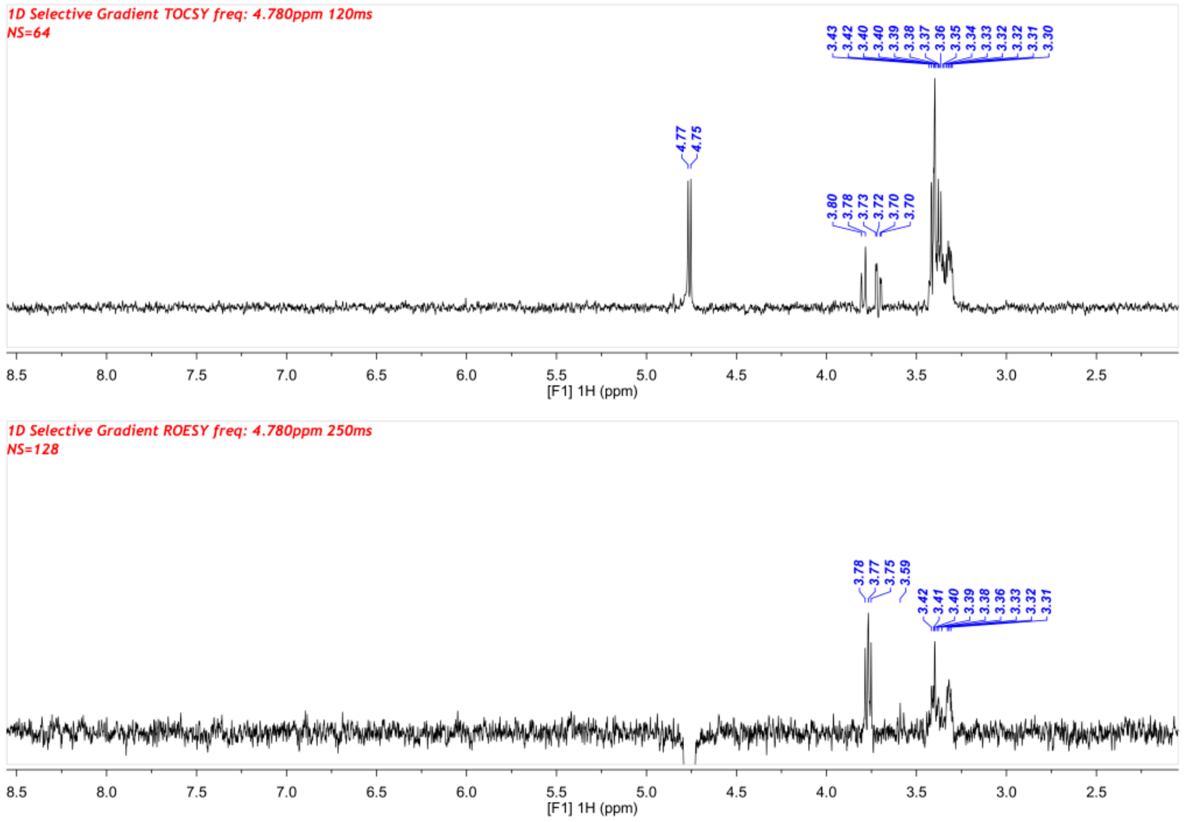

Compound 3

Figure S23. 1D ^{1}H-NMR spectrum of compound **3** (methanol-d_4, 500.18 MHz).

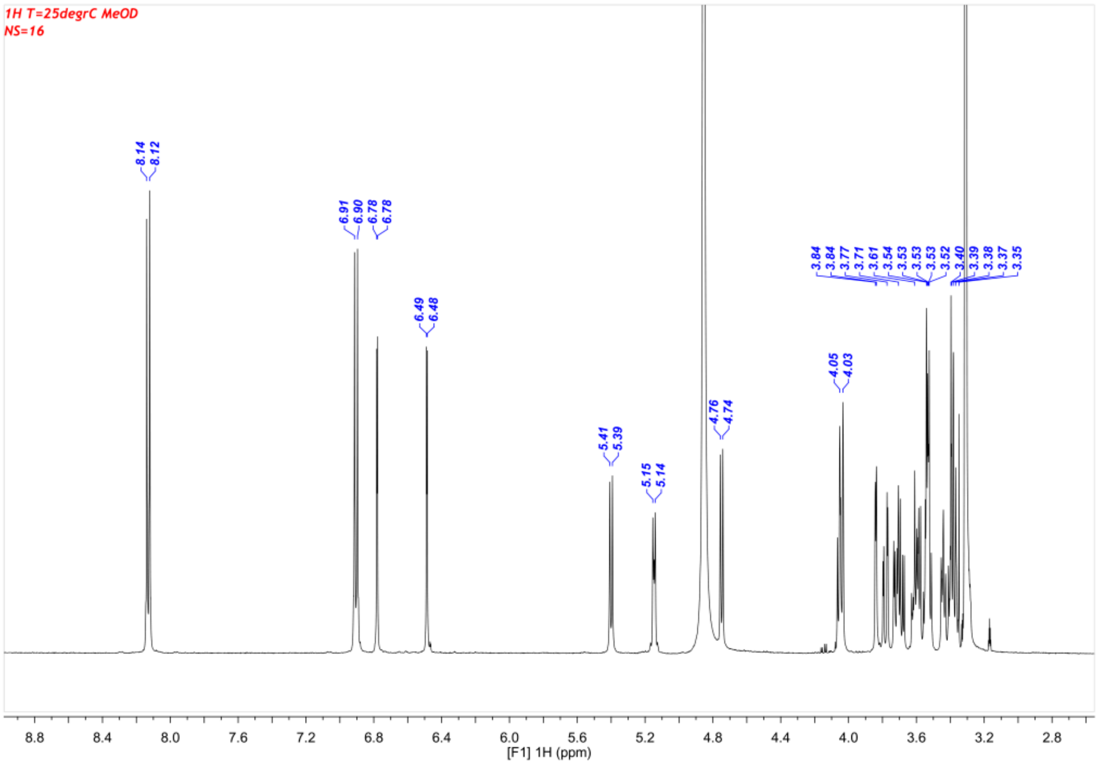

Figure S24. 1D ^{13}C-NMR spectra (1H decoupled and DEPT-135) of compound **3** (methanol-d_4, 125.77 MHz).

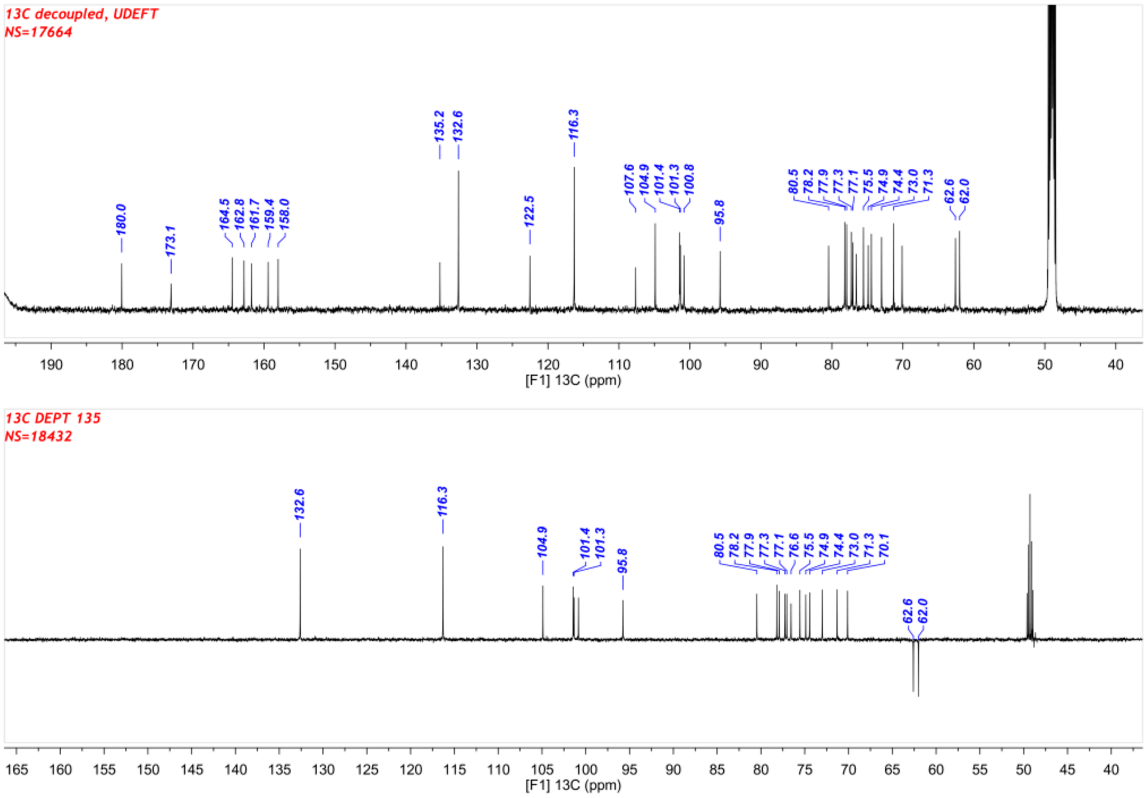

Figure S25. 2D *g*-COSY NMR spectrum of compound **3** (methanol-d_4, 500.18 MHz).

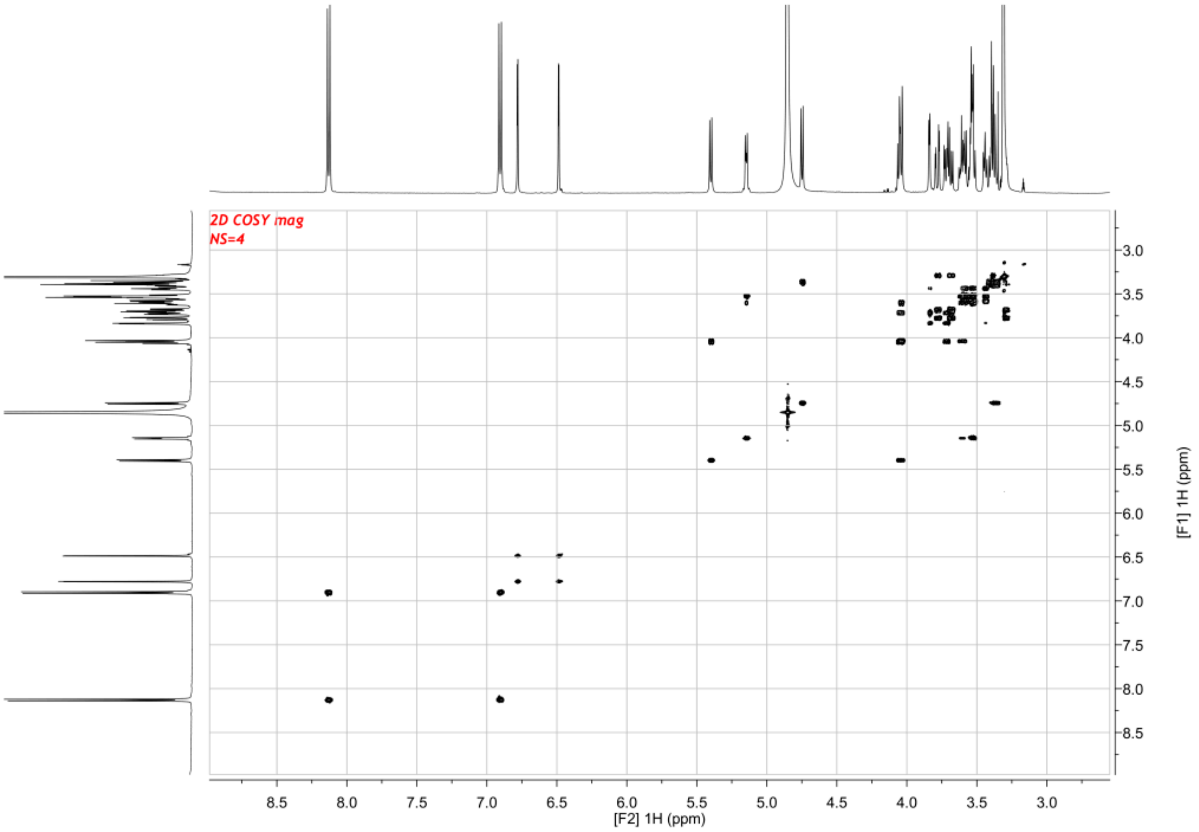

Figure S26. 2D TOCSY NMR spectrum of compound **3** (methanol-d_4, 500.18 MHz).

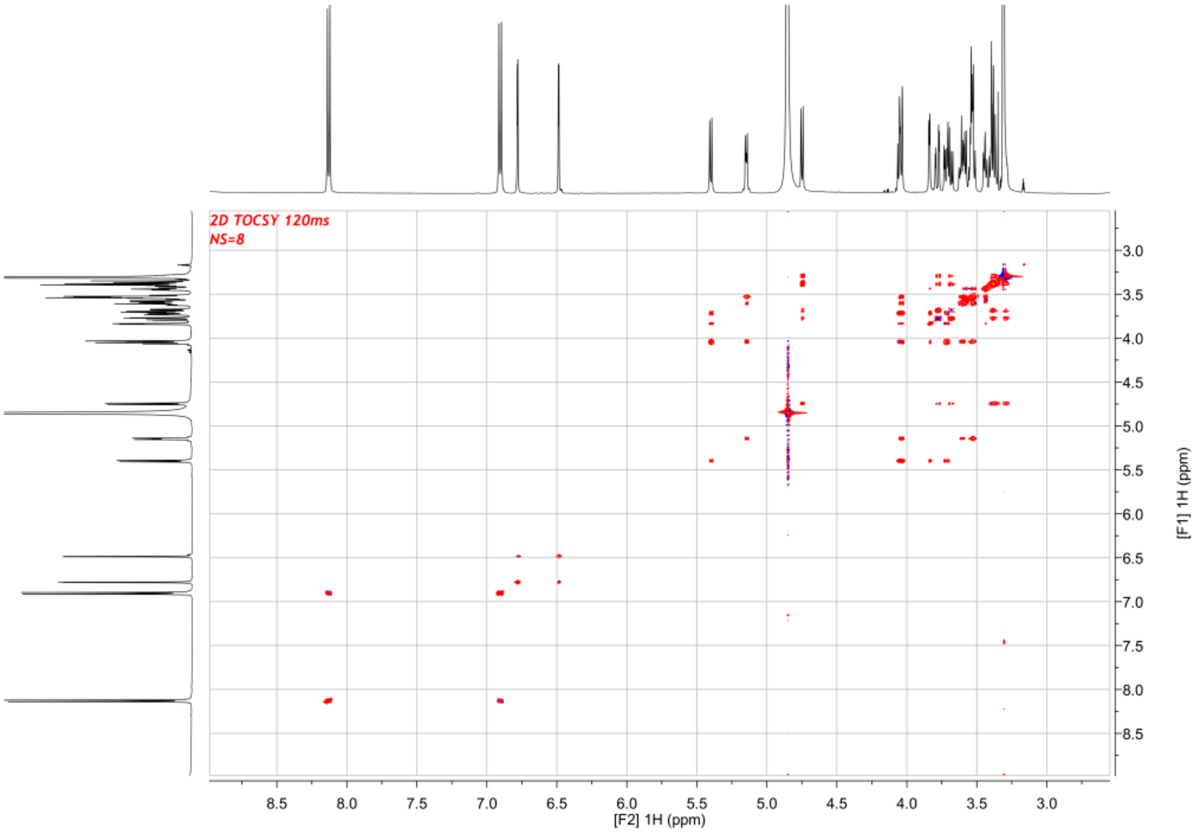

Figure S27. 2D ROESY NMR spectrum of compound **3** (methanol-*d*4, 500.18 MHz).

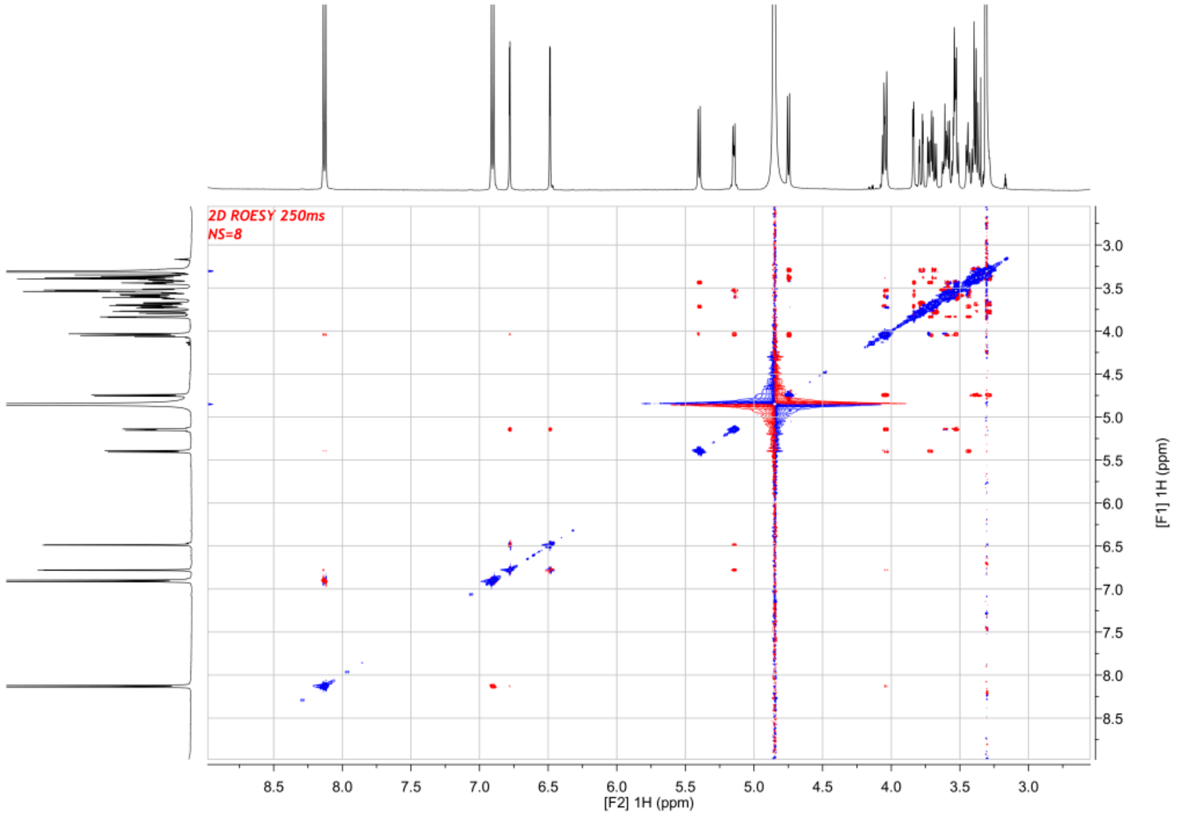

Figure S28. 2D *g*-HSQC-NMR spectrum of compound **3** (methanol-*d*4, 500.18 MHz, 125.77 MHz).

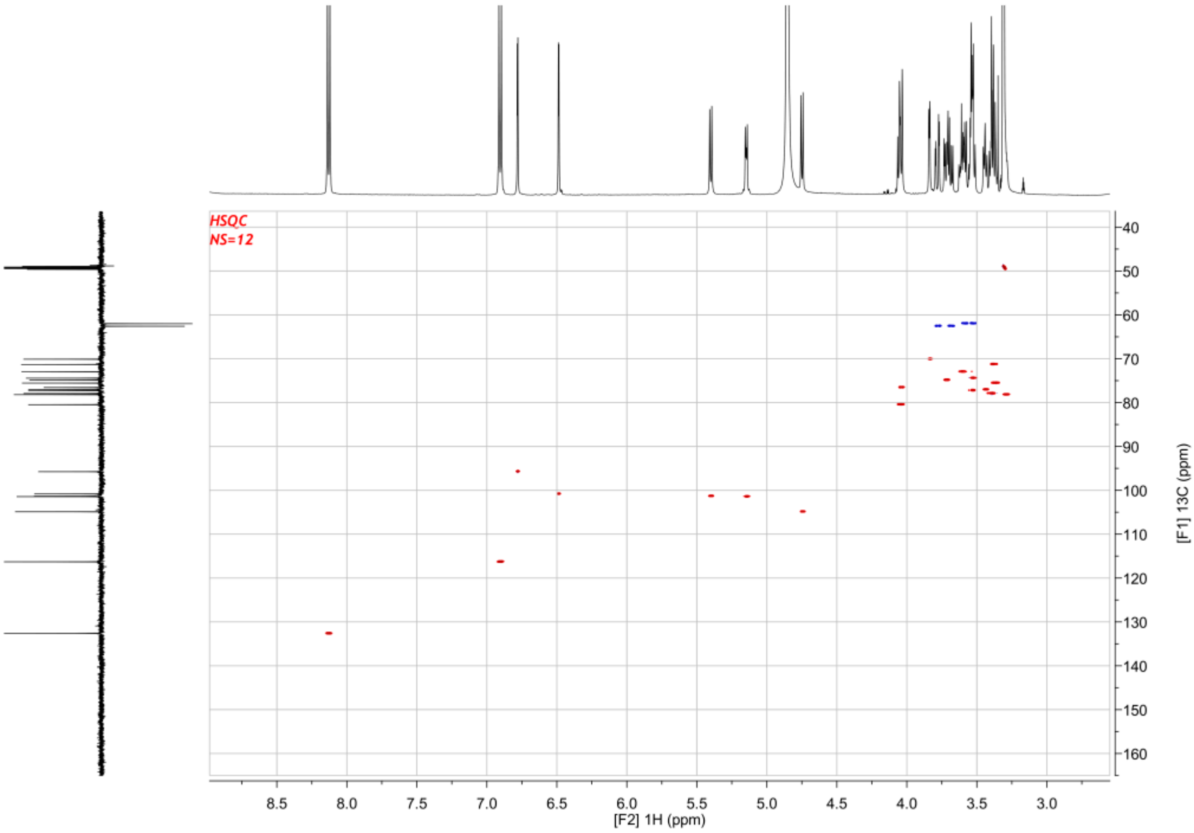

Figure S29. 2D *g*-HSQC-TOCSY NMR spectrum of compound **3** (methanol-*d*4, 500.18 MHz, 125.77 MHz).

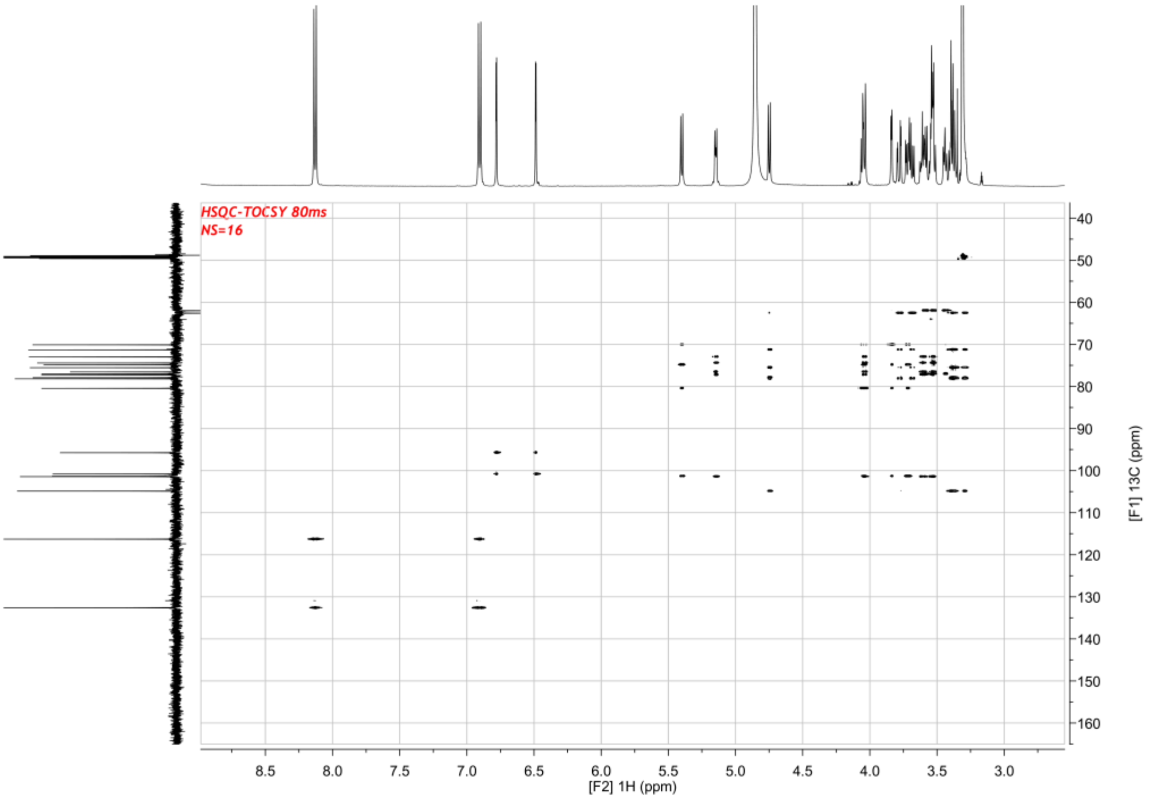

Figure S30. 2D *g*-HMBC-NMR spectrum of compound **3** (methanol-*d*4, 500.18 MHz, 125.77 MHz).

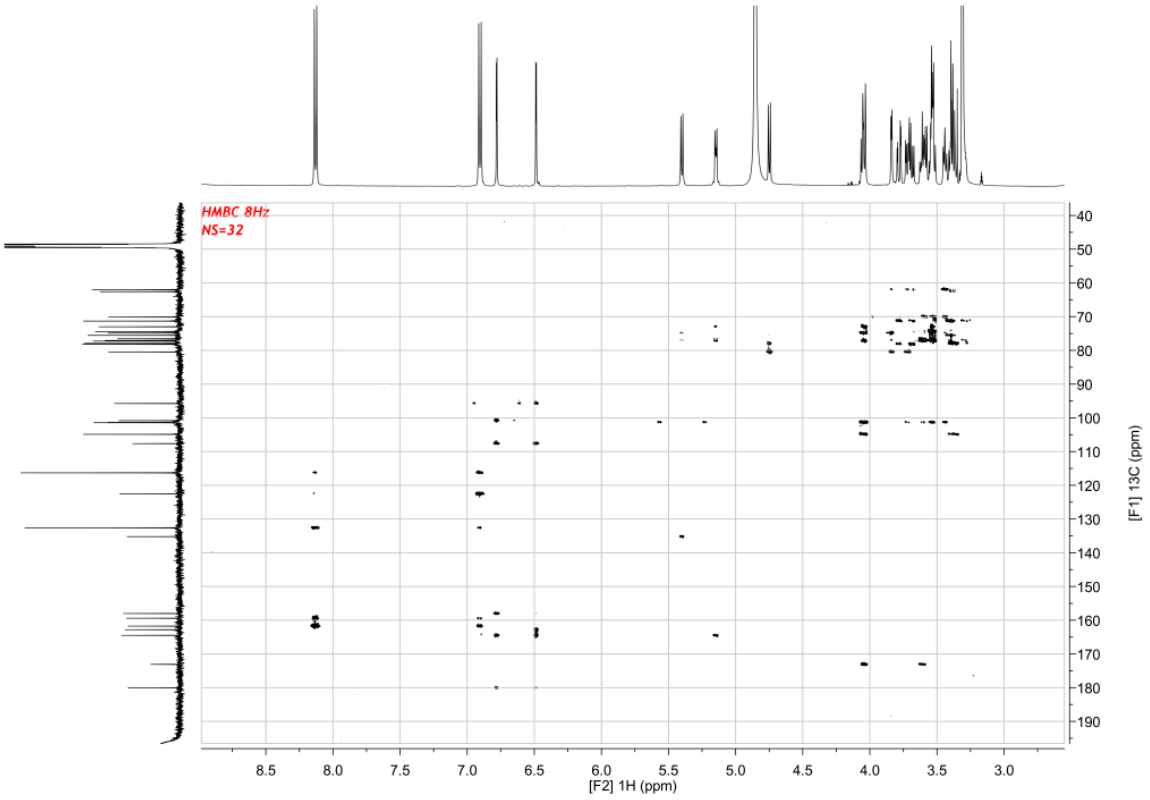

Figure S31. 1D TOCSY and 1D ROESY NMR subspectra of H-1(7-*O*-β-GlcA) in compound **3** (methanol-d_4, 500.18 MHz).

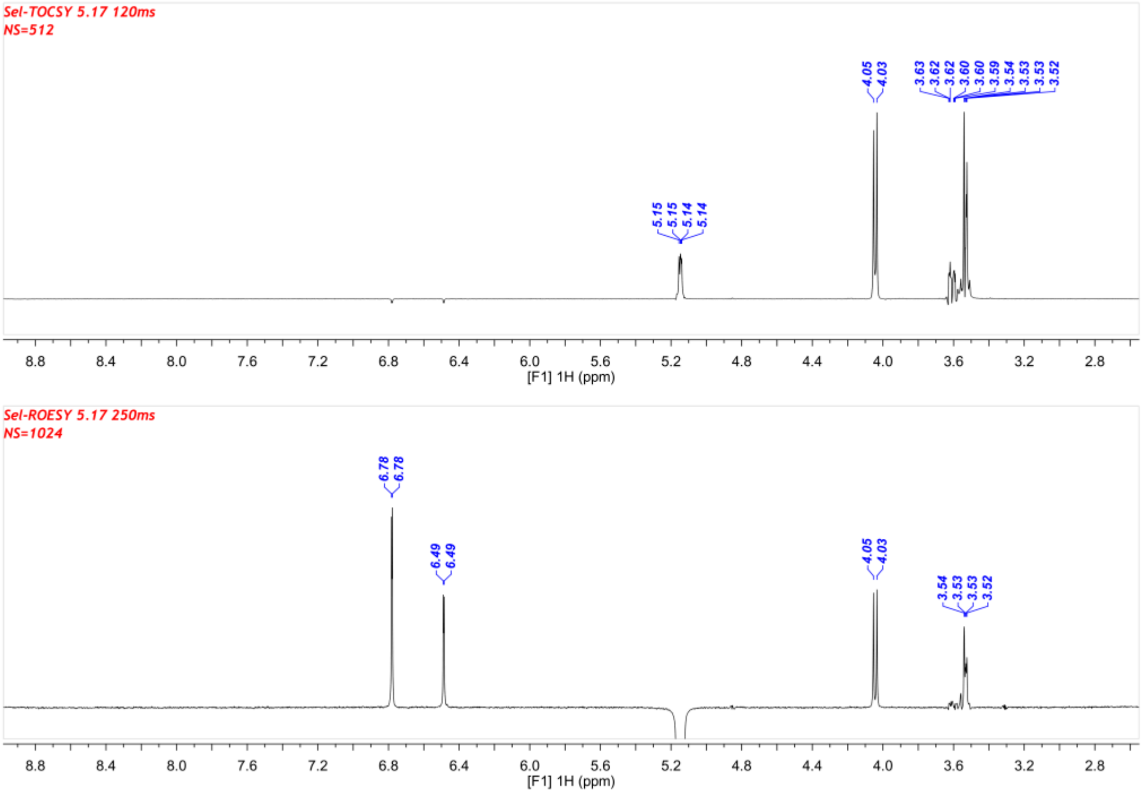

Figure S32. 1D TOCSY and 1D ROESY NMR subspectra of H-1(3-*O*-β-Gal) in compound **3** (methanol-d_4, 500.18 MHz).

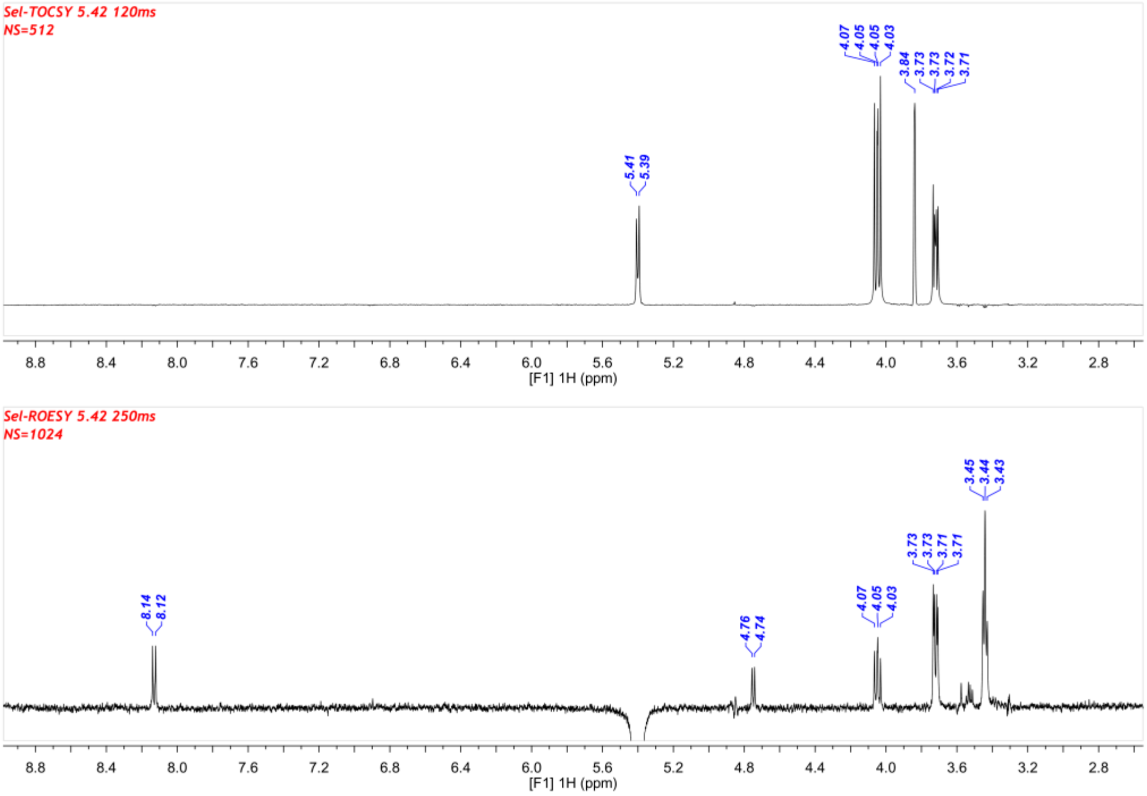

Figure S33. 1D TOCSY and 1D ROESY NMR subspectra of H-1$_{(2^{Gal}\text{-}O\text{-}\beta\text{-}Glc)}$ in compound **3** (methanol-*d*4, 500.18 MHz).

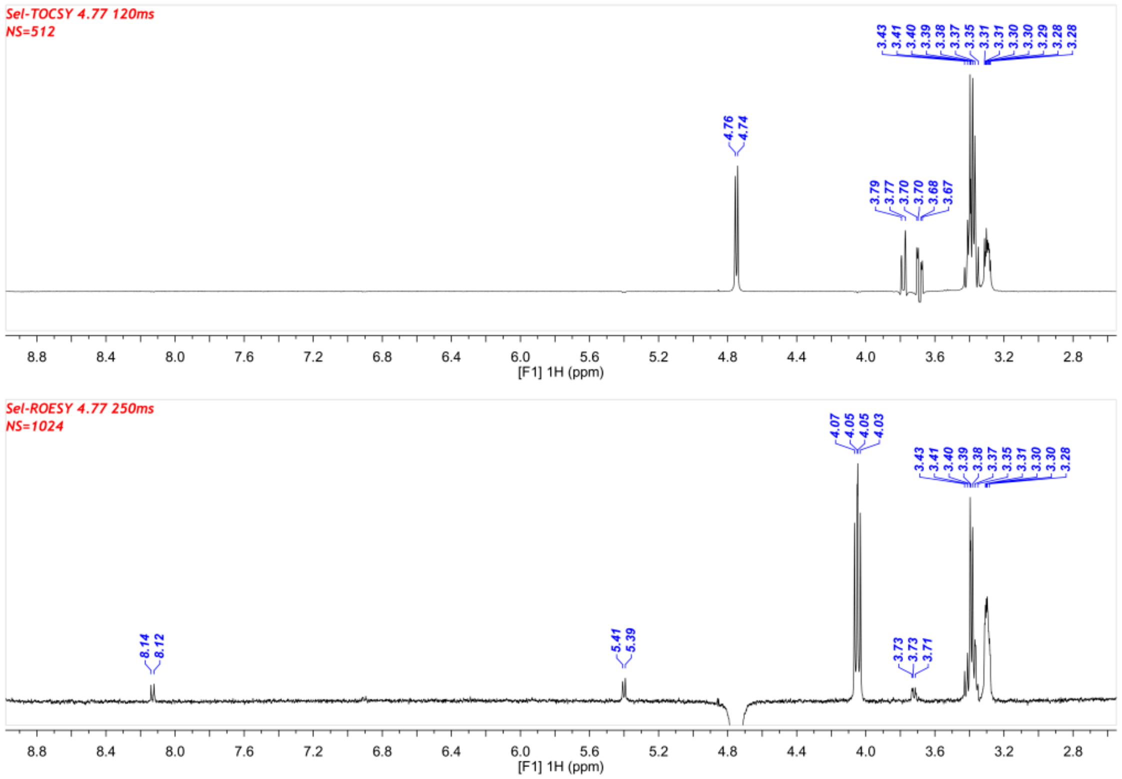

Compound 4

Figure S34. 1D ^{1}H-NMR spectrum of compound **4** (methanol-*d*4, 500.18 MHz).

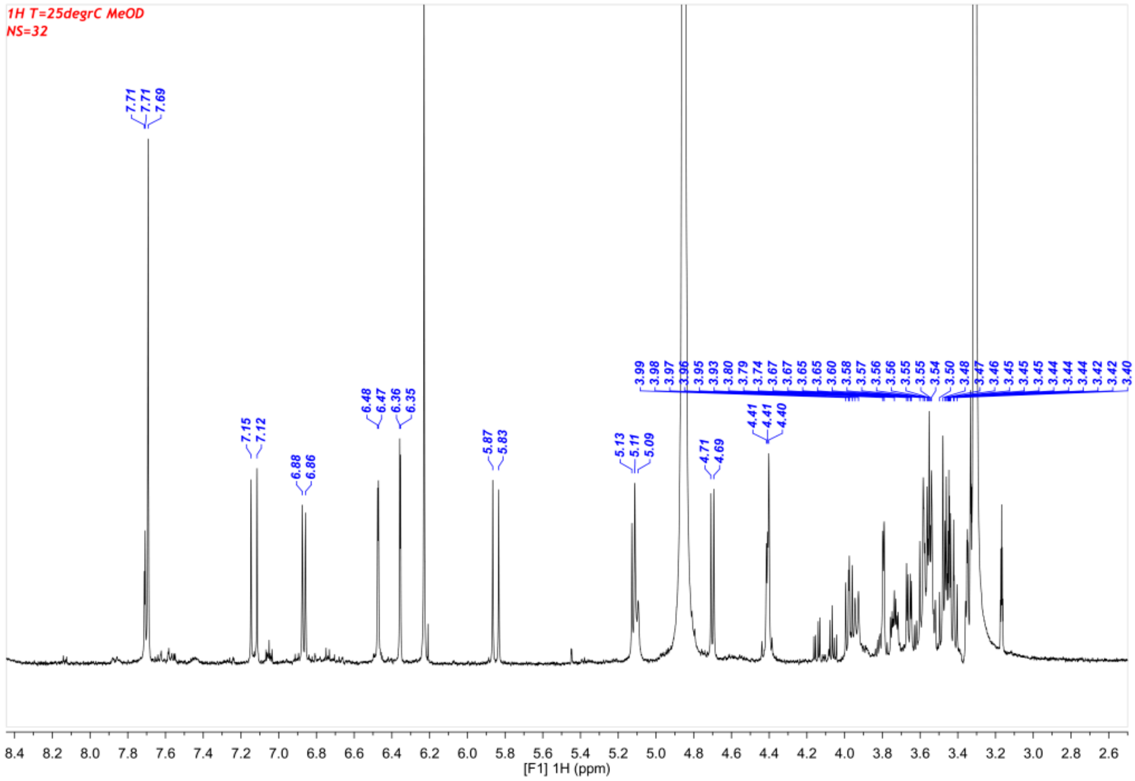

Figure S35. 1D ^{13}C-NMR spectra (1H decoupled and DEPT-135) of compound **4** (methanol-*d*$_4$, 125.77 MHz).

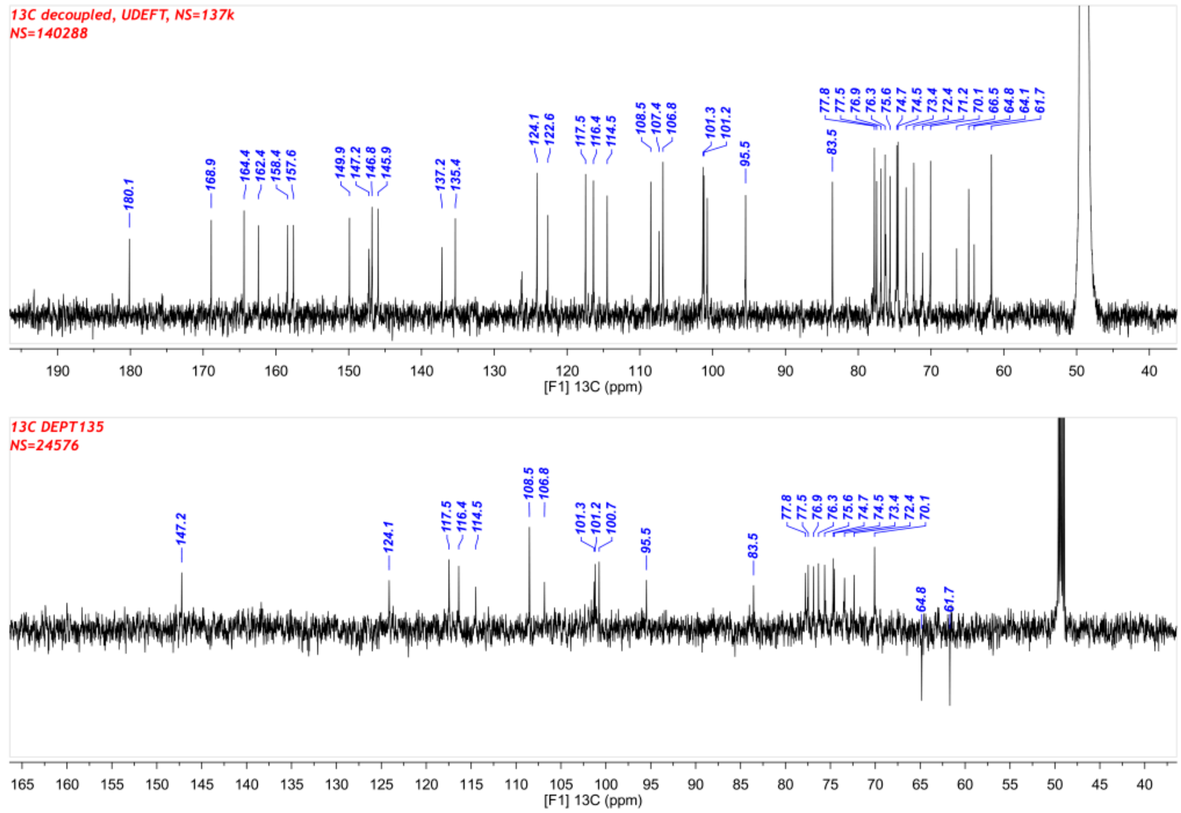

Figure S36. 2D *g*-COSY NMR spectrum of compound **4** (methanol-*d*$_4$, 500.18 MHz).

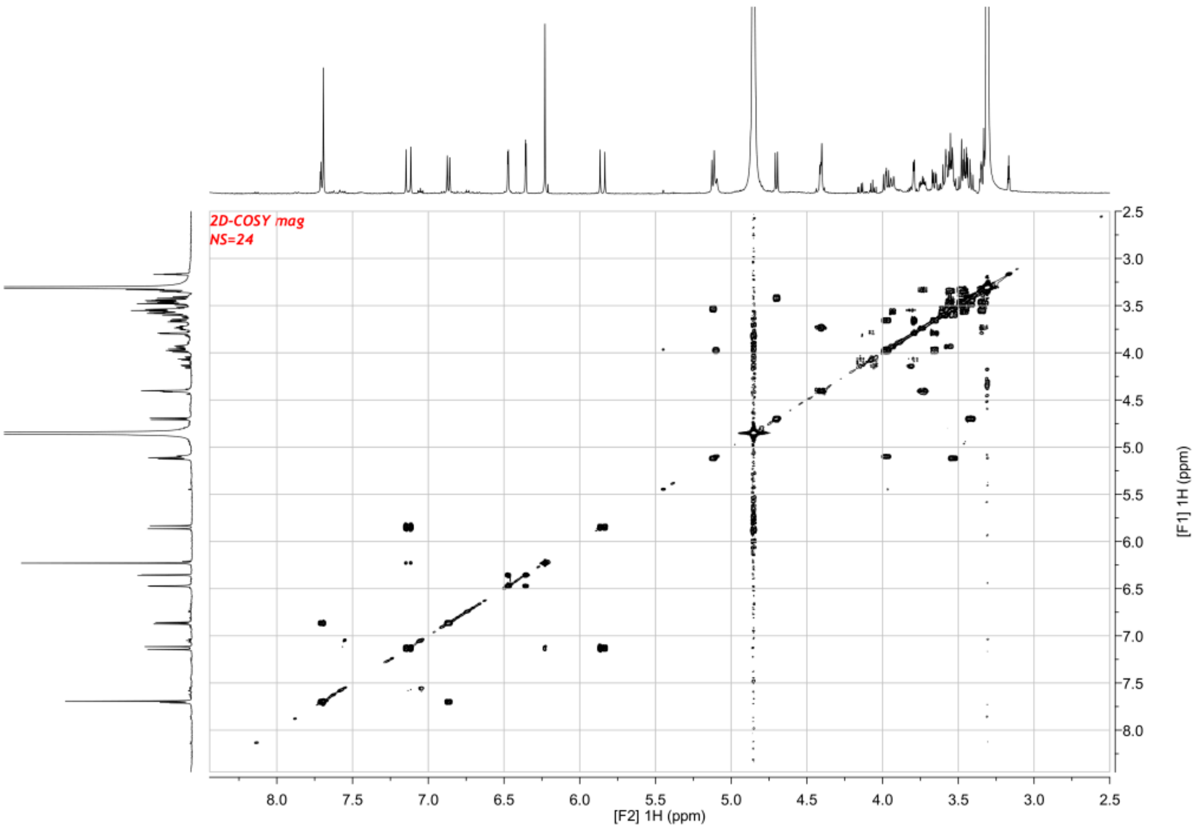

Figure S37. 2D TOCSY NMR spectrum of compound **4** (methanol-*d*4, 500.18 MHz).

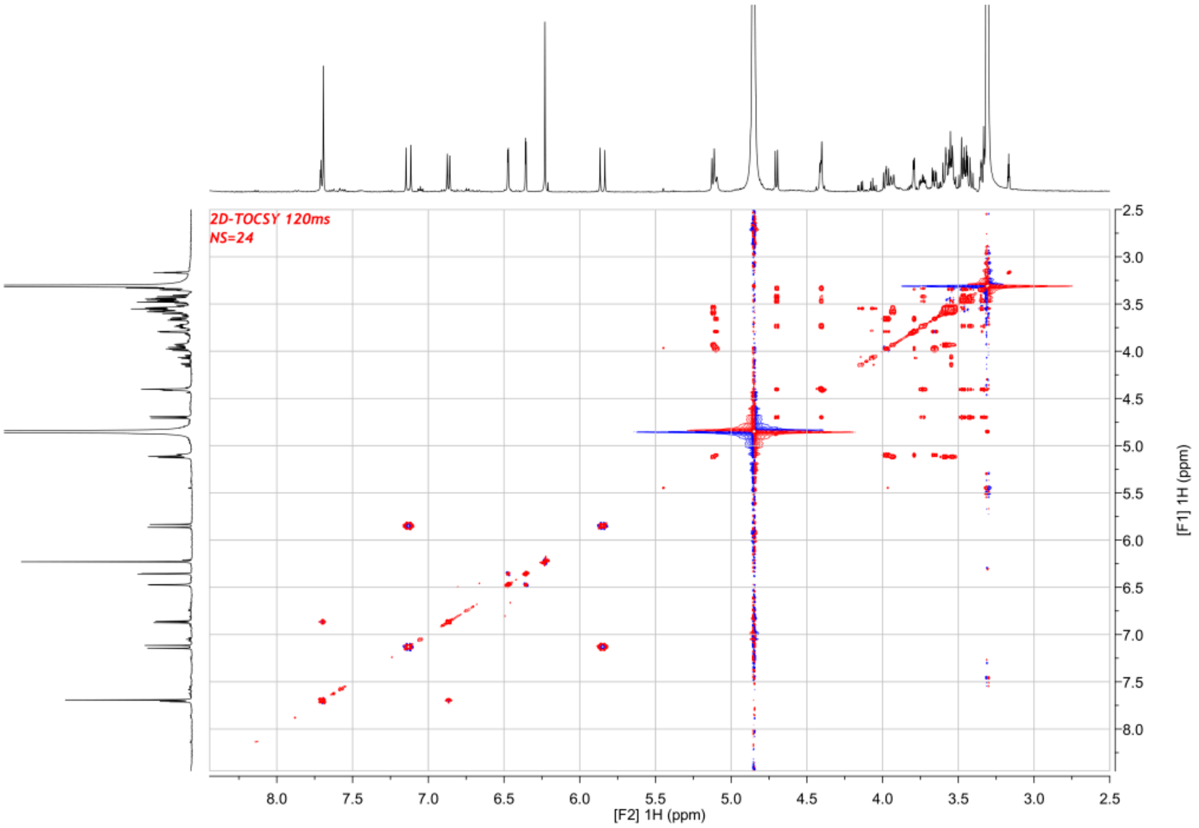

Figure S38. 2D ROESY NMR spectrum of compound **4** (methanol-*d*4, 500.18 MHz).

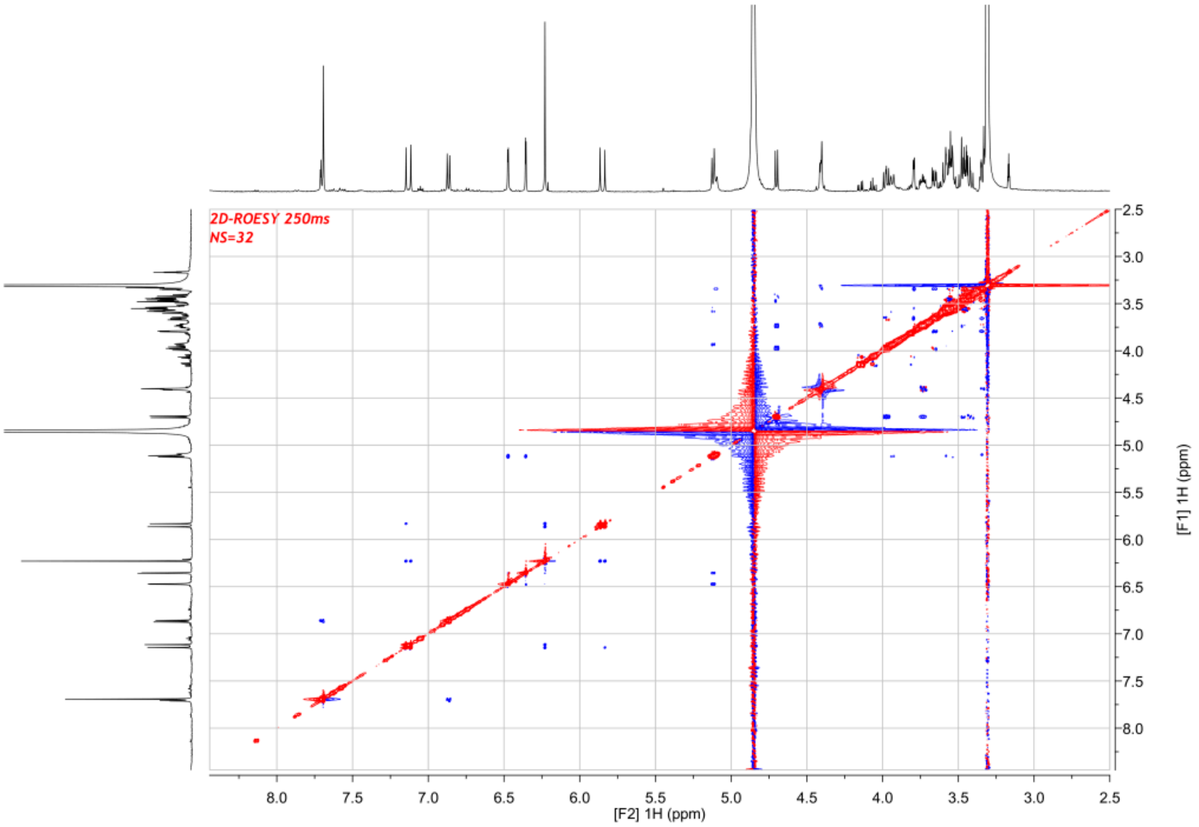

Figure S39. 2D *g*-HSQC-NMR spectrum of compound **4** (methanol-*d*4, 500.18 MHz, 125.77 MHz).

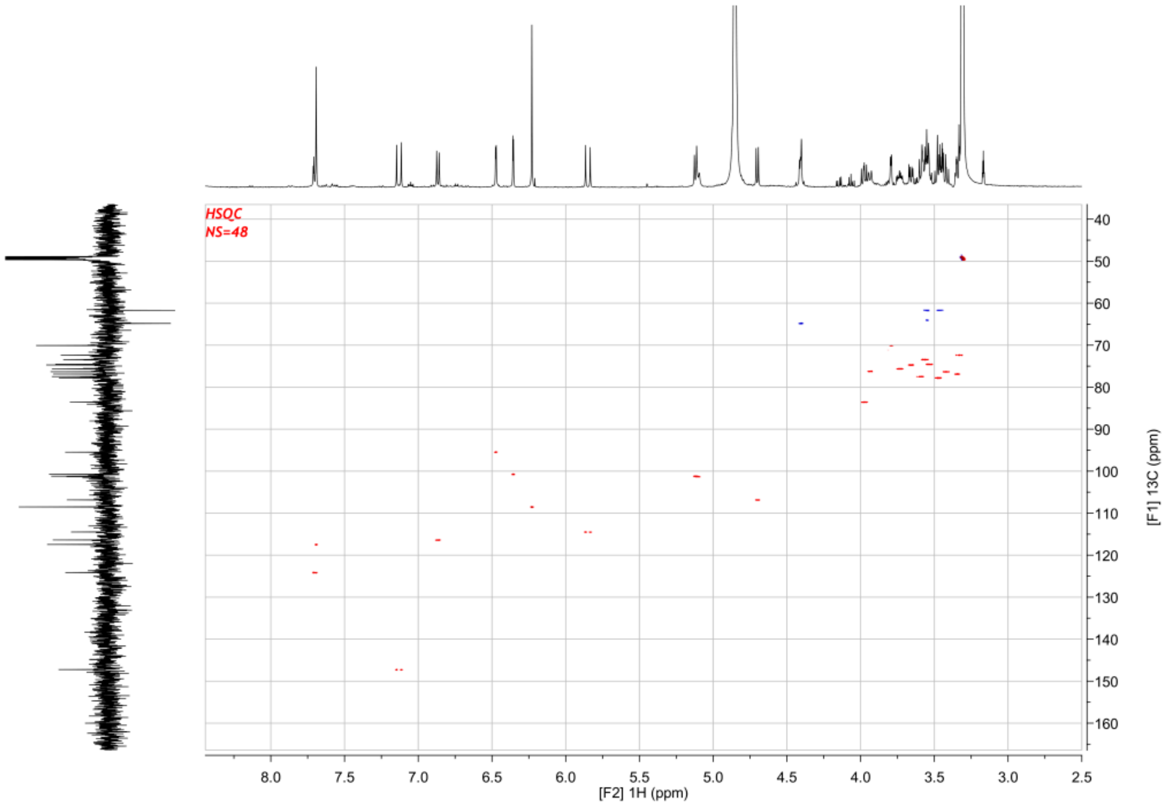

Figure S40. 2D *g*-HSQC-TOCSY NMR spectrum of compound **4** (methanol-*d*4, 500.18 MHz, 125.77 MHz).

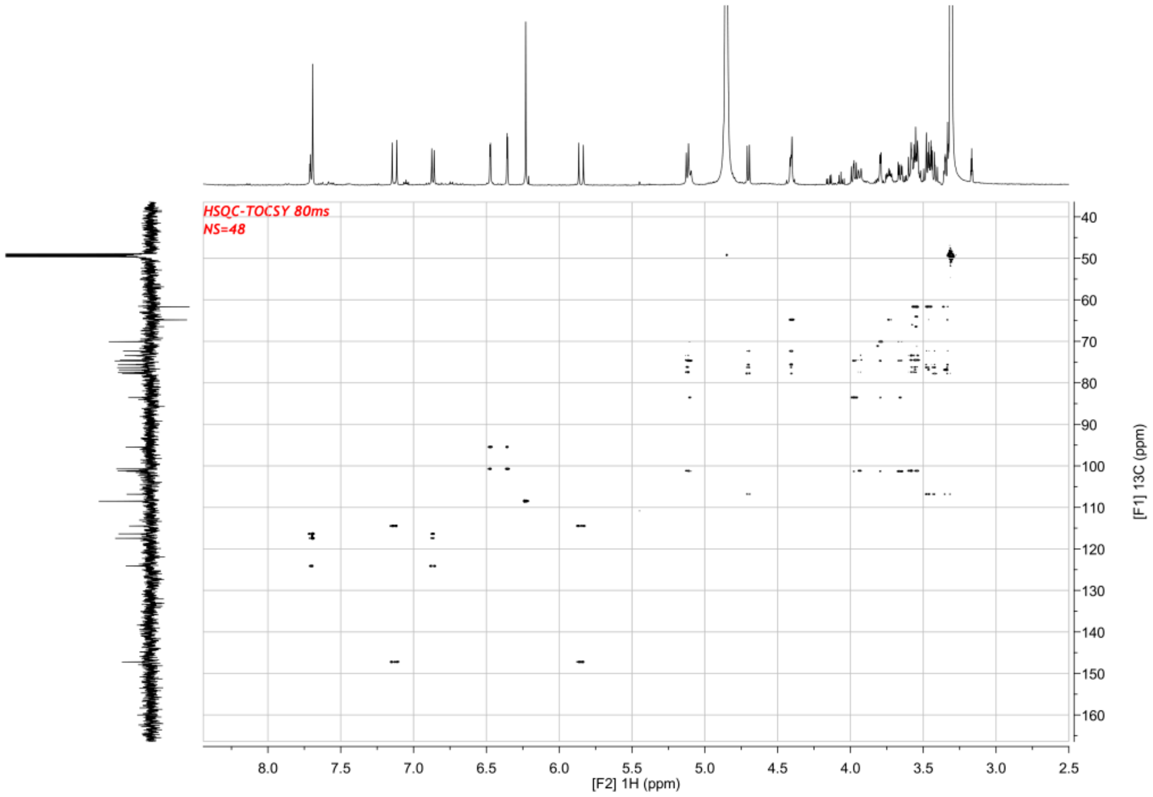

Figure S41. 2D *g*-HMBC-NMR spectrum of compound **4** (methanol-*d*4, 500.18 MHz, 125.77 MHz).

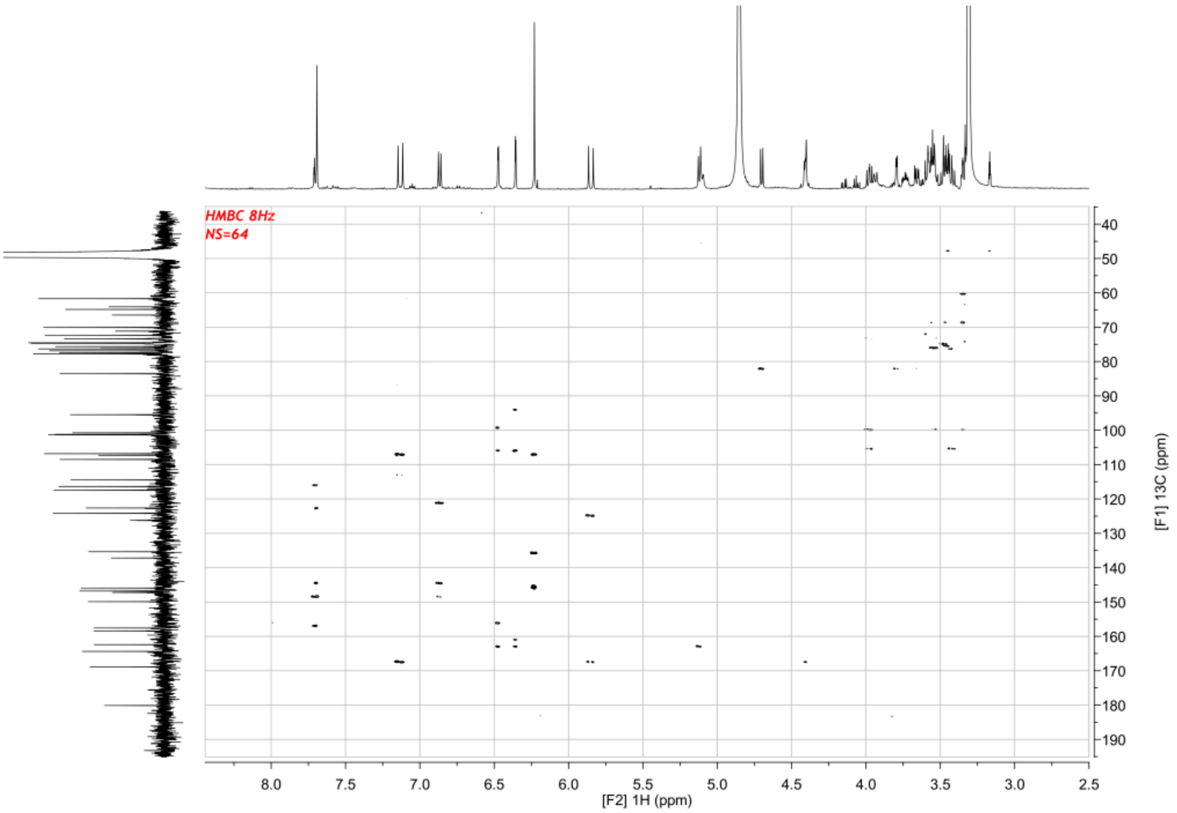

Figure S42. 1D TOCSY and 1D ROESY NMR subspectra of H-1(7-*O*-β-GlcA) and H-1(3-*O*-β-Gal) in compound **4** (methanol-*d*4, 500.18 MHz).

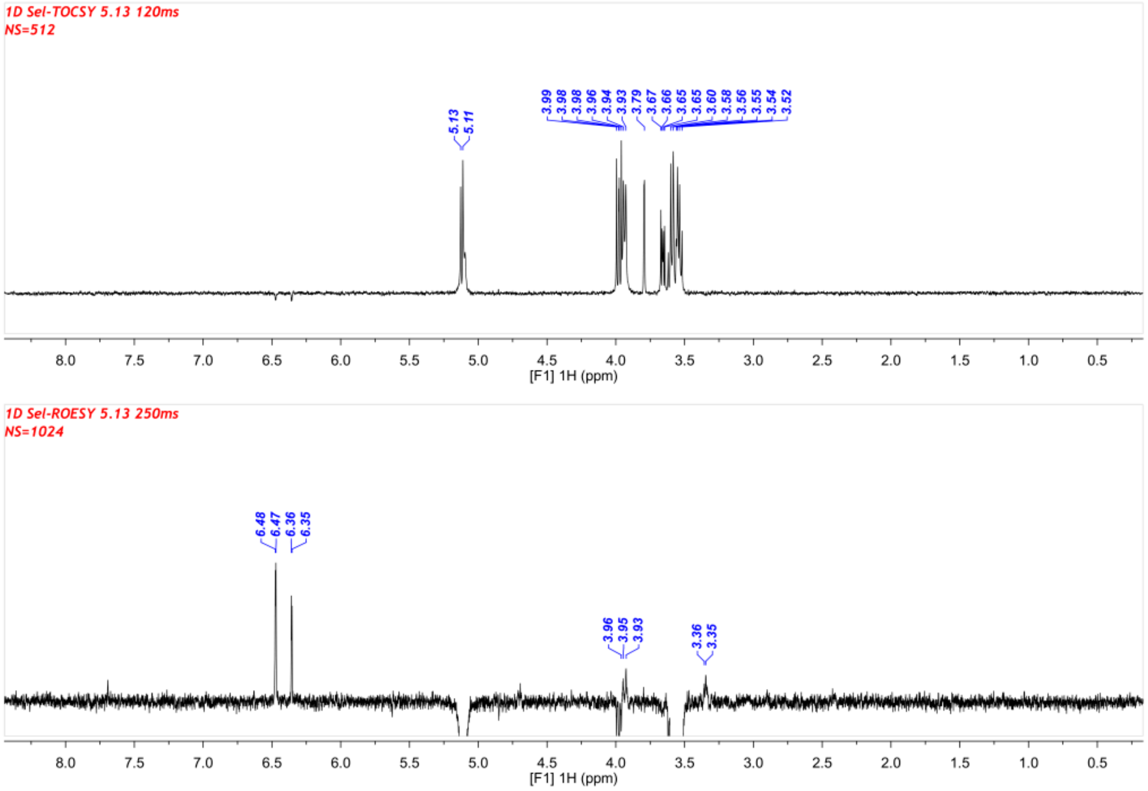

Figure S43. 1D TOCSY and 1D ROESY NMR subspectra of H-1$_{(2^{Gal}\text{-}O\text{-}\beta\text{-}Glc)}$ in compound **4** (methanol-d_4, 500.18 MHz).

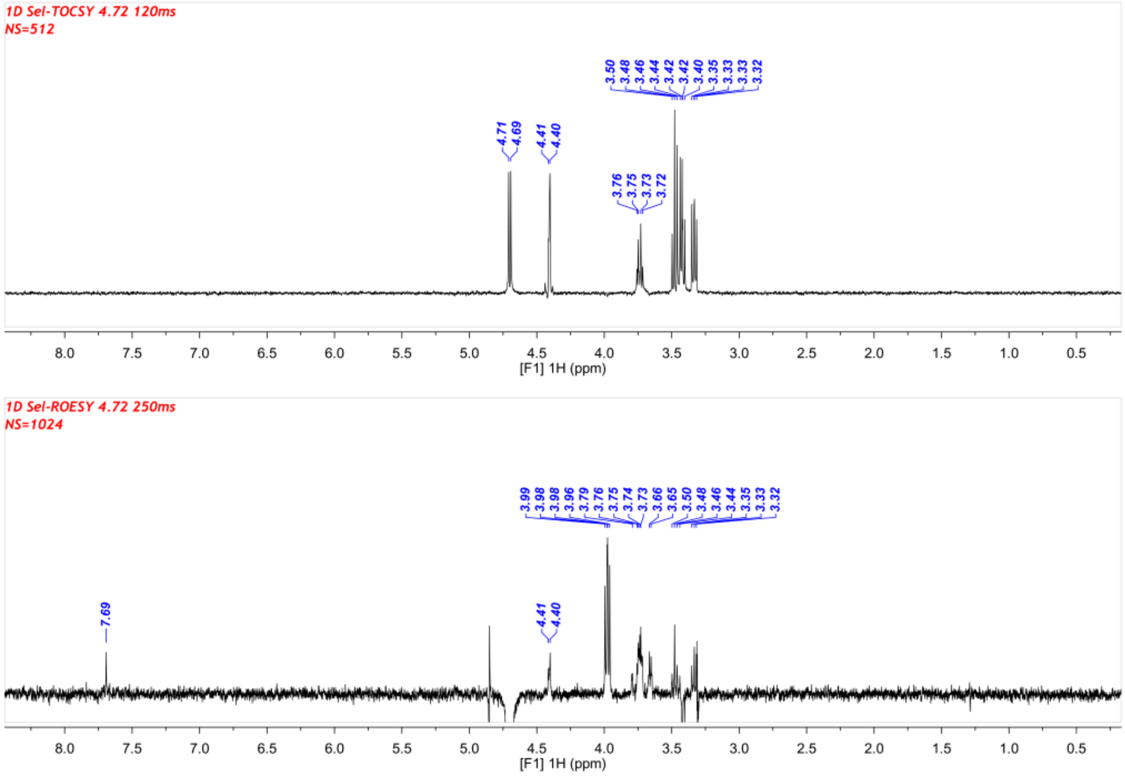

Compound 5

Figure S44. 1D ^{1}H-NMR spectrum of compound **5** (methanol-d_4, 500.18 MHz).

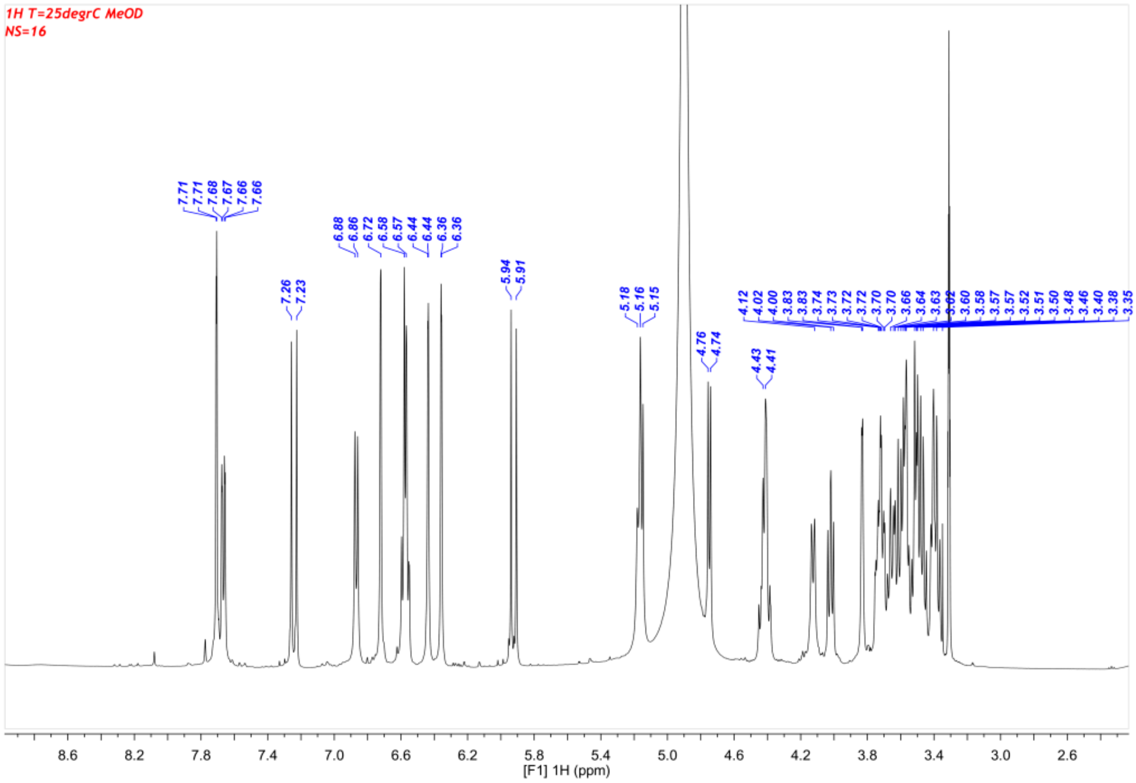

Figure S45. 1D ^{13}C-NMR spectra (1H decoupled and DEPT-135) of compound **5** (methanol-d_4, 125.77 MHz).

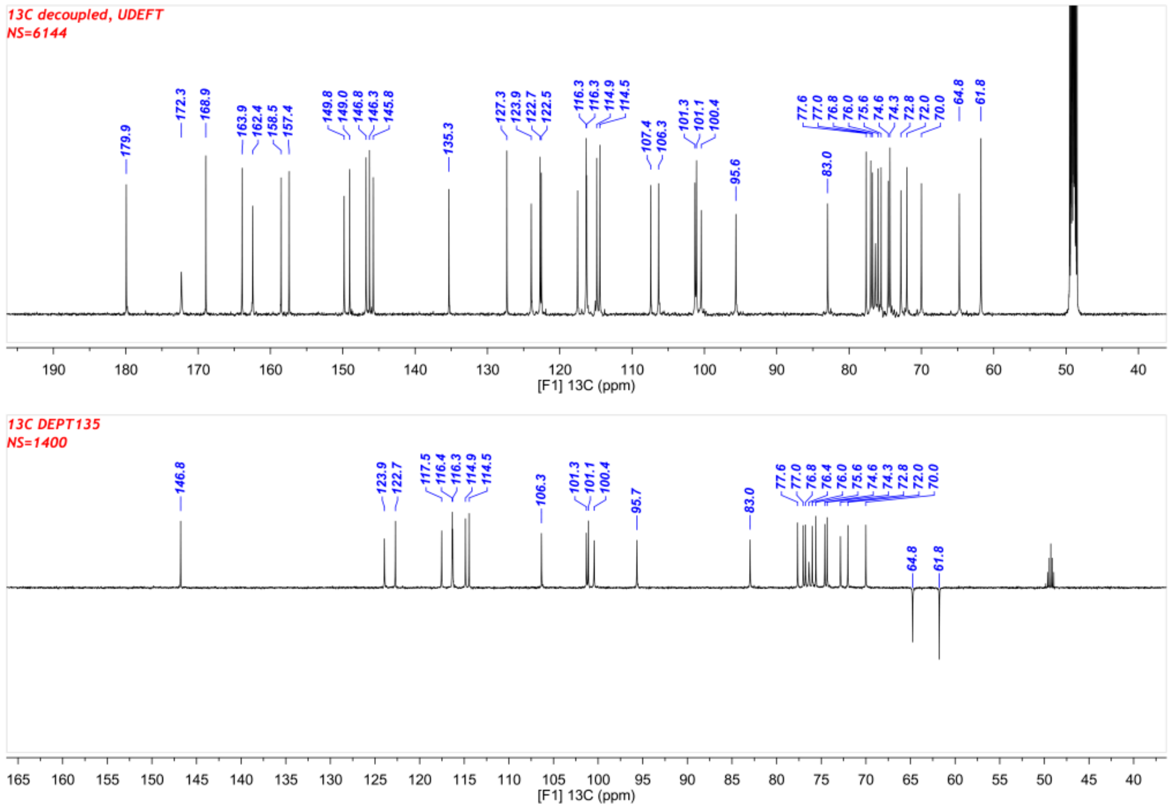

Figure S46. 2D *g*-COSY NMR spectrum of compound **5** (methanol-d_4, 500.18 MHz).

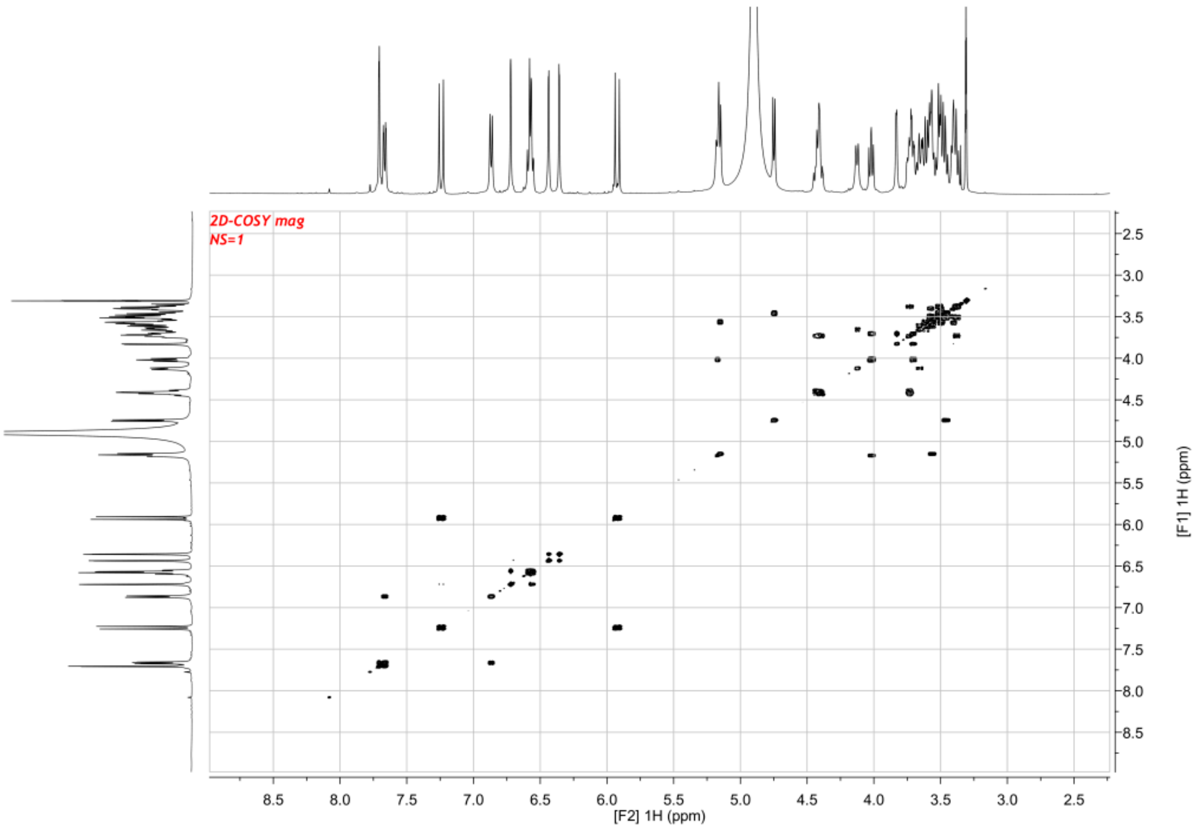

Figure S47. 2D TOCSY NMR spectrum of compound **5** (methanol-d_4, 500.18 MHz).

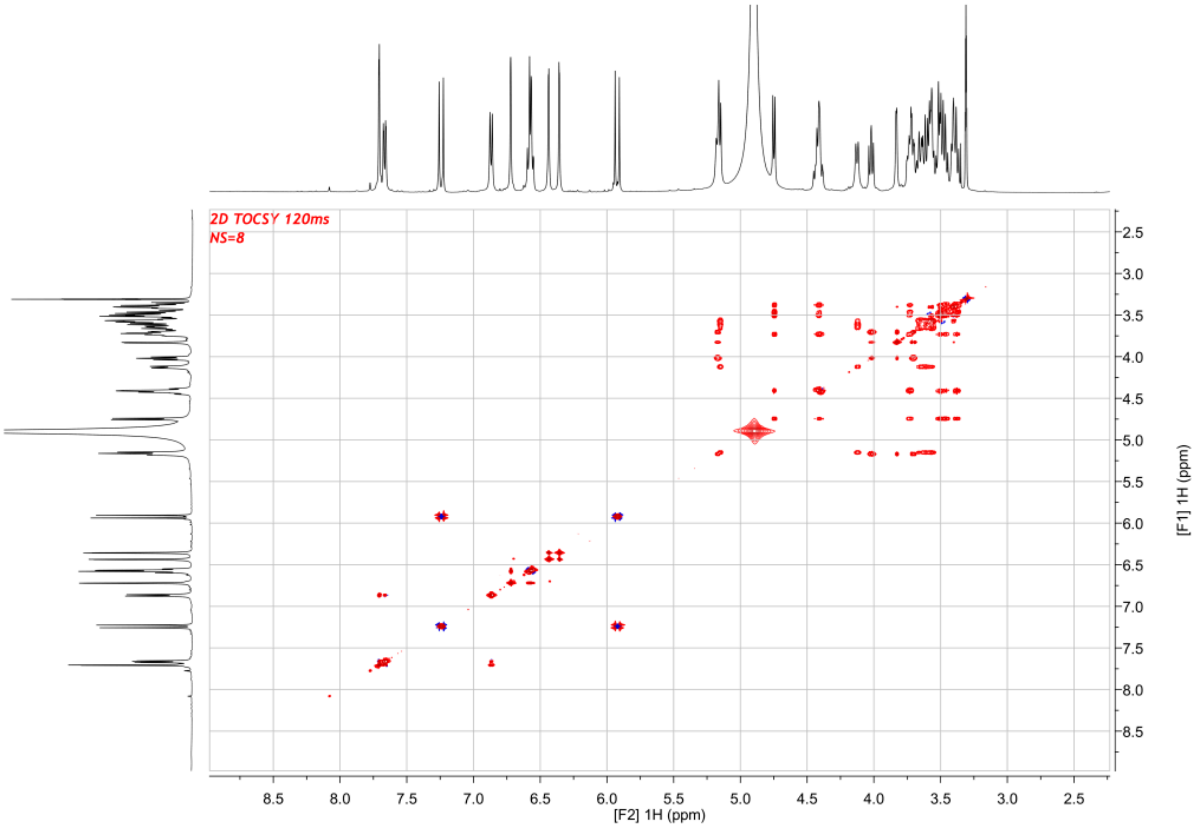

Figure S48. 2D ROESY NMR spectrum of compound **5** (methanol-d_4, 500.18 MHz).

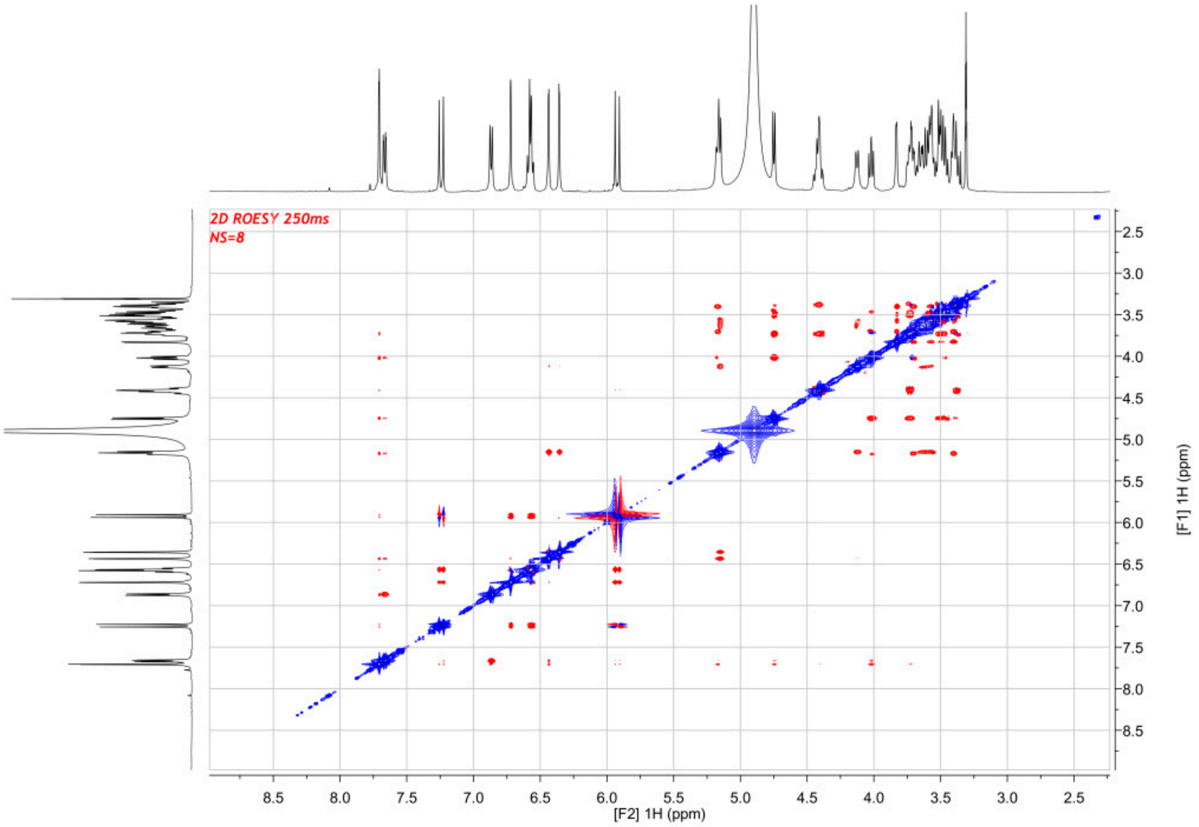

Figure S49. 2D *g*-HSQC-NMR spectrum of compound **5** (methanol-*d*4, 500.18 MHz, 125.77 MHz).

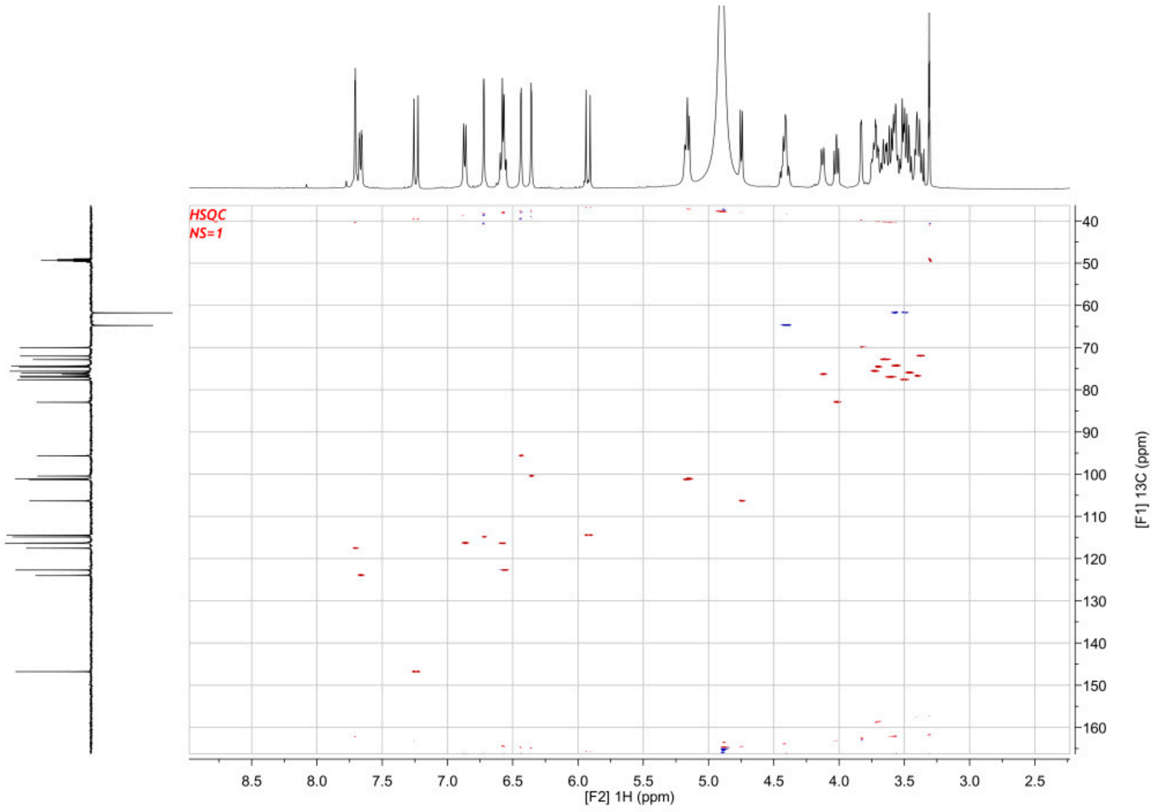

Figure S50. 2D *g*-HSQC-TOCSY NMR spectrum of compound **5** (methanol-*d*4, 500.18 MHz, 125.77 MHz).

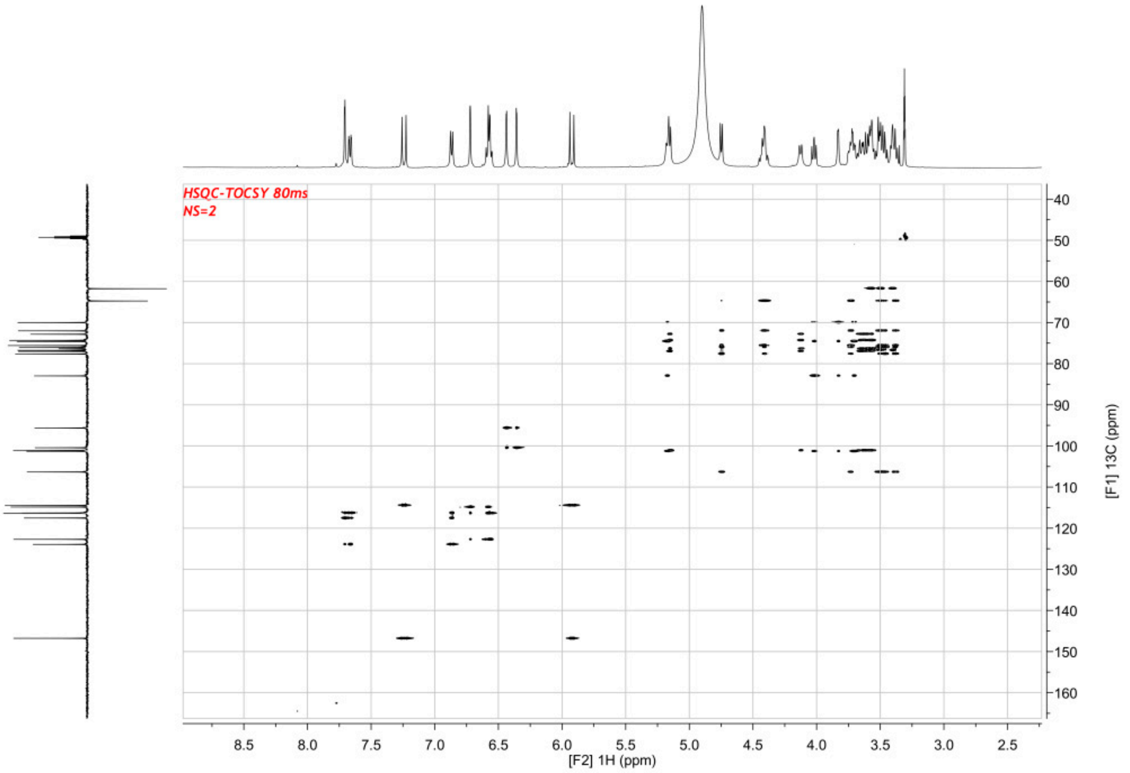

Figure S51. 2D *g*-HMBC-NMR spectrum of compound **5** (methanol-d_4, 500.18 MHz, 125.77 MHz).

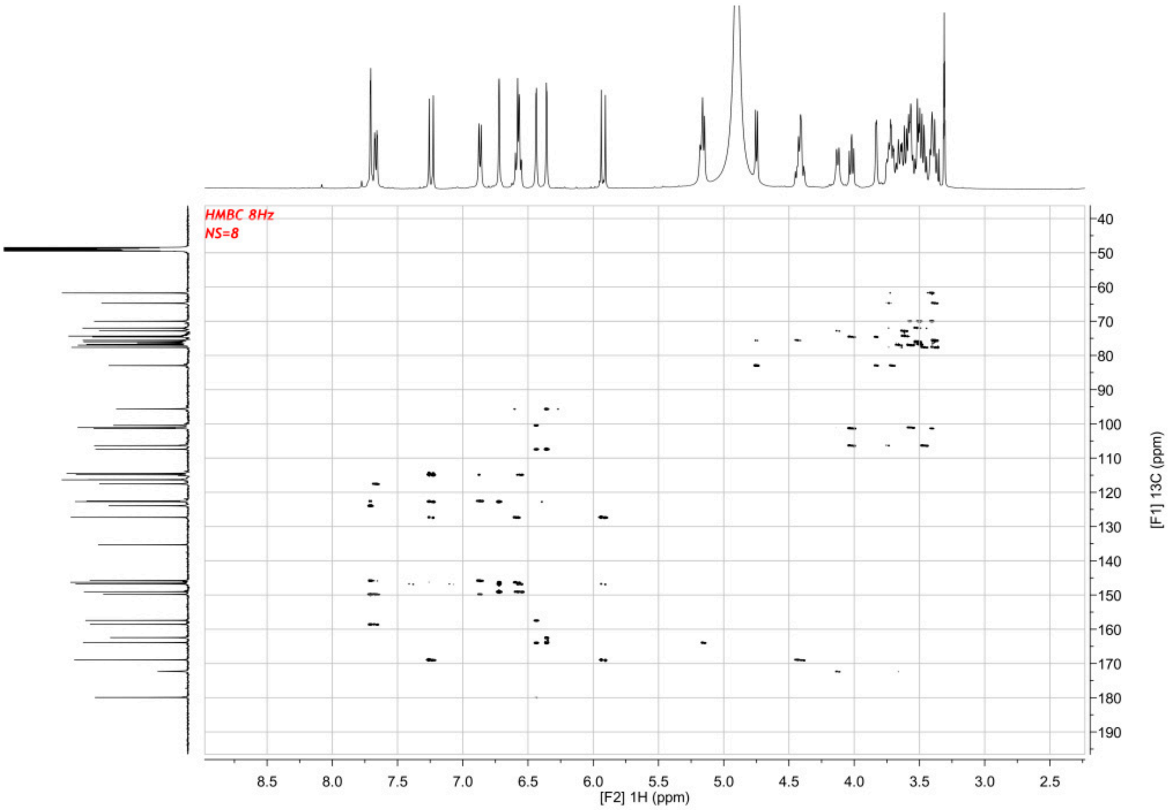

Figure S52. 1D TOCSY and 1D ROESY NMR subspectra of H-1(7-*O*-β-GlcA) and H-1(3-*O*-β-Gal) in compound **5** (methanol-d_4, 500.18 MHz).

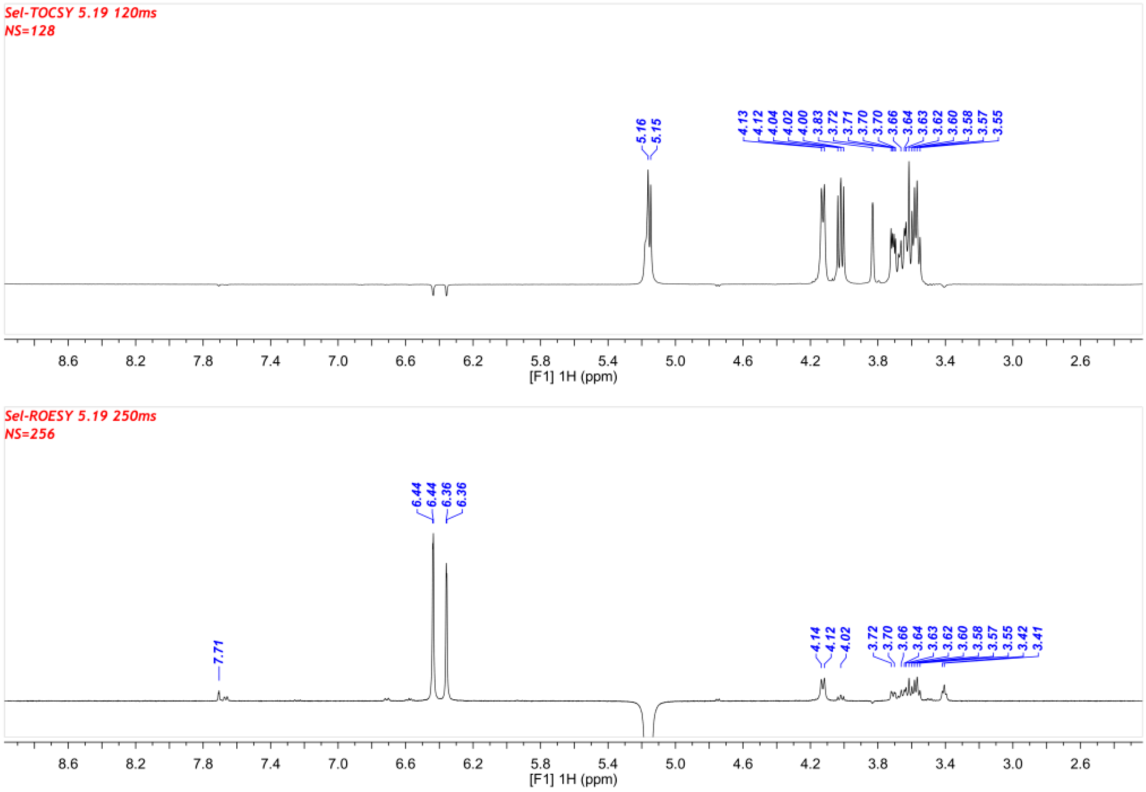

Figure S53. 1D TOCSY and 1D ROESY NMR subspectra of H-1$_{(2^{Gal}\text{-}O\text{-}\beta\text{-}Glc)}$ in compound **5** (methanol-d_4, 500.18 MHz).

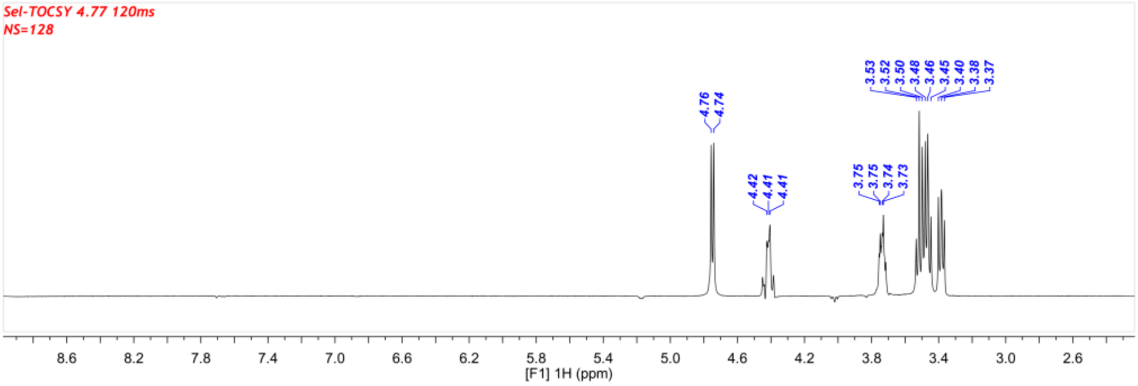

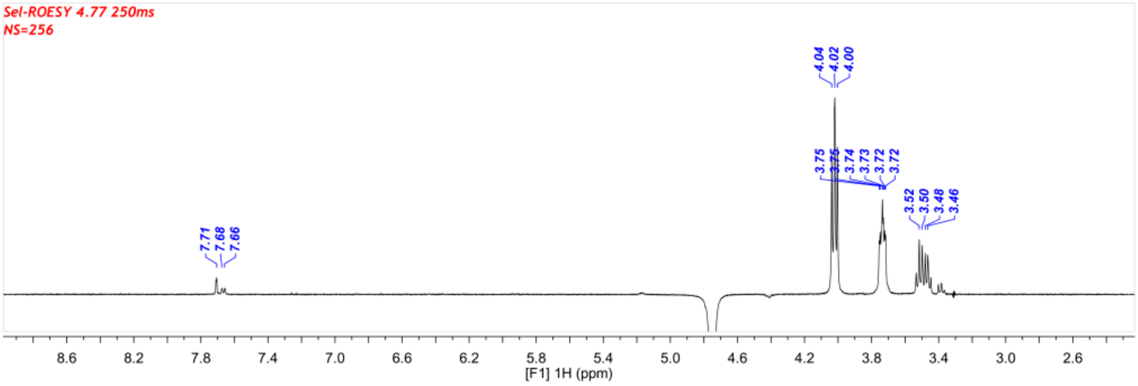

Compound 6

Figure S54. 1D ^{1}H-NMR spectrum of compound **6** (methanol-d_4, 500.18 MHz).

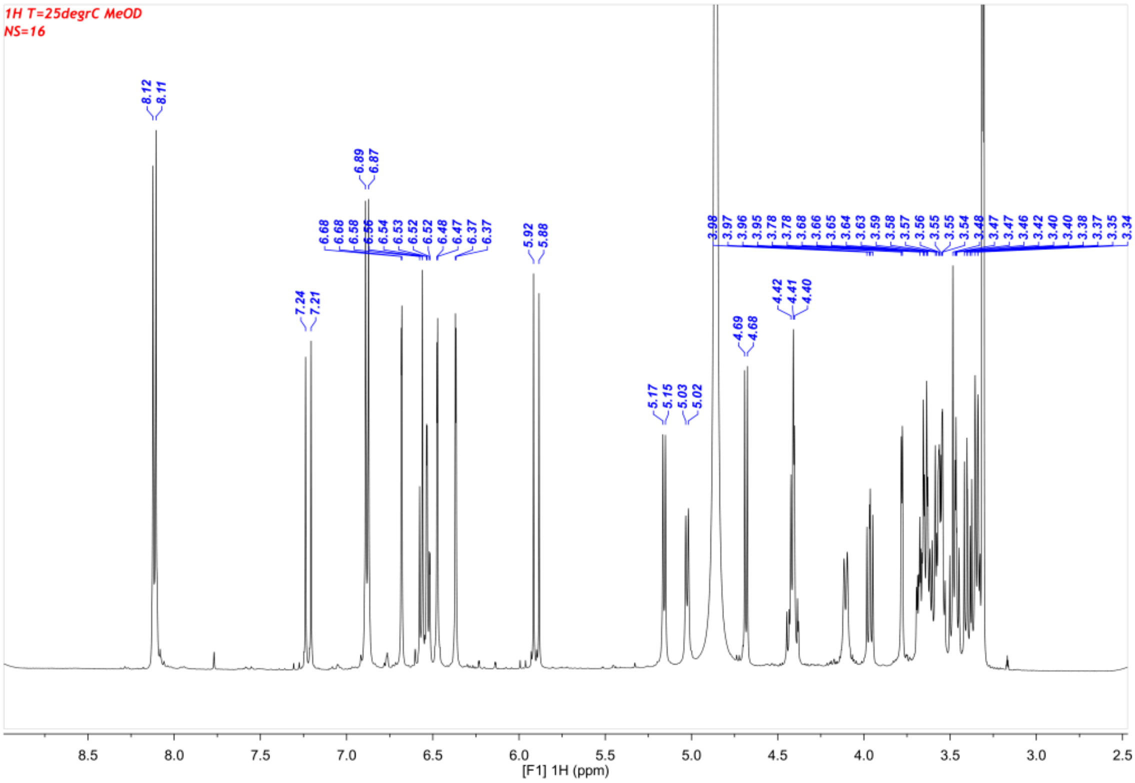

Figure S55. 1D ^{13}C-NMR spectra (1H decoupled and DEPT-135) of compound **6** (methanol-d_4, 125.77 MHz).

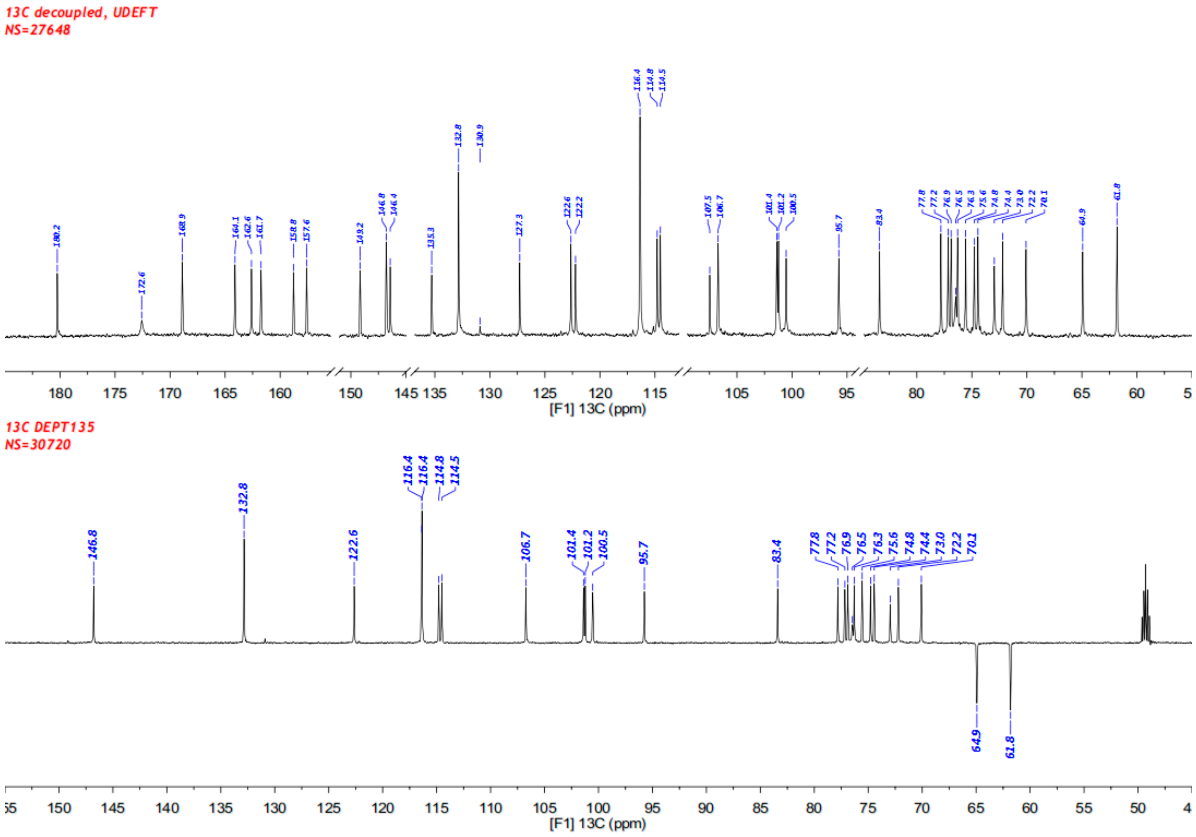

Figure S56. 2D *g*-COSY NMR spectrum of compound **6** (methanol-d_4, 500.18 MHz).

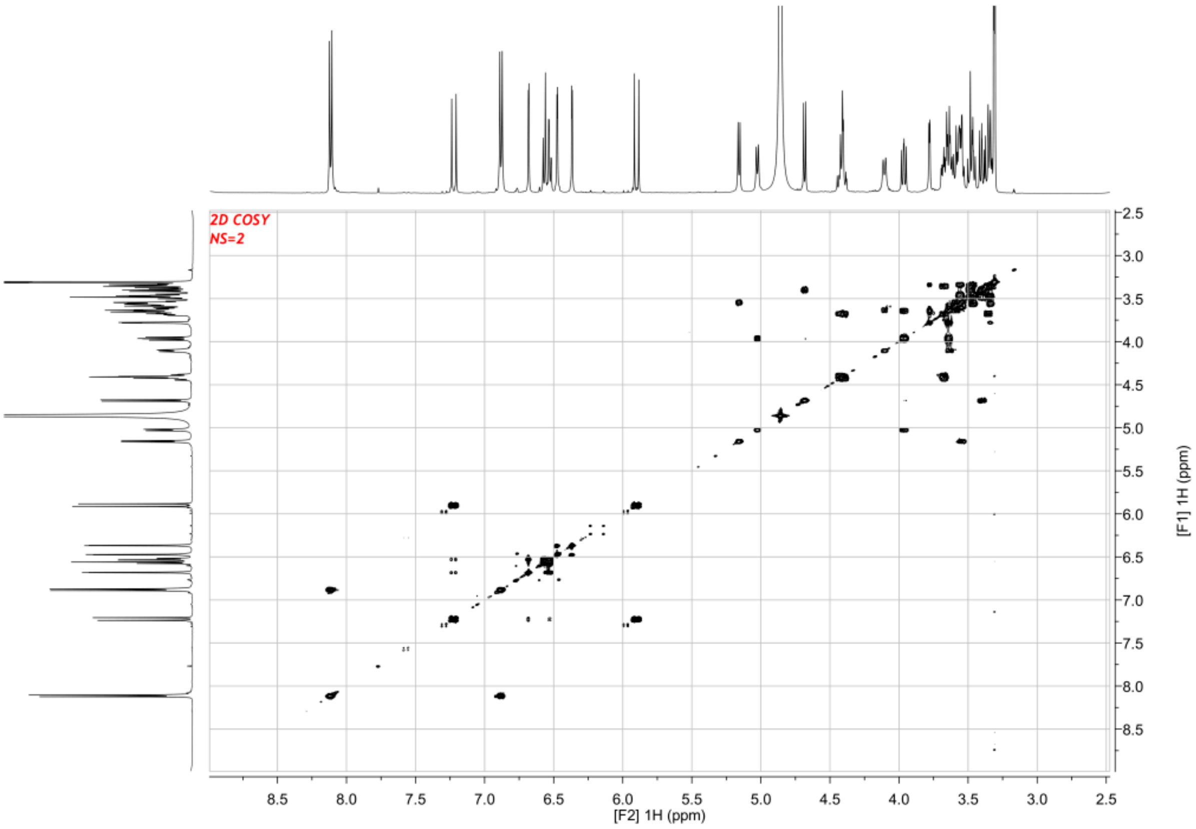

Figure S57. 2D TOCSY NMR spectrum of compound **6** (methanol-*d*4, 500.18 MHz).

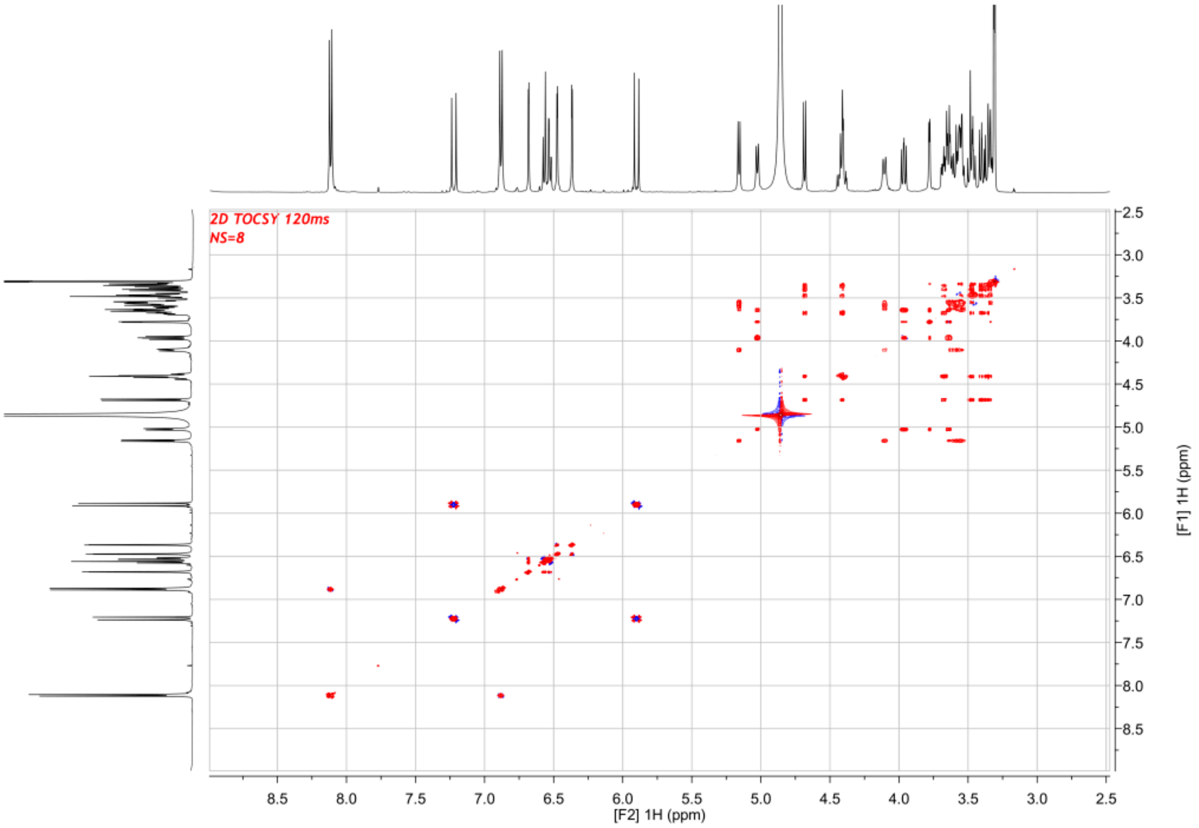

Figure S58. 2D ROESY NMR spectrum of compound **6** (methanol-*d*4, 500.18 MHz).

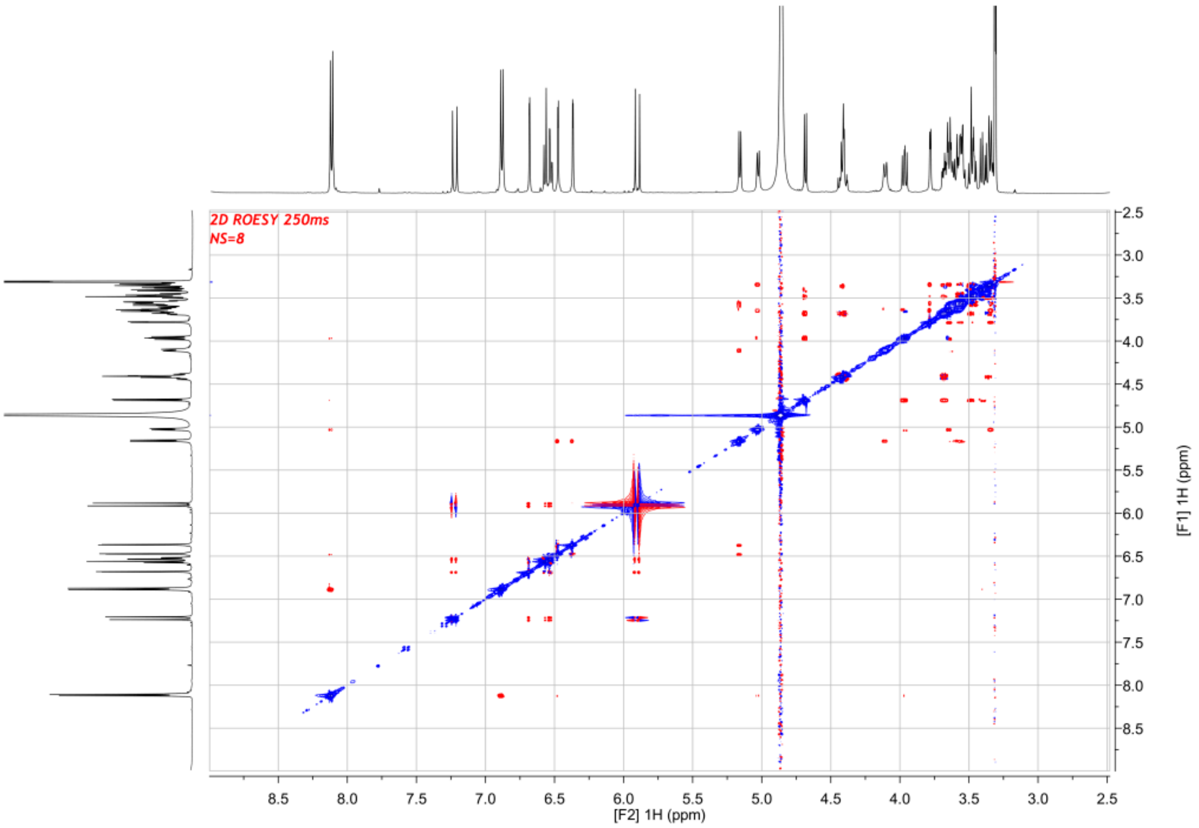

Figure S59. 2D *g*-HSQC-NMR spectrum of compound **6** (methanol-*d*4, 500.18 MHz, 125.77 MHz).

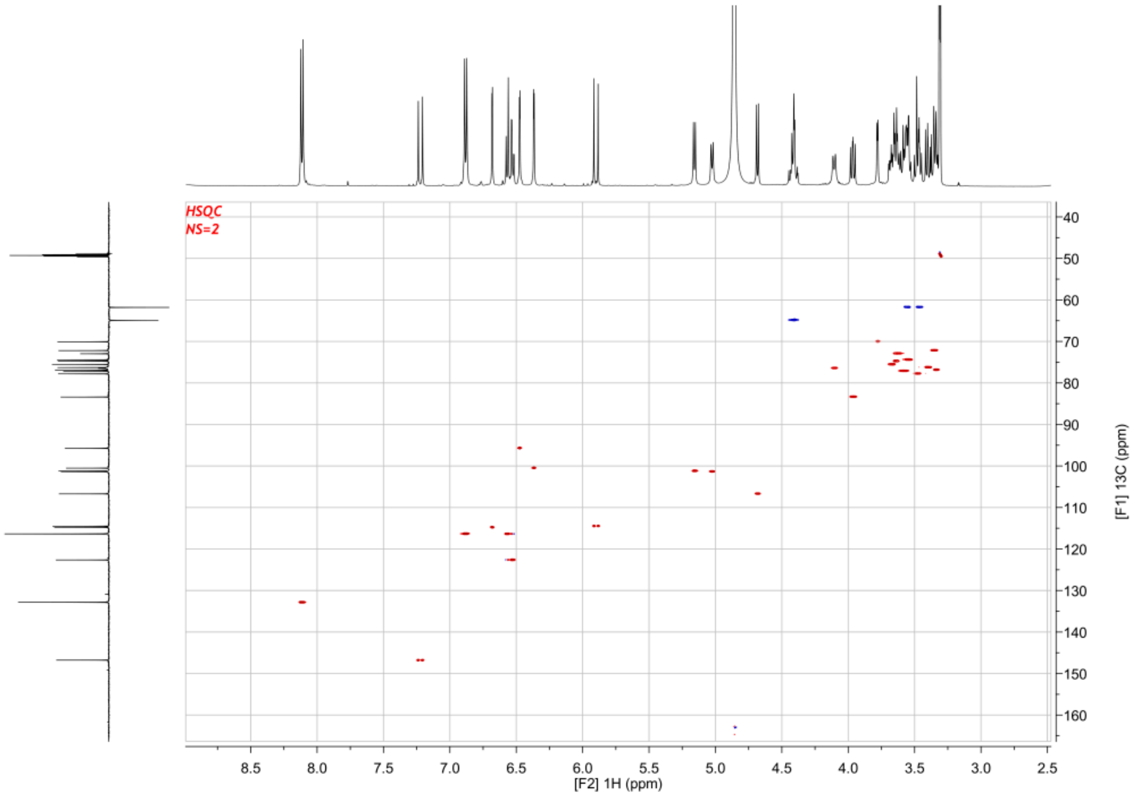

Figure S60. 2D *g*-HSQC-TOCSY NMR spectrum of compound **6** (methanol-*d*4, 500.18 MHz, 125.77 MHz).

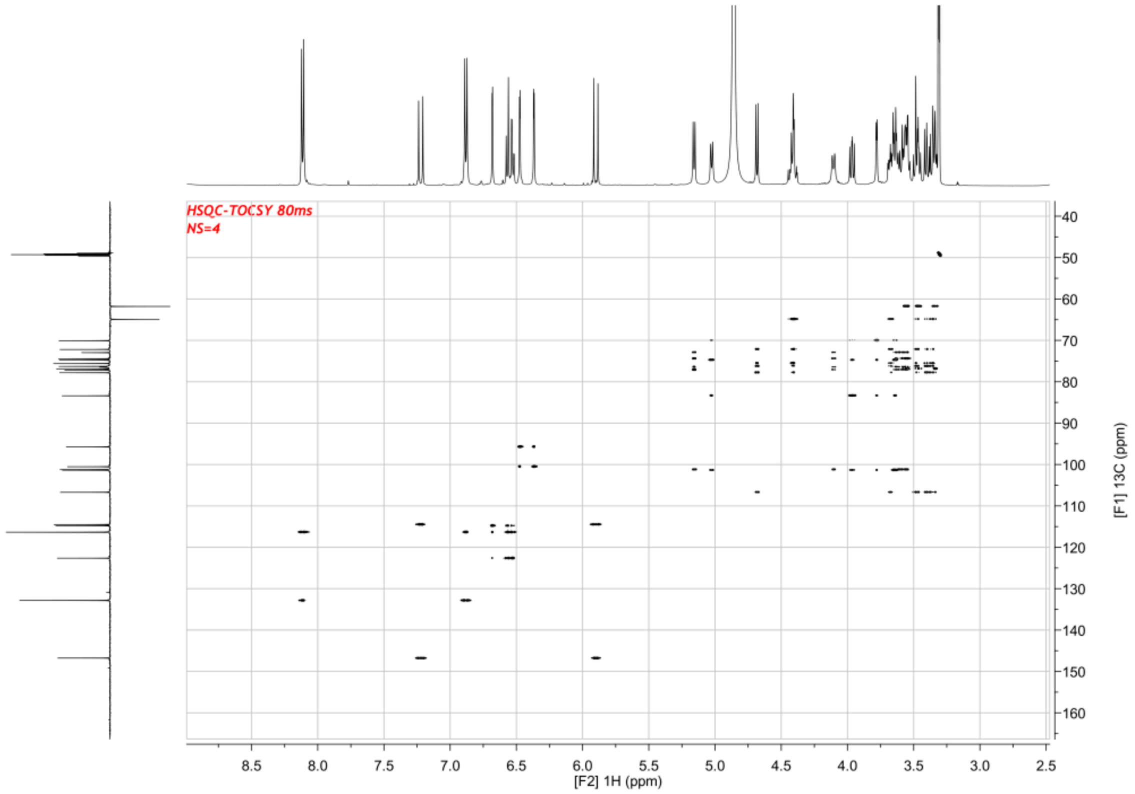

Figure S61. 2D *g*-HMBC-NMR spectrum of compound **6** (methanol-d_4, 500.18 MHz, 125.77 MHz).

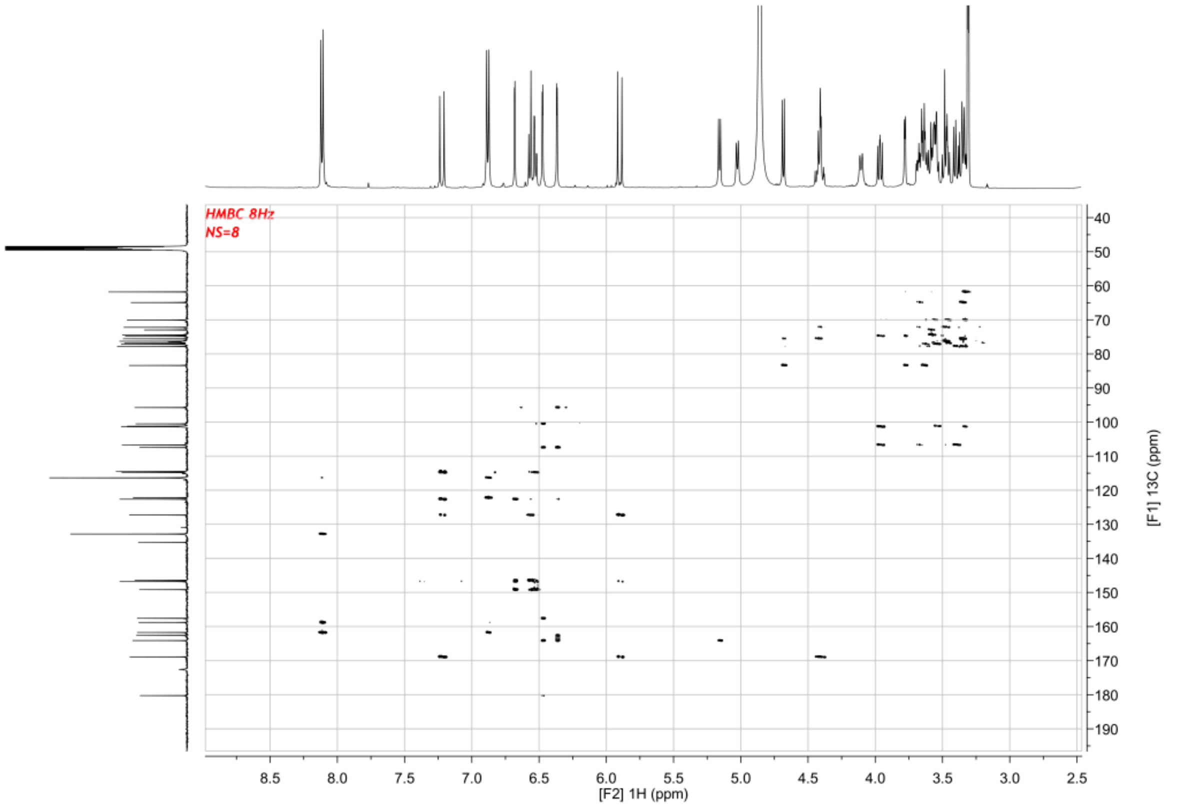

Figure S62. 1D TOCSY and 1D ROESY NMR subspectra of H-1(7-*O*-β-GlcA) in compound **6** (methanol-d_4, 500.18 MHz).

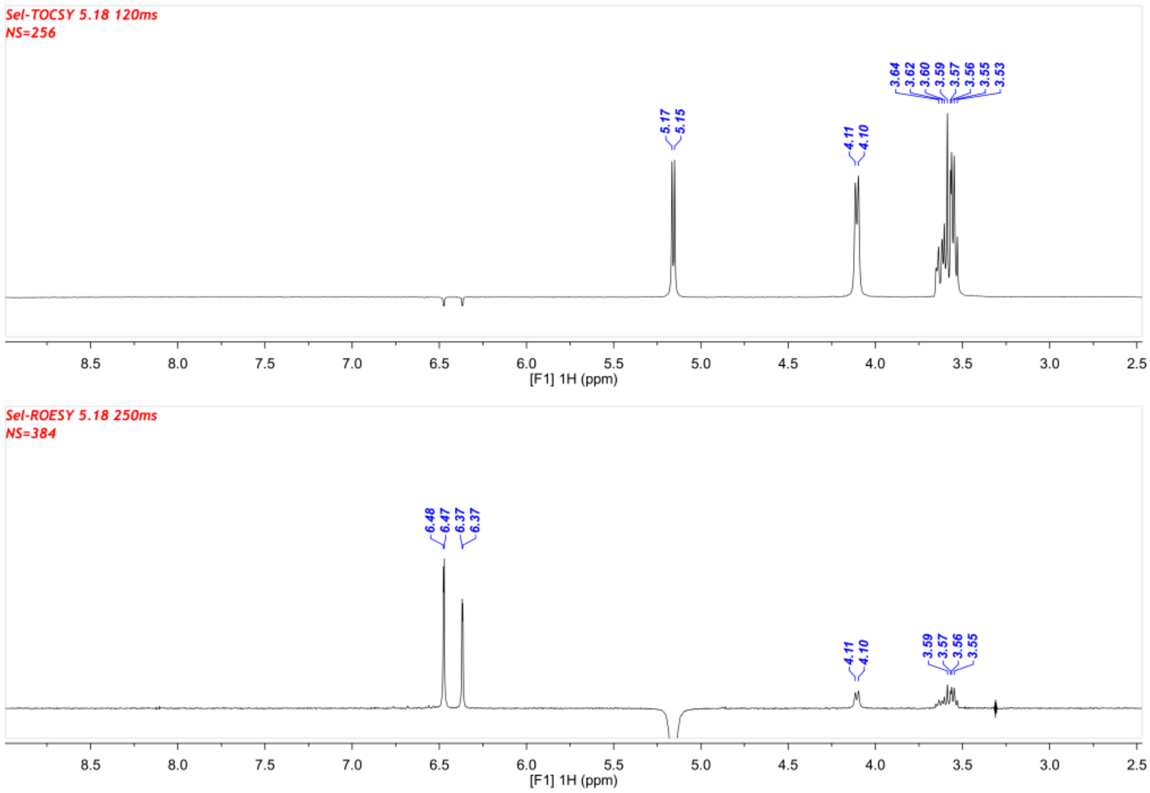

Figure S63. 1D TOCSY and 1D ROESY NMR subspectra of H-1(3-*O*-β-Gal) in compound **6** (methanol-*d*4, 500.18 MHz).

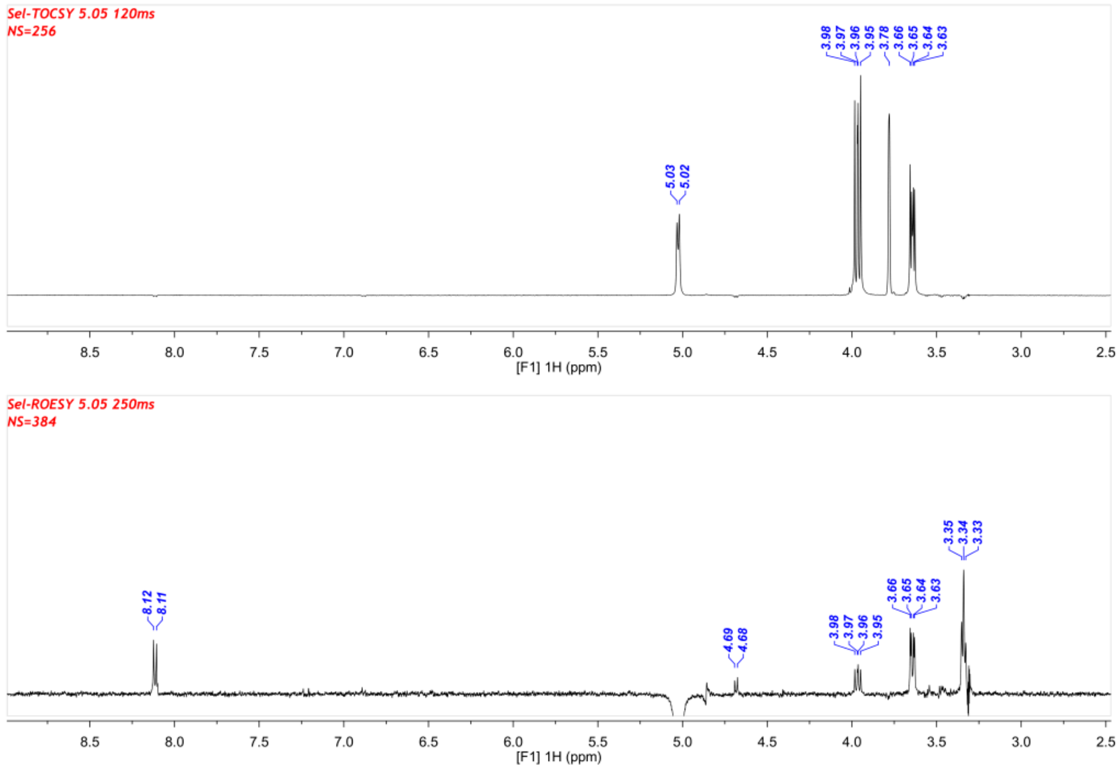

Figure S64. 1D TOCSY and 1D ROESY NMR subspectra of H-1(2^{Gal}-*O*-β-Glc) in compound **6** (methanol-*d*4, 500.18 MHz).

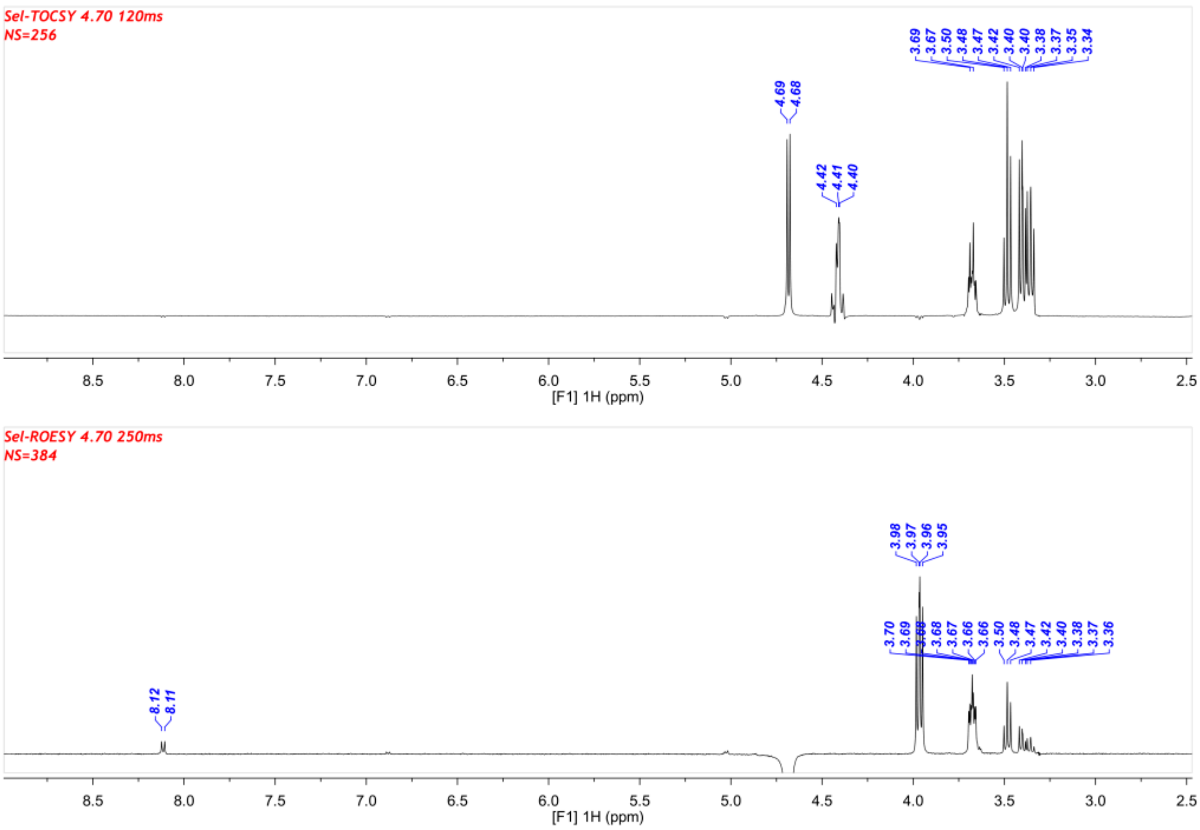

Compound 7

Figure S65. 1D ^{1}H-NMR spectrum of compound **7** (methanol-*d*4, 500.18 MHz).

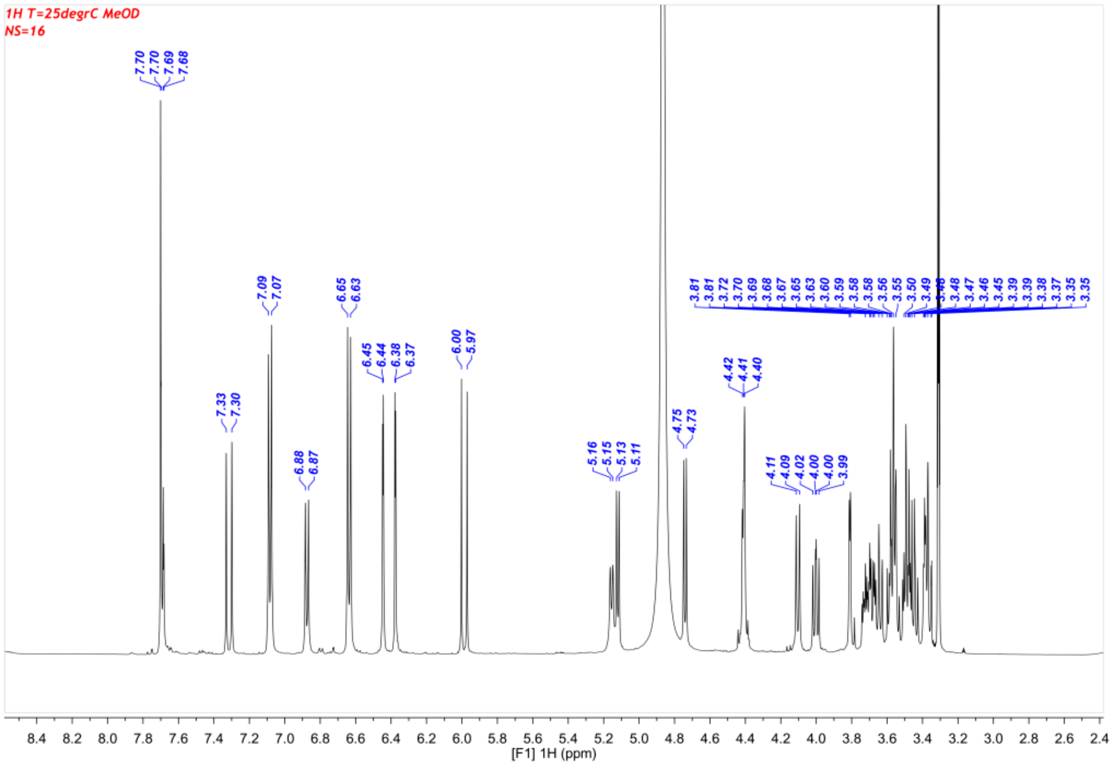

Figure S66. 1D ^{13}C-NMR spectra (1H decoupled and DEPT-135) of compound **7** (methanol-*d*4, 125.77 MHz).

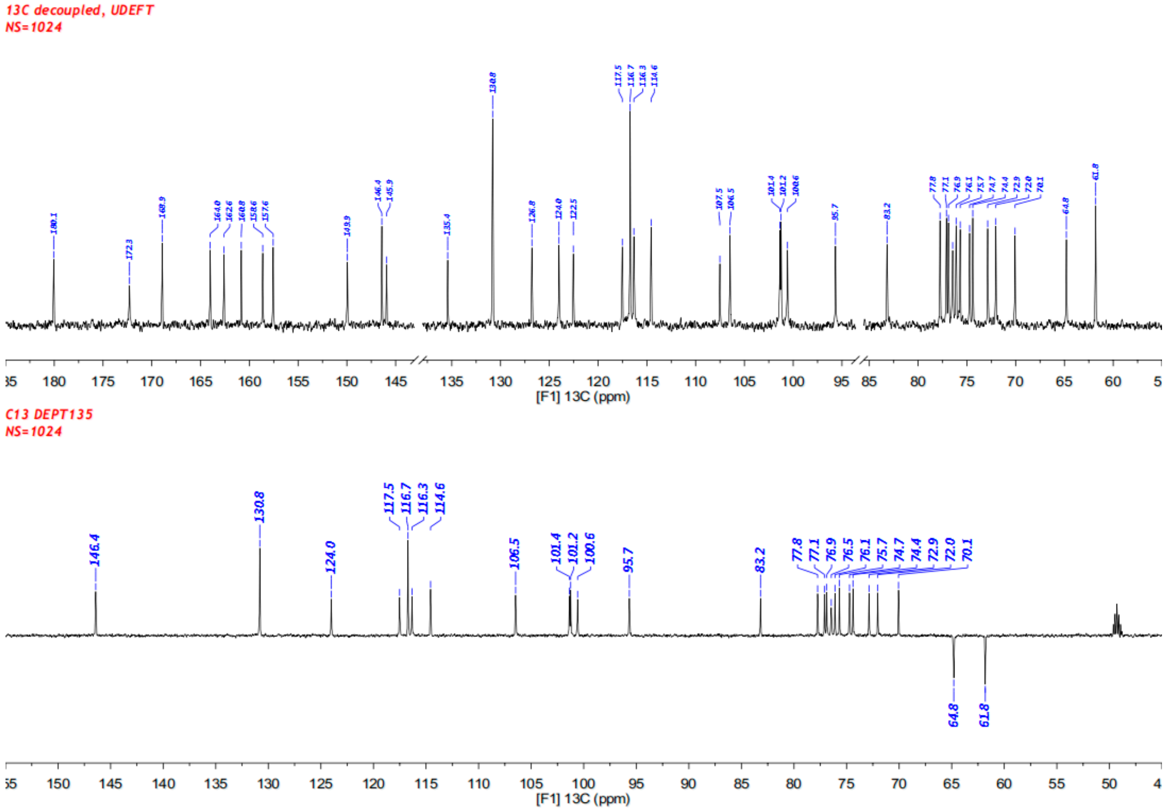

Figure S67. 2D *g*-COSY NMR spectrum of compound **7** (methanol-d_4, 500.18 MHz).

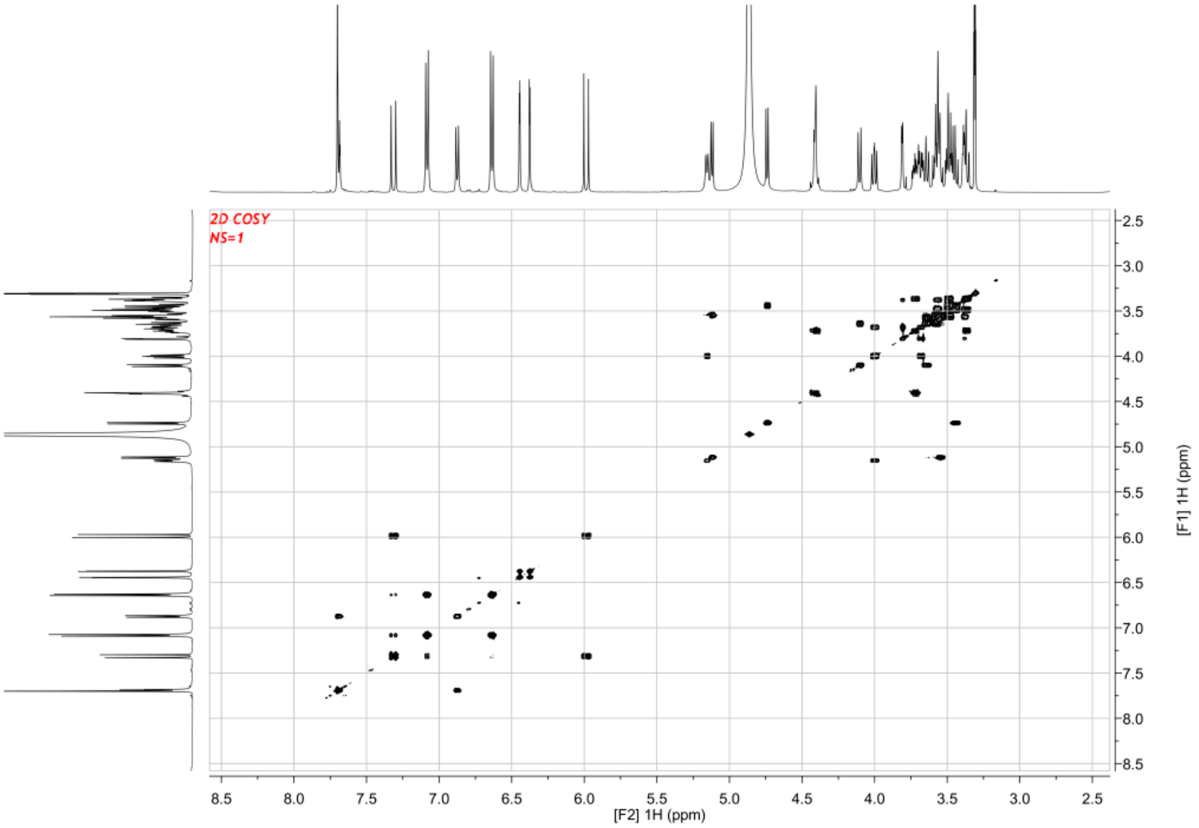

Figure S68. 2D TOCSY NMR spectrum of compound **7** (methanol-d_4, 500.18 MHz).

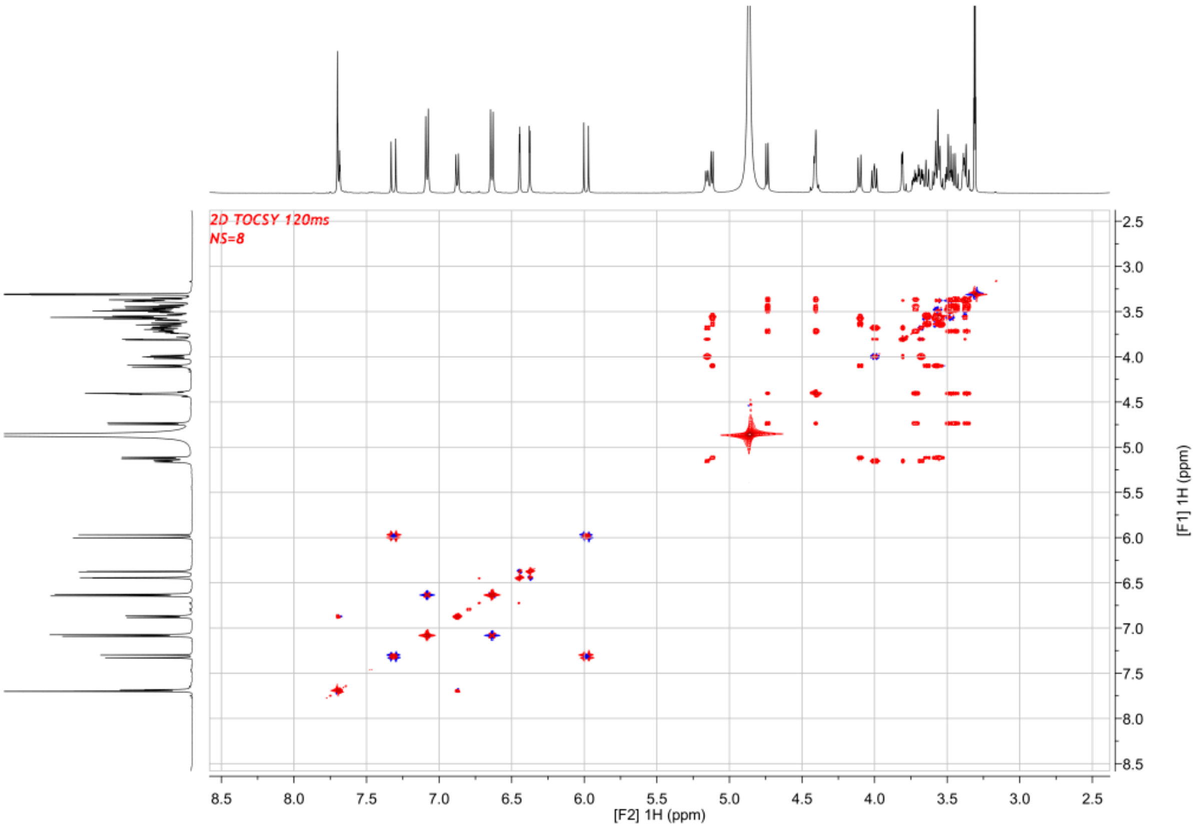

Figure S69. 2D ROESY NMR spectrum of compound **7** (methanol-*d*4, 500.18 MHz).

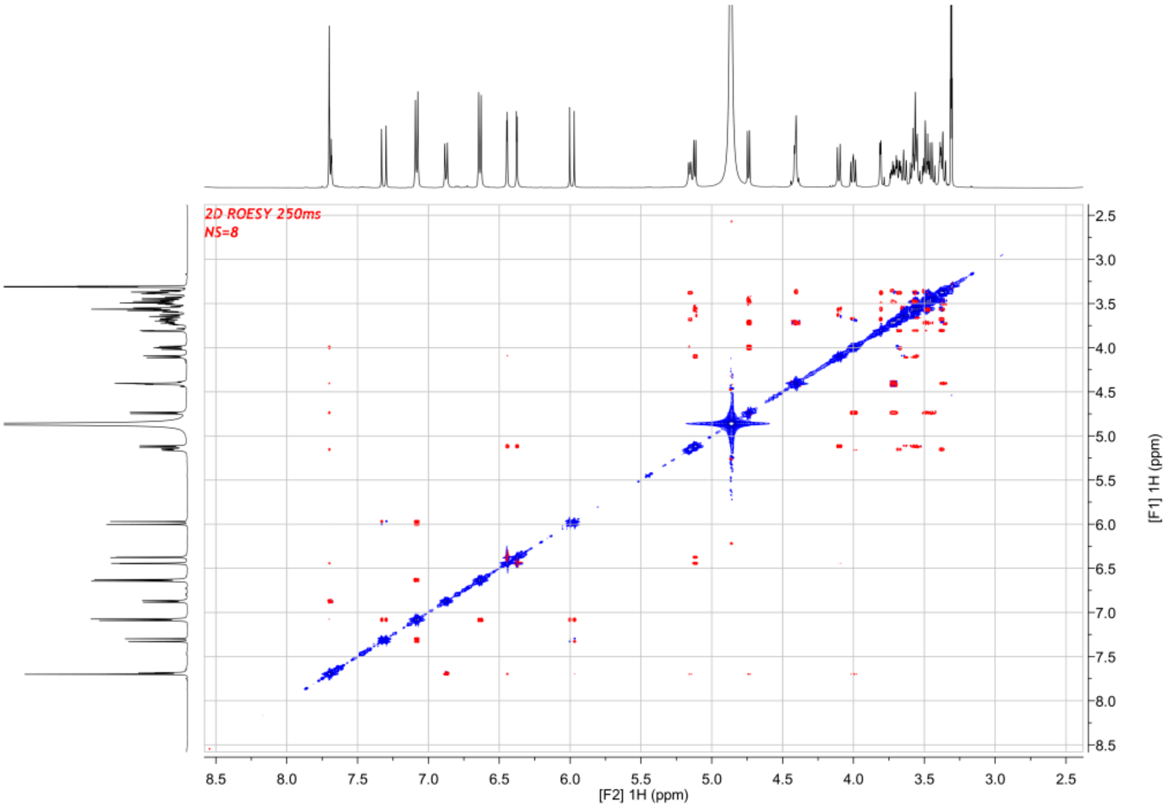

Figure S70. 2D *g*-HSQC-NMR spectrum of compound **7** (methanol-*d*4, 500.18 MHz, 125.77 MHz).

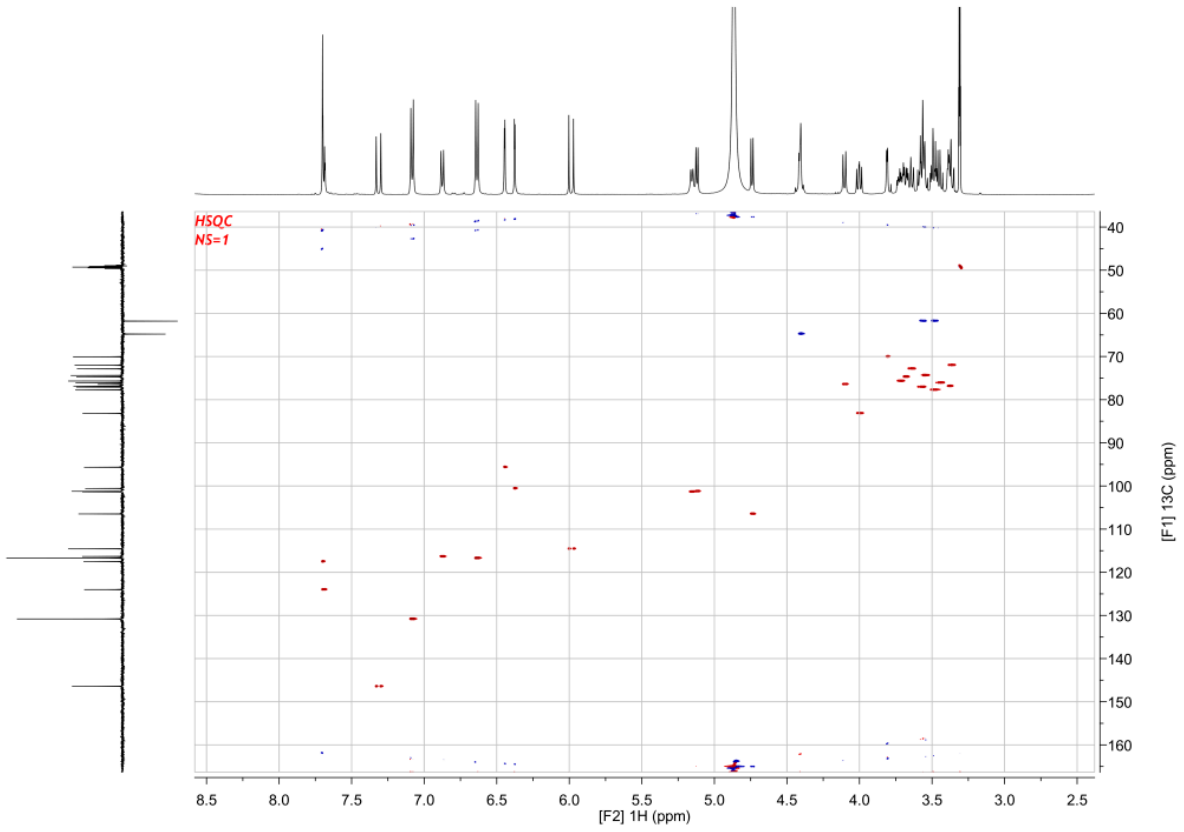

Figure S71. 2D *g*-HSQC-TOCSY NMR spectrum of compound **7** (methanol-*d*4, 500.18 MHz, 125.77 MHz).

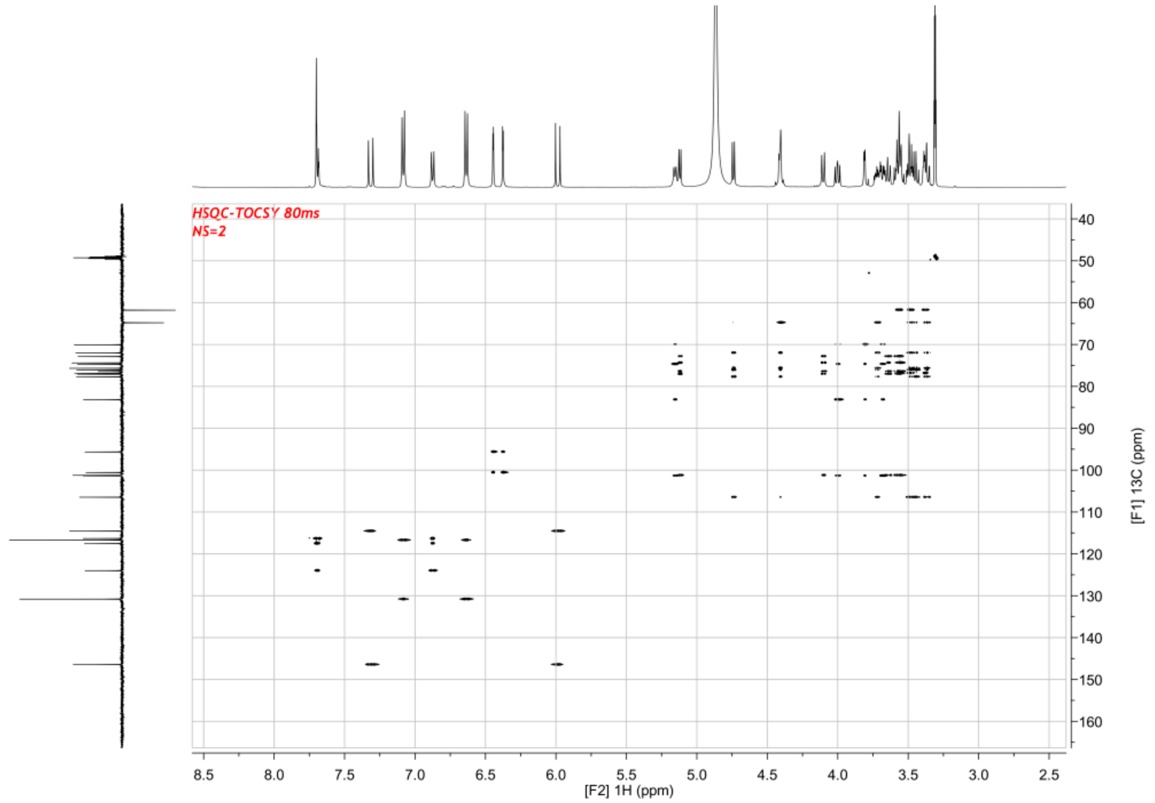

Figure S72. 2D *g*-HMBC-NMR spectrum of compound **7** (methanol-*d*4, 500.18 MHz, 125.77 MHz).

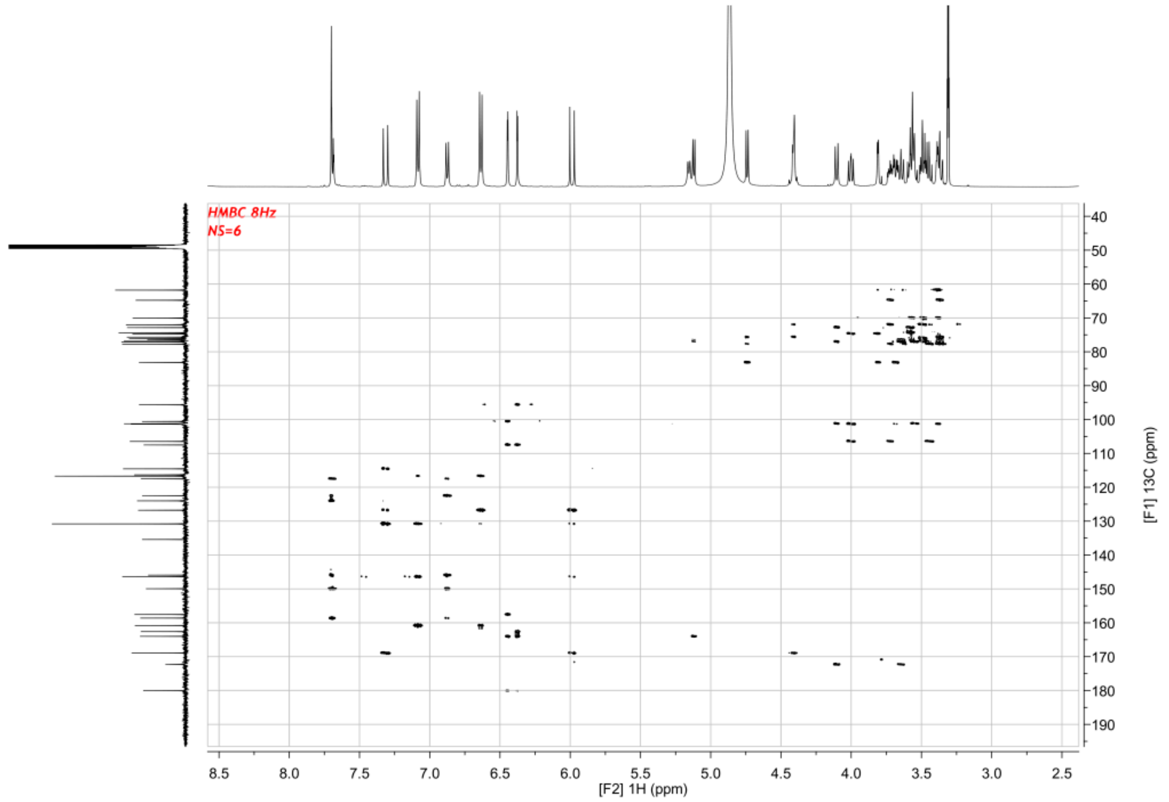

Figure S73. 1D TOCSY and 1D ROESY NMR subspectra of H-1(7-*O*-β-GlcA) in compound **7** (methanol-d_4, 500.18 MHz).

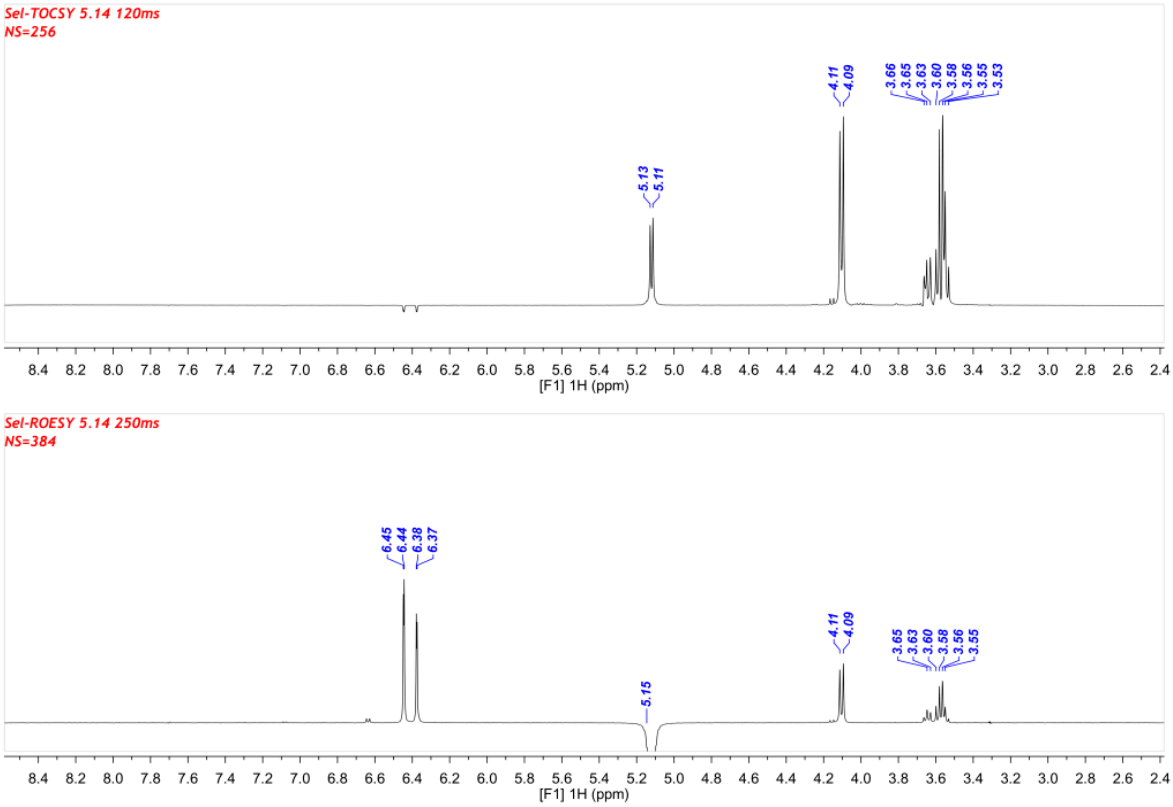

Figure S74. 1D TOCSY and 1D ROESY NMR subspectra of H-1(3-*O*-β-Gal) in compound **7** (methanol-d_4, 500.18 MHz).

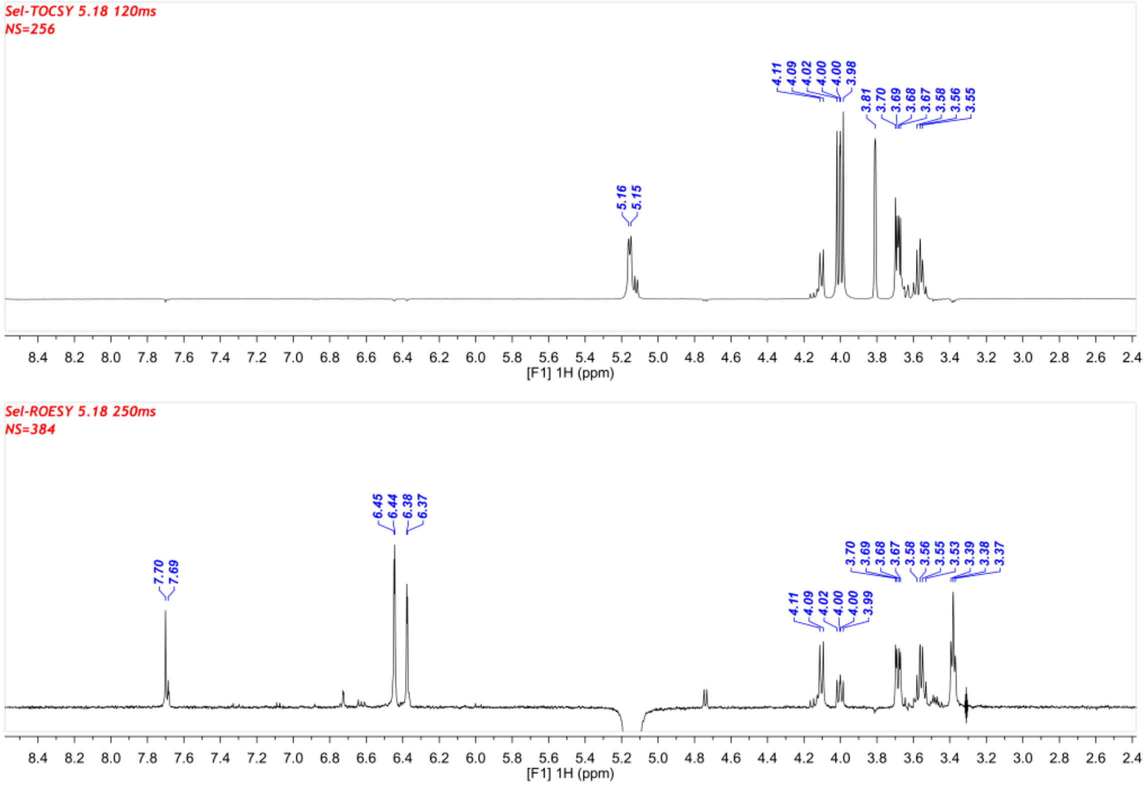

Figure S75. 1D TOCSY and 1D ROESY NMR subspectra of H-1$_{(2^{Gal}\text{-}O\text{-}\beta\text{-}Glc)}$ in compound **7** (methanol-*d*4, 500.18 MHz).

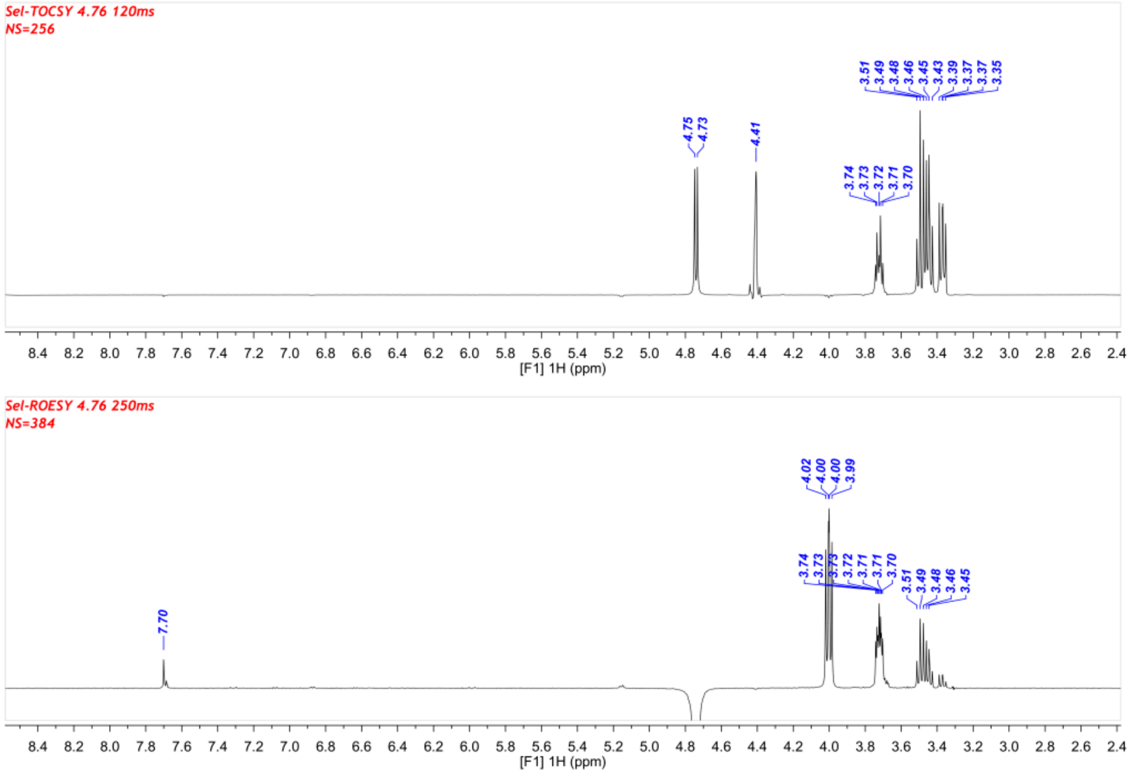

Compound 8

Figure S76. 1D ^{1}H-NMR spectrum of compound **8** (methanol-*d*4, 500.18 MHz).

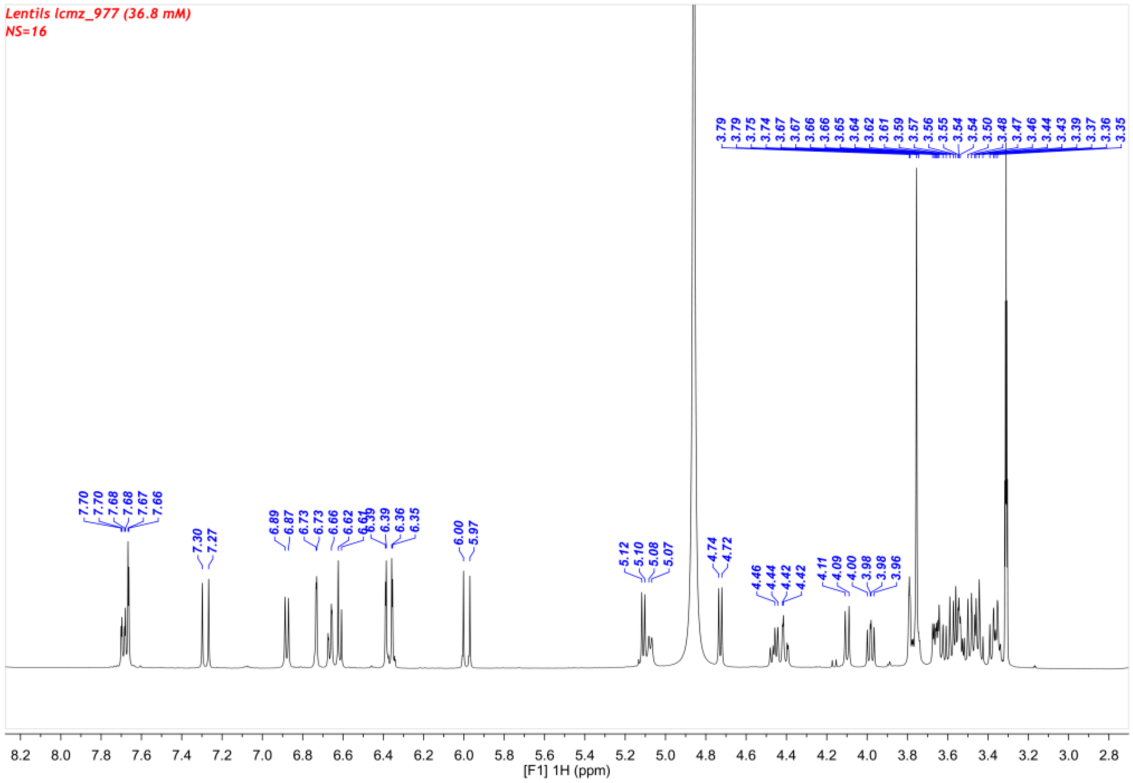

Figure S77. 1D ^{13}C-NMR spectra (1H decoupled and DEPT-135) of compound **8** (methanol-d_4, 125.77 MHz).

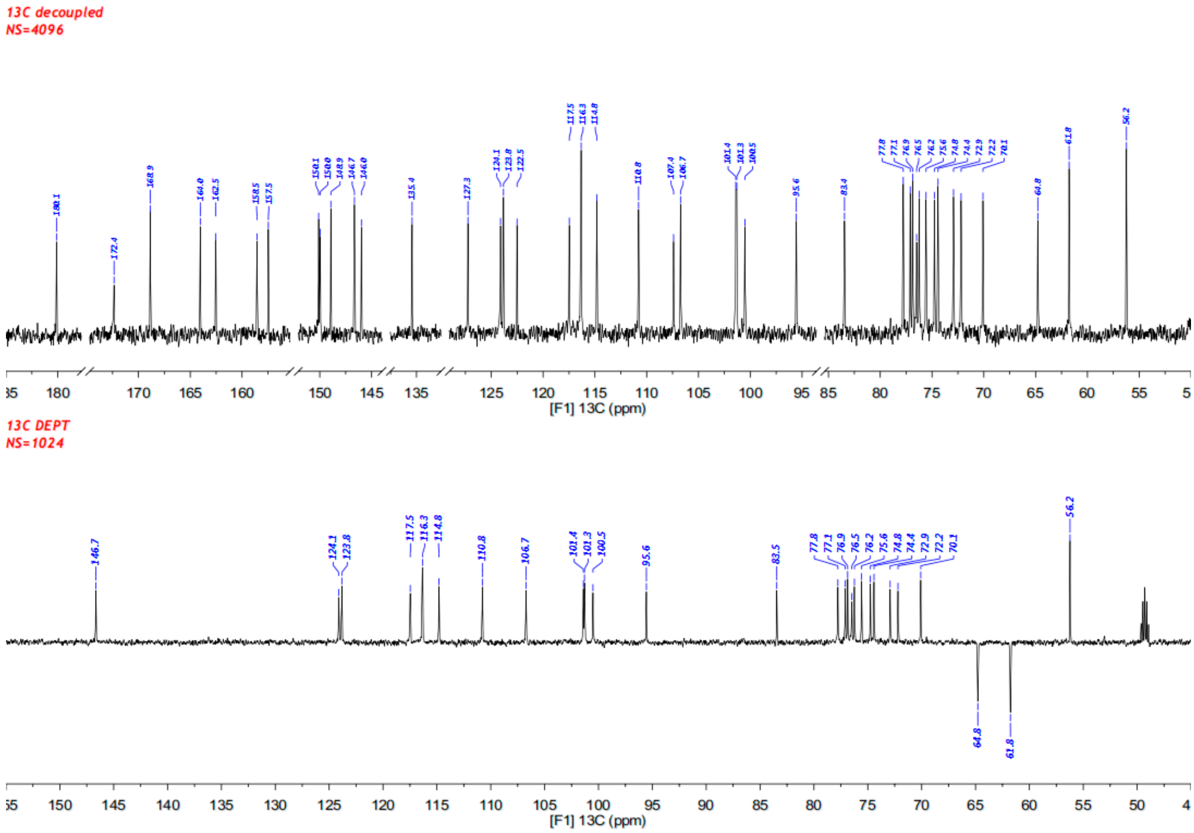

Figure S78. 2D *g*-COSY NMR spectrum of compound **8** (methanol-d_4, 500.18 MHz).

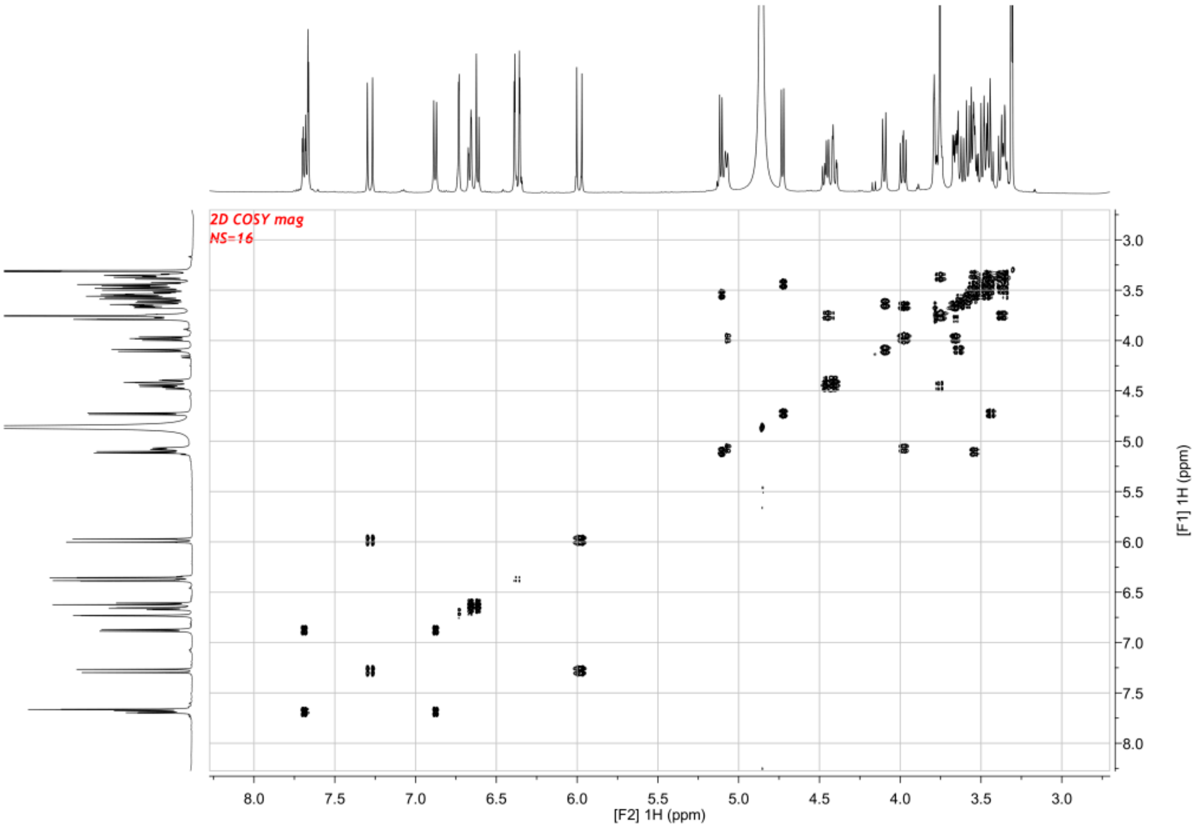

Figure S79. 2D TOCSY NMR spectrum of compound **8** (methanol-d_4, 500.18 MHz).

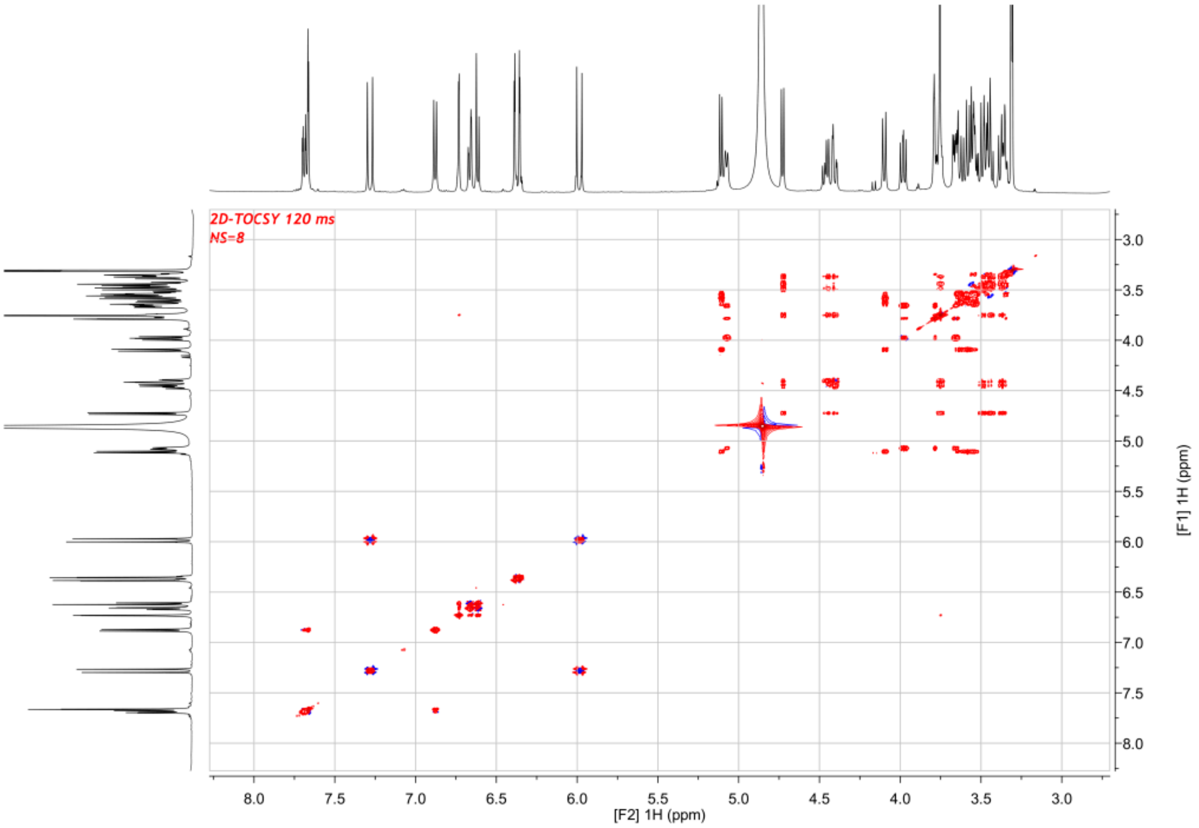

Figure S80. 2D ROESY NMR spectrum of compound **8** (methanol-d_4, 500.18 MHz).

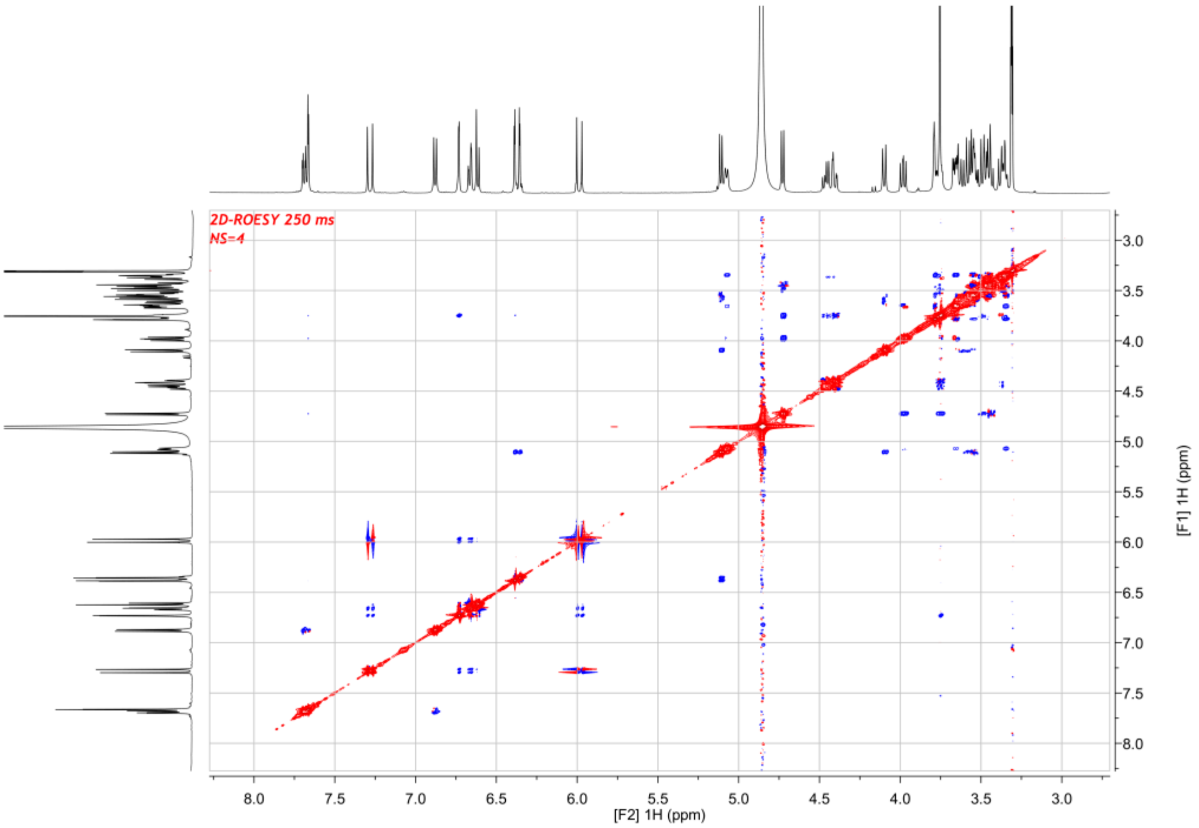

Figure S81. 2D *g*-HSQC-NMR spectrum of compound **8** (methanol-*d*4, 500.18 MHz, 125.77 MHz).

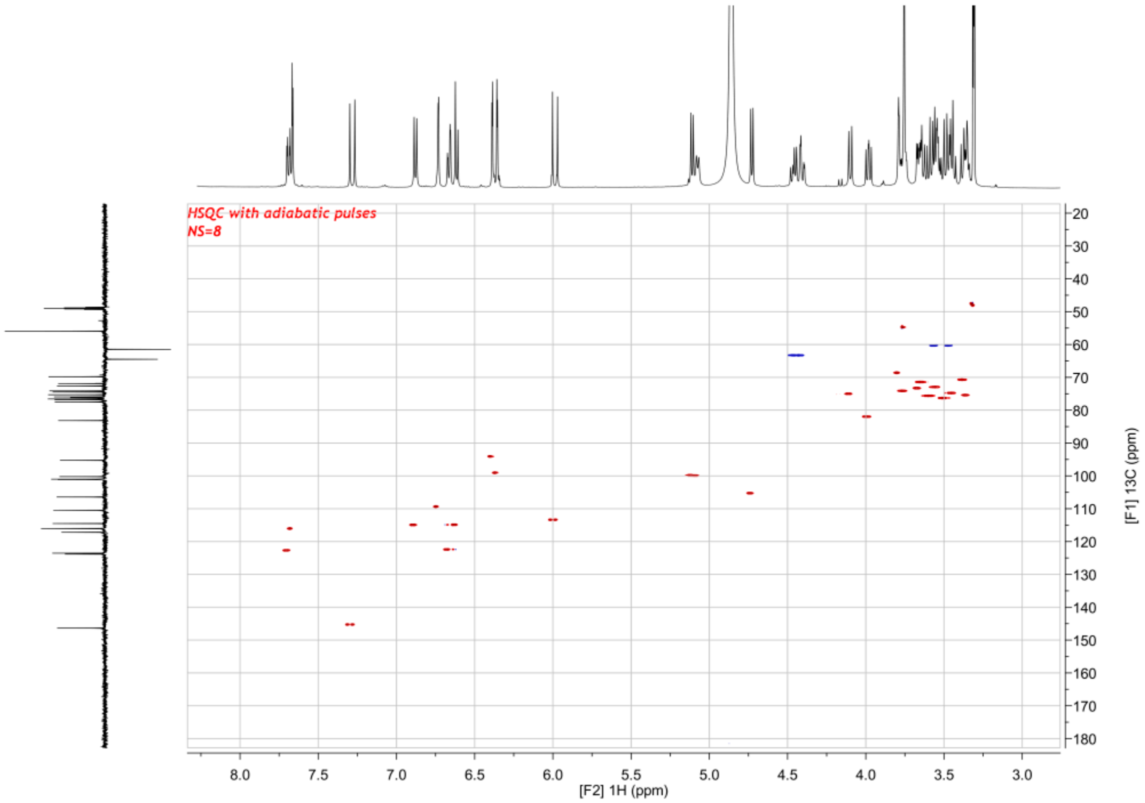

Figure S82. 2D *g*-HSQC-TOCSY NMR spectrum of compound **8** (methanol-*d*4, 500.18 MHz, 125.77 MHz).

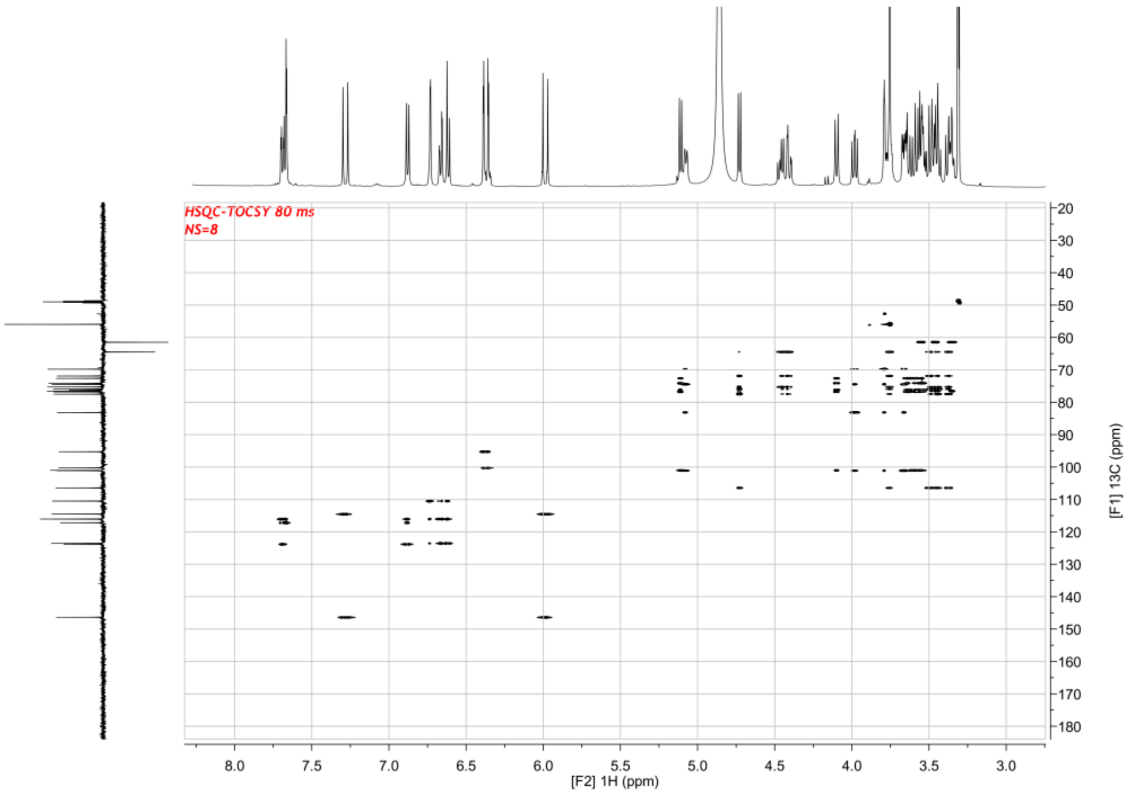

Figure S83. 2D *g*-HMBC-NMR spectrum of compound **8** (methanol-*d*4, 500.18 MHz, 125.77 MHz).

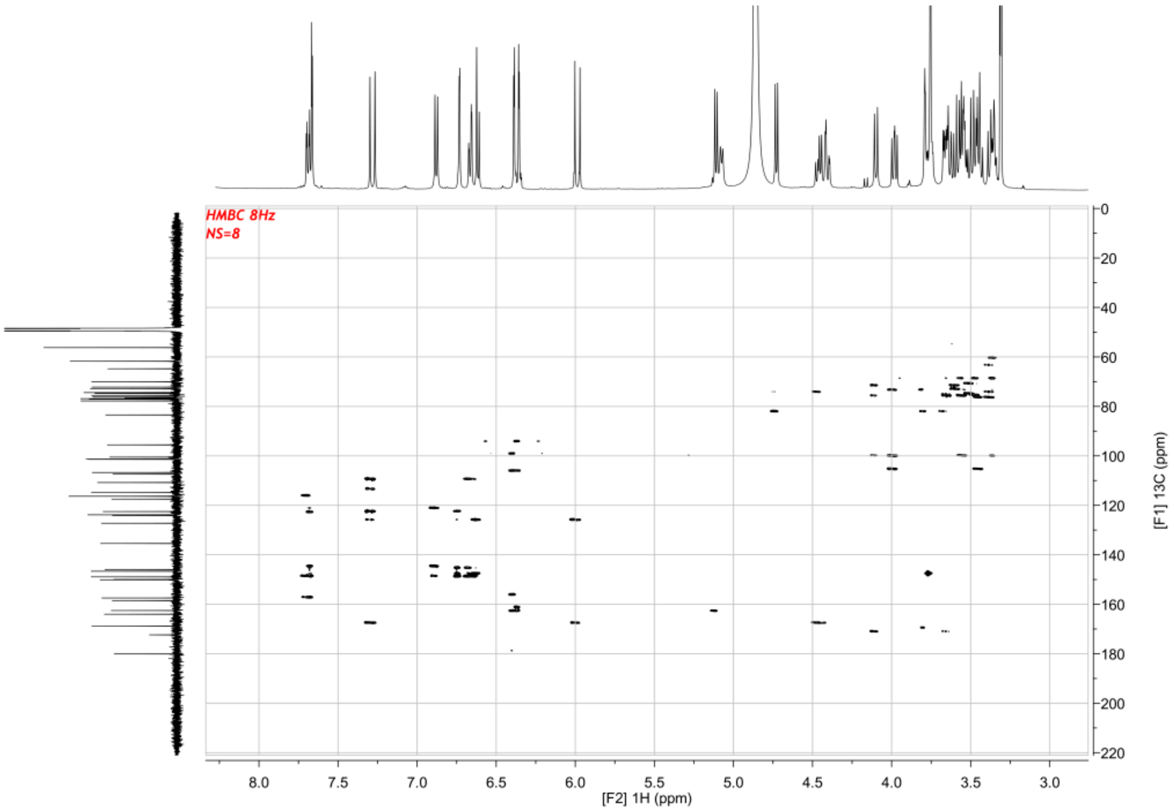

Figure S84. 1D TOCSY and 1D ROESY NMR subspectra of H-1(7-*O*-β-GlcA) in compound **8** (methanol-*d*4, 500.18 MHz).

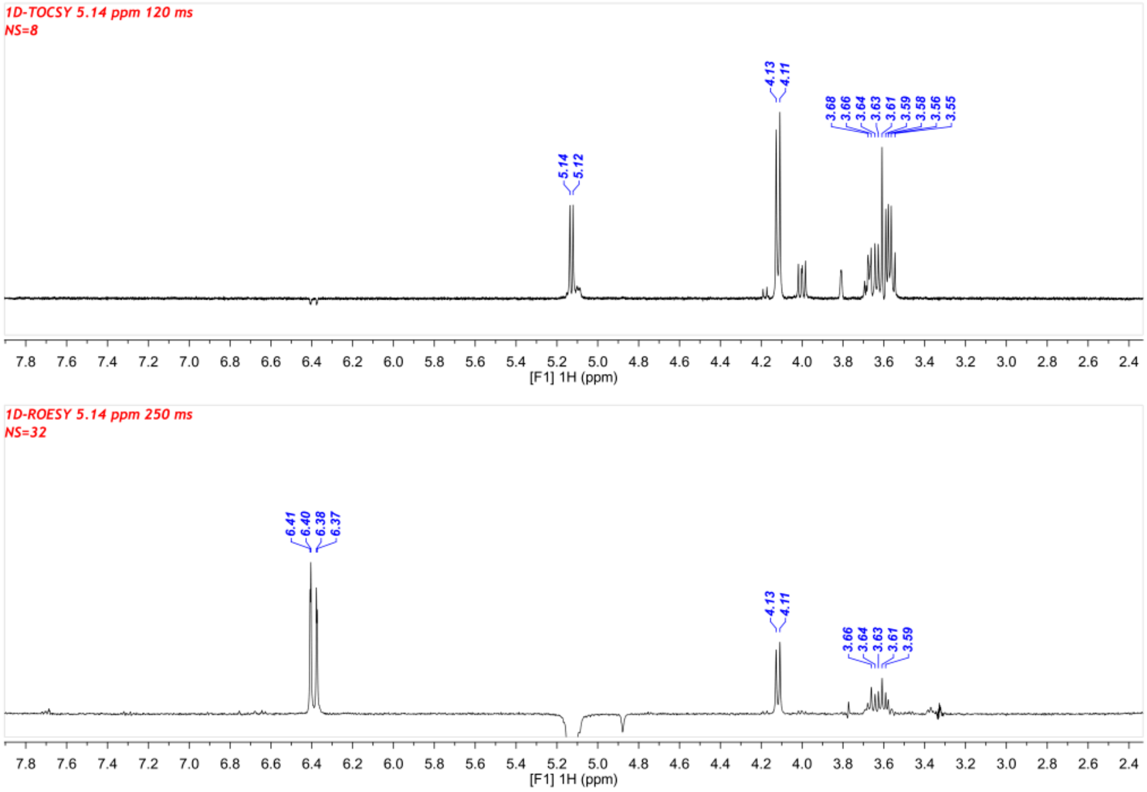

Figure S85. 1D TOCSY and 1D ROESY NMR subspectra of H-1$_{(3\text{-}O\text{-}\beta\text{-Gal})}$ in compound **8** (methanol-d_4, 500.18 MHz).

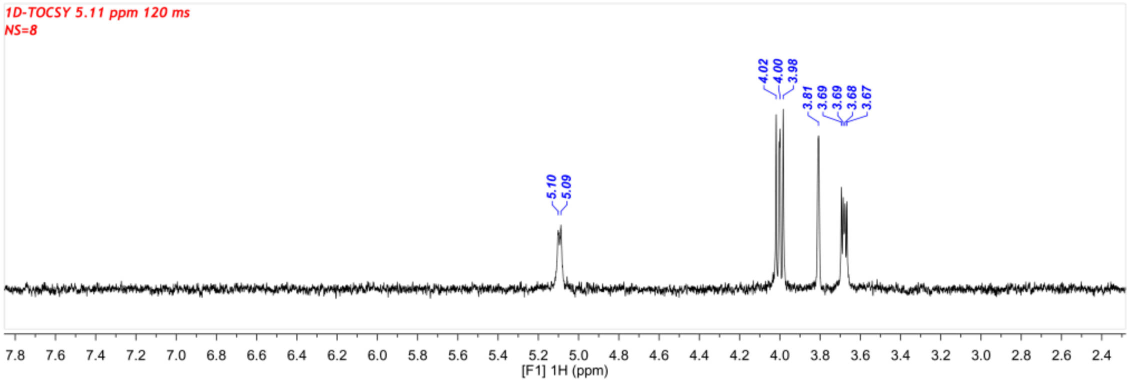

Figure S86. 1D TOCSY and 1D ROESY NMR subspectra of H-1$_{(2^{Gal}\text{-}O\text{-}\beta\text{-Glc})}$ in compound **8** (methanol-d_4, 500.18 MHz).

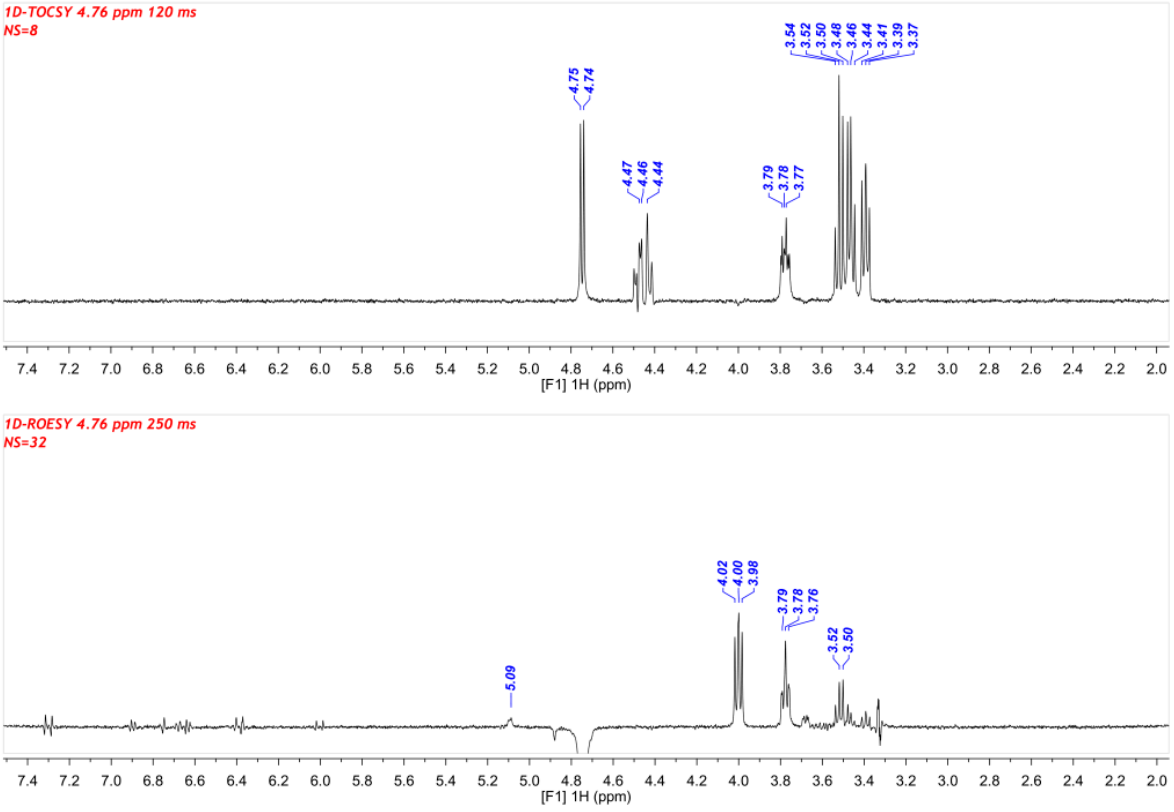

Compound 9

Figure S87. 1D ^{1}H-NMR spectrum of compound **9** (methanol-d_4, 500.18 MHz).

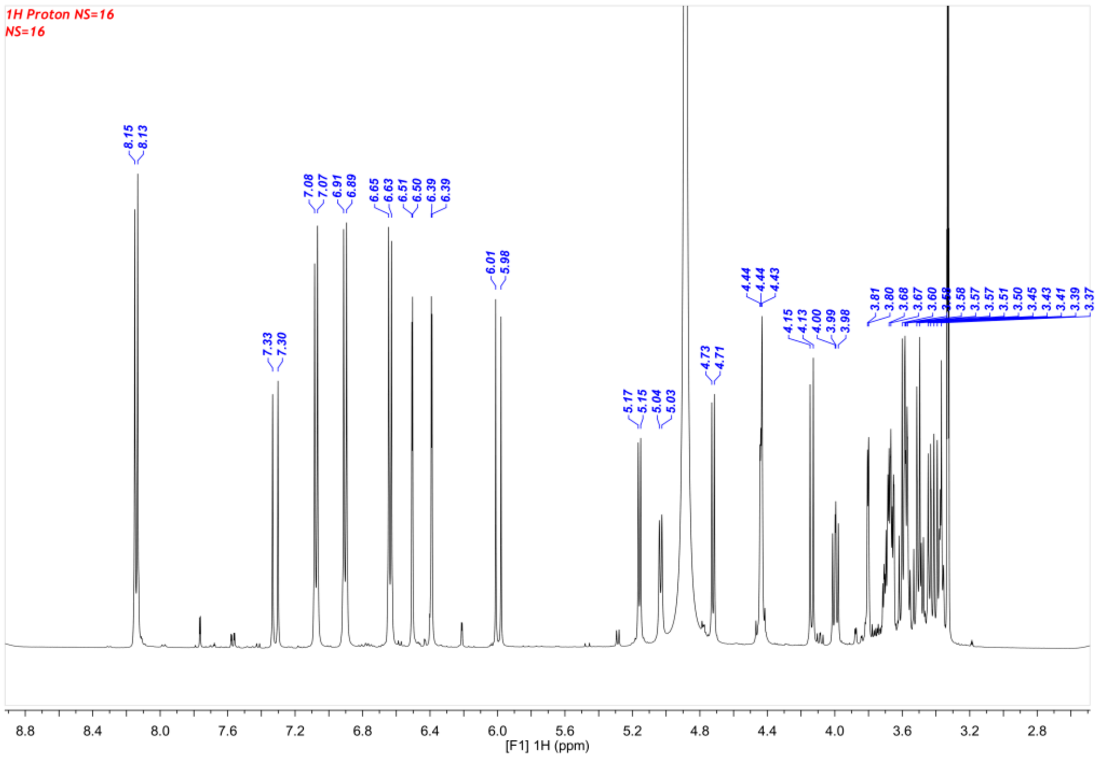

Figure S88. 1D ^{13}C-NMR spectra (1H decoupled and DEPT-135) of compound **9** (methanol-d_4, 125.77 MHz).

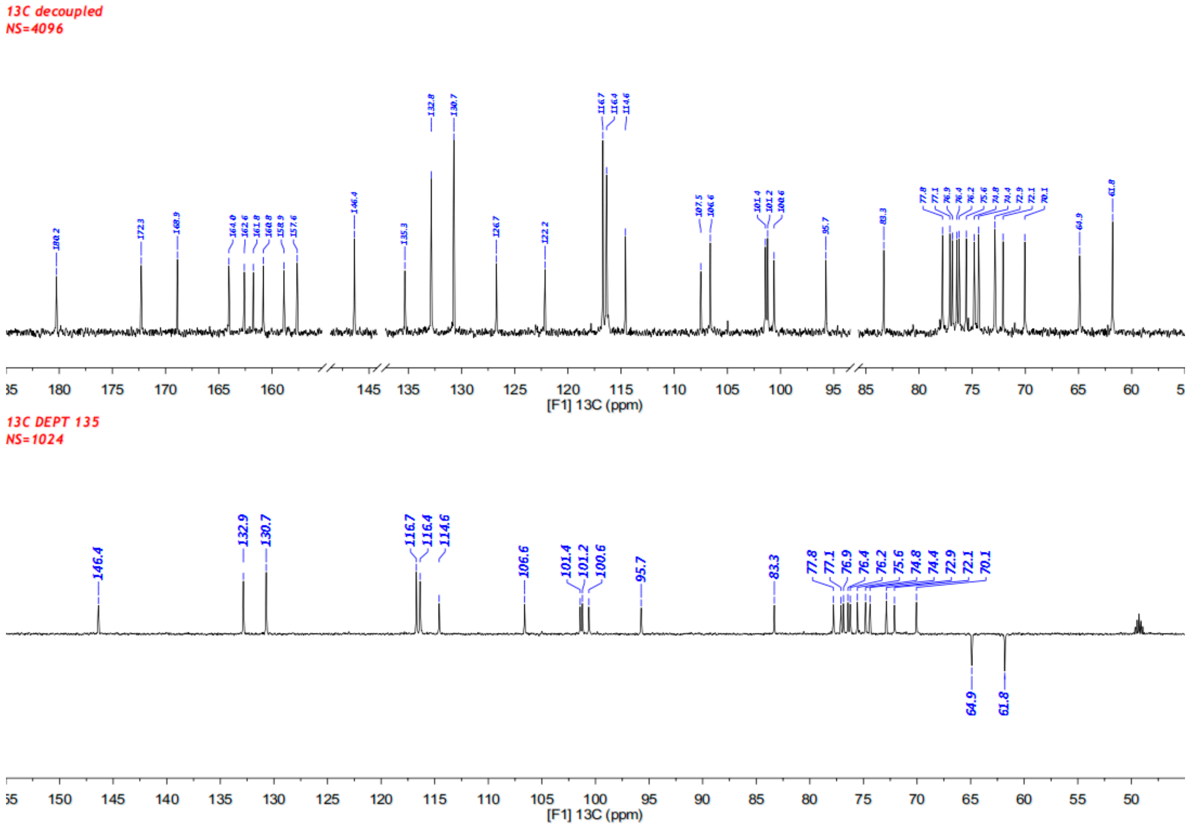

Figure S89. 2D *g*-COSY NMR spectrum of compound **9** (methanol-d_4, 500.18 MHz).

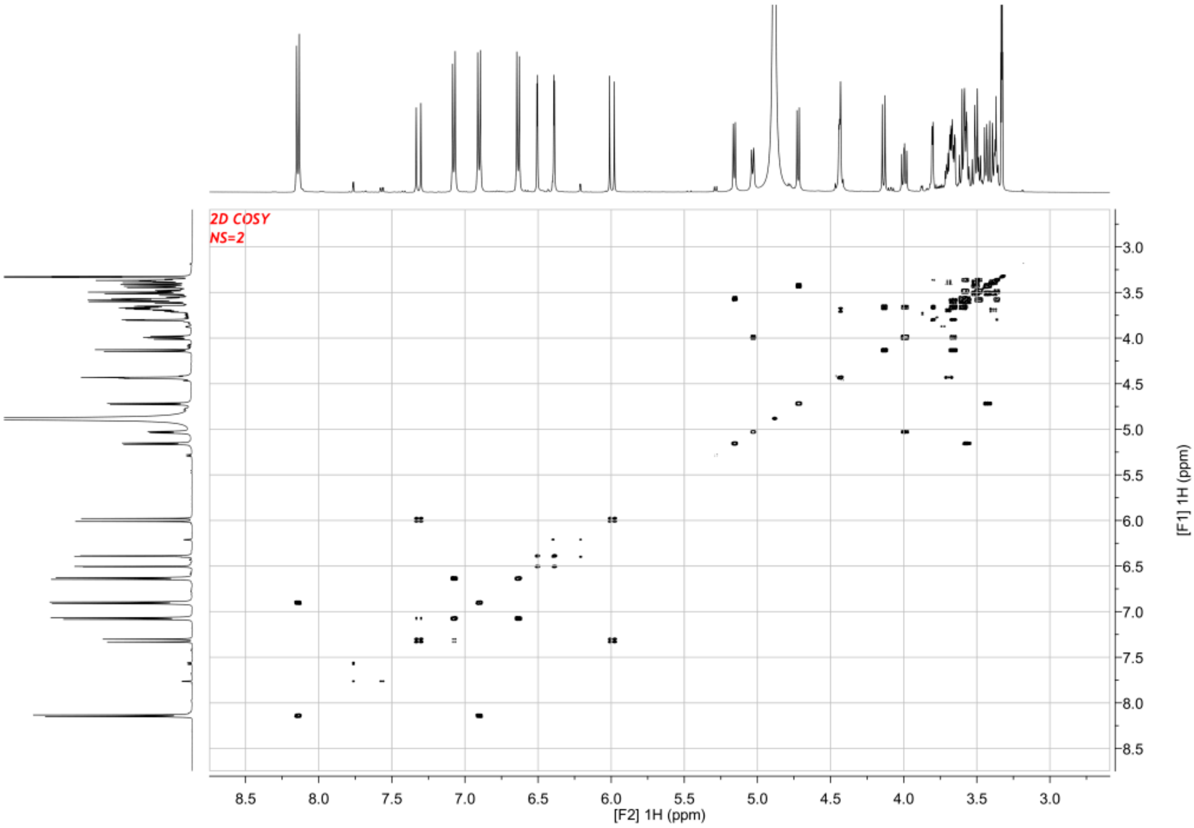

Figure S90. 2D TOCSY NMR spectrum of compound **9** (methanol-d_4, 500.18 MHz).

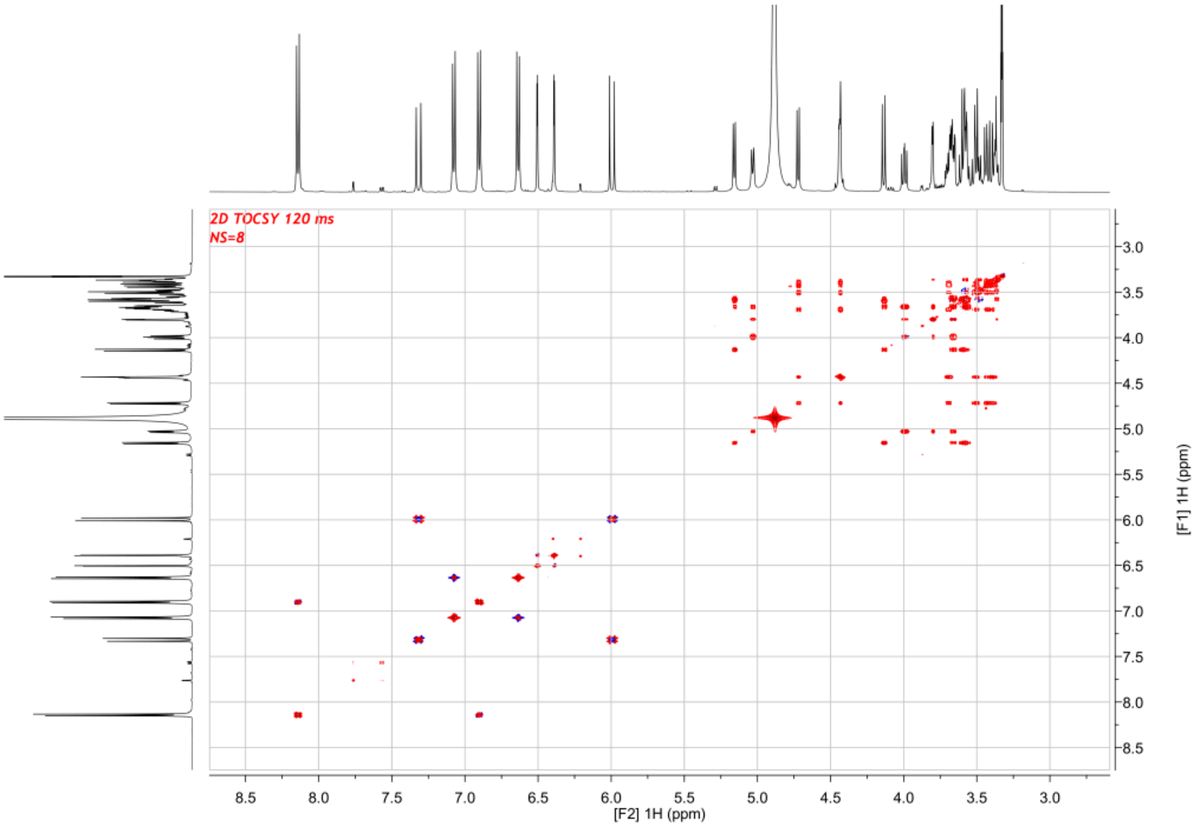

Figure S91. 2D ROESY NMR spectrum of compound **9** (methanol-*d*4, 500.18 MHz).

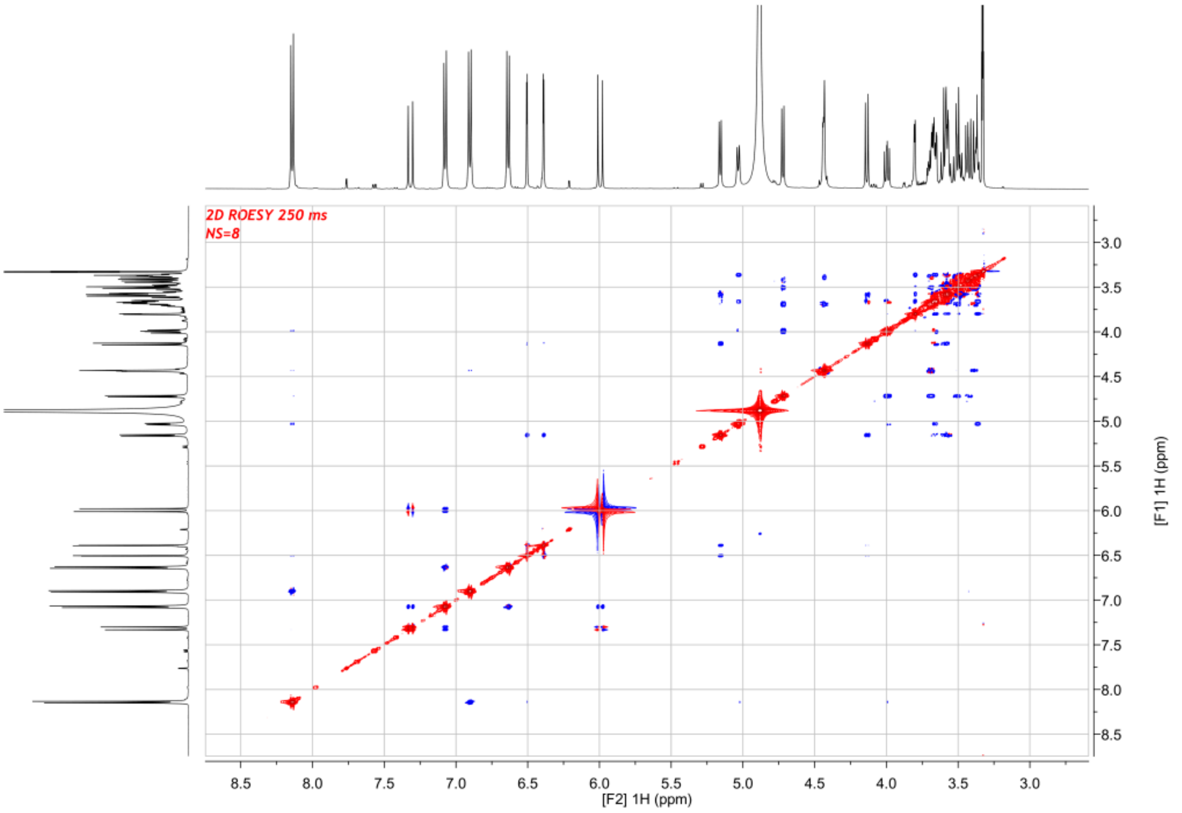

Figure S92. 2D *g*-HSQC-NMR spectrum of compound **9** (methanol-*d*4, 500.18 MHz, 125.77 MHz).

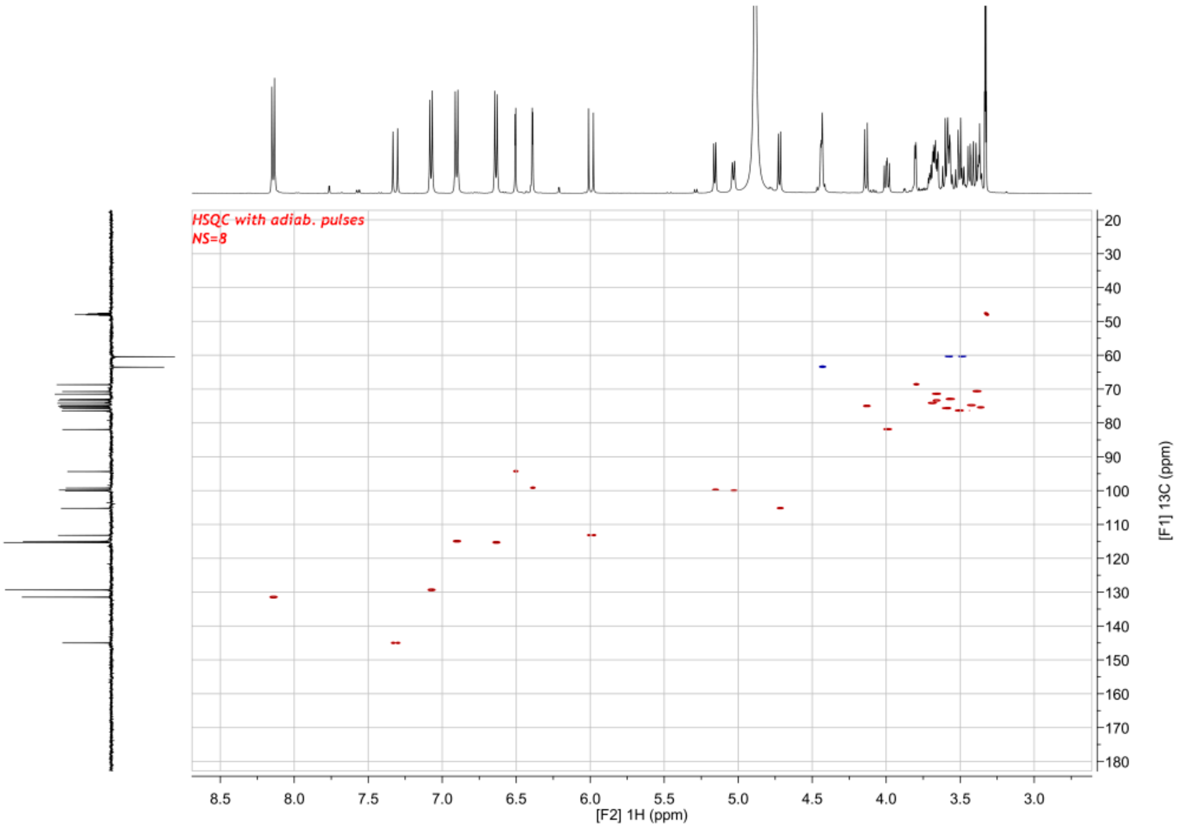

Figure S93. 2D *g*-HSQC-TOCSY NMR spectrum of compound **9** (methanol-d_4, 500.18 MHz, 125.77 MHz).

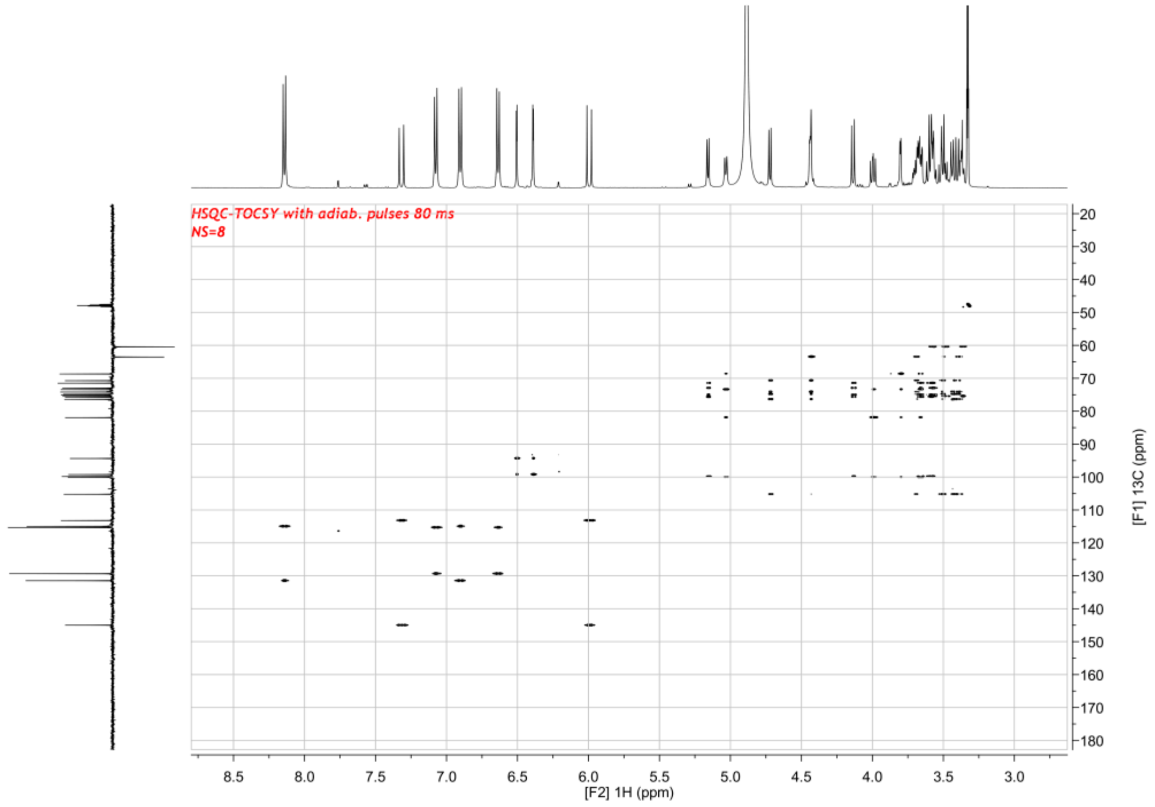

Figure S94. 2D *g*-HMBC-NMR spectrum of compound **9** (methanol-d_4, 500.18 MHz, 125.77 MHz).

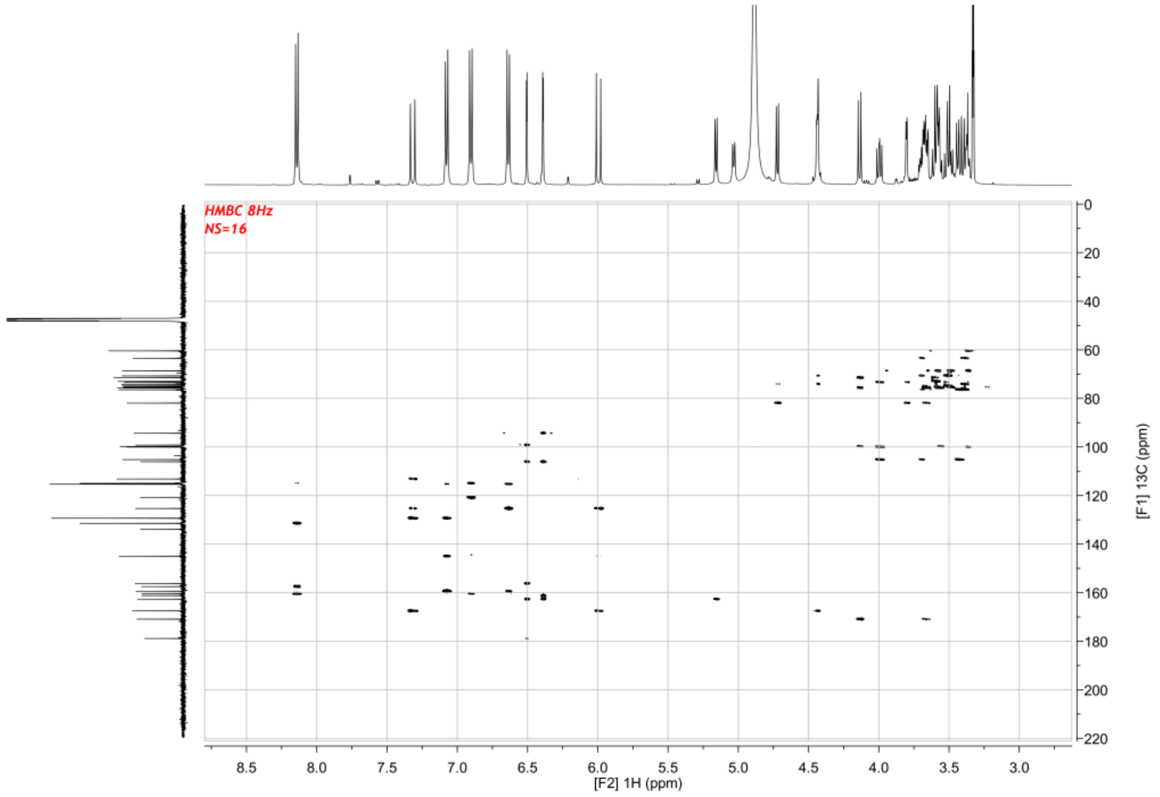

Figure S95. 1D TOCSY and 1D ROESY NMR subspectra of H-1$_{(7\text{-}O\text{-}\beta\text{-GlcA})}$ in compound **9** (methanol-d_4, 500.18 MHz).

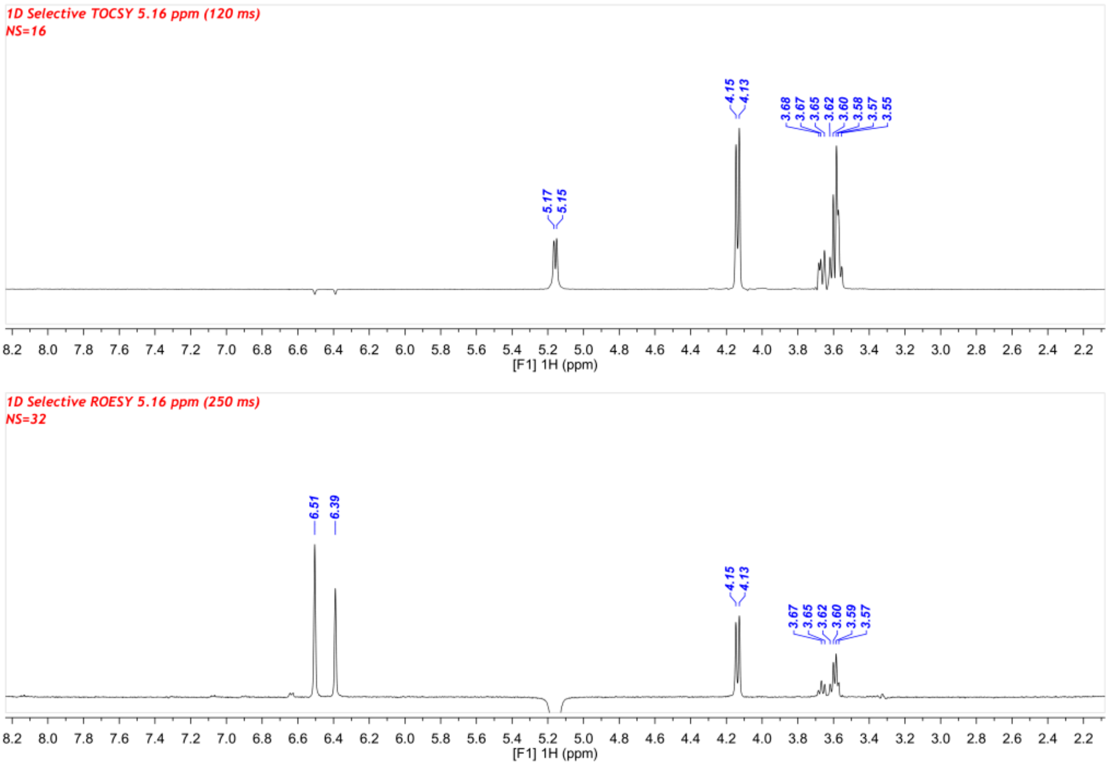

Figure S96. 1D TOCSY and 1D ROESY NMR subspectra of H-1$_{(3\text{-}O\text{-}\beta\text{-Gal})}$ in compound **9** (methanol-d_4, 500.18 MHz).

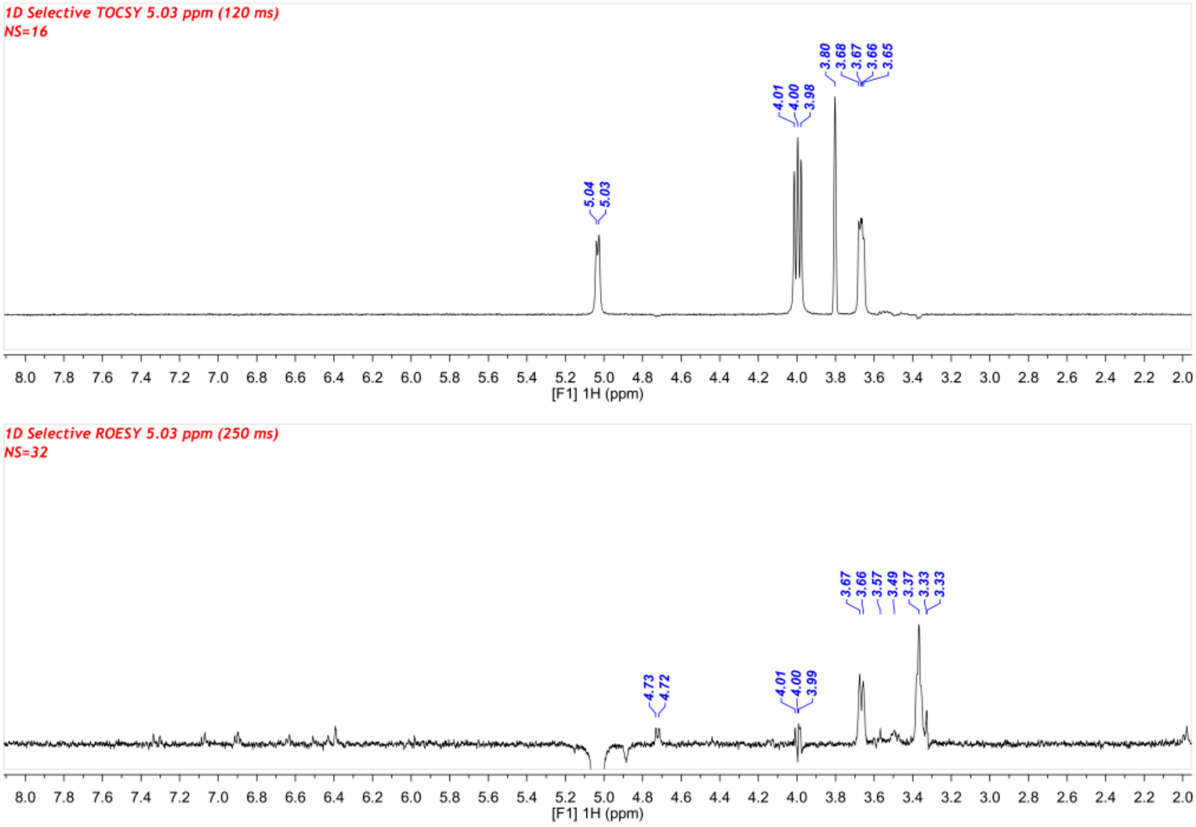

Figure S97. 1D TOCSY and 1D ROESY NMR subspectra of H-1$_{(2^{Gal}\text{-}O\text{-}\beta\text{-Glc})}$ in compound **9** (methanol-d_4, 500.18 MHz).

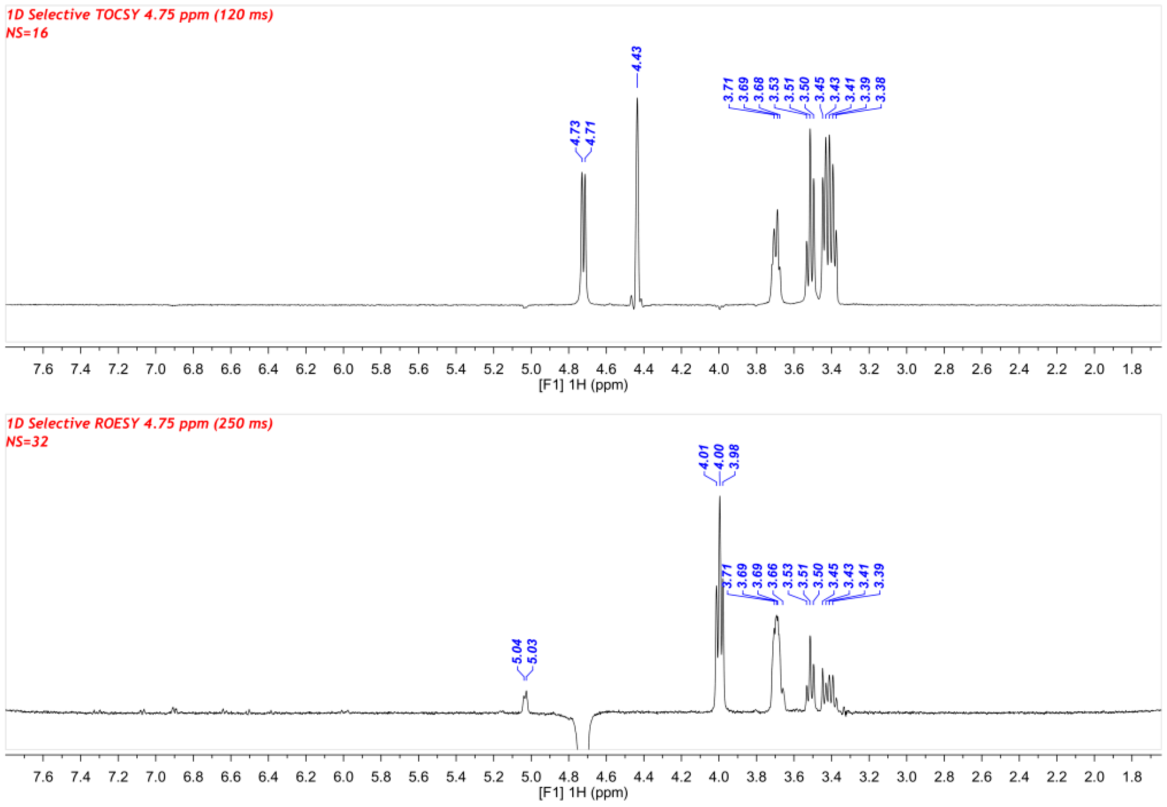

Compound 10

Figure S98. 1D ^{1}H-NMR spectrum of compound **10** (methanol-d_4, 500.18 MHz).

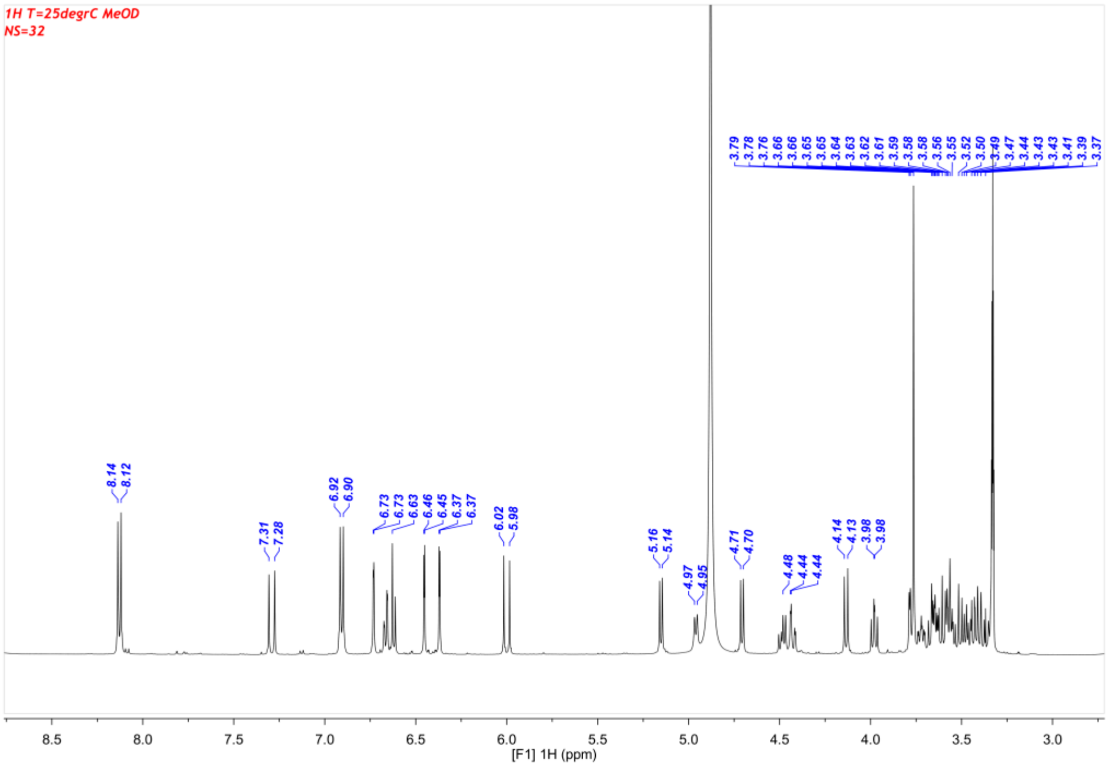

Figure S99. 1D ^{13}C-NMR spectra (1H decoupled and DEPT-135) of compound **10** (methanol-*d*$_4$, 125.77 MHz).

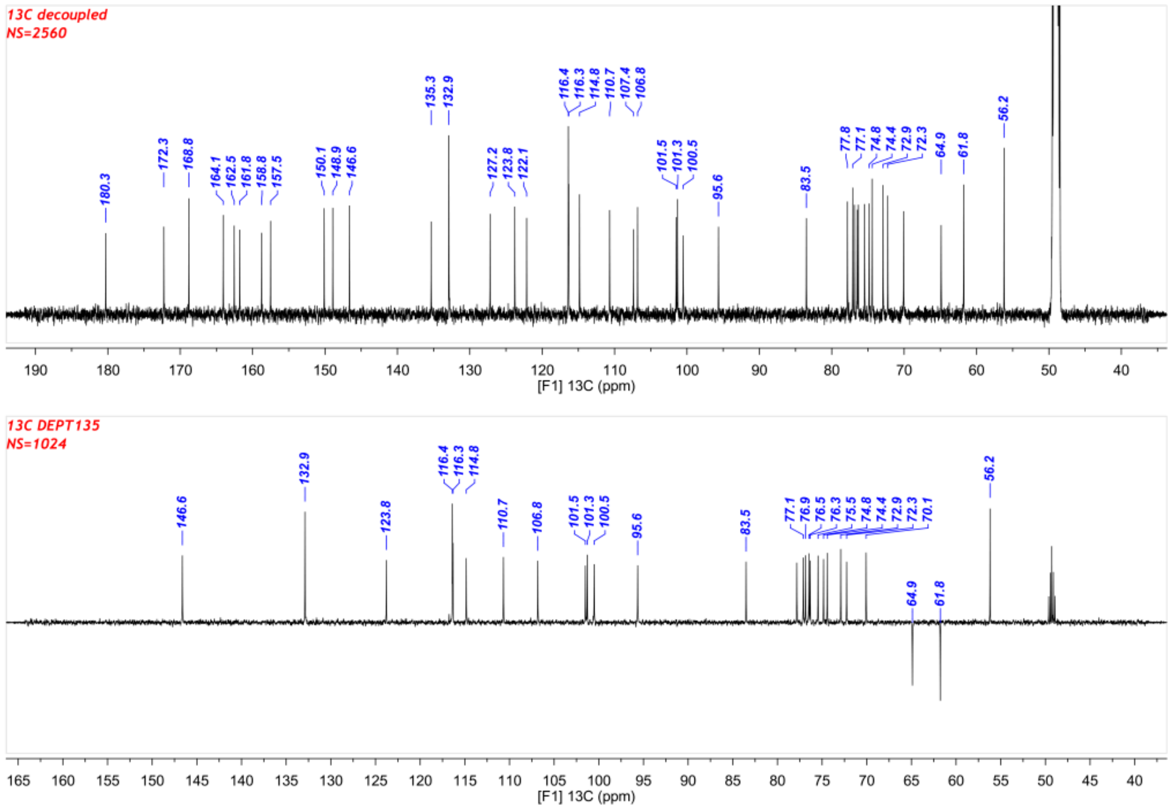

Figure S100. 2D *g*-COSY NMR spectrum of compound **10** (methanol-*d*$_4$, 500.18 MHz).

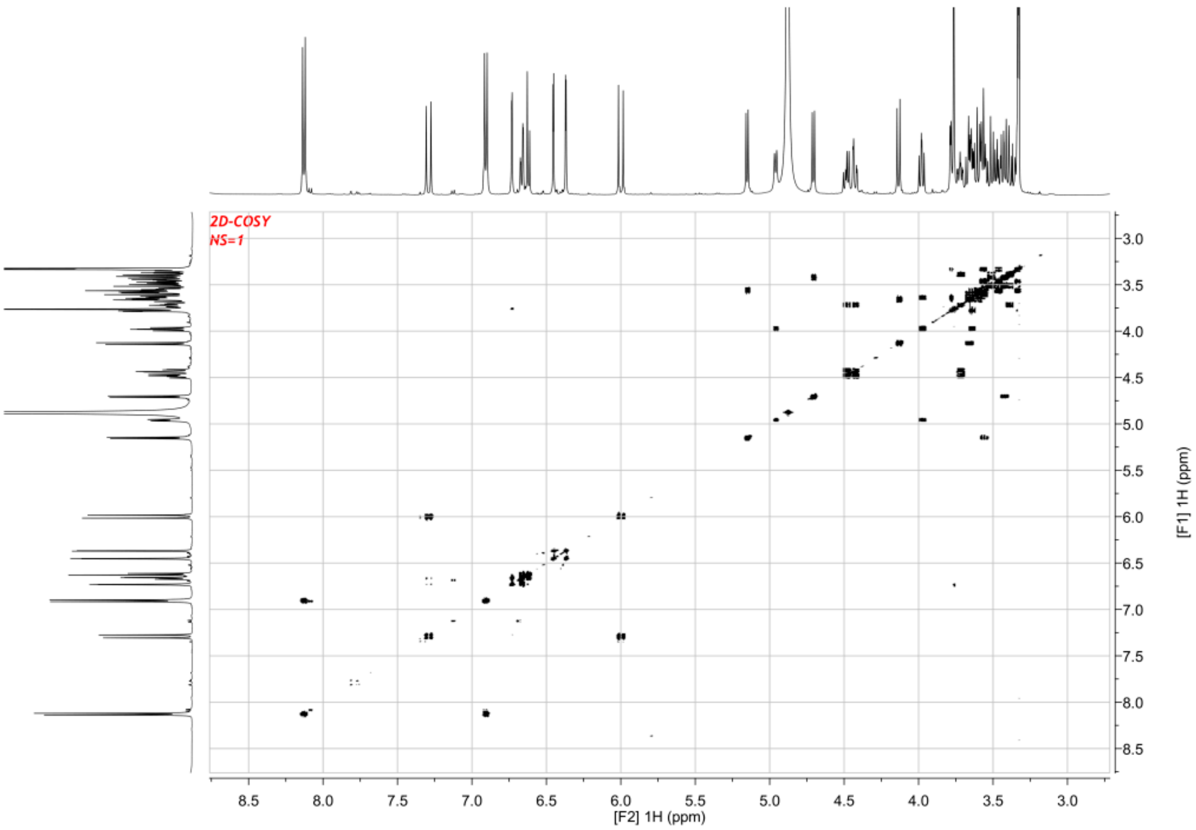

Figure S101. 2D TOCSY NMR spectrum of compound **10** (methanol-d_4, 500.18 MHz).

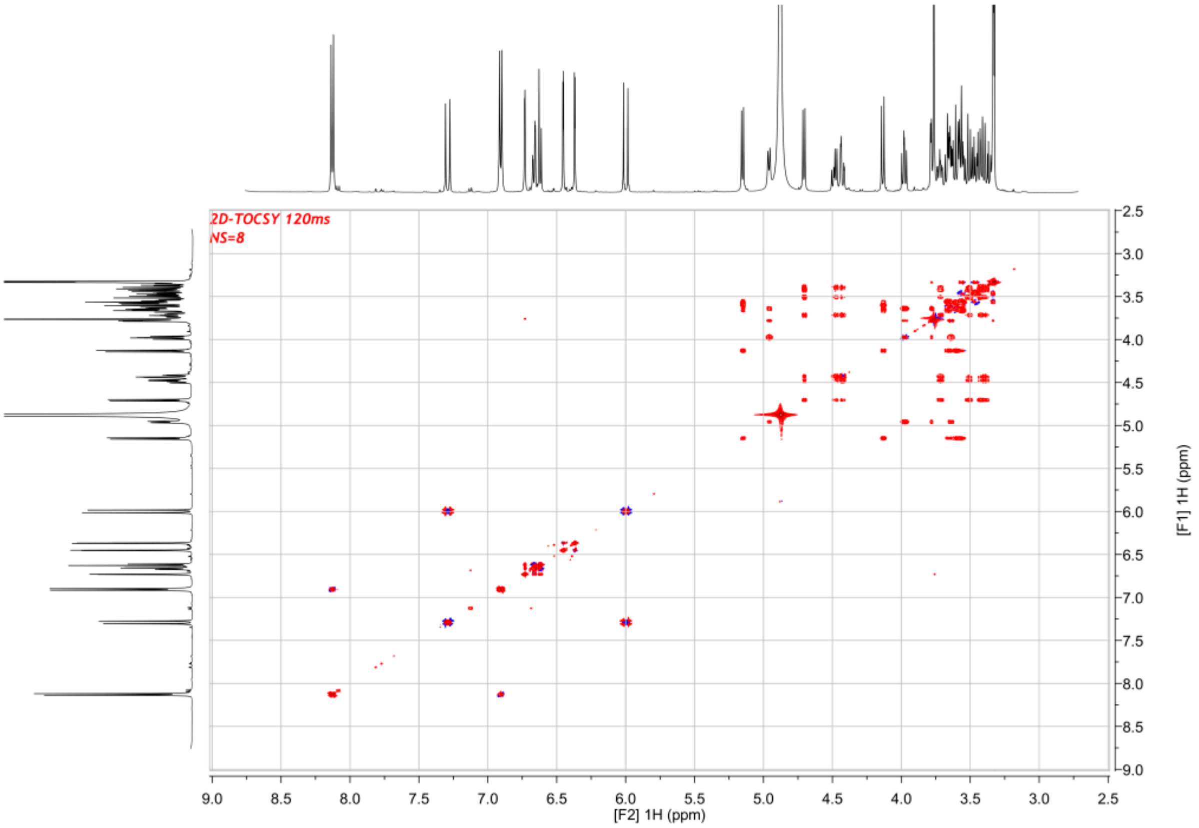

Figure S102. 2D ROESY NMR spectrum of compound **10** (methanol-d_4, 500.18 MHz).

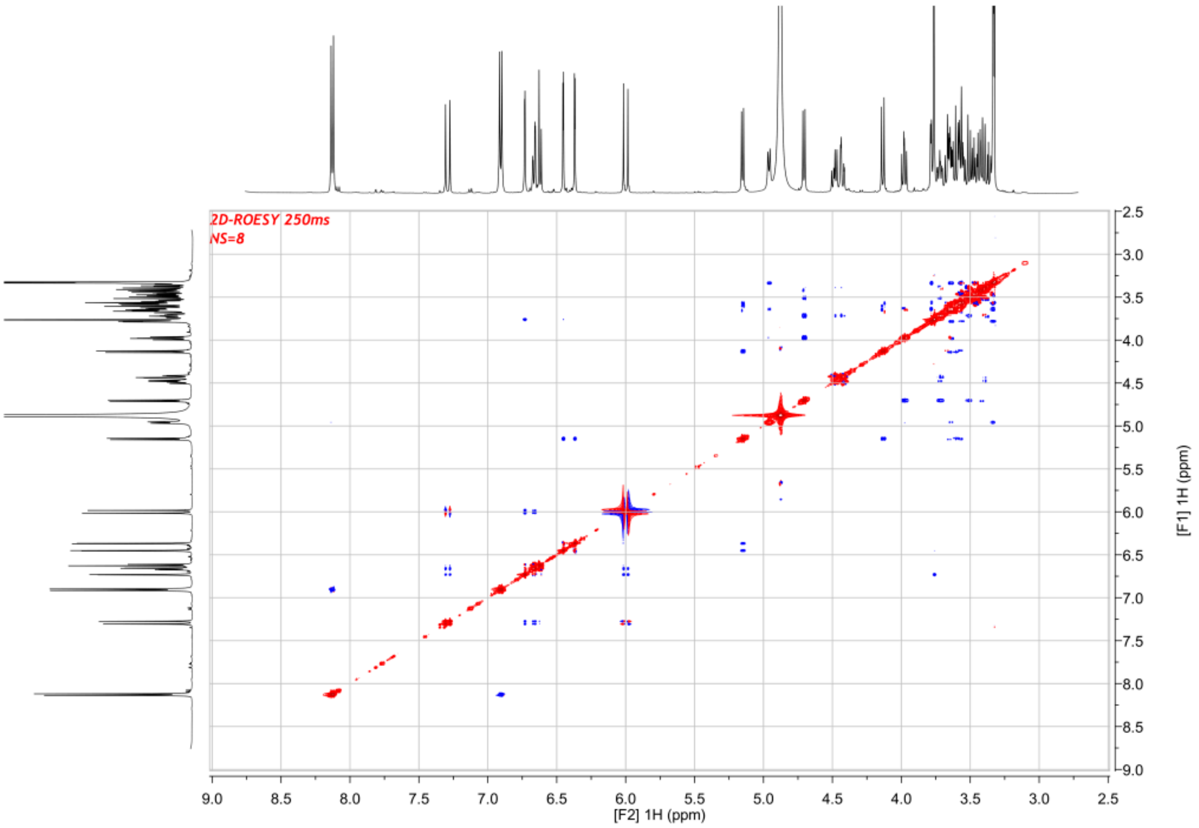

Figure S103. 2D *g*-HSQC-NMR spectrum of compound **10** (methanol-d_4, 500.18 MHz, 125.77 MHz).

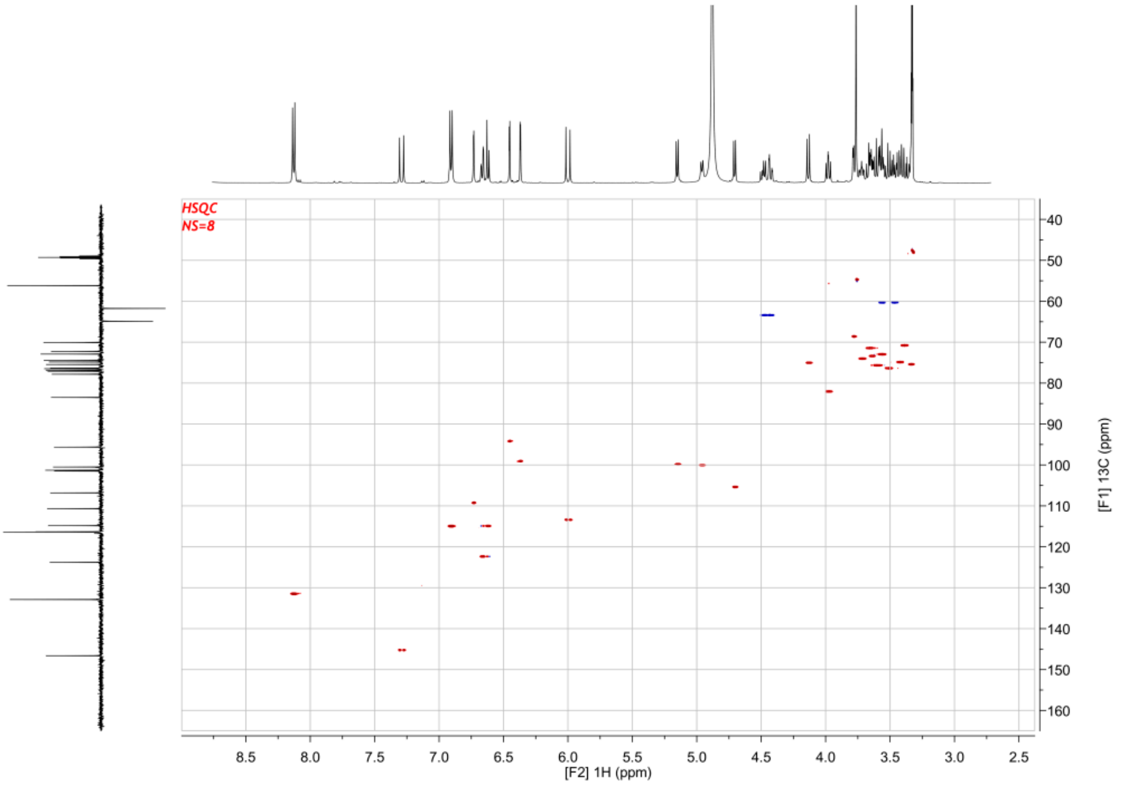

Figure S104. 2D *g*-HSQC-TOCSY NMR spectrum of compound **10** (methanol-d_4, 500.18 MHz, 125.77 MHz).

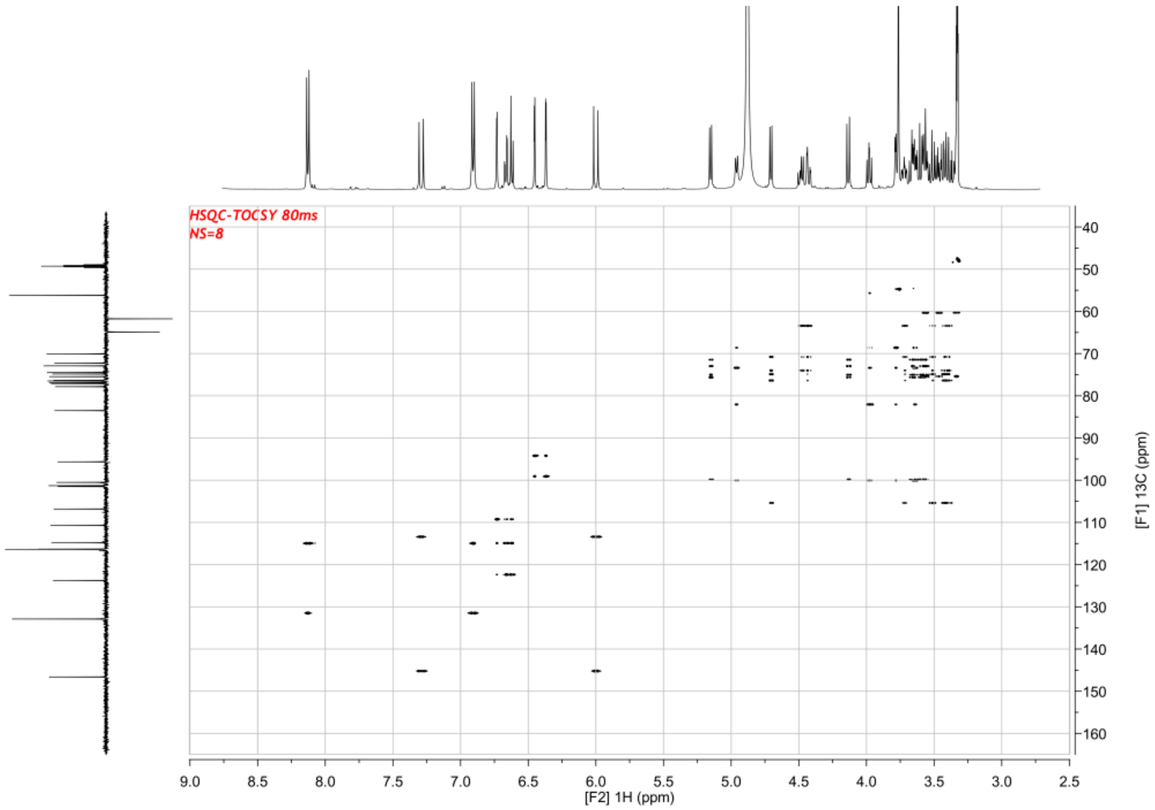

Figure S105. 2D *g*-HMBC-NMR spectrum of compound **10** (methanol-d_4, 500.18 MHz, 125.77 MHz).

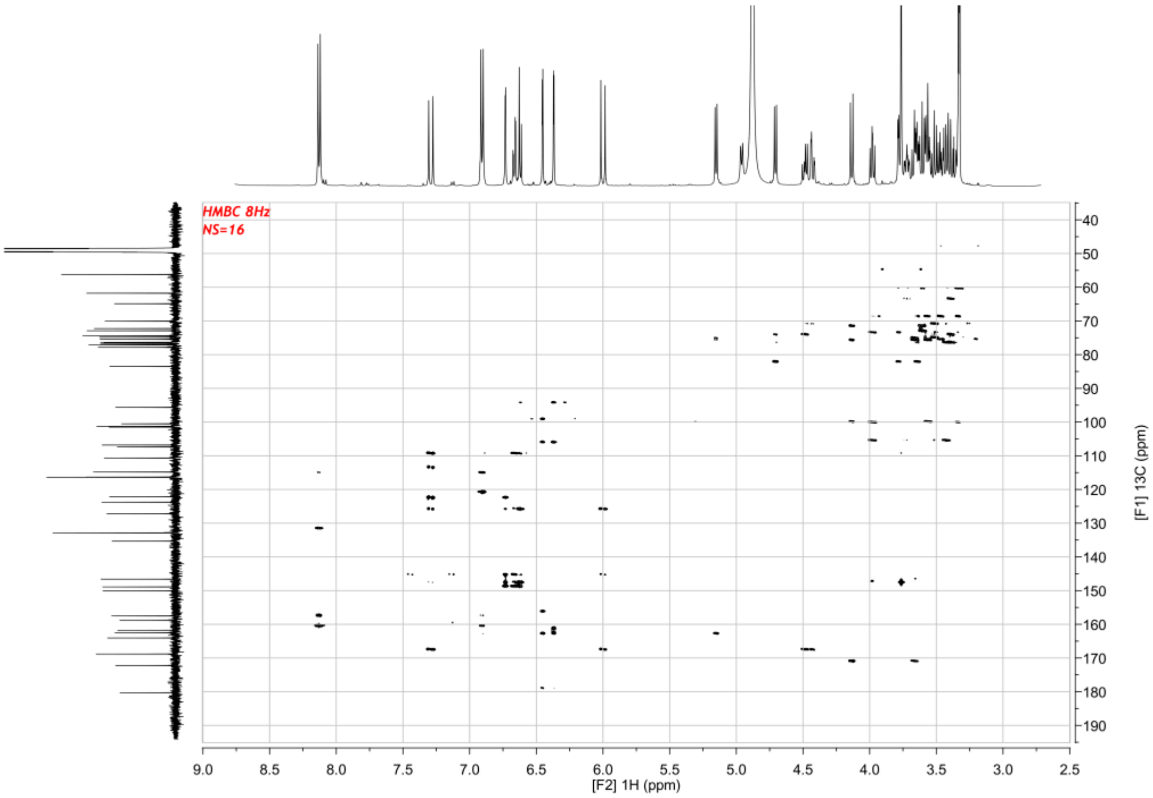

Figure S106. 1D TOCSY and 1D ROESY NMR subspectra of H-1$_{(7\text{-}O\text{-}\beta\text{-GlcA})}$ in compound **10** (methanol-d_4, 500.18 MHz).

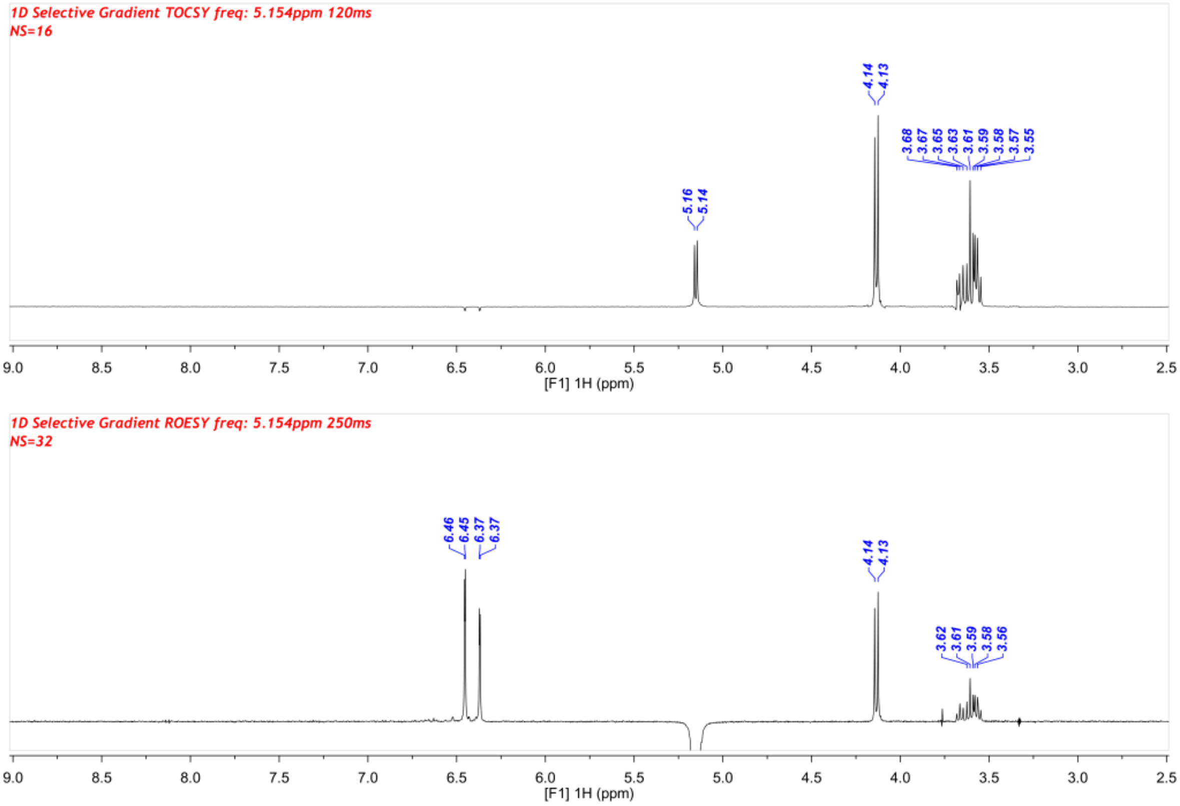

Figure S107. 1D TOCSY and 1D ROESY NMR subspectra of H-1(3-*O*-β-Gal) in compound **10** (methanol-*d*4, 500.18 MHz).

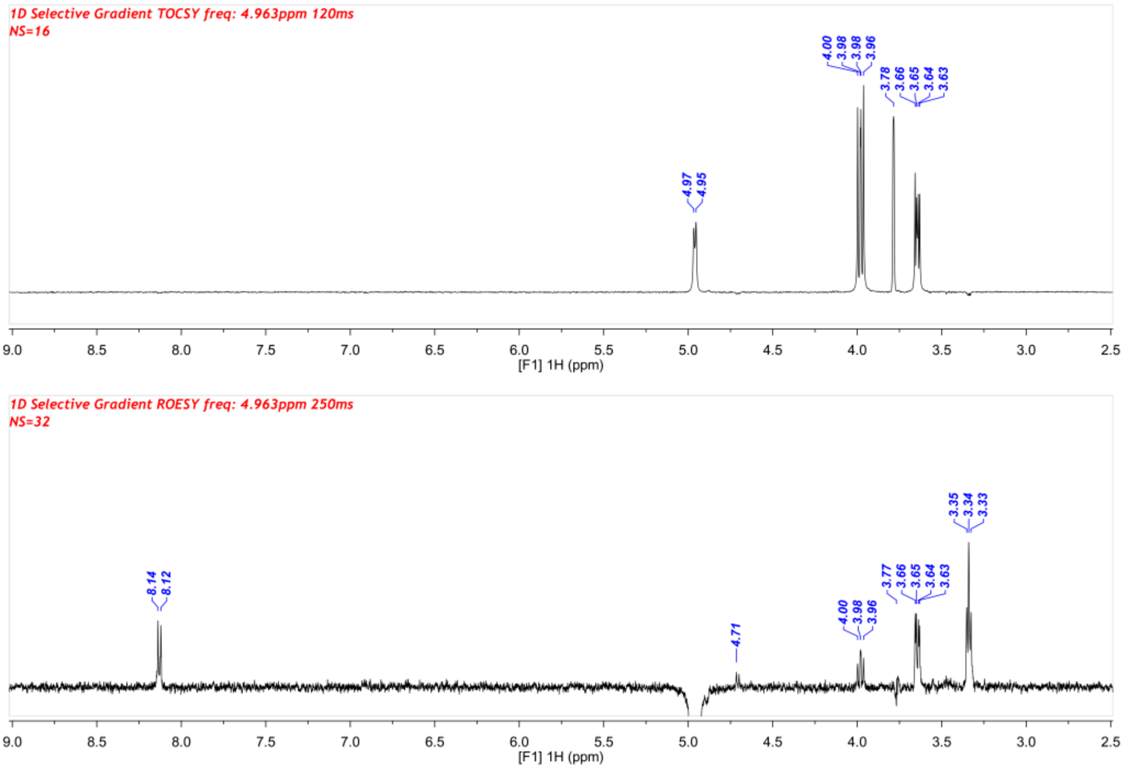

Figure S108. 1D TOCSY and 1D ROESY NMR subspectra of H-1(2Gal-*O*-β-Glc) in compound **10** (methanol-*d*4, 500.18 MHz).

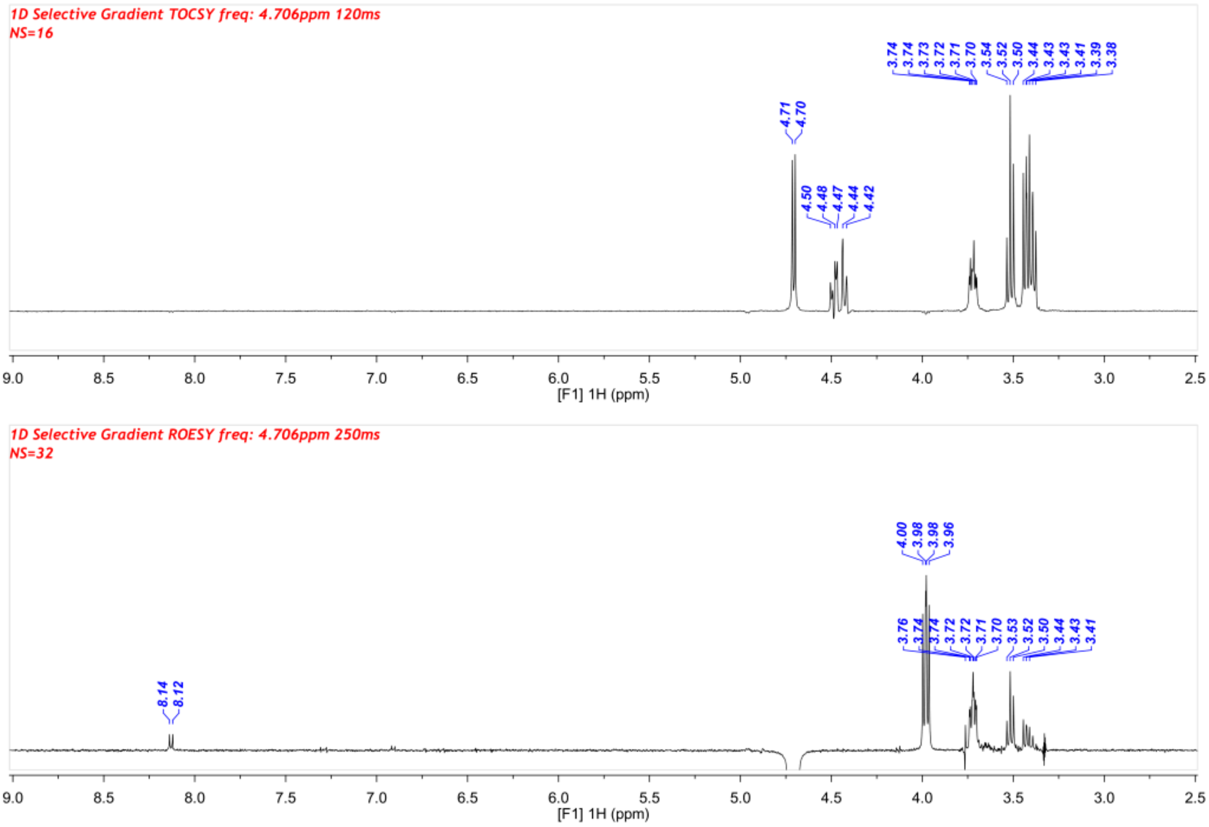

Compound 11

Figure S109. 1D ^{1}H-NMR spectrum of compound **11** (methanol-*d*4, 500.18 MHz).

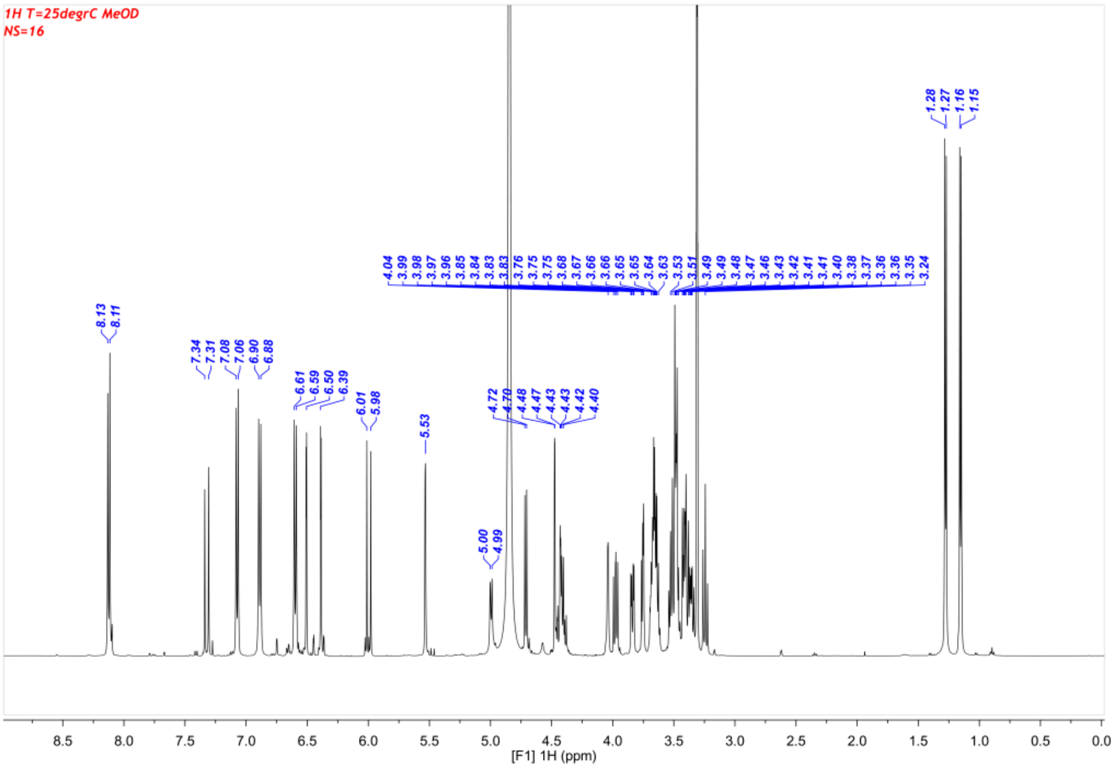

Figure S110. 1D ^{13}C-NMR spectra (1H decoupled and DEPT-135) of compound **11** (methanol-*d*4, 125.77 MHz).

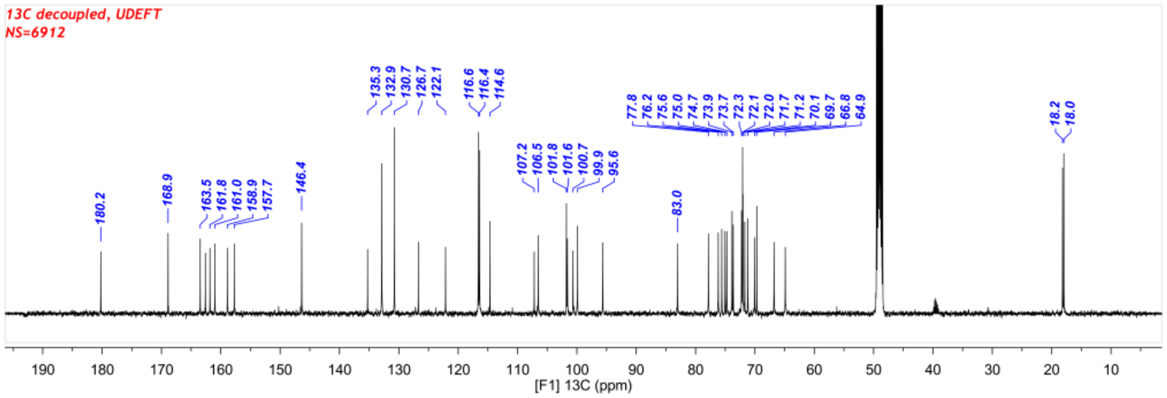

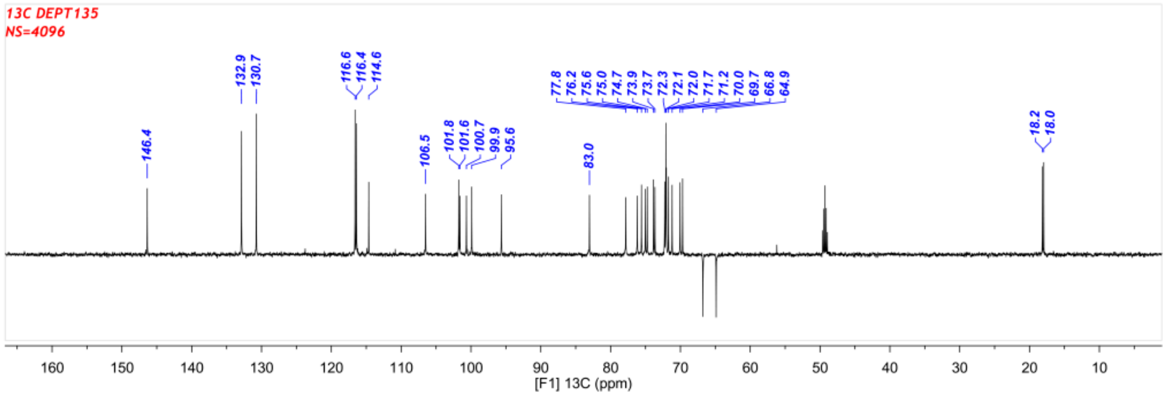

Figure S111. 2D *g*-COSY NMR spectrum of compound **11** (methanol-*d*4, 500.18 MHz).

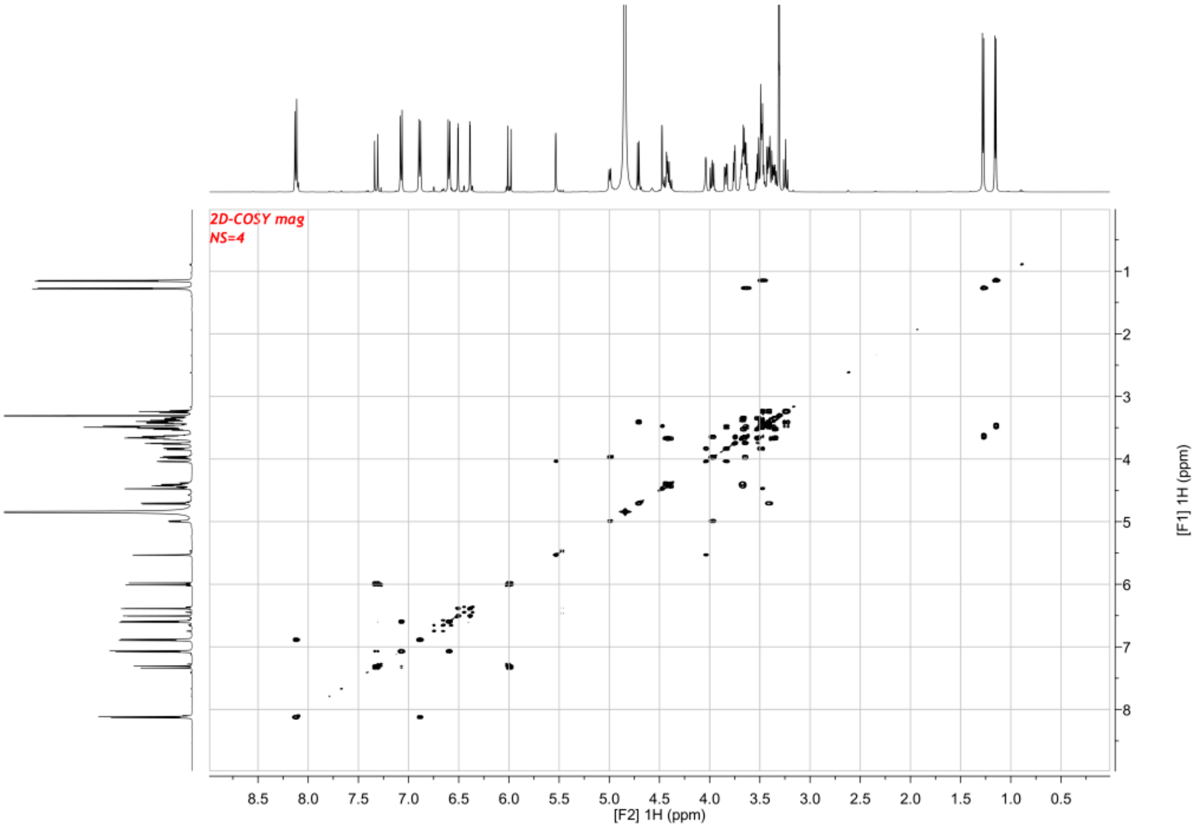

Figure S112. 2D TOCSY NMR spectrum of compound **11** (methanol-*d*4, 500.18 MHz).

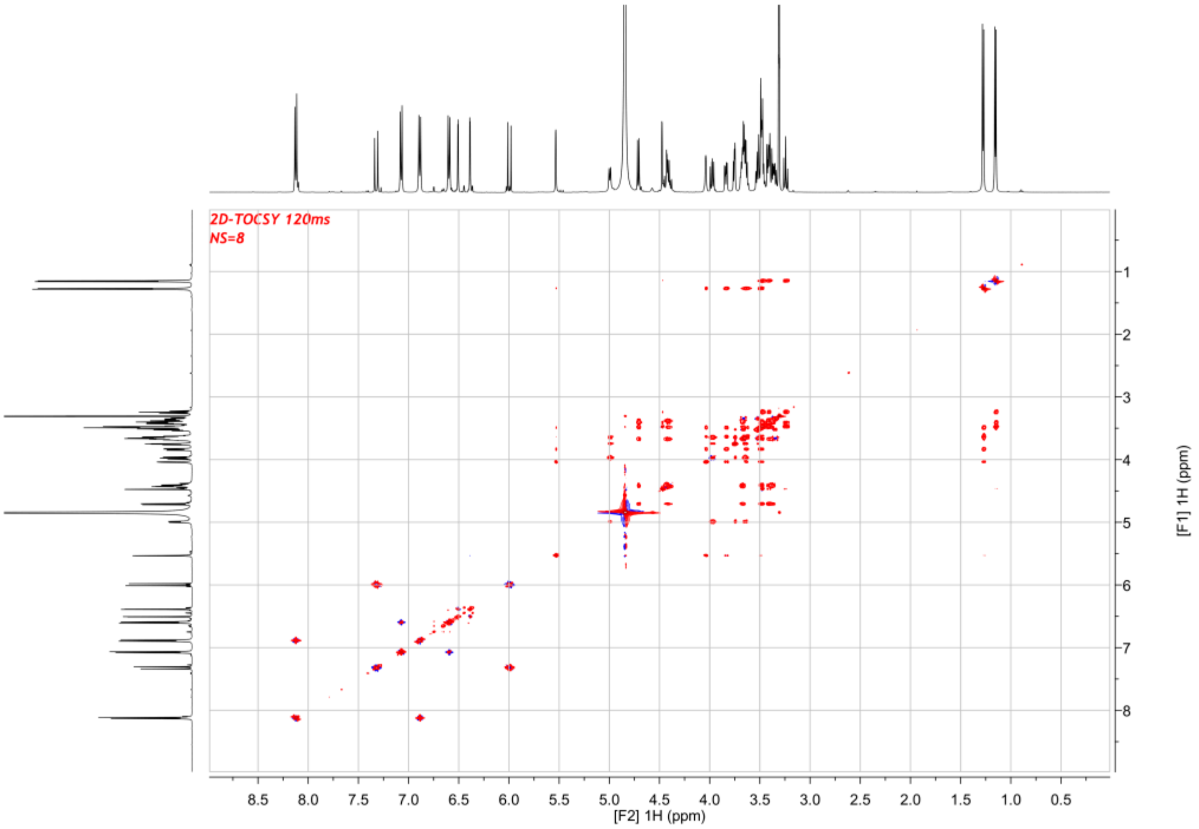

Figure S113. 2D ROESY NMR spectrum of compound **11** (methanol-*d*4, 500.18 MHz).

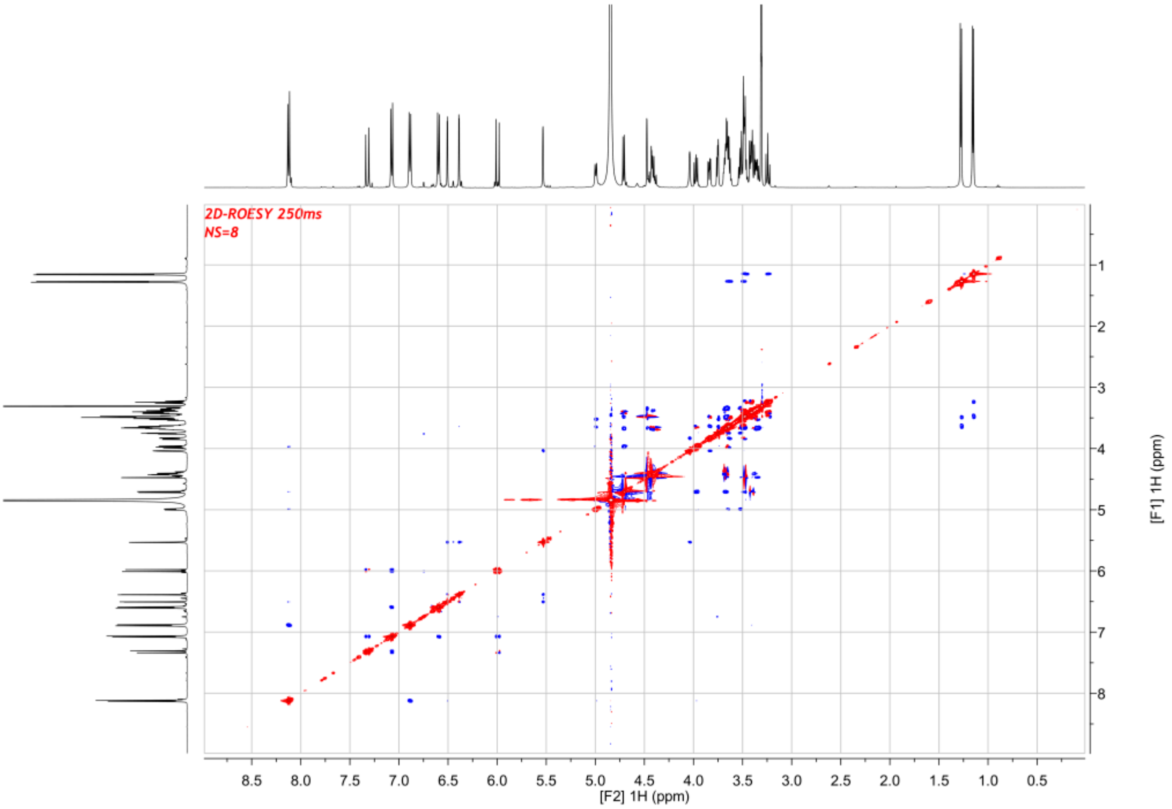

Figure S114. 2D *g*-HSQC-NMR spectrum of compound **11** (methanol-*d*4, 500.18 MHz, 125.77 MHz).

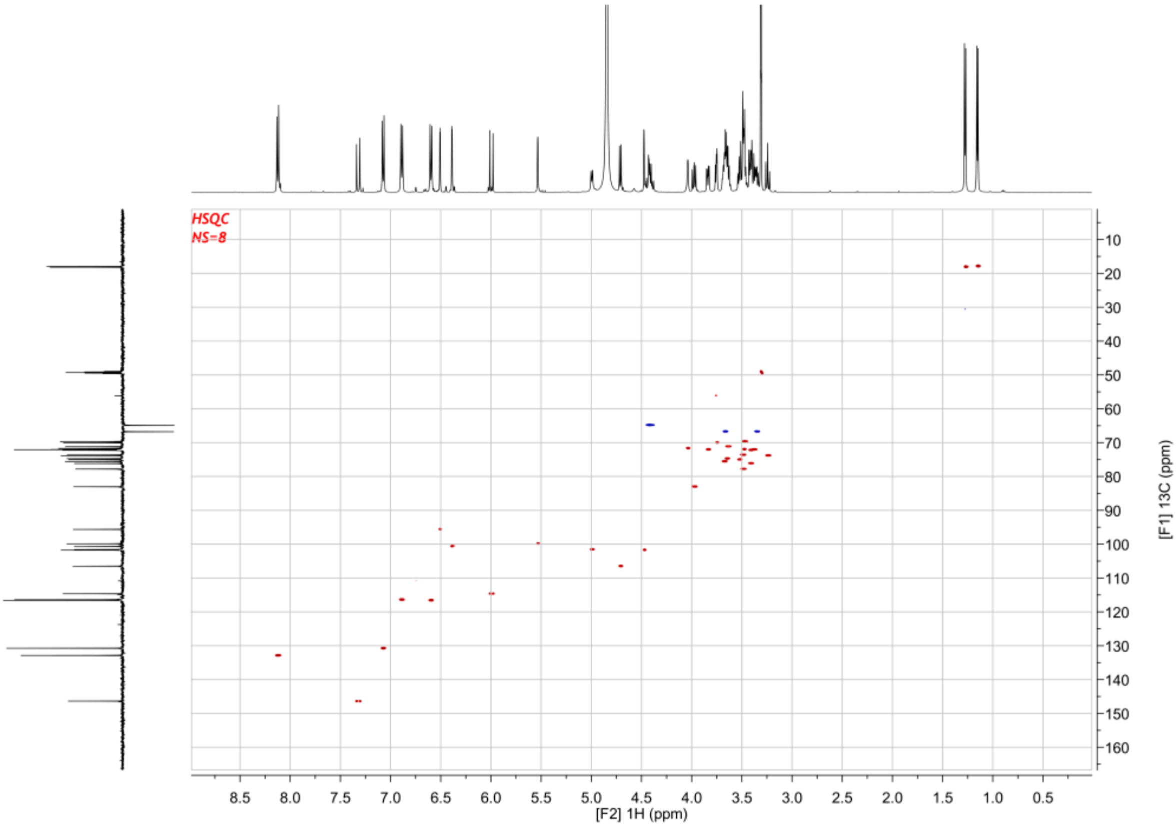

Figure S115. 2D *g*-HSQC-TOCSY NMR spectrum of compound **11** (methanol-*d*4, 500.18 MHz, 125.77 MHz).

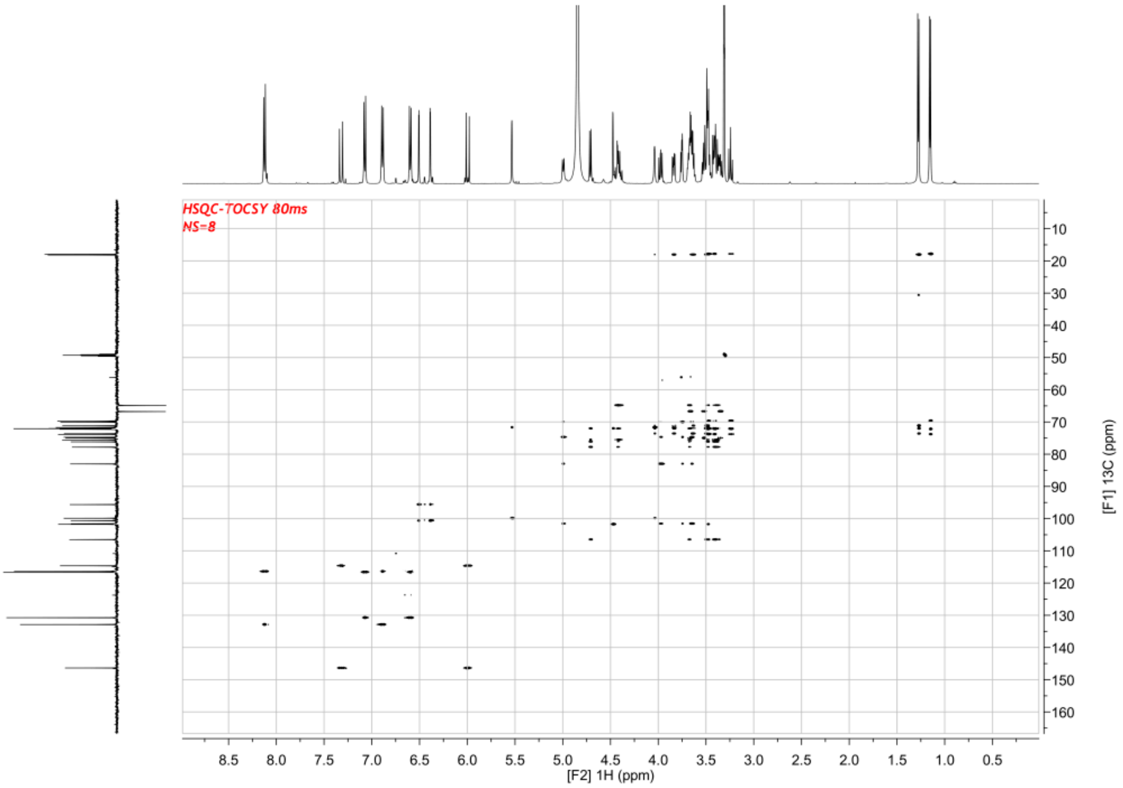

Figure S116. 2D *g*-HMBC-NMR spectrum of compound **11** (methanol-*d*4, 500.18 MHz, 125.77 MHz).

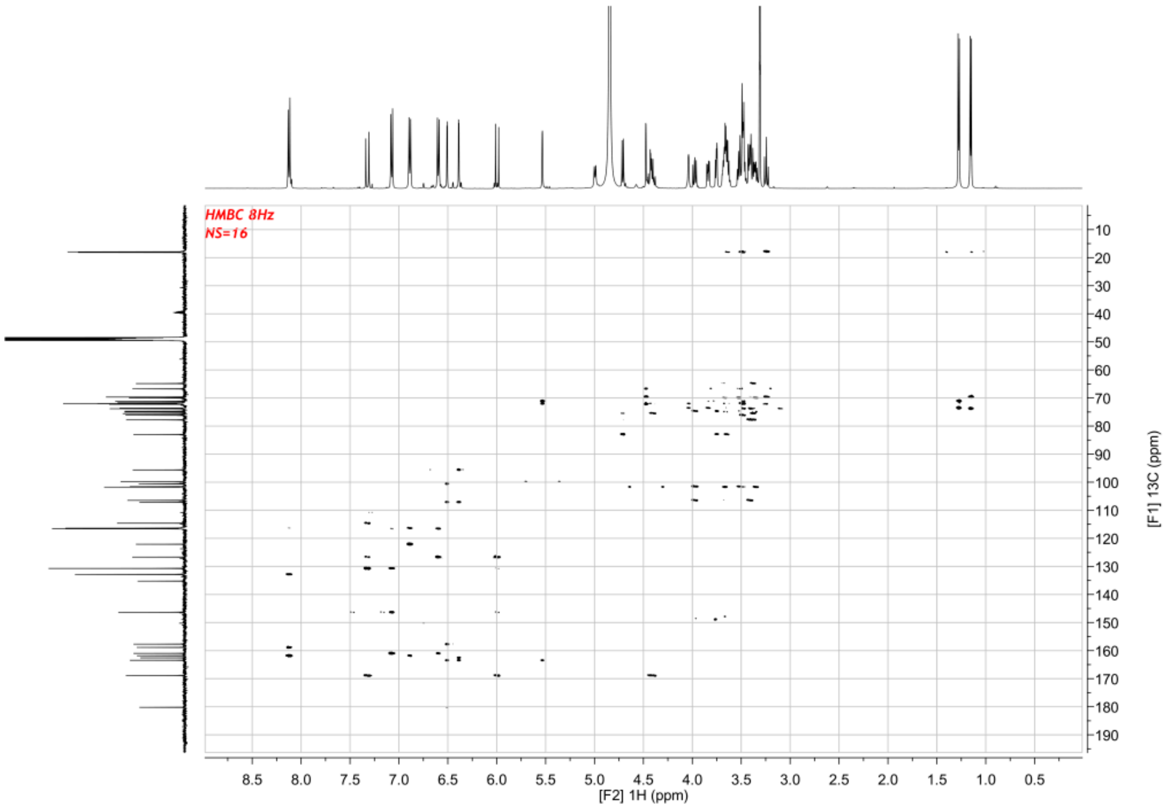

Figure S117. 1D TOCSY and 1D ROESY NMR subspectra of H-1$_{(7\text{-}O\text{-}\alpha\text{-Rha})}$ and H-6$_{(7\text{-}O\text{-}\alpha\text{-Rha})}$ in compound **11** (methanol-d_4, 500.18 MHz).

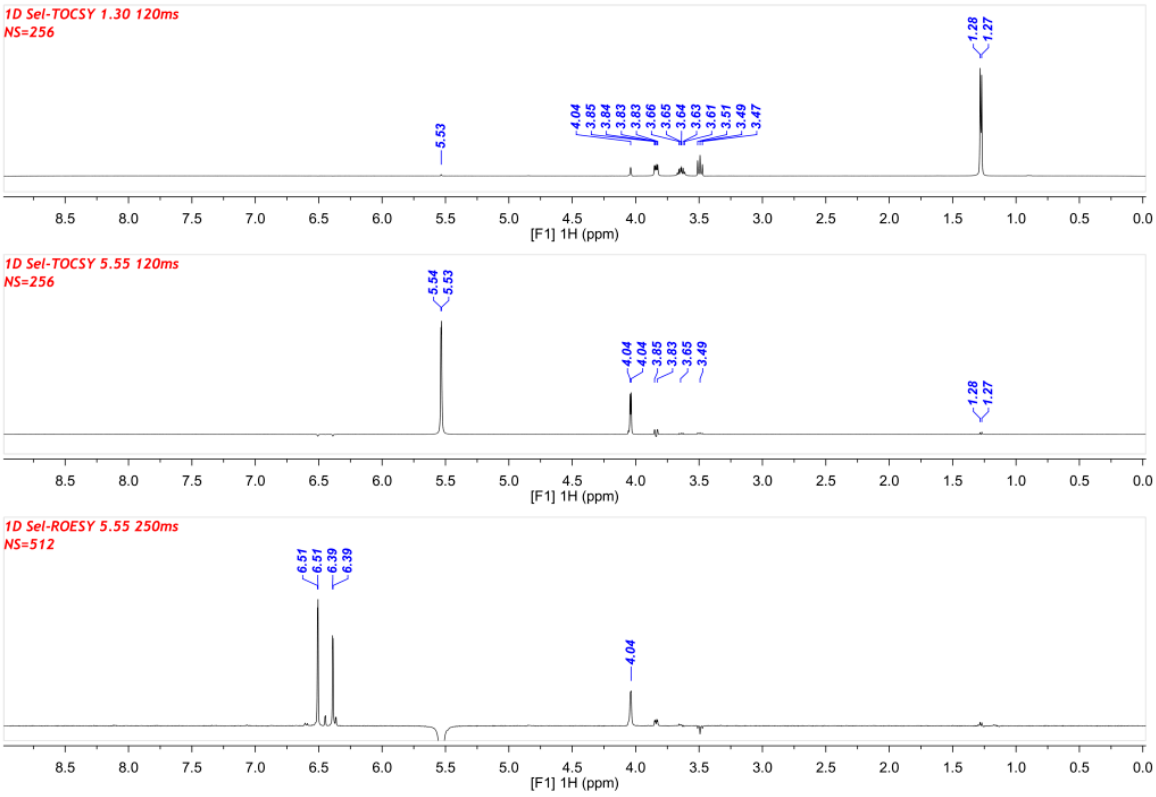

Figure S118. 1D TOCSY and 1D ROESY NMR subspectra of H-1$_{(3\text{-}O\text{-}\beta\text{-Gal})}$ in compound **11** (methanol-d_4, 500.18 MHz).

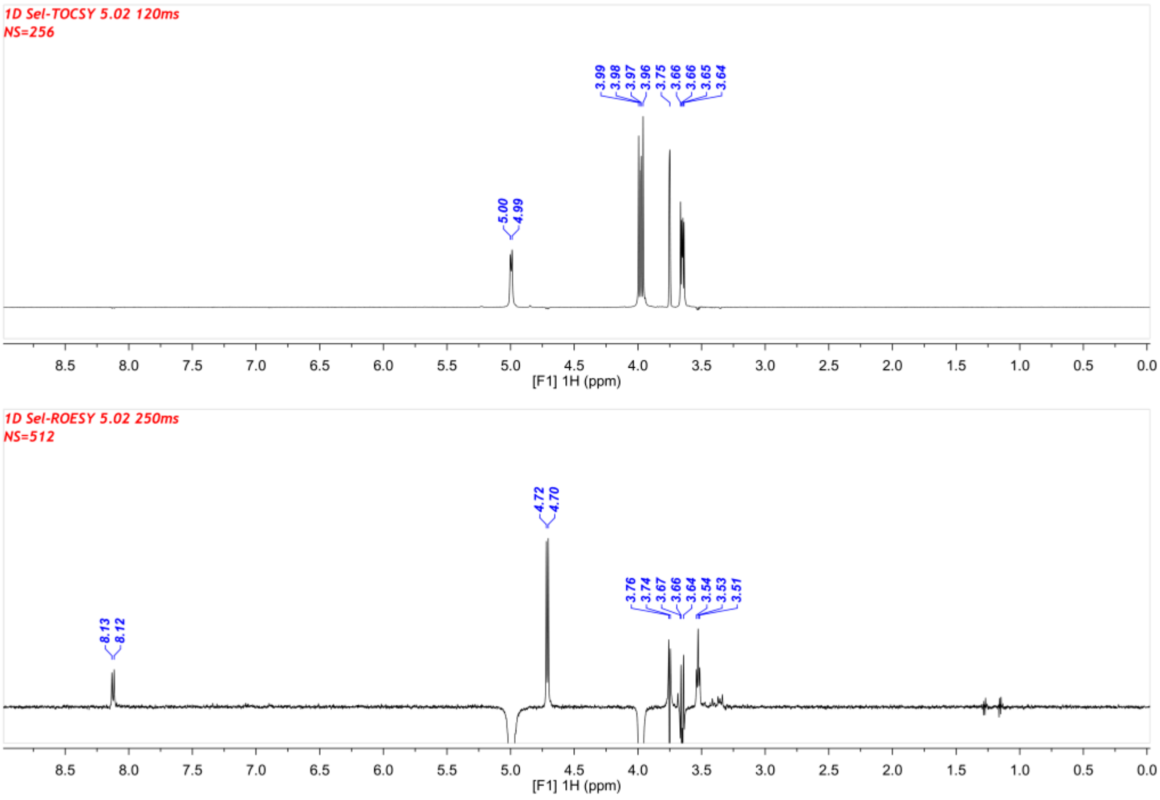

Figure S119. 1D TOCSY and 1D ROESY NMR subspectra of H-$1_{(2^{Gal}\text{-}O\text{-}\beta\text{-Glc})}$ in compound **11** (methanol-d_4, 500.18 MHz).

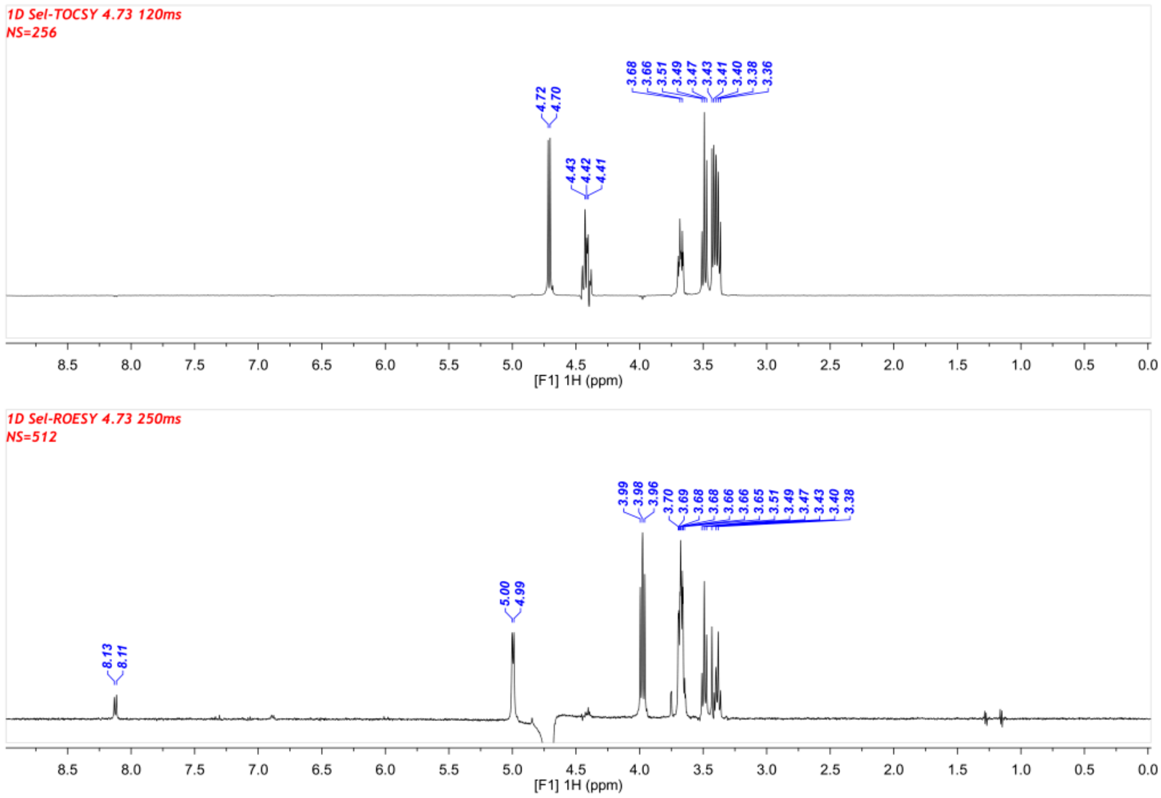

Figure S120. 1D TOCSY and 1D ROESY NMR subspectra of H-$1_{(6^{Gal}\text{-}O\text{-}\alpha\text{-Rha})}$ and H-$6_{(6^{Gal}\text{-}O\text{-}\alpha\text{-Rha})}$ in compound **11** (methanol-d_4, 500.18 MHz).

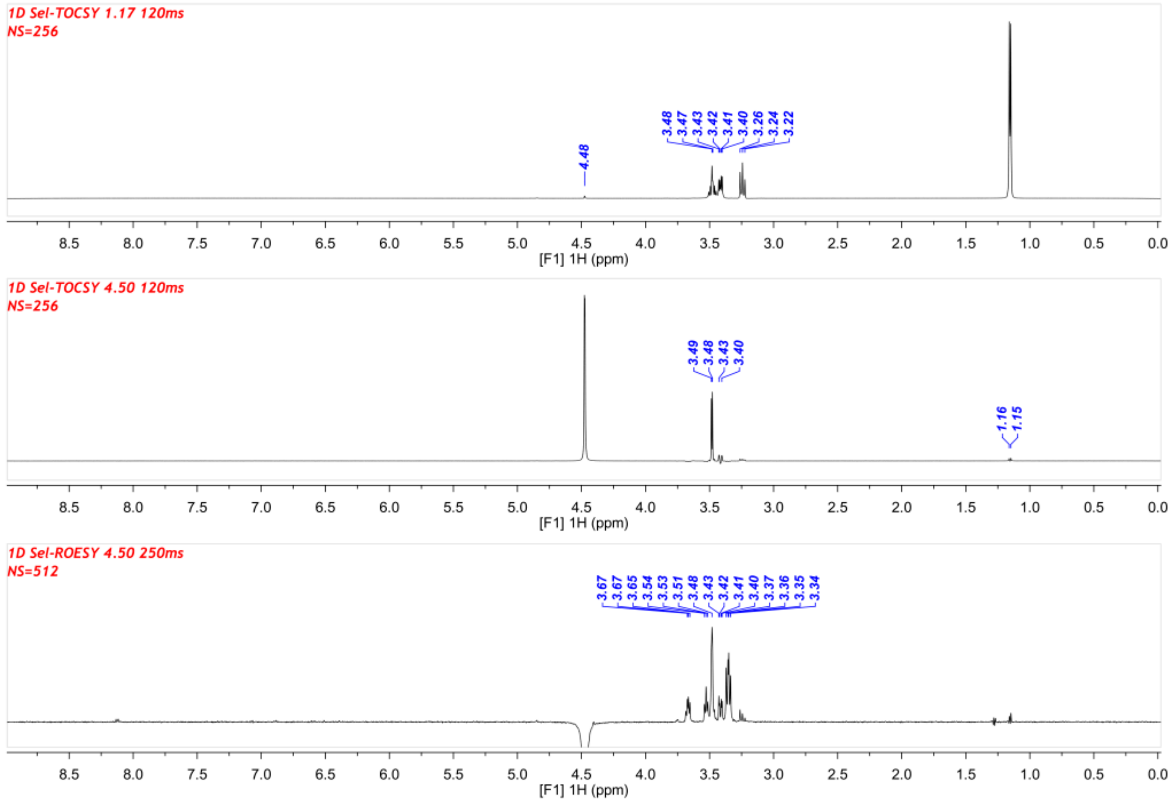

Compound 12

Figure S121. 1D ^{1}H-NMR spectrum of compound **12** (methanol-*d*4, 500.18 MHz).

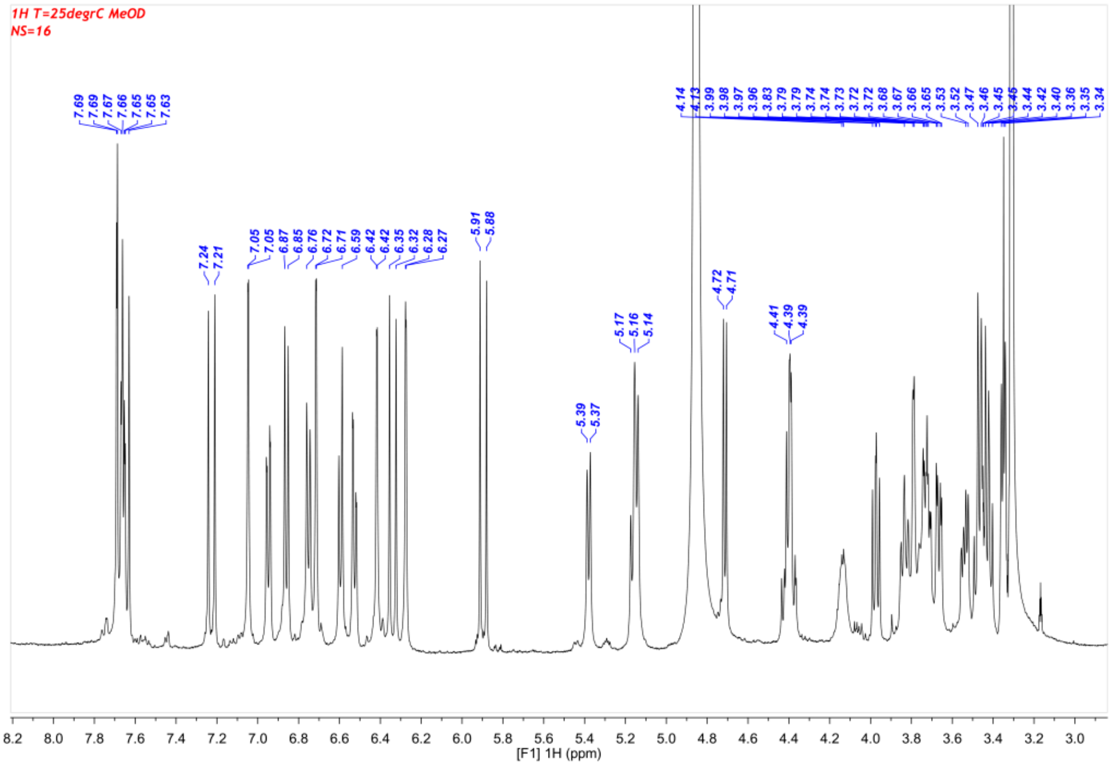

Figure S122. 1D ^{13}C-NMR spectra (1H decoupled and DEPT-135) of compound **12** (methanol-*d*4, 125.77 MHz).

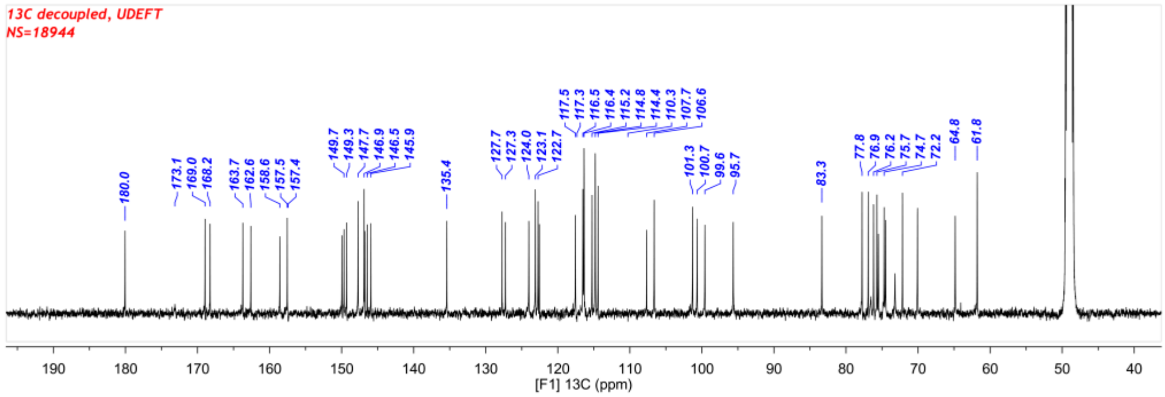

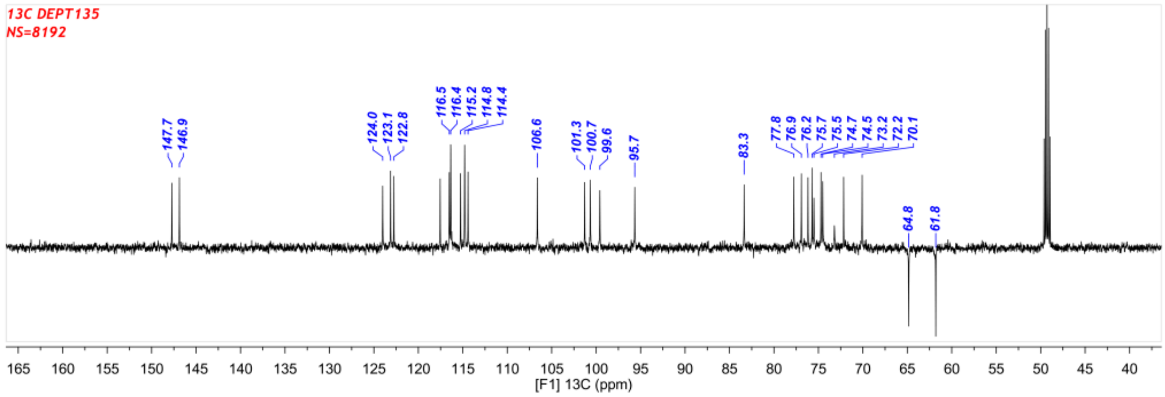

Figure S123. 2D *g*-COSY NMR spectrum of compound **12** (methanol-d_4, 500.18 MHz).

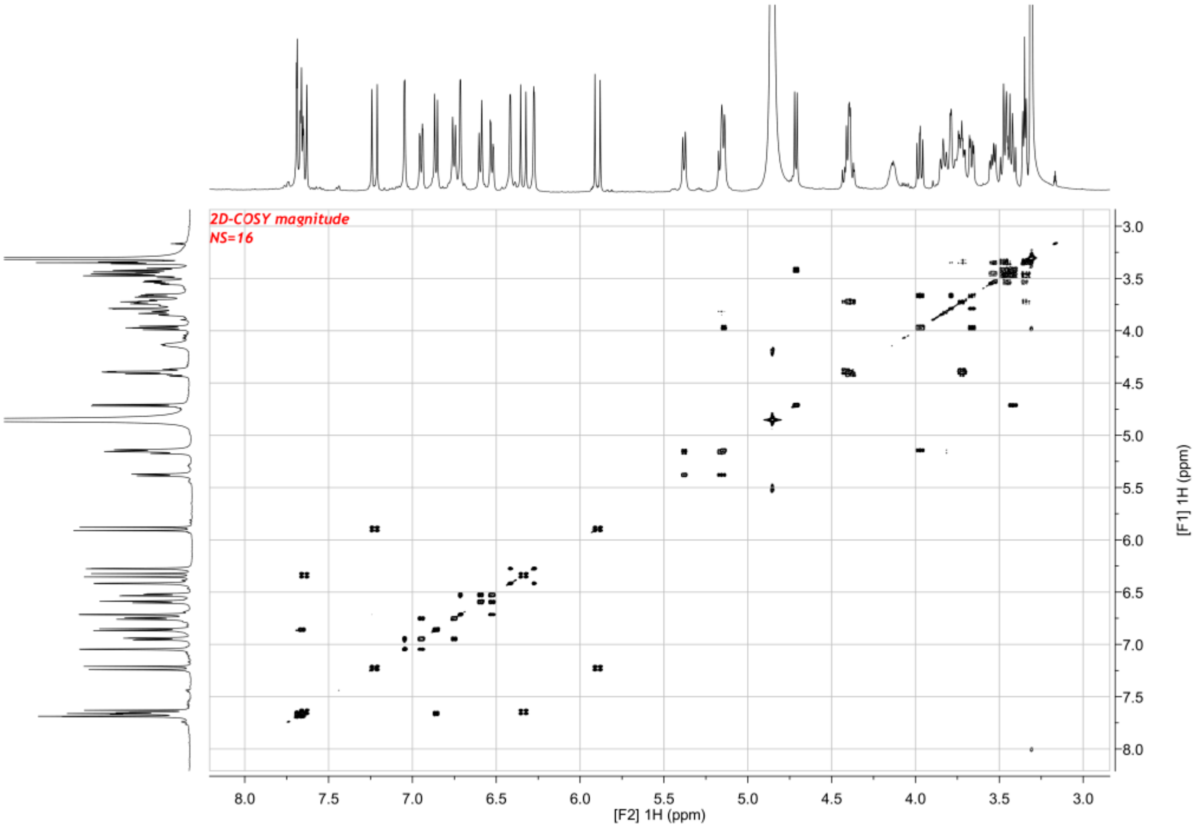

Figure S124. 2D TOCSY NMR spectrum of compound **12** (methanol-d_4, 500.18 MHz).

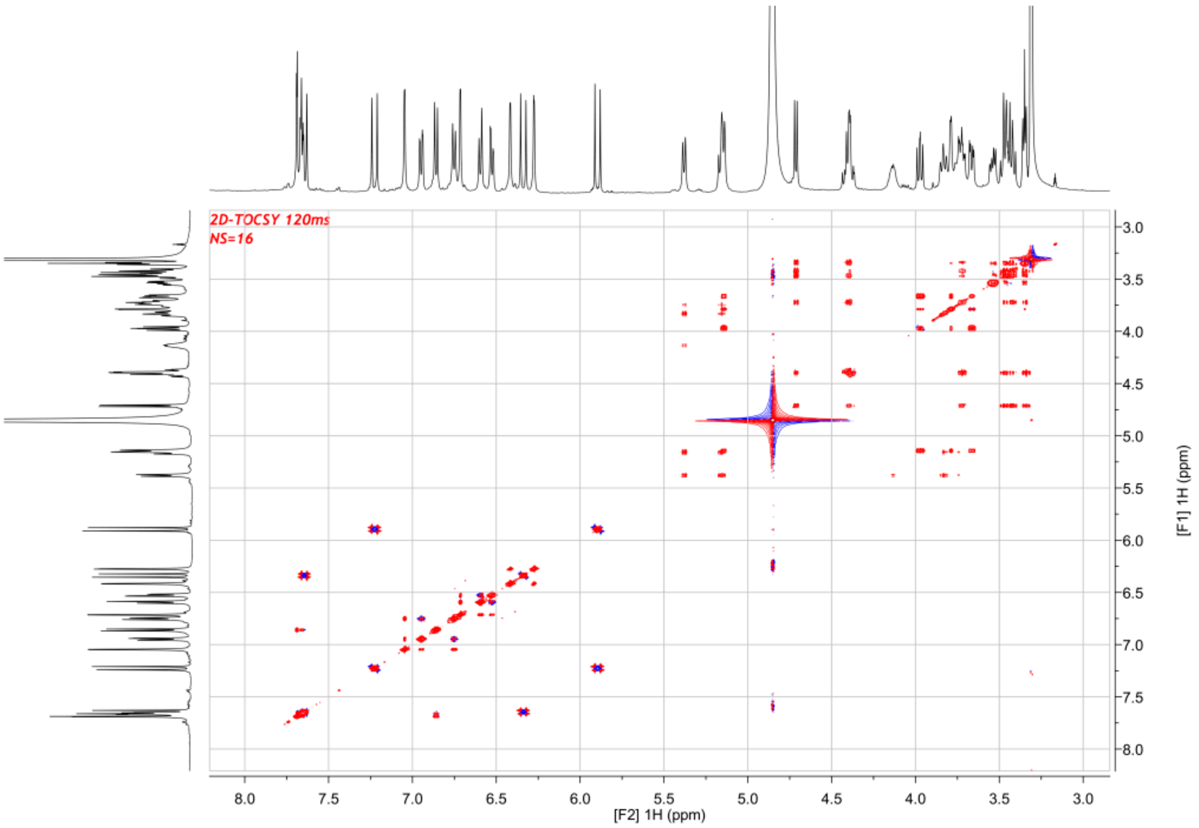

Figure S125. 2D ROESY NMR spectrum of compound **12** (methanol-d_4, 500.18 MHz).

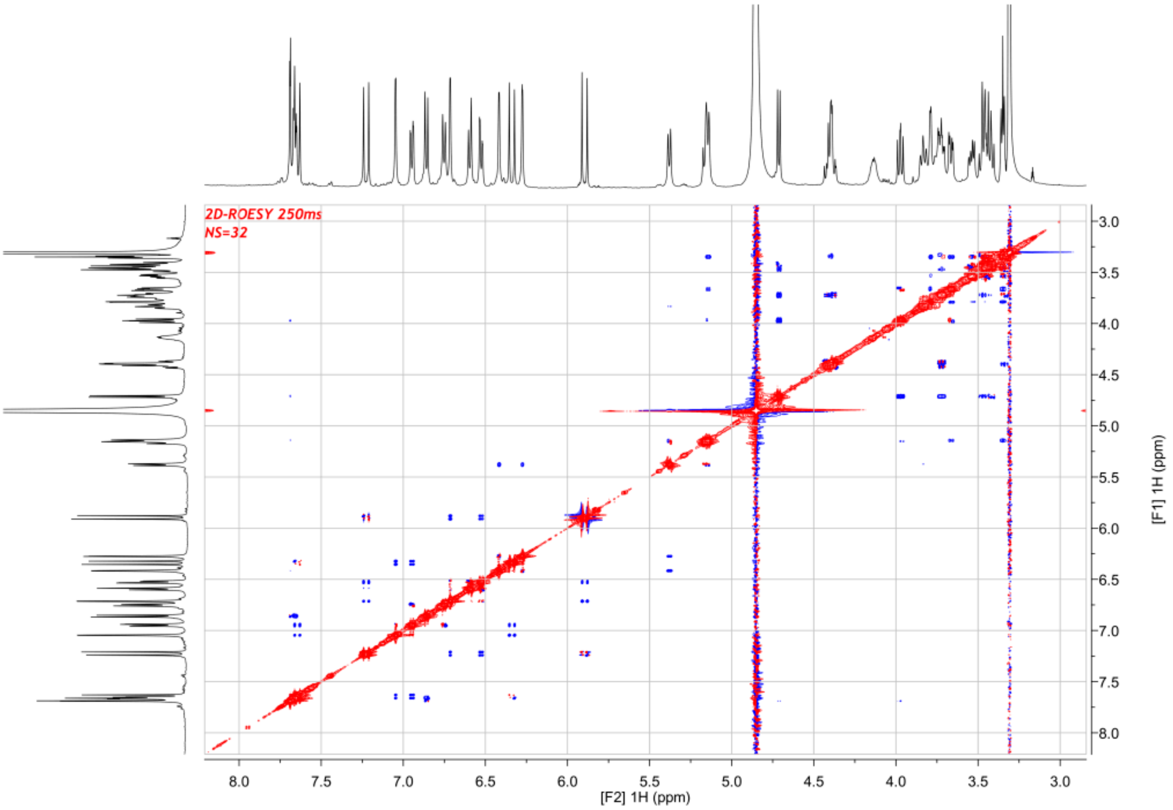

Figure S126. 2D *g*-HSQC-NMR spectrum of compound **12** (methanol-d_4, 500.18 MHz, 125.77 MHz).

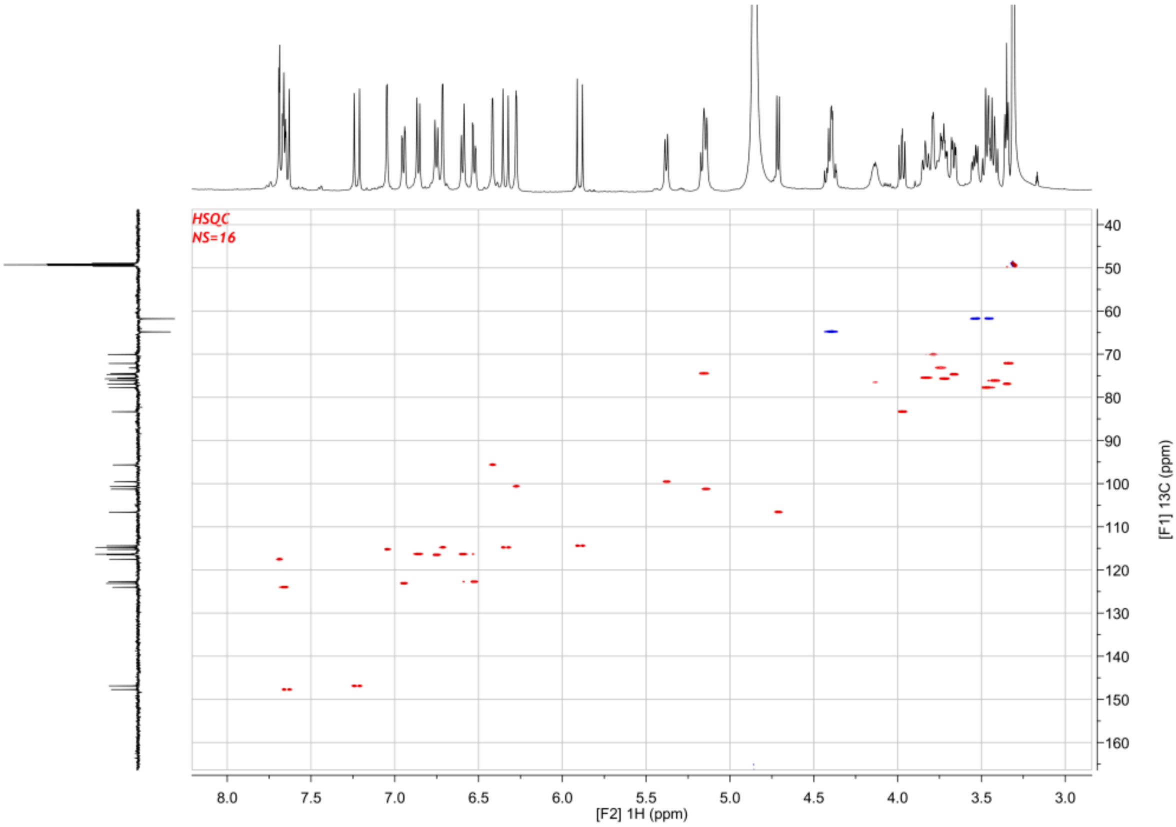

Figure S127. 2D *g*-HSQC-TOCSY NMR spectrum of compound **12** (methanol-*d*4, 500.18 MHz, 125.77 MHz).

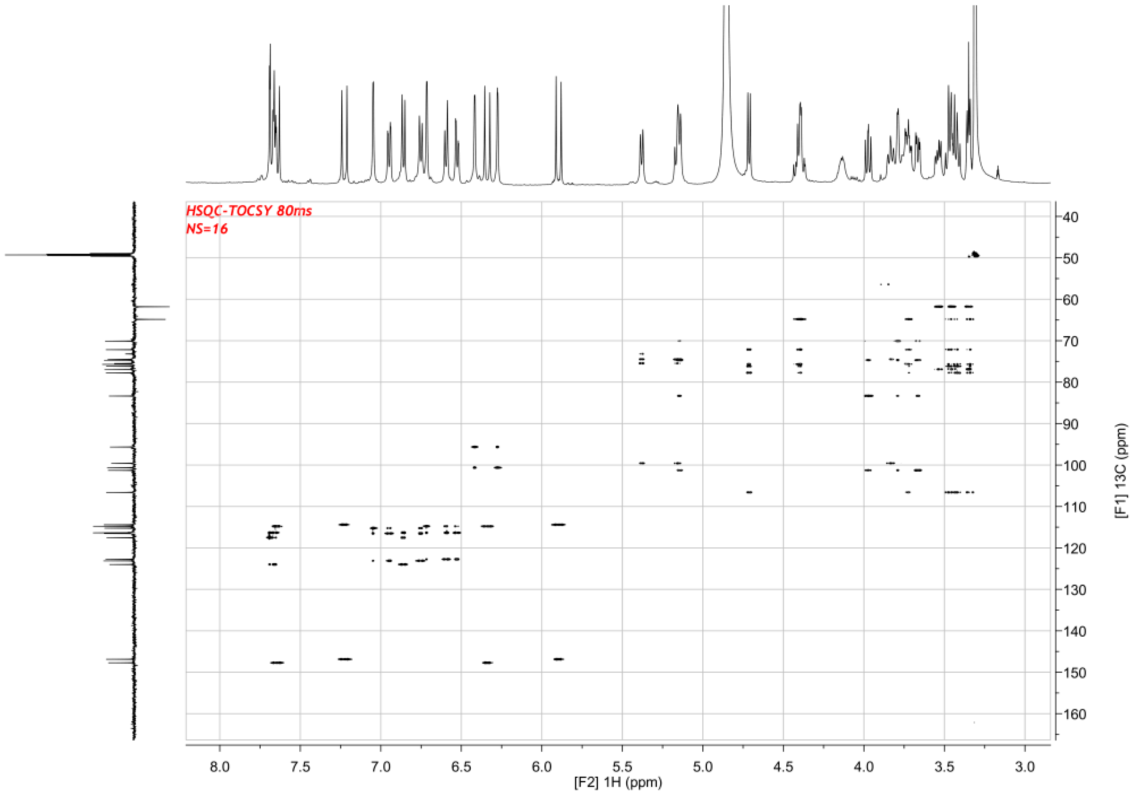

Figure S128. 2D *g*-HMBC-NMR spectrum of compound **12** (methanol-*d*4, 500.18 MHz, 125.77 MHz).

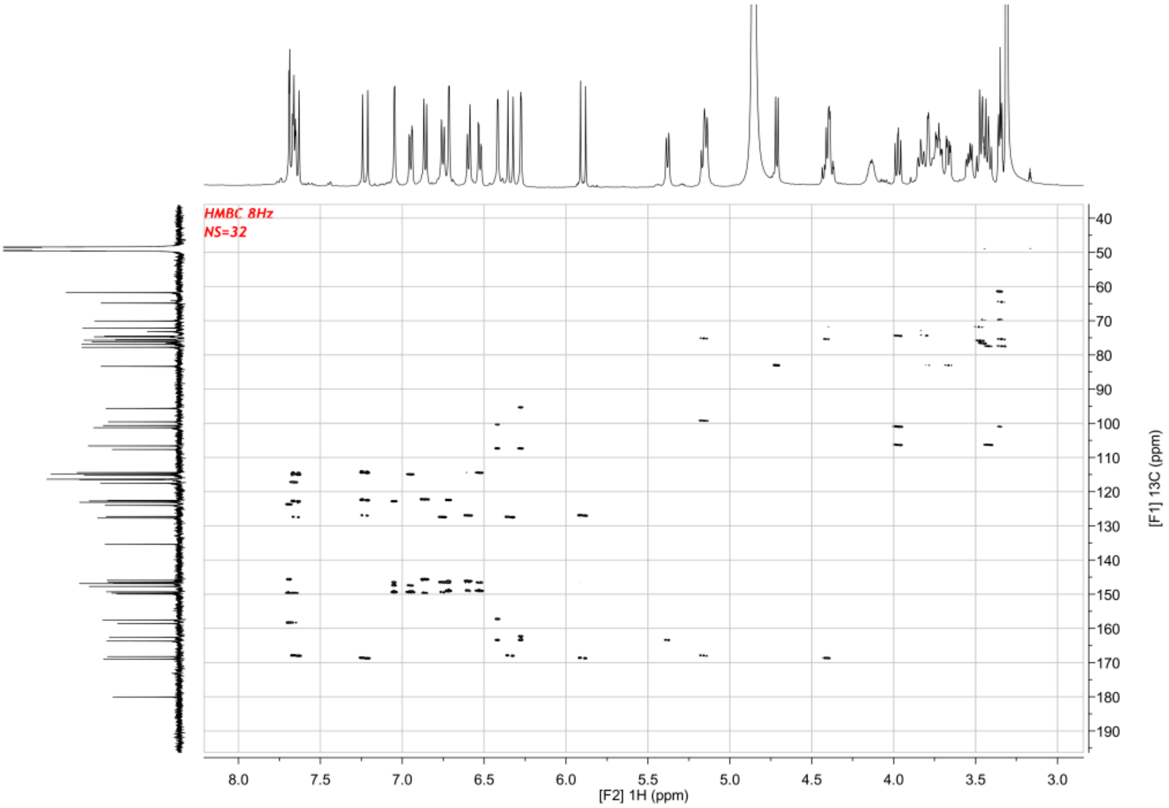

Figure S129. 1D TOCSY and 1D ROESY NMR subspectra of H-1$_{(7\text{-}O\text{-}\beta\text{-GlcA})}$ in compound **12** (methanol-d_4, 500.18 MHz).

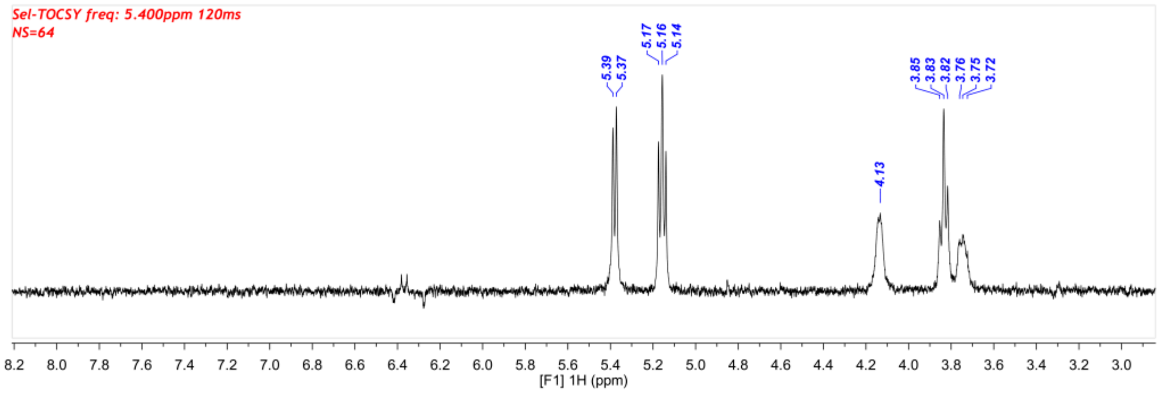

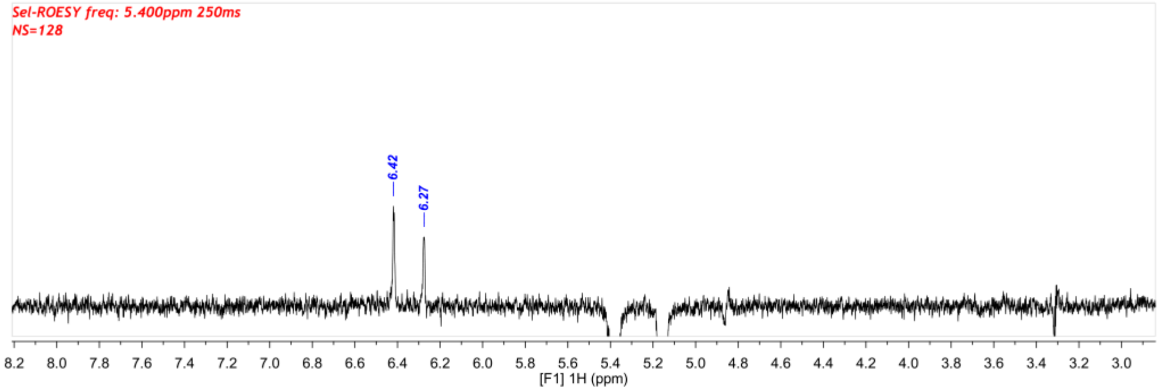

Figure S130. 1D TOCSY NMR subspectrum of H-2$_{(3\text{-}O\text{-}\beta\text{-Gal})}$ in compound **12** (methanol-d_4, 500.18 MHz).

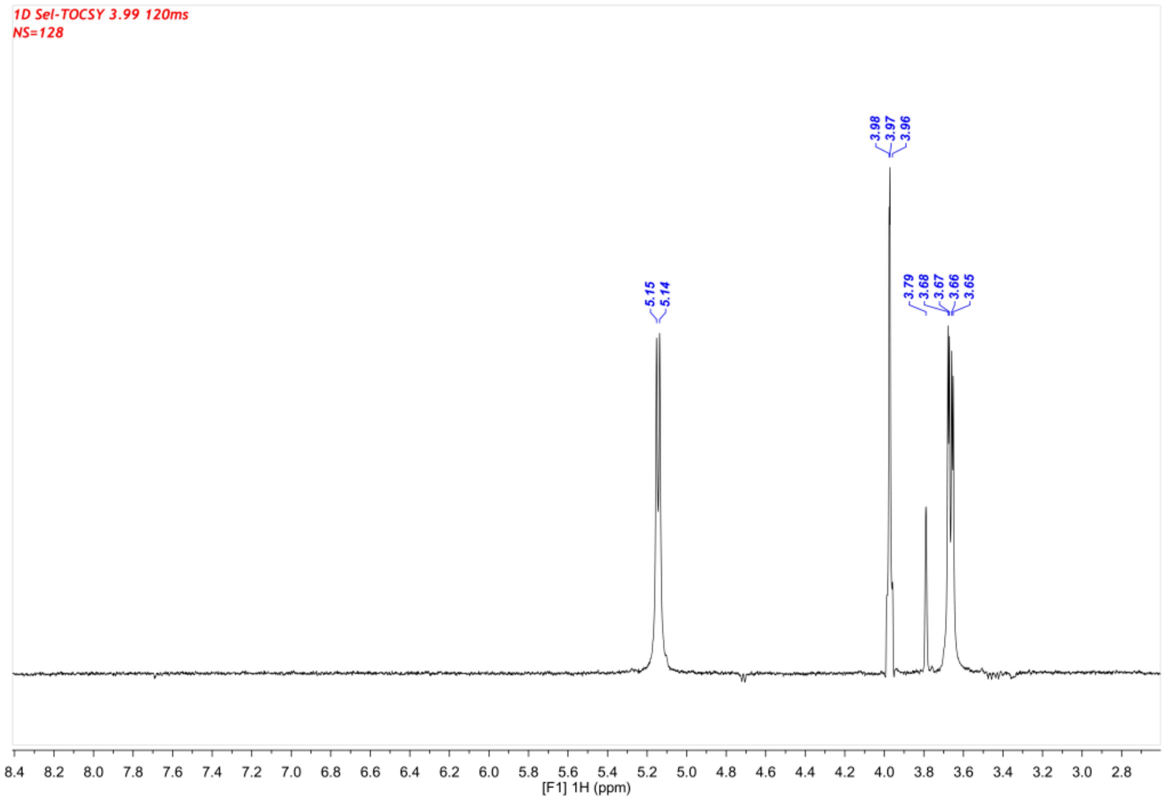

Figure S131. 1D TOCSY and 1D ROESY NMR subspectra of H-1$_{(2^{Gal}\text{-}O\text{-}\beta\text{-}Glc)}$ in compound **12** (methanol-*d*4, 500.18 MHz).

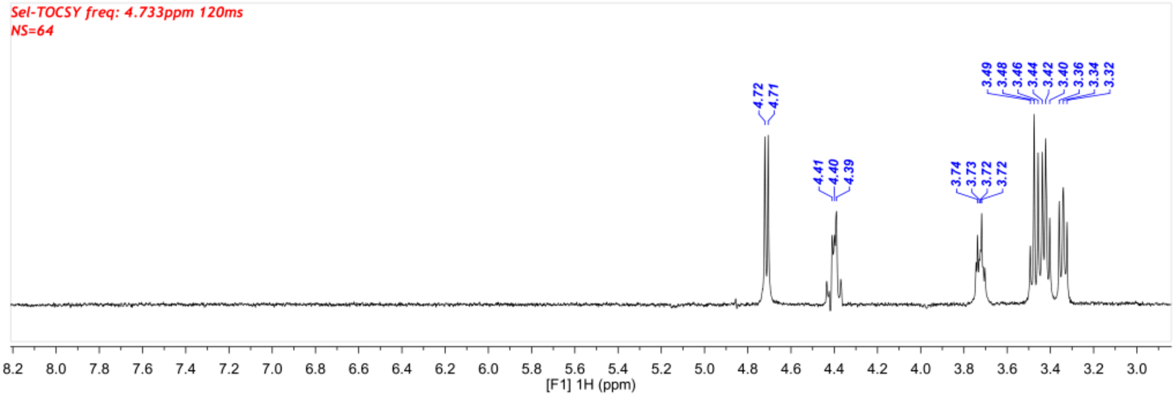

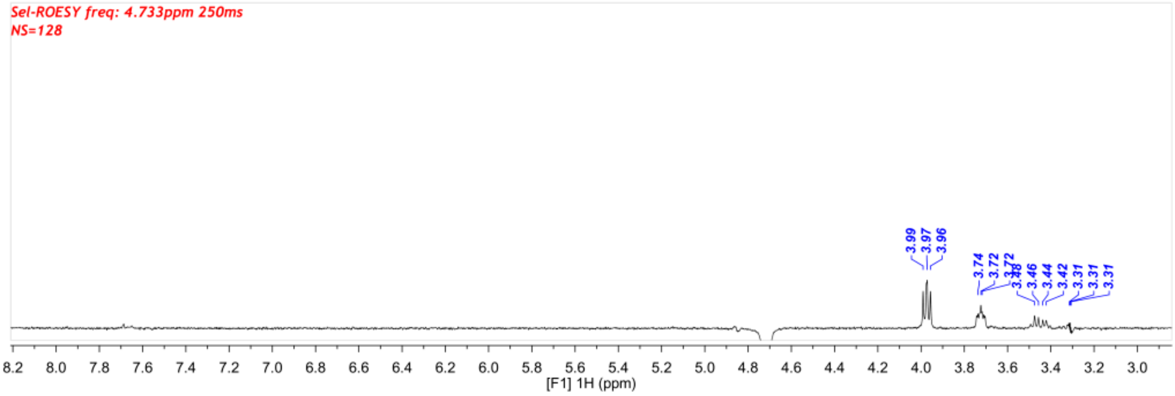

Compound 13

Figure S132. 1D ^{1}H-NMR spectrum of compound **13** (methanol-*d*4, 500.18 MHz).

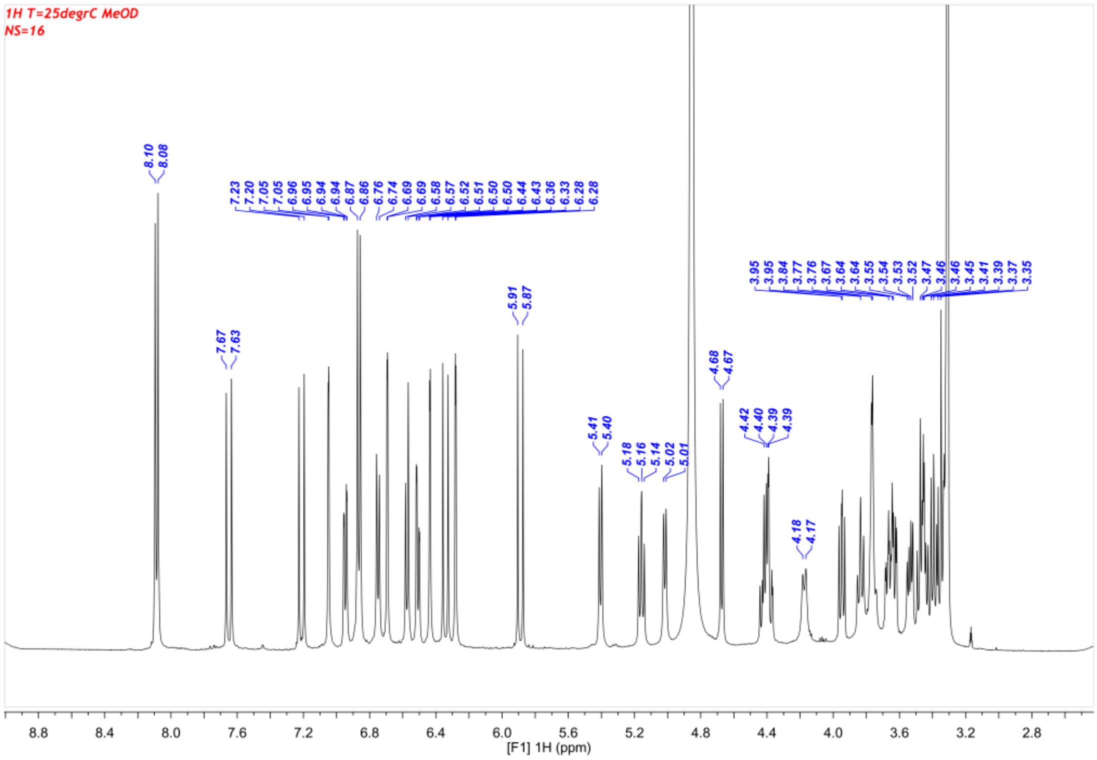

Figure S133. 1D ^{13}C-NMR spectra (1H decoupled and DEPT-135) of compound **13** (methanol-*d*4, 125.77 MHz).

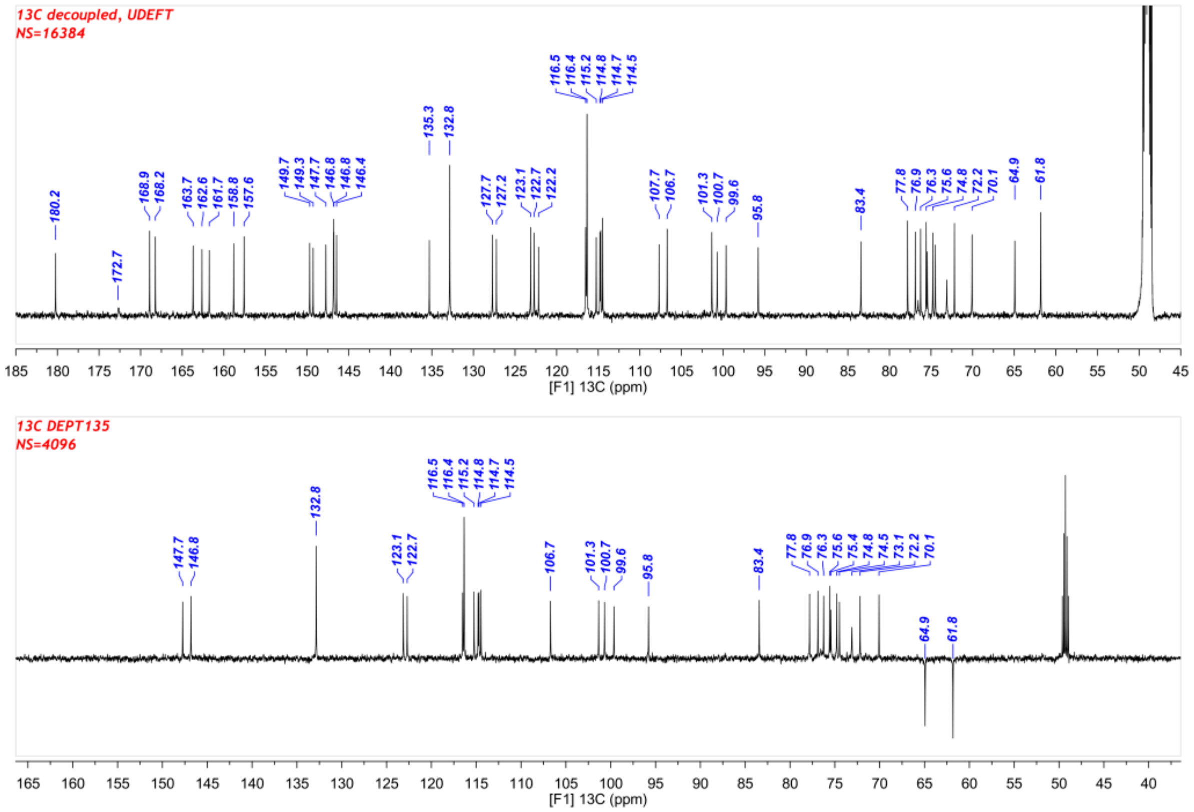

Figure S134. 2D *g*-COSY NMR spectrum of compound **13** (methanol-*d*4, 500.18 MHz).

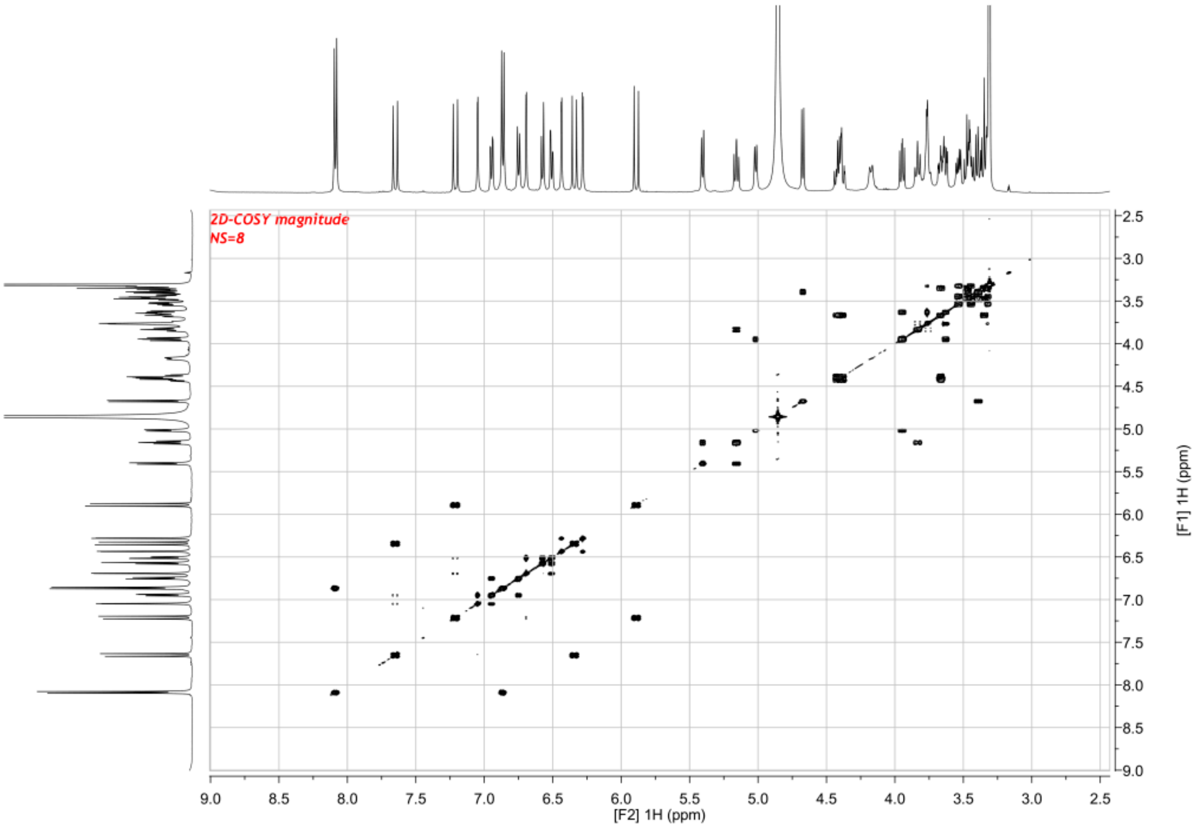

Figure S135. 2D TOCSY NMR spectrum of compound **13** (methanol-d_4, 500.18 MHz).

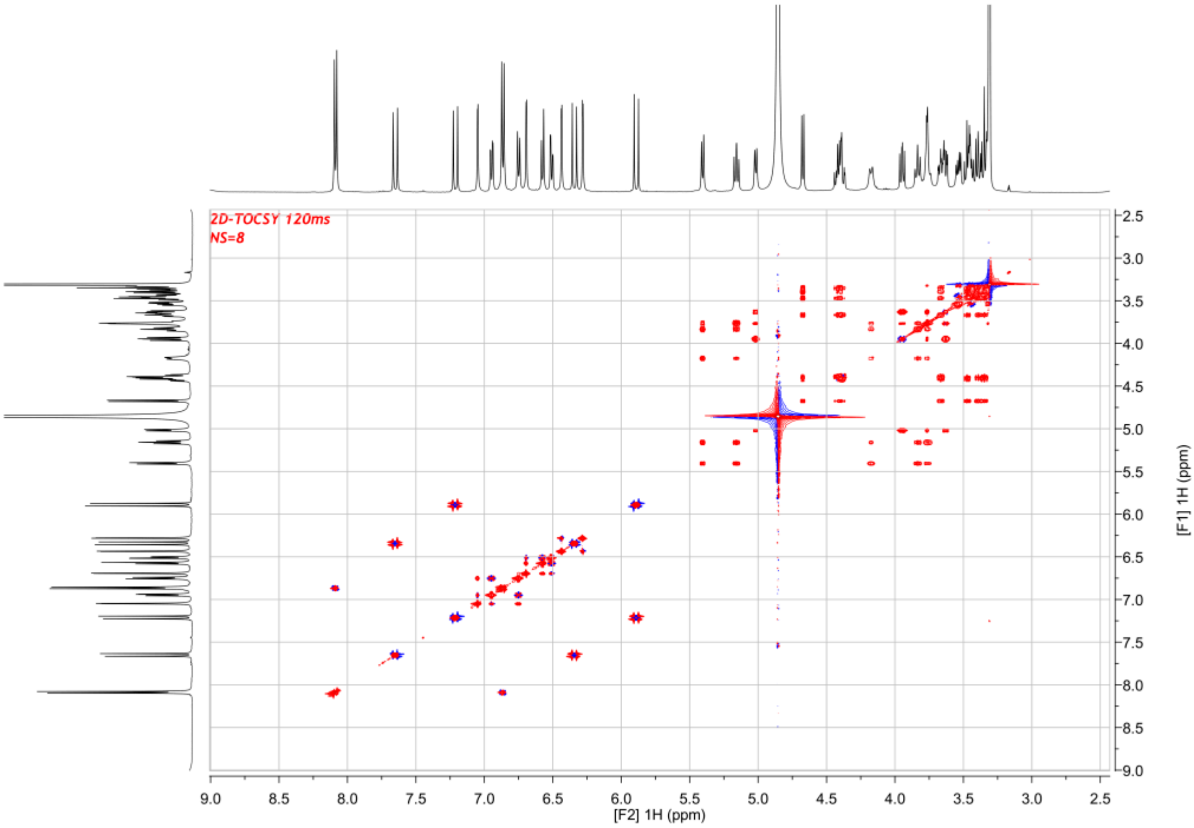

Figure S136. 2D ROESY NMR spectrum of compound **13** (methanol-d_4, 500.18 MHz).

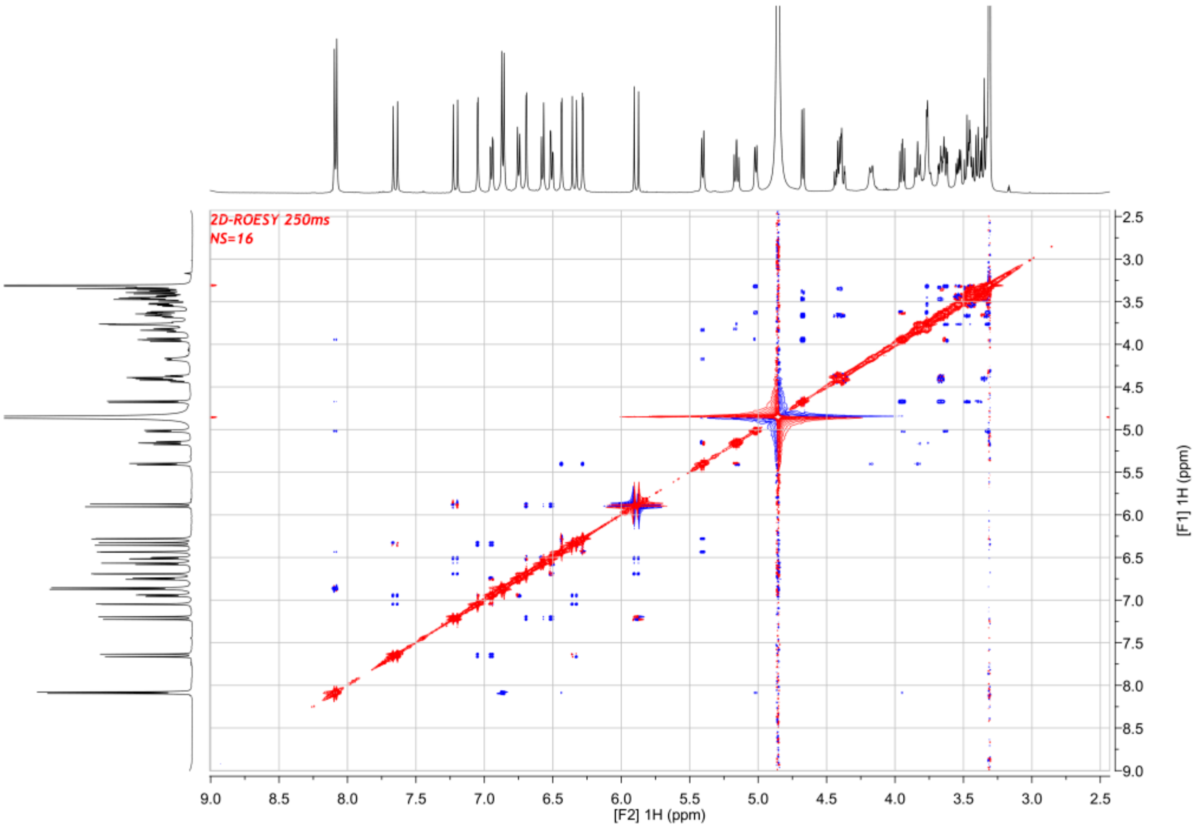

Figure S137. 2D *g*-HSQC-NMR spectrum of compound **13** (methanol-d_4, 500.18 MHz, 125.77 MHz).

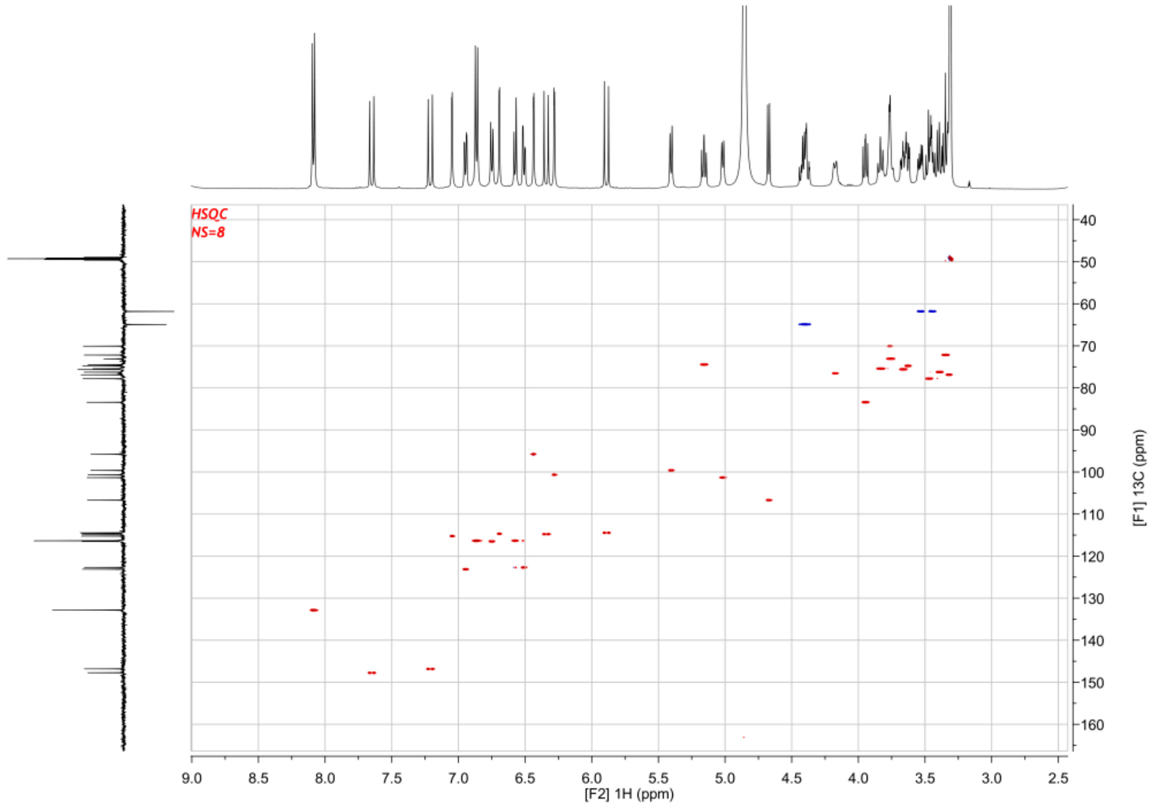

Figure S138. 2D *g*-HSQC-TOCSY NMR spectrum of compound **13** (methanol-d_4, 500.18 MHz, 125.77 MHz).

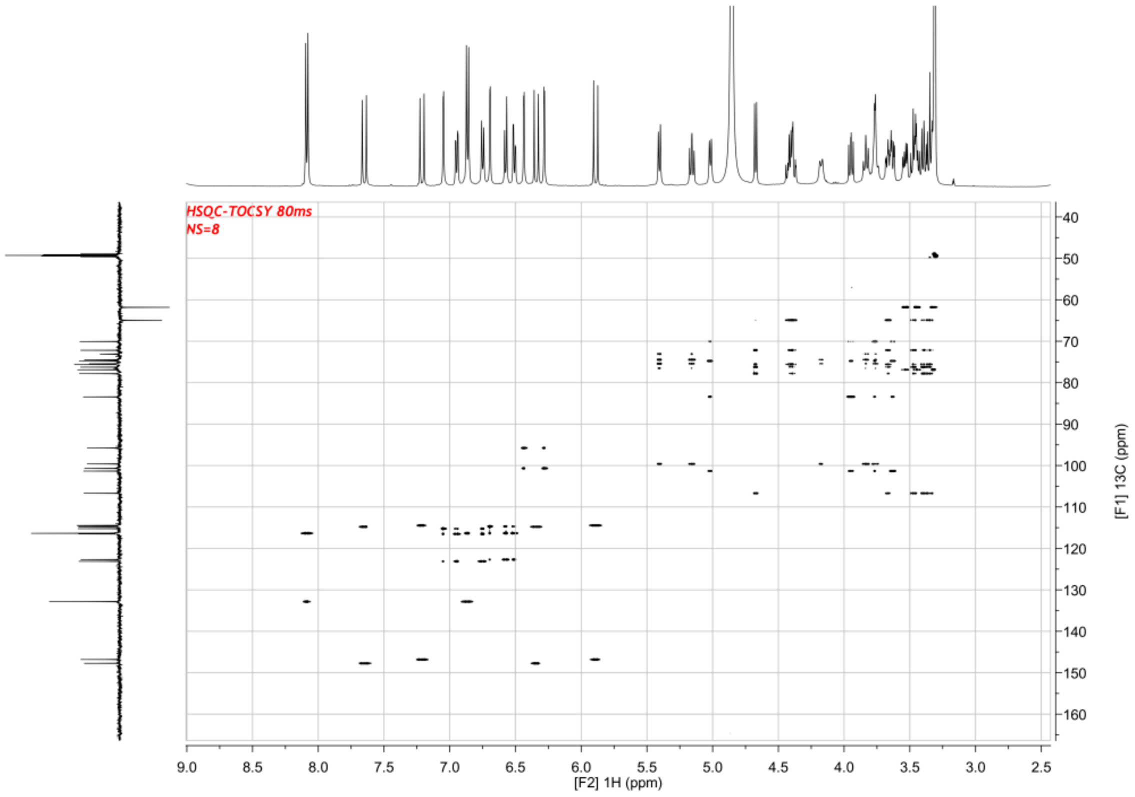

Figure S139. 2D *g*-HMBC-NMR spectrum of compound **13** (methanol-d_4, 500.18 MHz, 125.77 MHz).

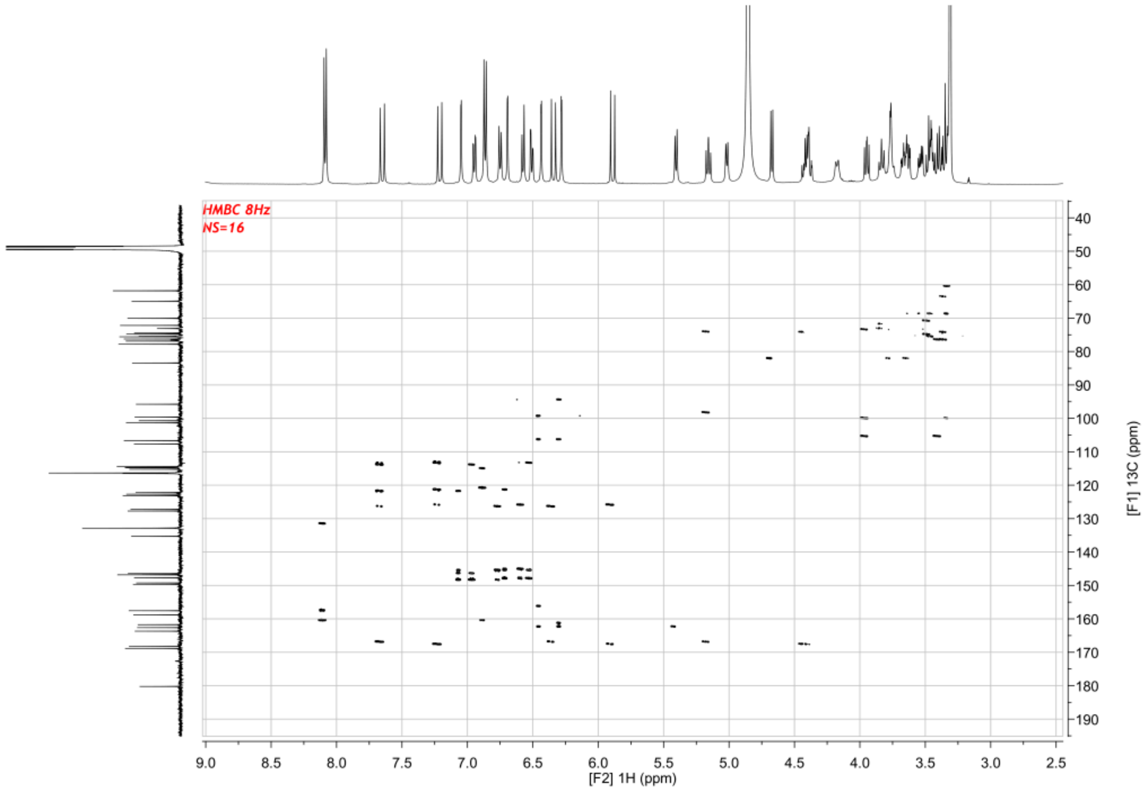

Figure S140. 1D TOCSY and 1D ROESY NMR subspectra of H-1$_{(7\text{-}O\text{-}\beta\text{-GlcA})}$ in compound **13** (methanol-d_4, 500.18 MHz).

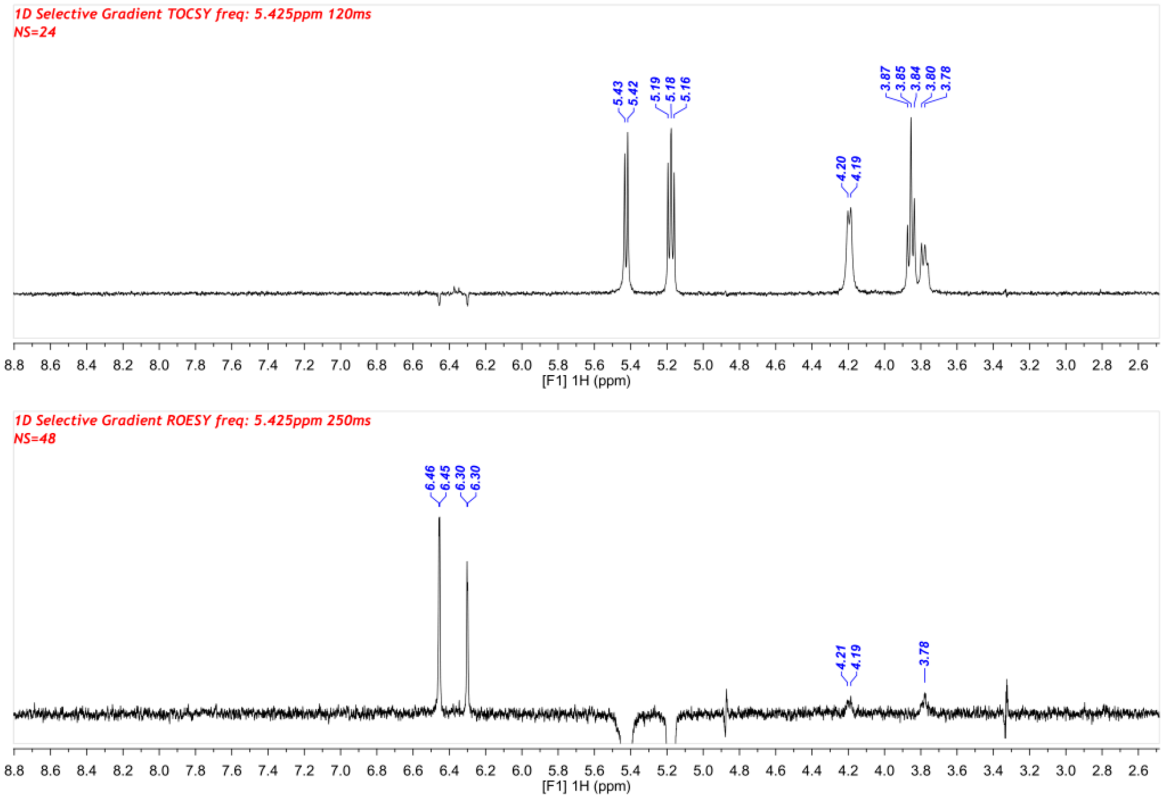

Figure S141. 1D TOCSY and 1D ROESY NMR subspectra of H-1(3-*O*-β-Gal) in compound **13** (methanol-d_4, 500.18 MHz).

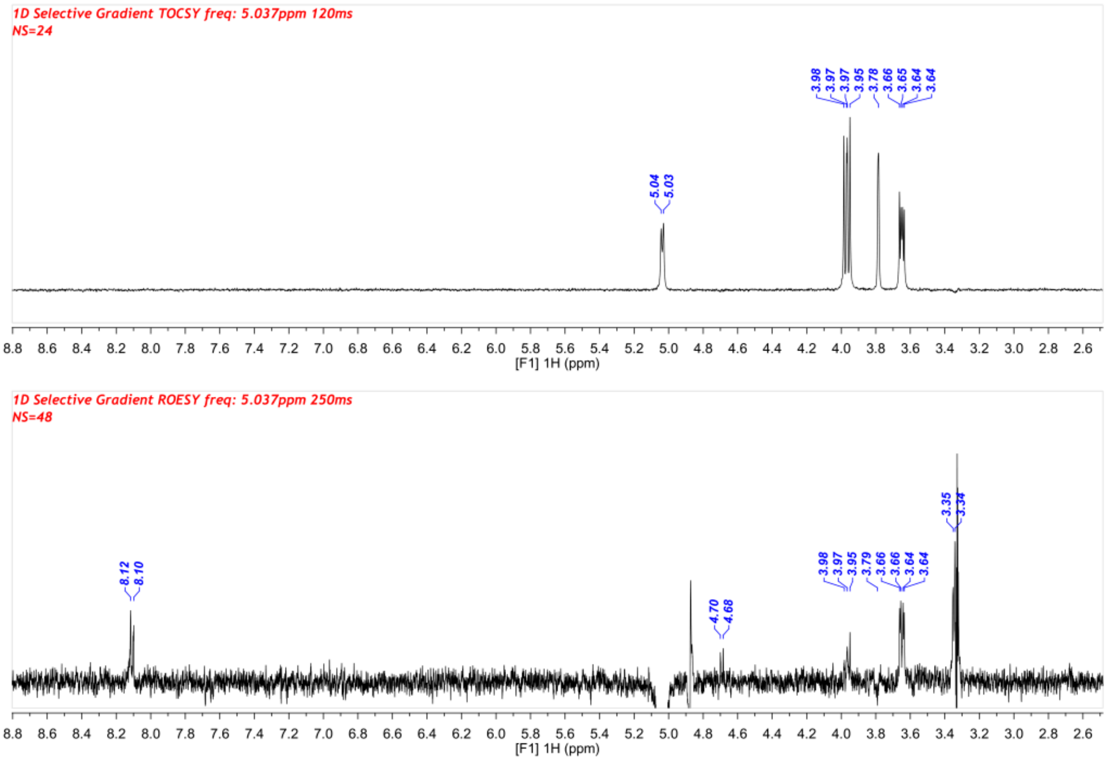

Figure S142. 1D TOCSY and 1D ROESY NMR subspectra of H-1(2^Gal-*O*-β-Glc) in compound **13** (methanol-d_4, 500.18 MHz).

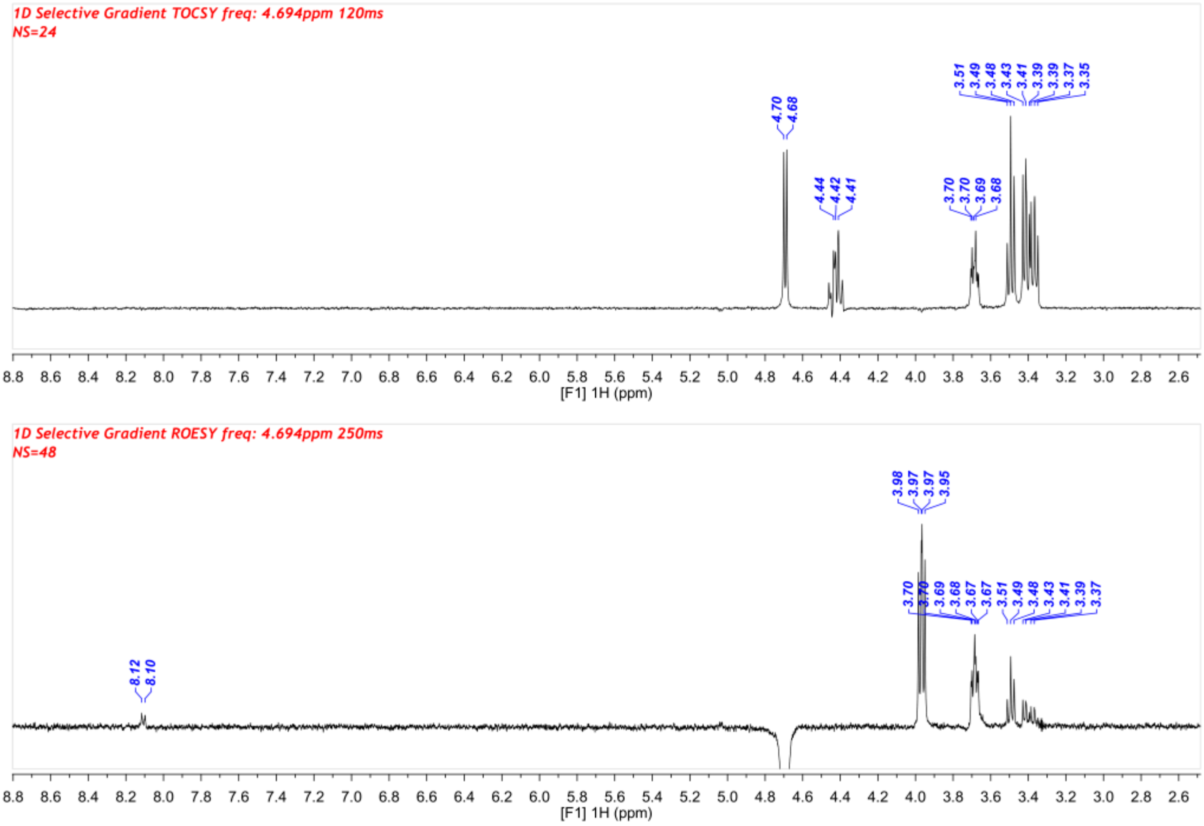

Compound 14

Figure S143. 1D ^{1}H-NMR spectrum of compound **14** (methanol-d_4, 500.18 MHz).

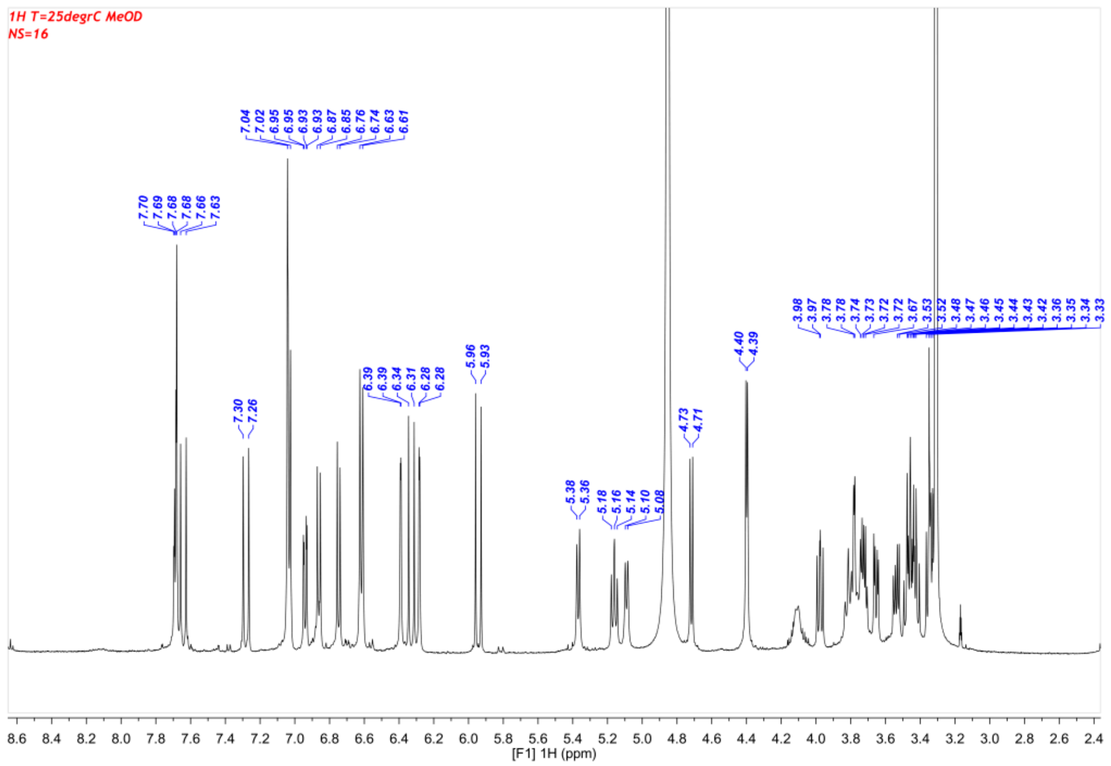

Figure S144. 1D ^{13}C-NMR spectra (1H decoupled and DEPT-135) of compound **14** (methanol-d_4, 125.77 MHz).

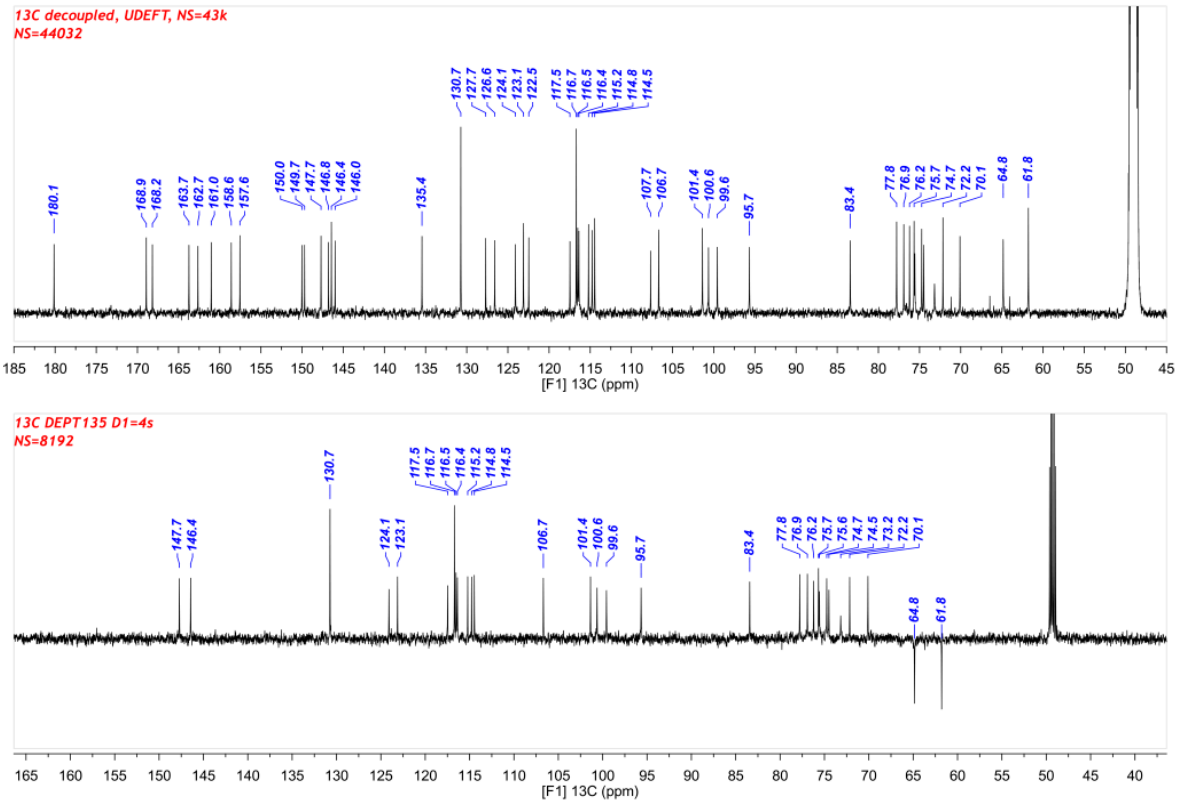

Figure S145. 2D *g*-COSY NMR spectrum of compound **14** (methanol-*d*4, 500.18 MHz).

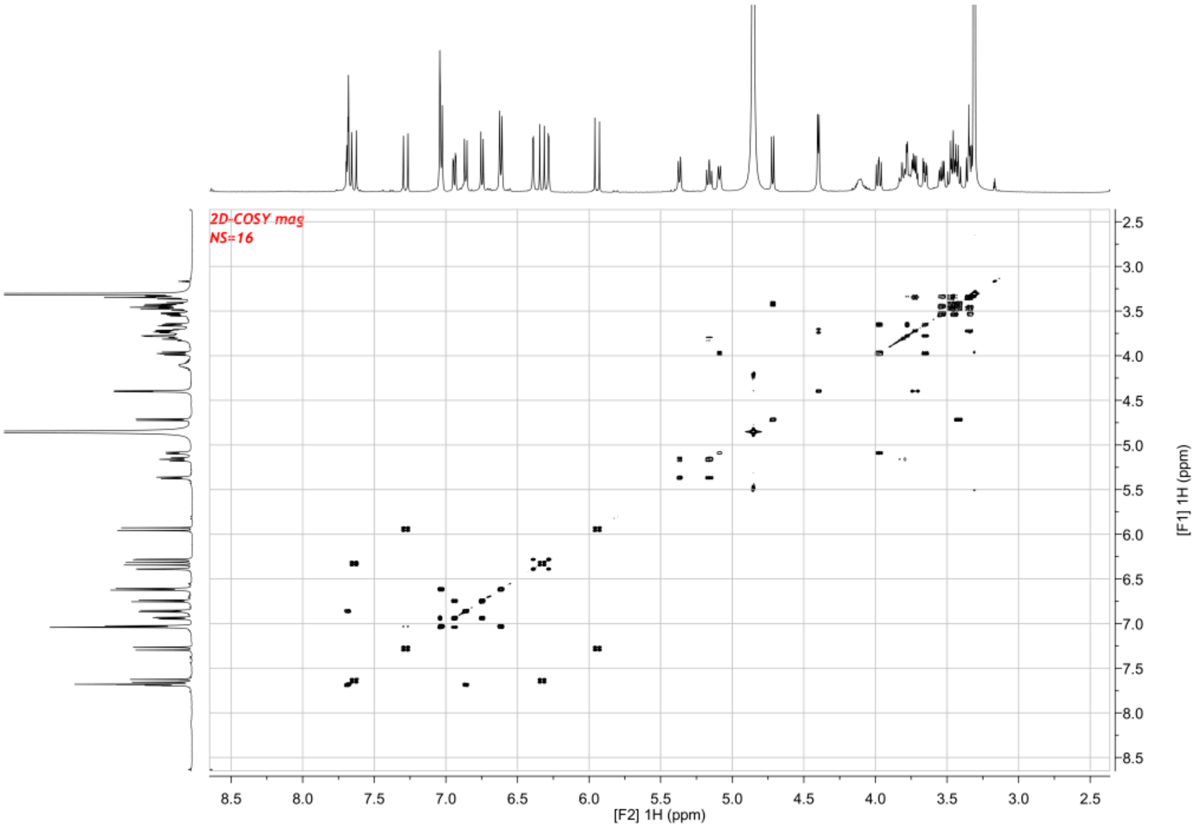

Figure S146. 2D TOCSY NMR spectrum of compound **14** (methanol-*d*4, 500.18 MHz).

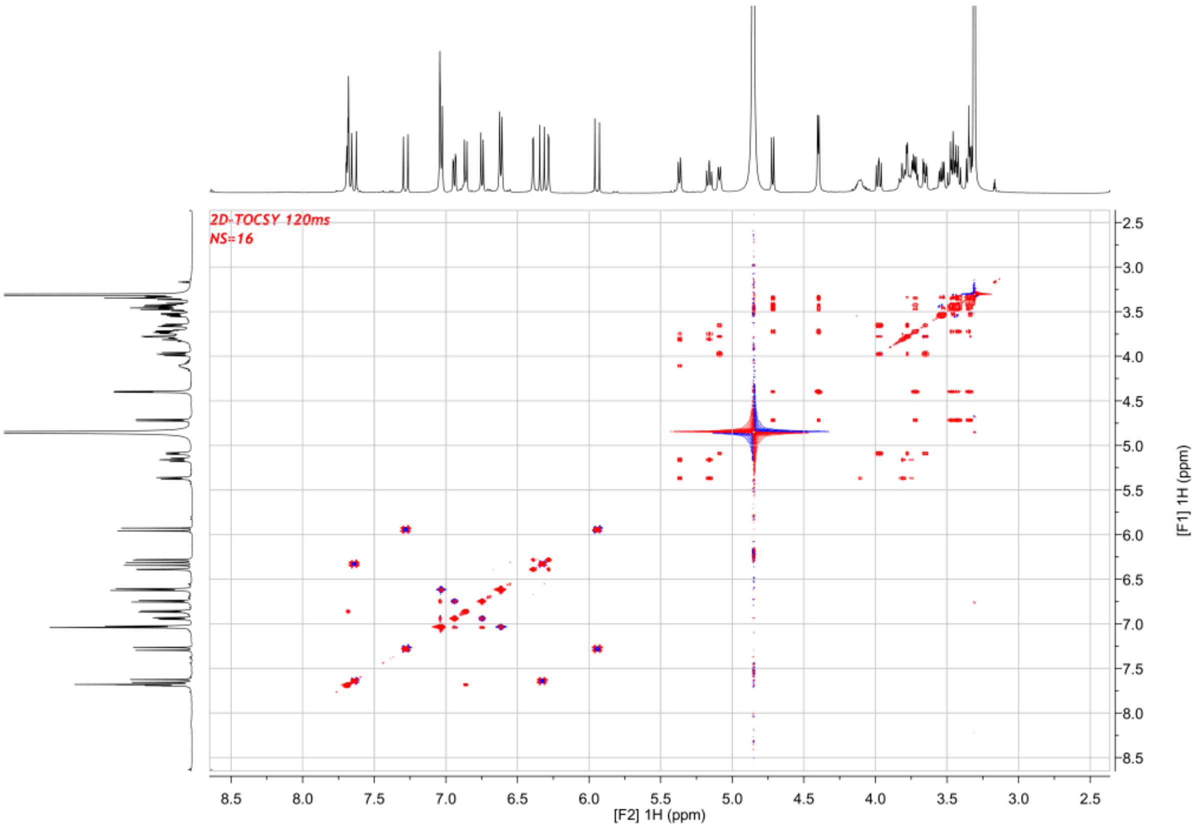

Figure S147. 2D ROESY NMR spectrum of compound **14** (methanol-d_4, 500.18 MHz).

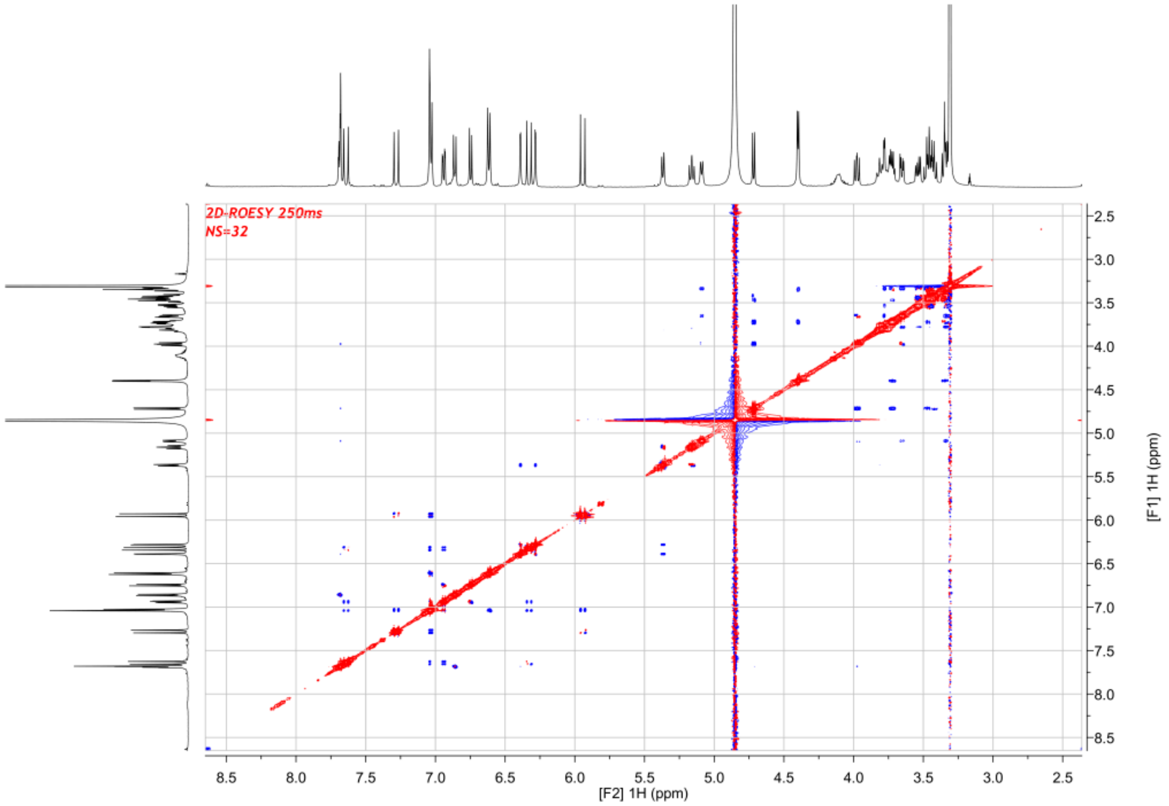

Figure S148. 2D *g*-HSQC-NMR spectrum of compound **14** (methanol-d_4, 500.18 MHz, 125.77 MHz).

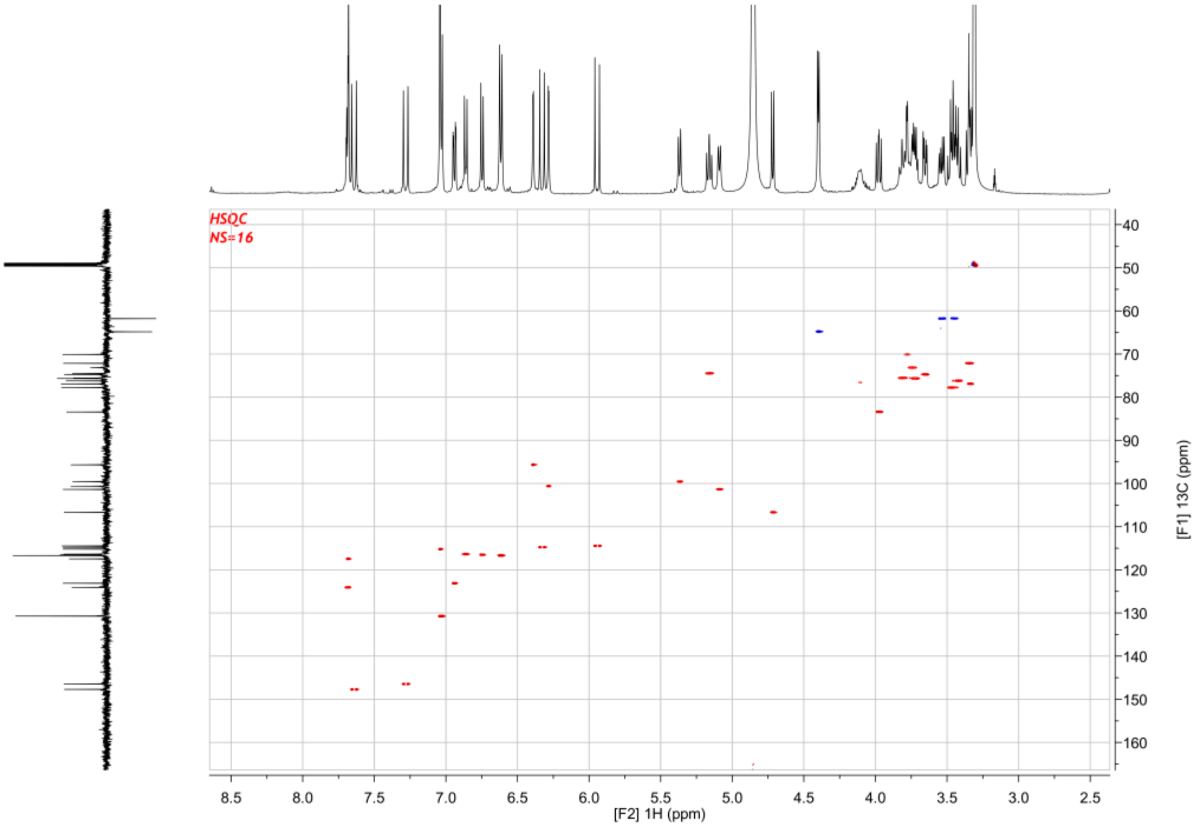

Figure S149. 2D *g*-HSQC-TOCSY NMR spectrum of compound **14** (methanol-d_4, 500.18 MHz, 125.77 MHz).

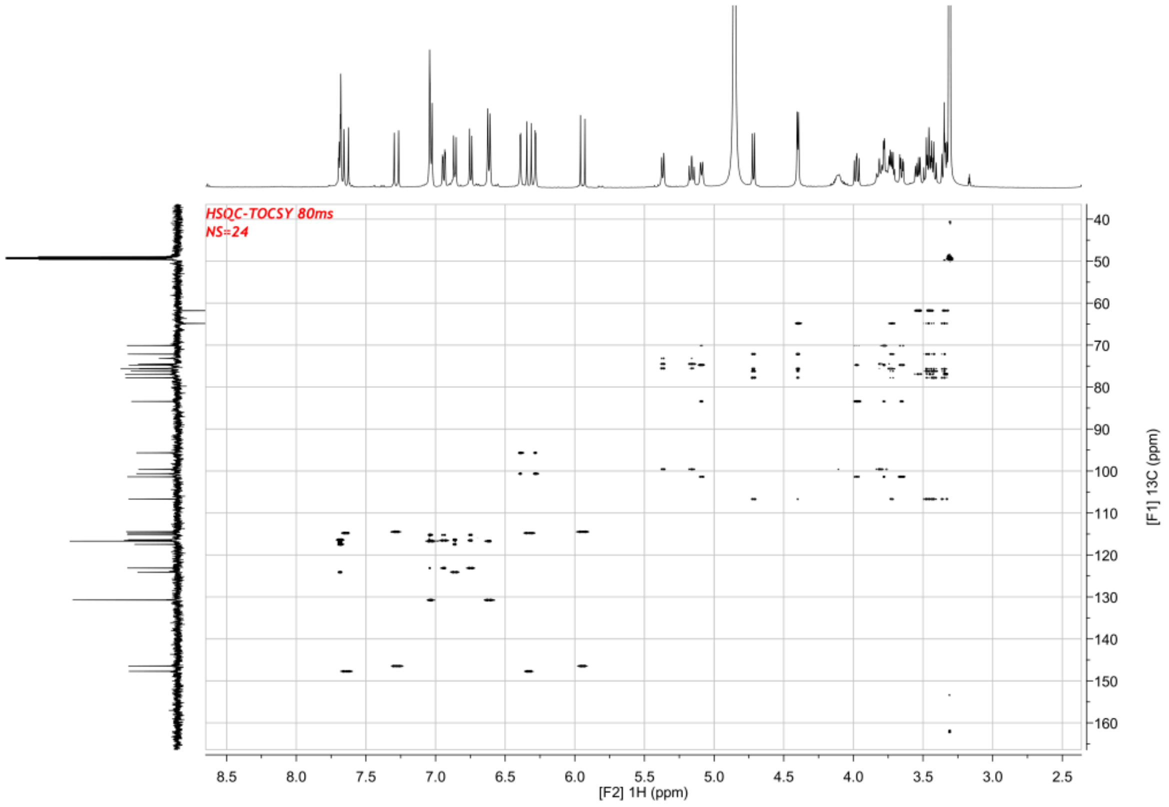

Figure S150. 2D *g*-HMBC-NMR spectrum of compound **14** (methanol-d_4, 500.18 MHz, 125.77 MHz).

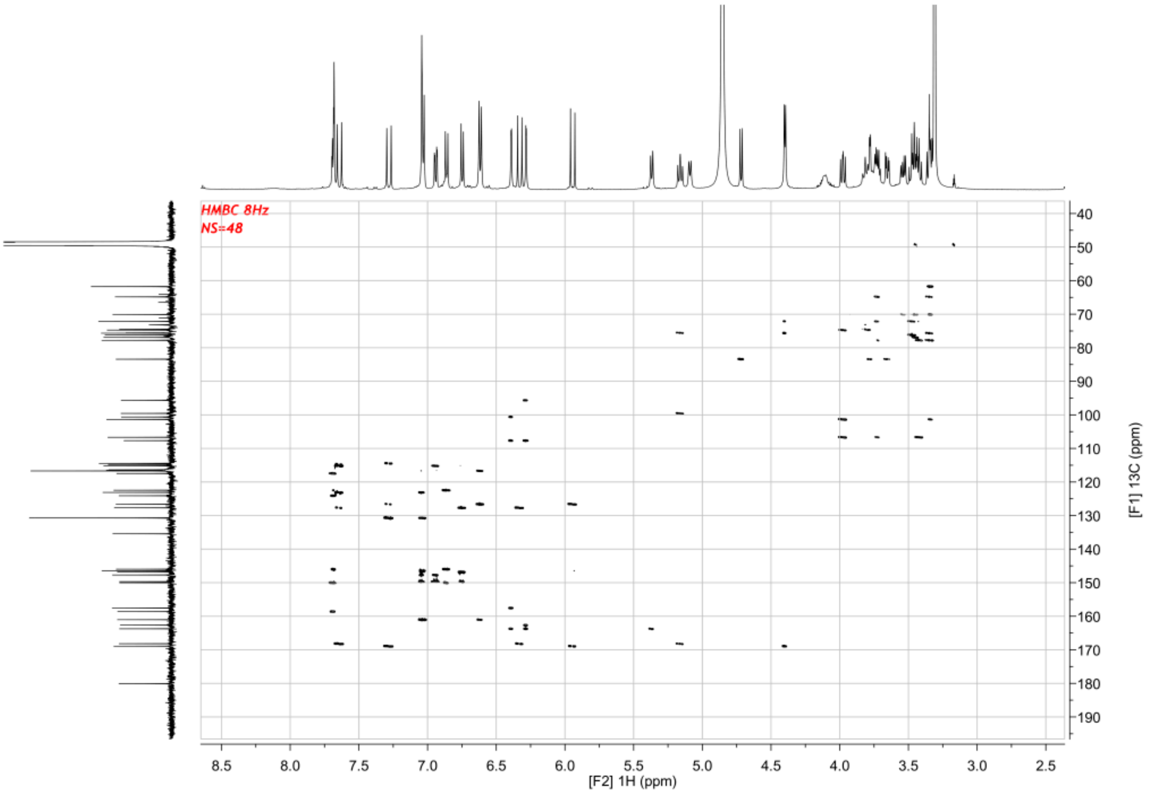

Figure S151. 1D TOCSY and 1D ROESY NMR subspectra of H-1(7-*O*-β-GlcA) in compound **14** (methanol-*d*4, 500.18 MHz).

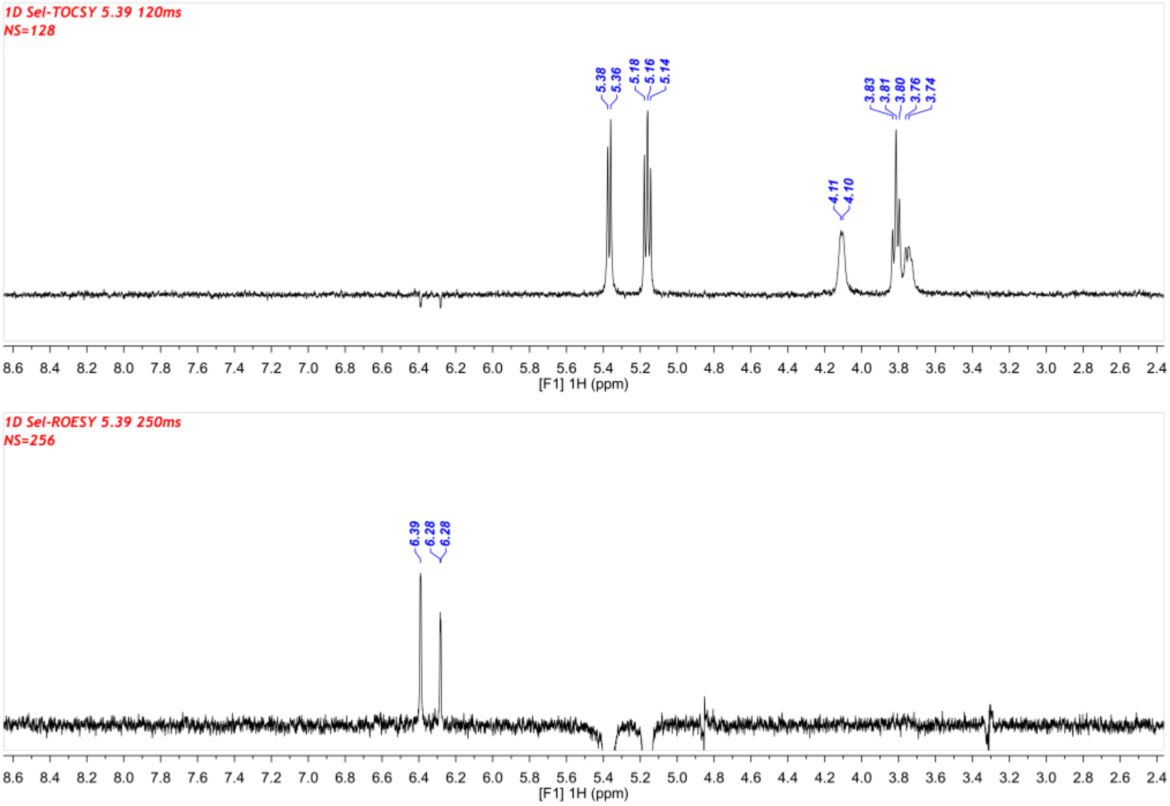

Figure S152. 1D TOCSY and 1D ROESY NMR subspectra of H-1(3-*O*-β-Gal) in compound **14** (methanol-*d*4, 500.18 MHz).

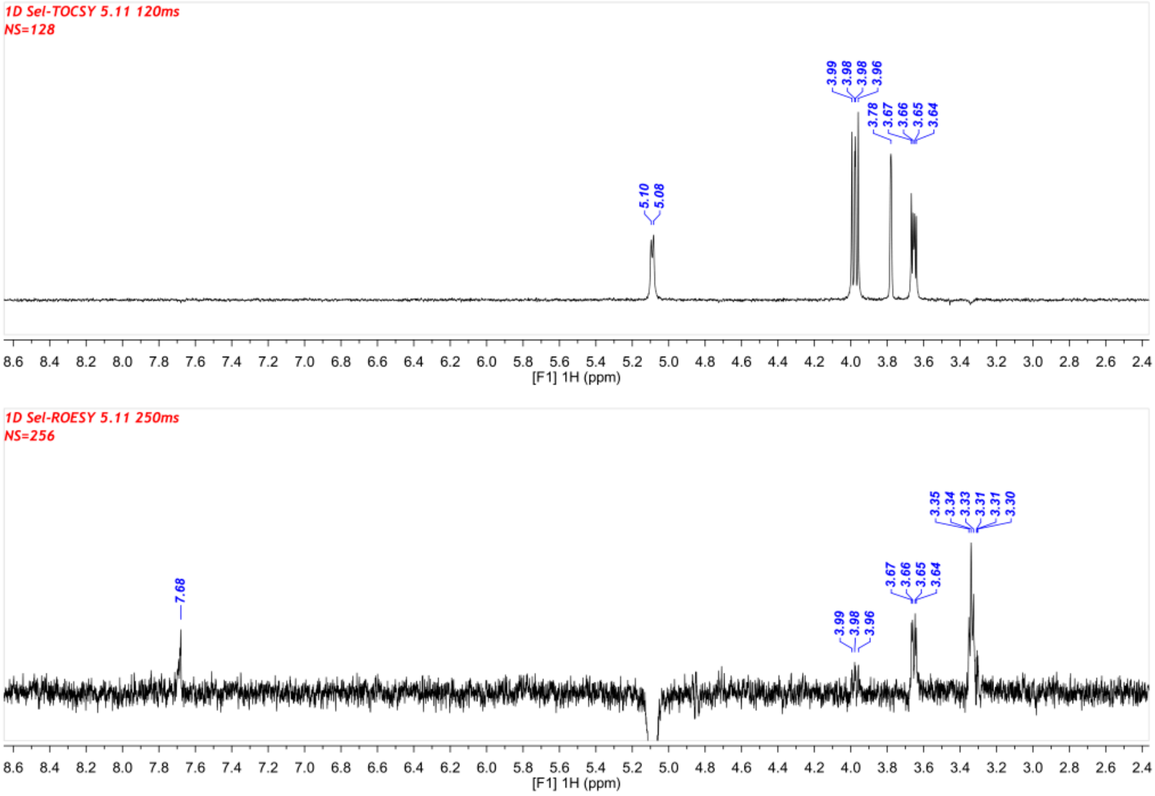

Figure S153. 1D TOCSY and 1D ROESY NMR subspectra of H-1$_{(2^{Gal}\text{-}O\text{-}\beta\text{-Glc})}$ in compound **14** (methanol-d_4, 500.18 MHz).

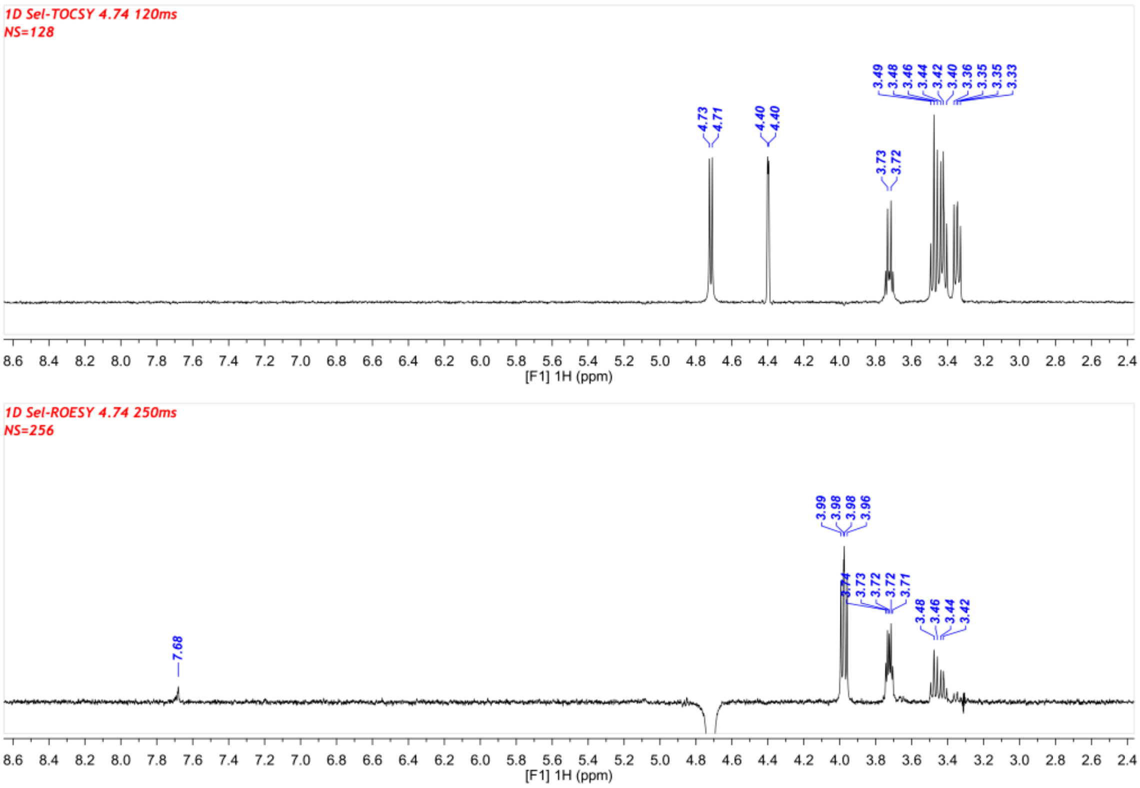

Compound 15

Figure S154. 1D ^{1}H-NMR spectrum of compound **15** (methanol-d_4, 500.18 MHz).

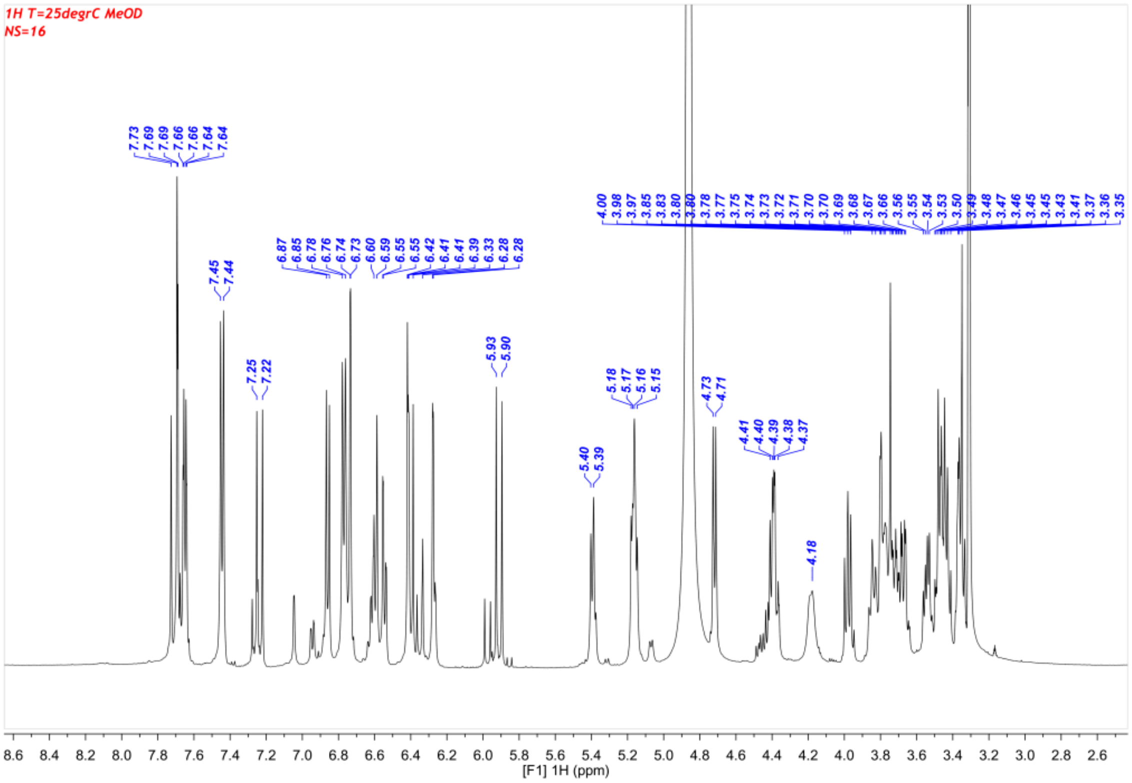

Figure S155. 1D ^{13}C-NMR spectra (1H decoupled and DEPT-135) of compound **15** (methanol-d_4, 125.77 MHz).

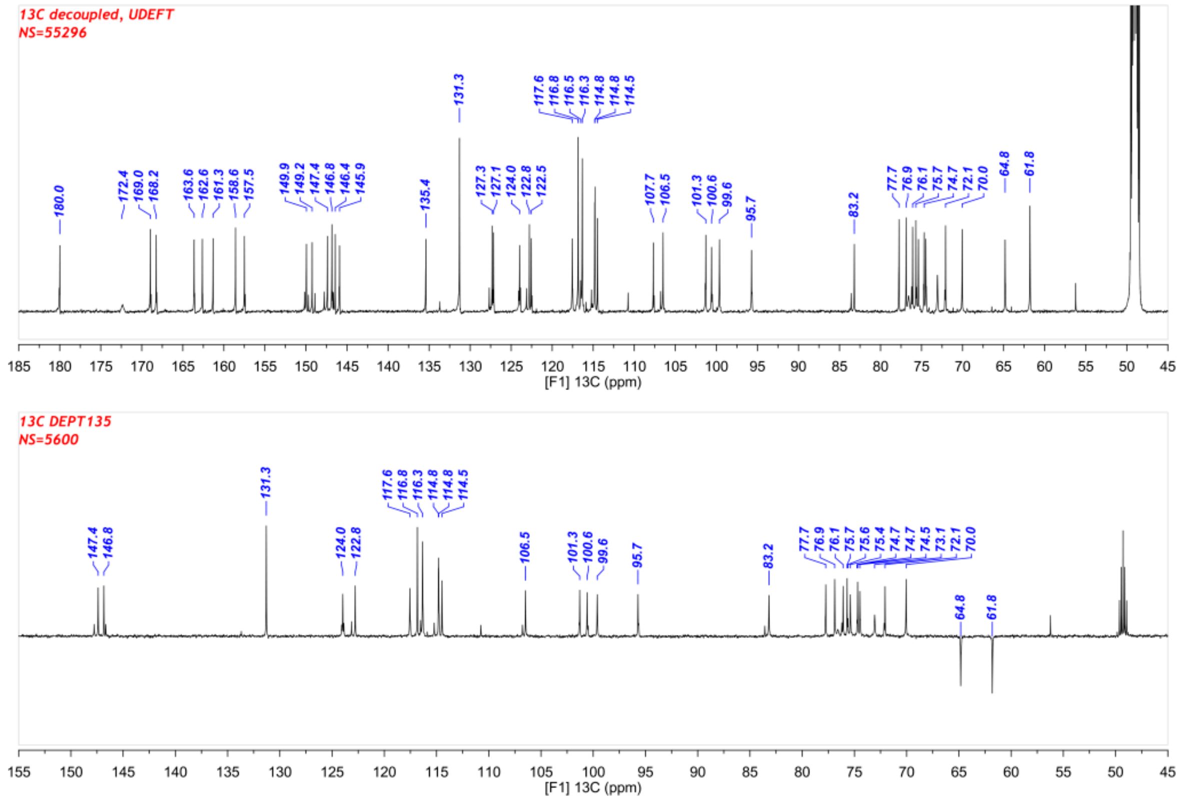

Figure S156. 2D *g*-COSY NMR spectrum of compound **15** (methanol-d_4, 500.18 MHz).

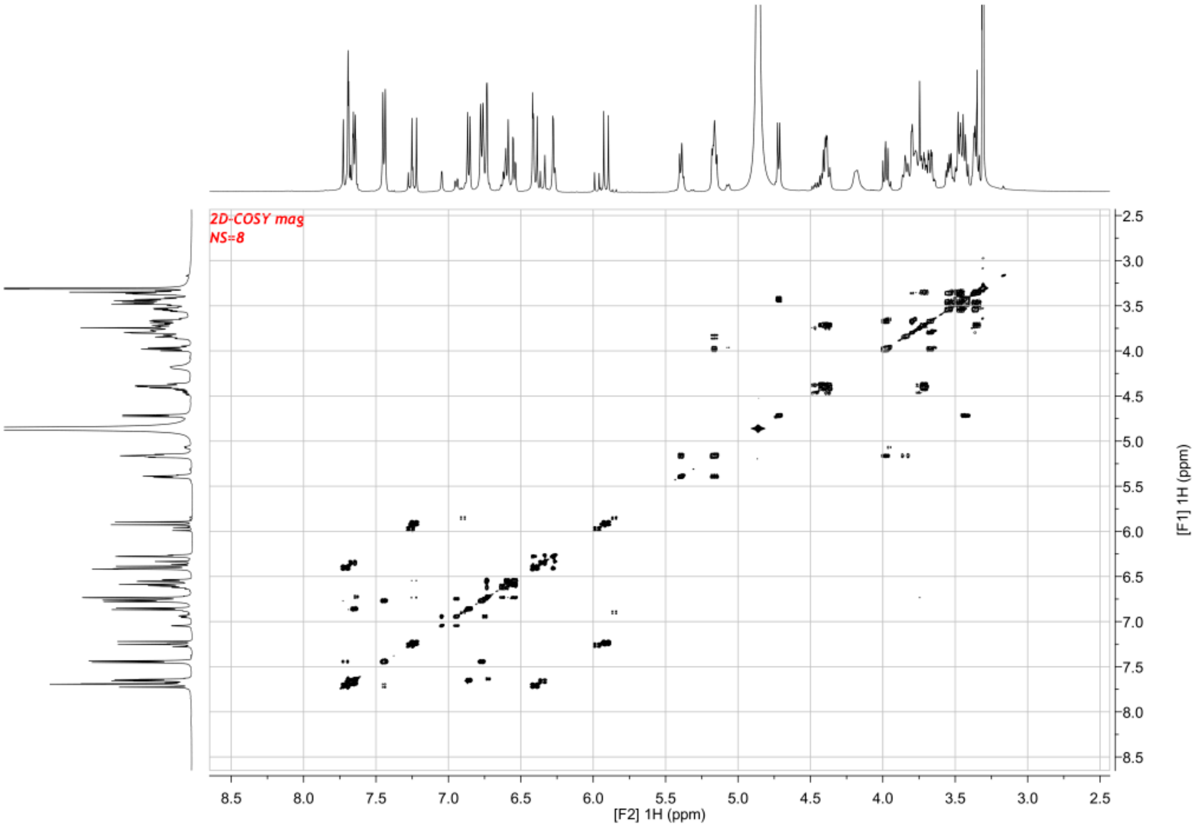

Figure S157. 2D TOCSY NMR spectrum of compound **15** (methanol-*d*4, 500.18 MHz).

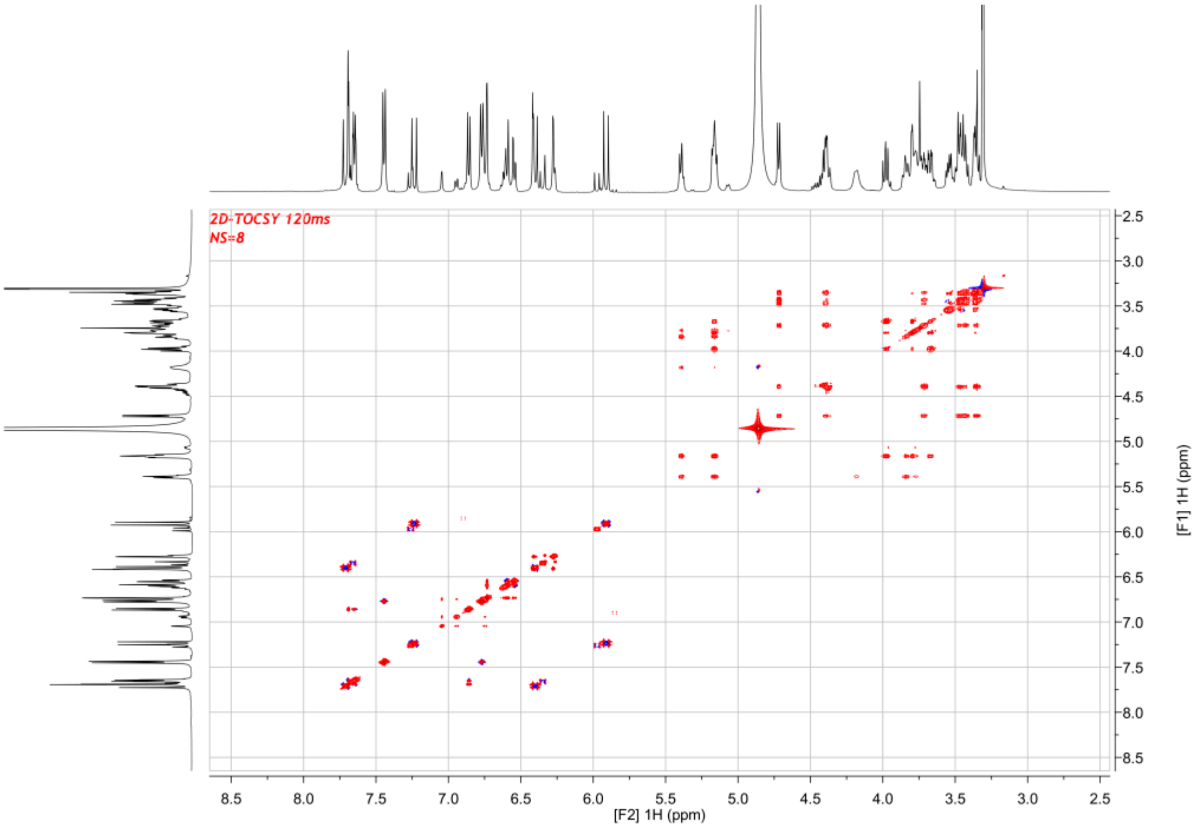

Figure S158. 2D ROESY NMR spectrum of compound **15** (methanol-*d*4, 500.18 MHz).

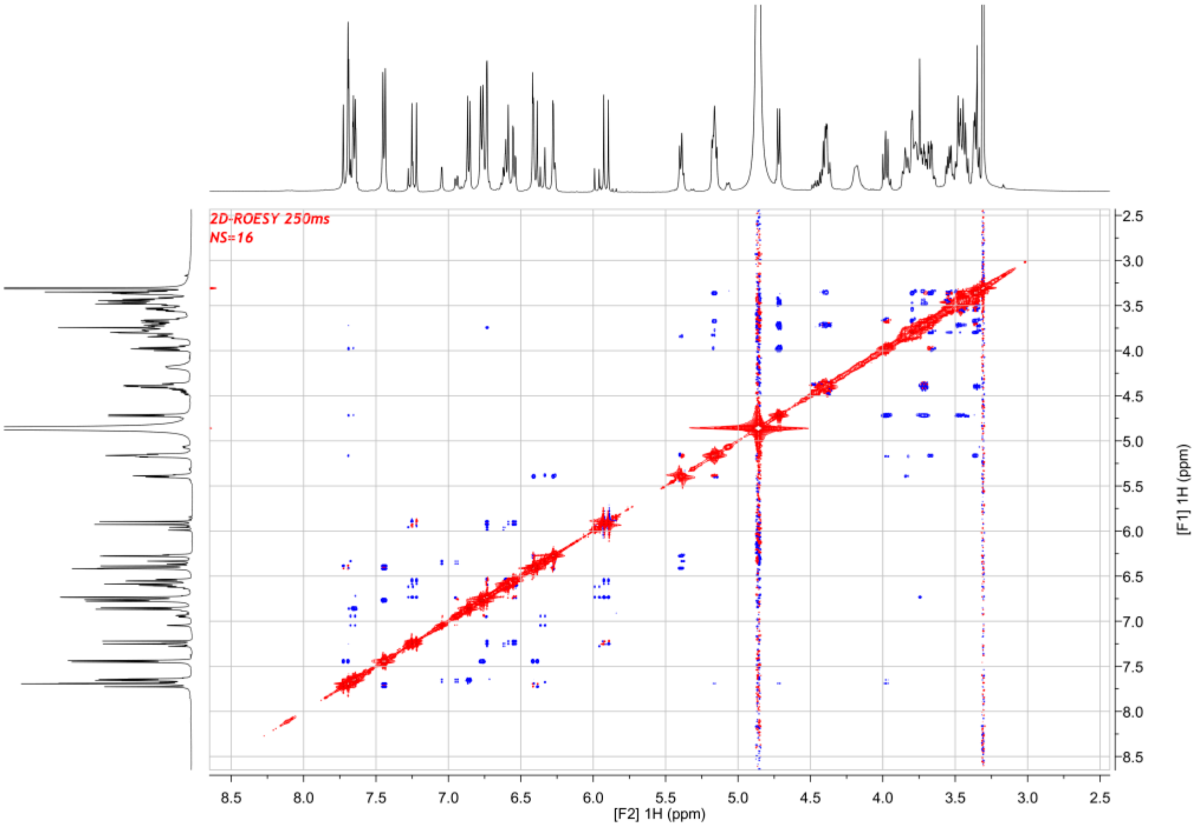

Figure S159. 2D *g*-HSQC-NMR spectrum of compound **15** (methanol-*d*4, 500.18 MHz, 125.77 MHz).

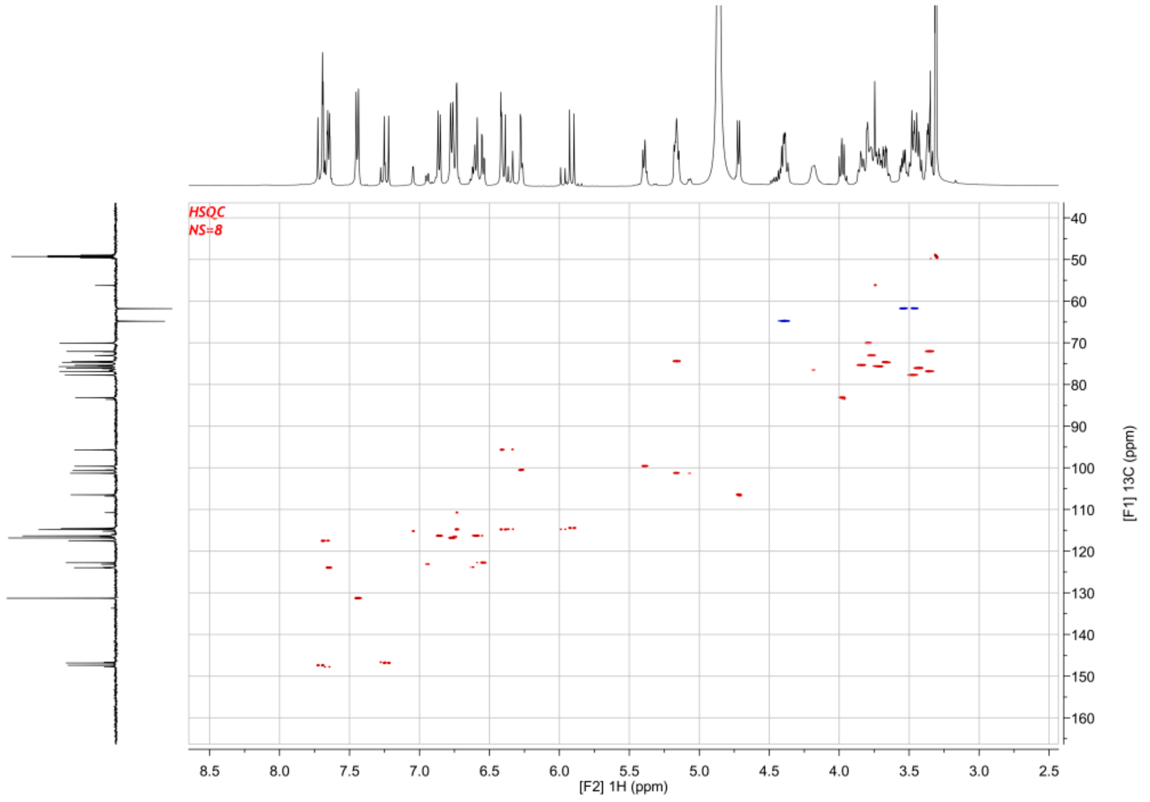

Figure S160. 2D *g*-HSQC-TOCSY NMR spectrum of compound **15** (methanol-*d*4, 500.18 MHz, 125.77 MHz).

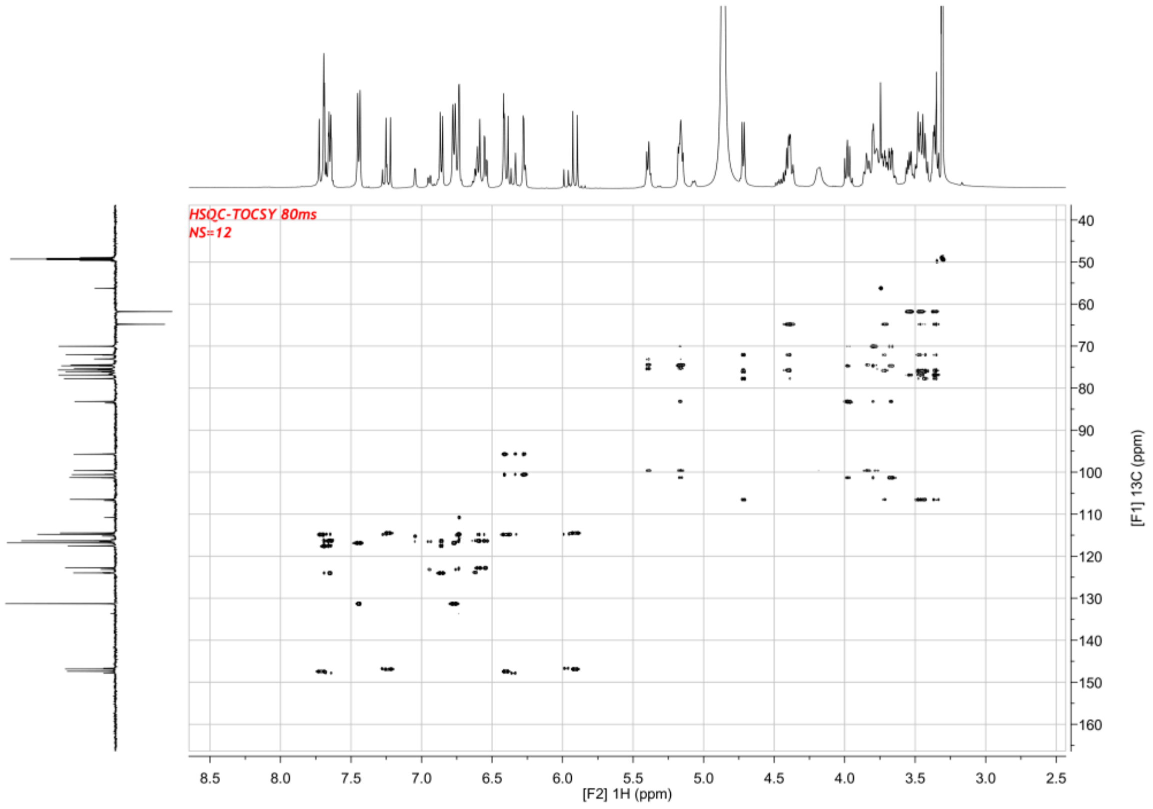

Figure S161. 2D *g*-HMBC-NMR spectrum of compound **15** (methanol-d_4, 500.18 MHz, 125.77 MHz).

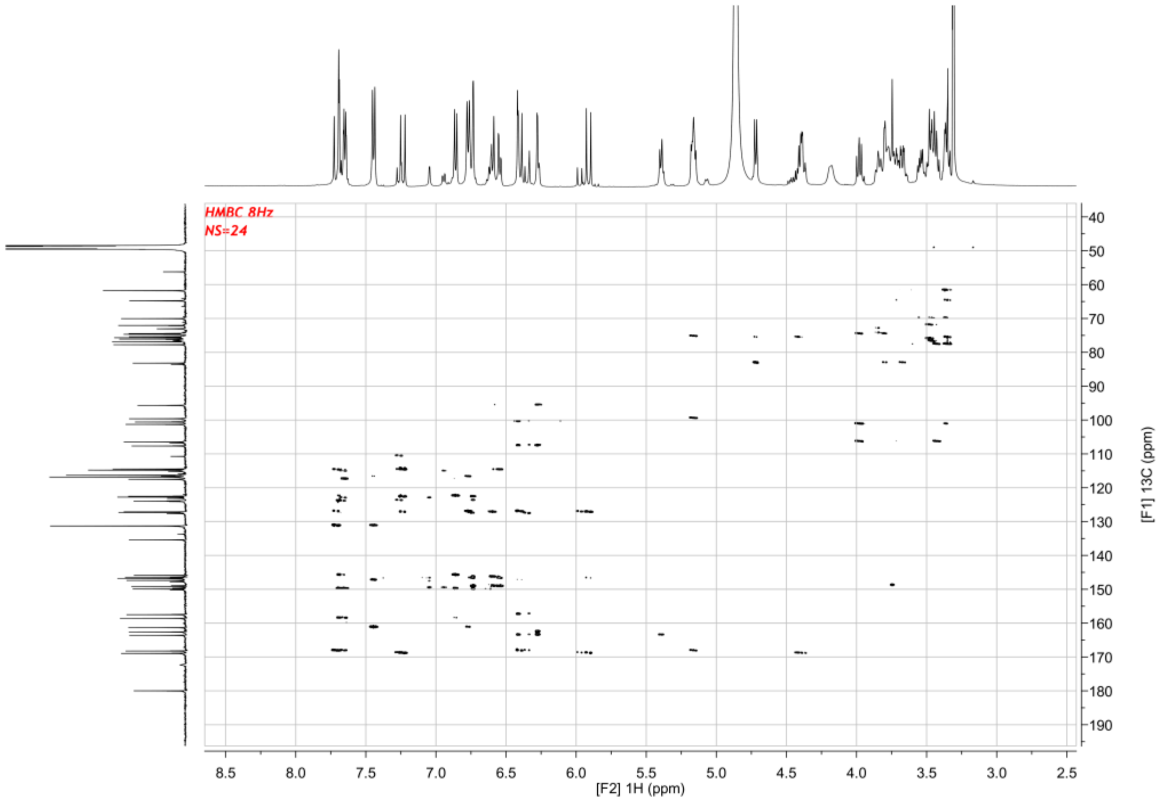

Figure S162. 1D TOCSY and 1D ROESY NMR subspectra of H-1$_{(7\text{-}O\text{-}\beta\text{-GlcA})}$ in compound **15** (methanol-d_4, 500.18 MHz).

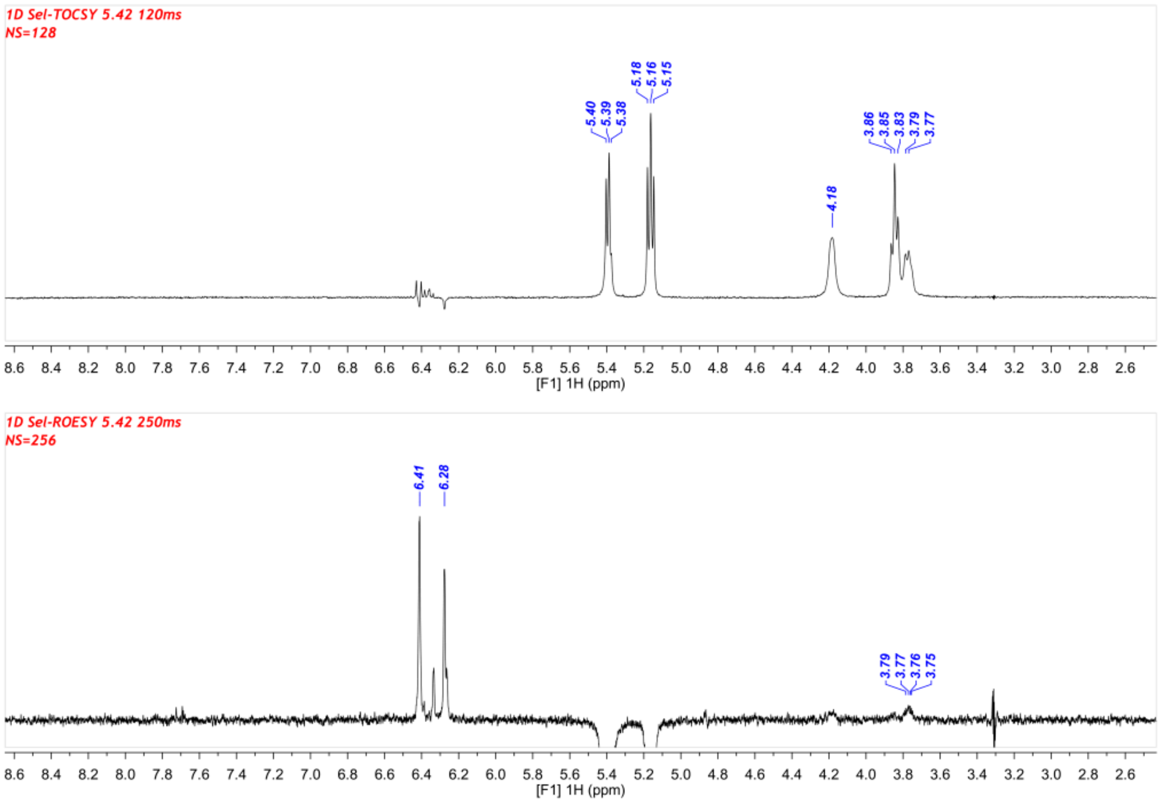

Figure S163. 1D TOCSY NMR subspectrum of H-2(3-*O*-β-Gal) in compound **15** (methanol-*d*4, 500.18 MHz).

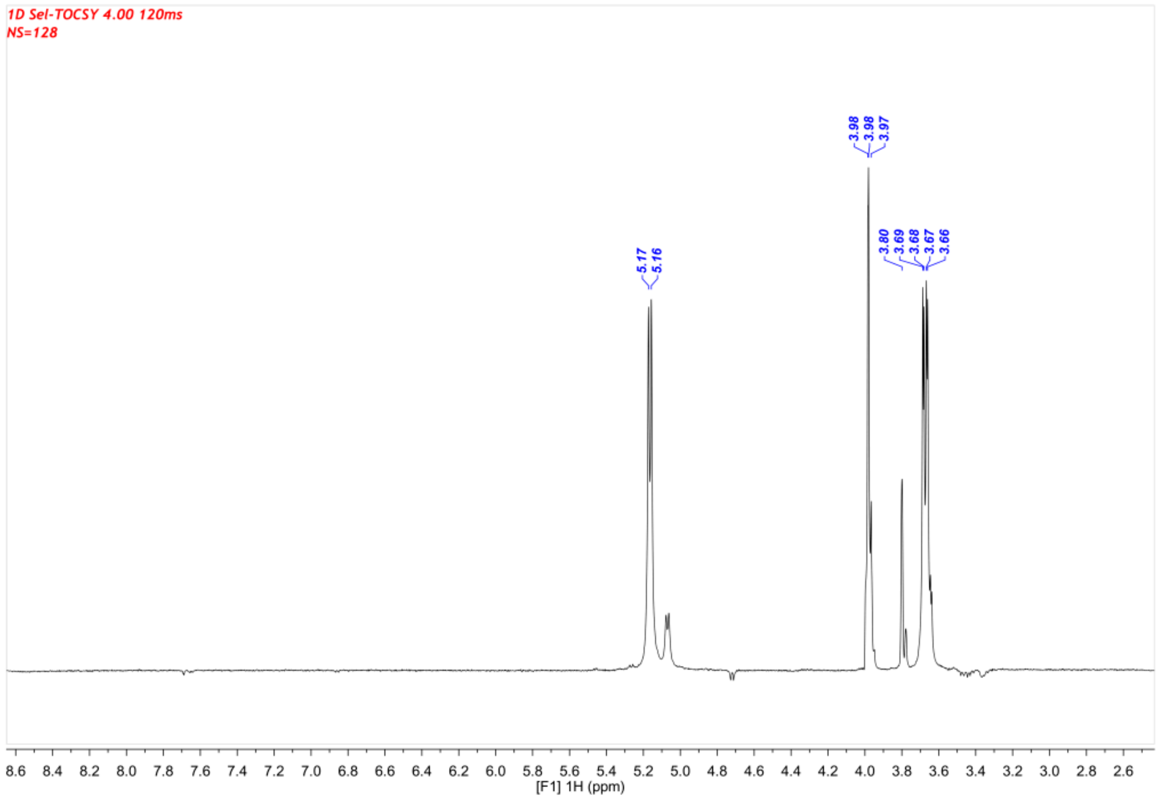

Figure S164. 1D TOCSY and 1D ROESY NMR subspectra of H-1(2Gal-*O*-β-Glc) in compound **15** (methanol-*d*4, 500.18 MHz).

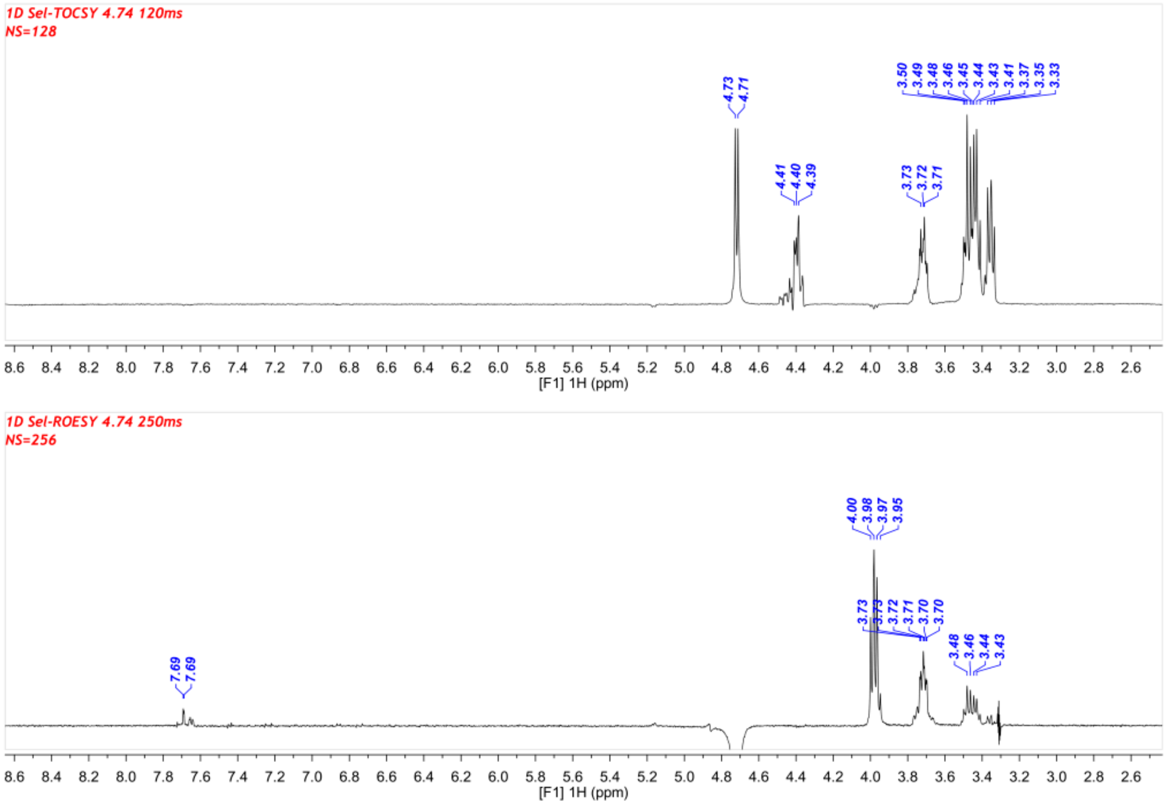

Compound 16

Figure S165. 1D ^{1}H-NMR spectrum of compound **16** (methanol-d_4, 500.18 MHz).

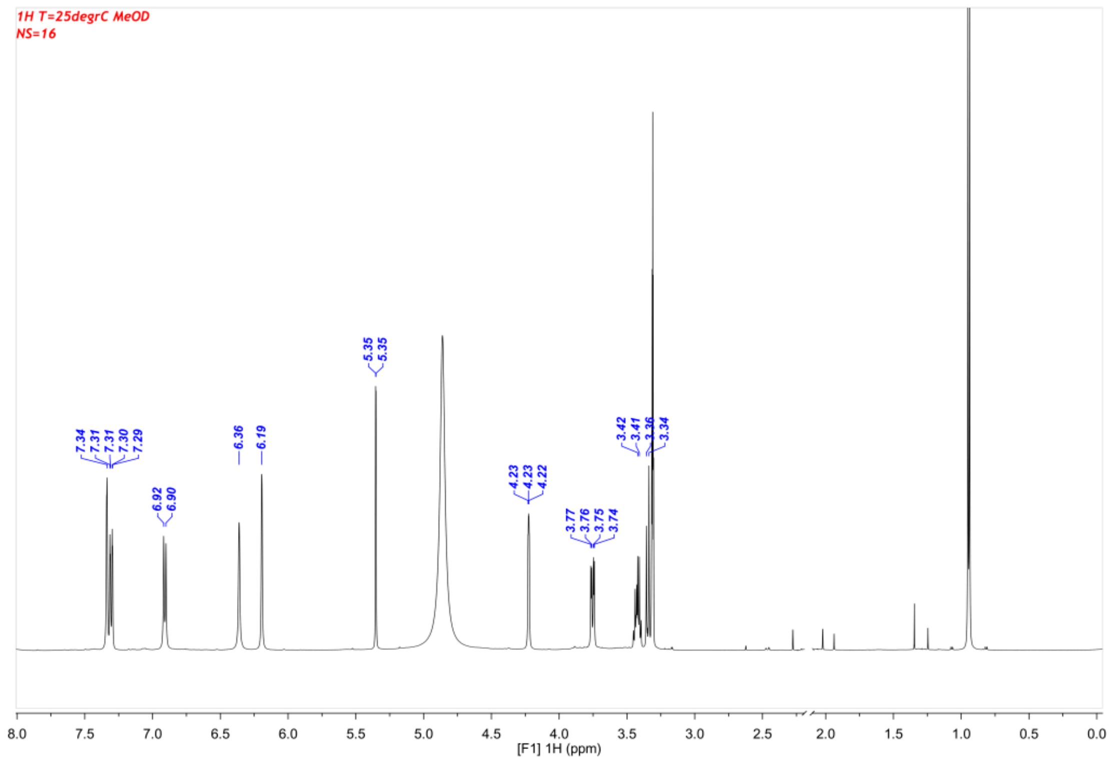

Figure S166. 1D ^{13}C-NMR spectra (1H decoupled and DEPT-135) of compound **16** (methanol-d_4, 125.77 MHz).

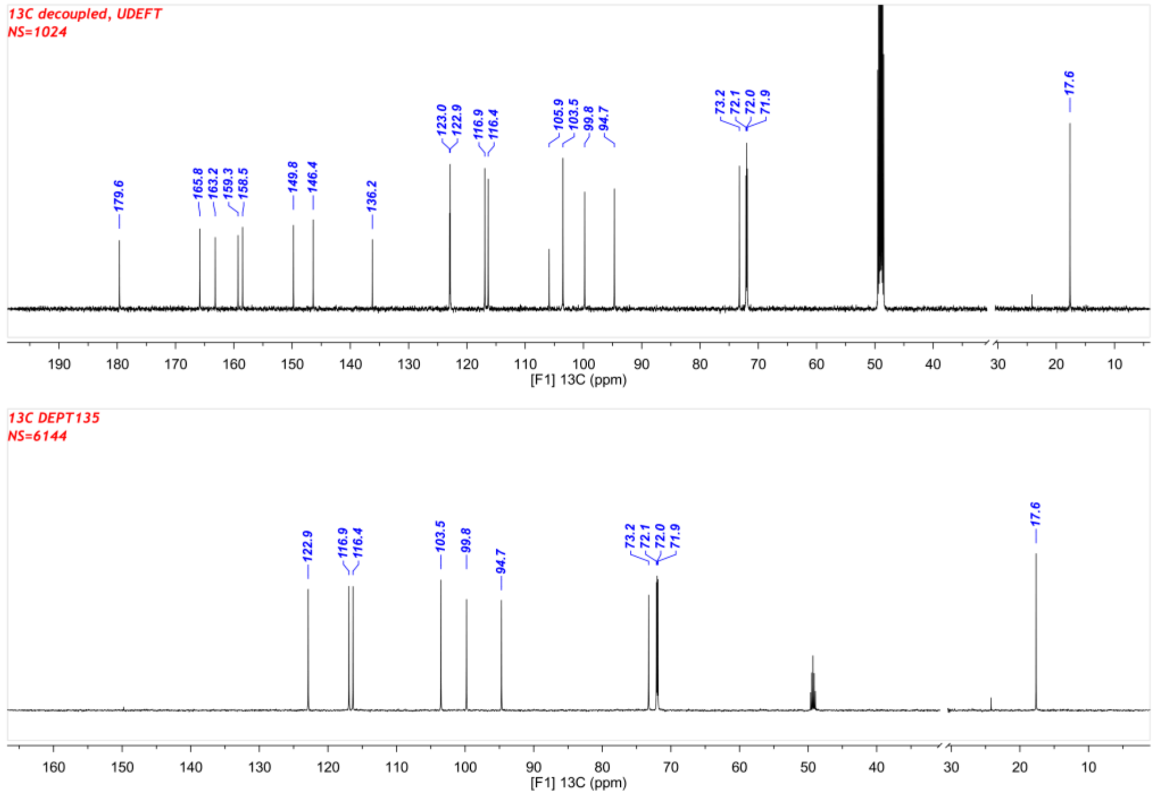

Figure S167. 2D *g*-COSY NMR spectrum of compound **16** (methanol-*d*4, 500.18 MHz).

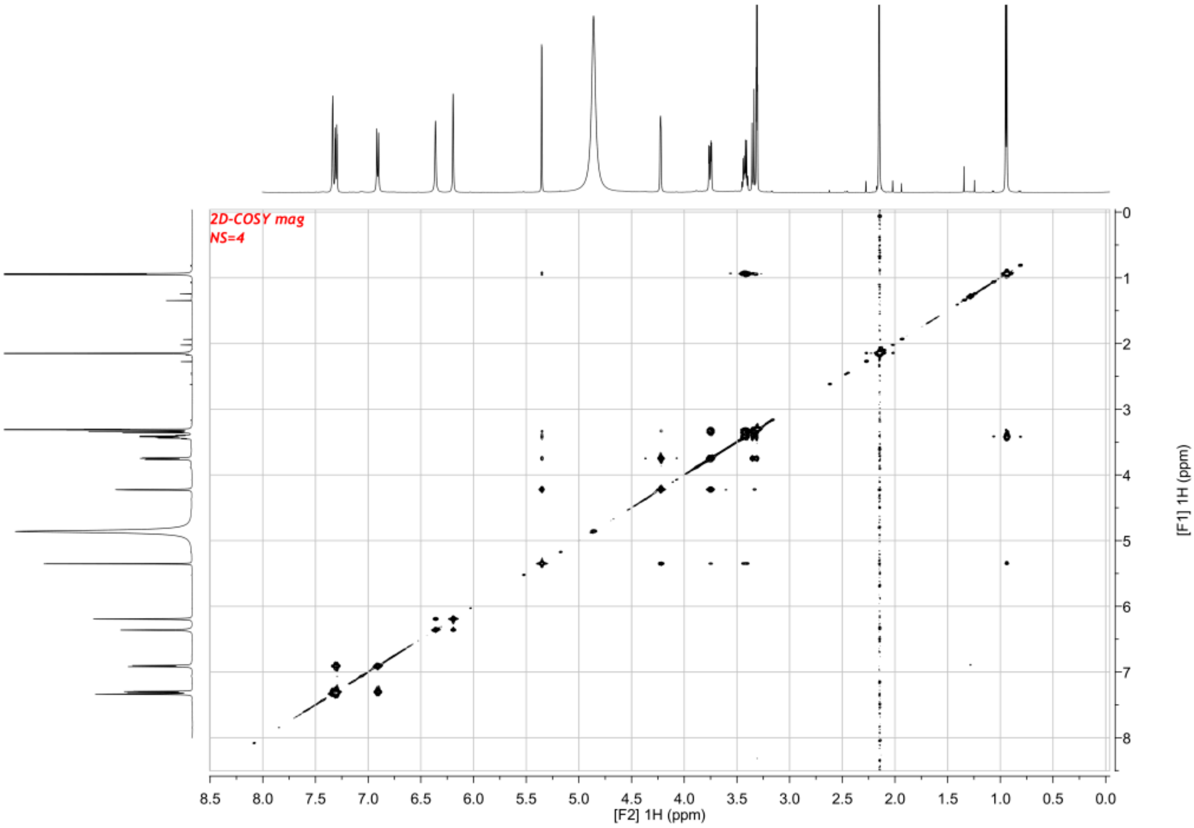

Figure S168. 2D ROESY NMR spectrum of compound **16** (methanol-*d*4, 500.18 MHz).

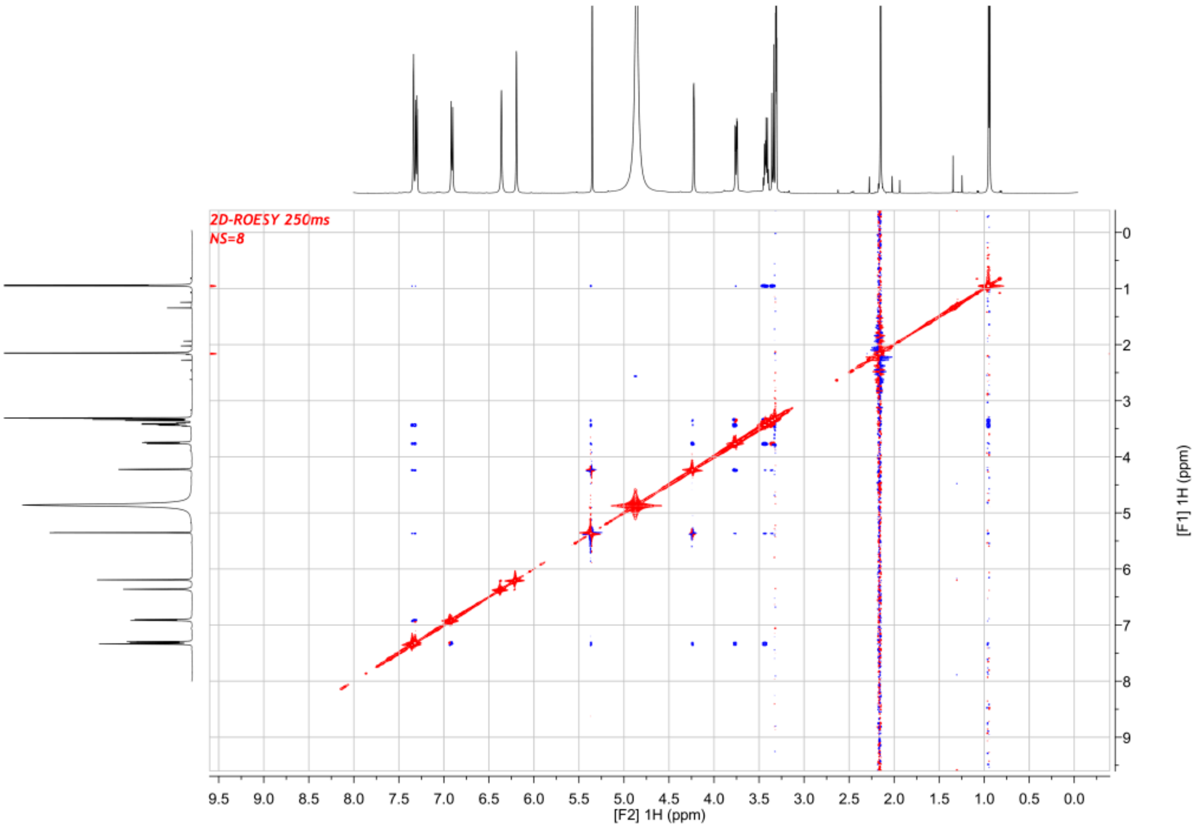

Figure S169. 2D *g*-HSQC-NMR spectrum of compound **16** (methanol-*d*4, 500.18 MHz, 125.77 MHz).

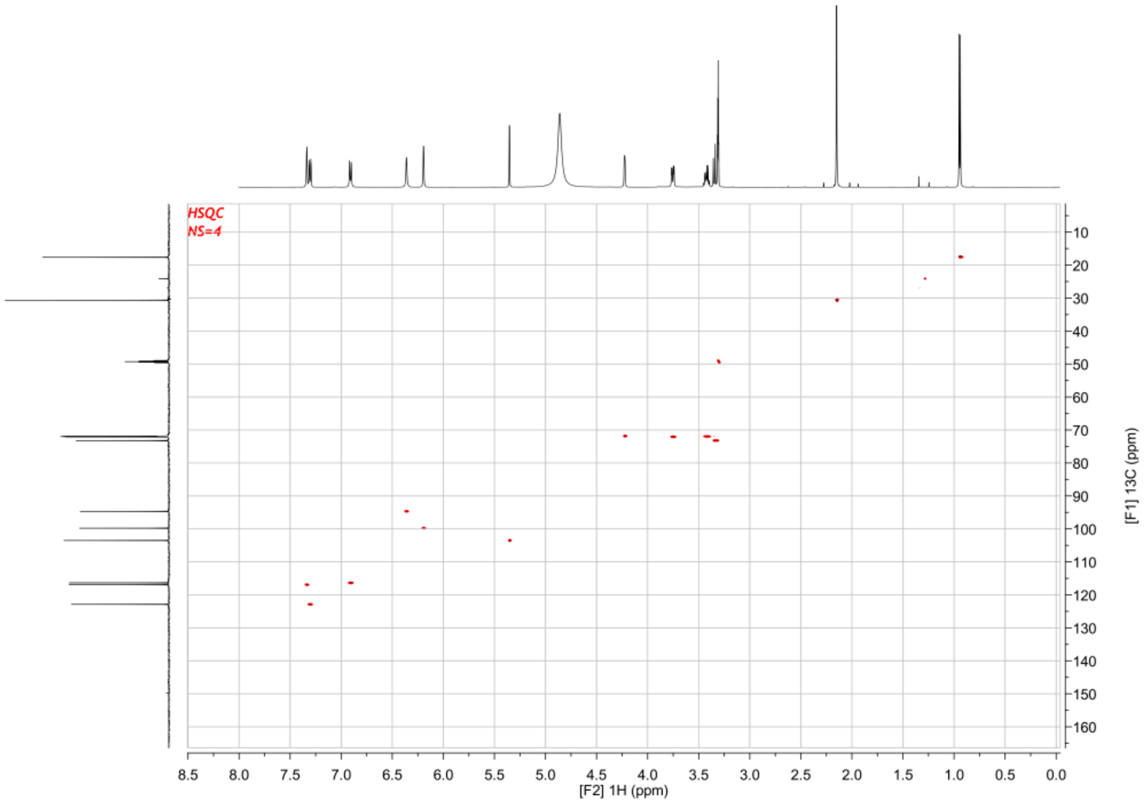

Figure S170. 2D *g*-HMBC-NMR spectrum of compound **16** (methanol-*d*4, 500.18 MHz, 125.77 MHz).

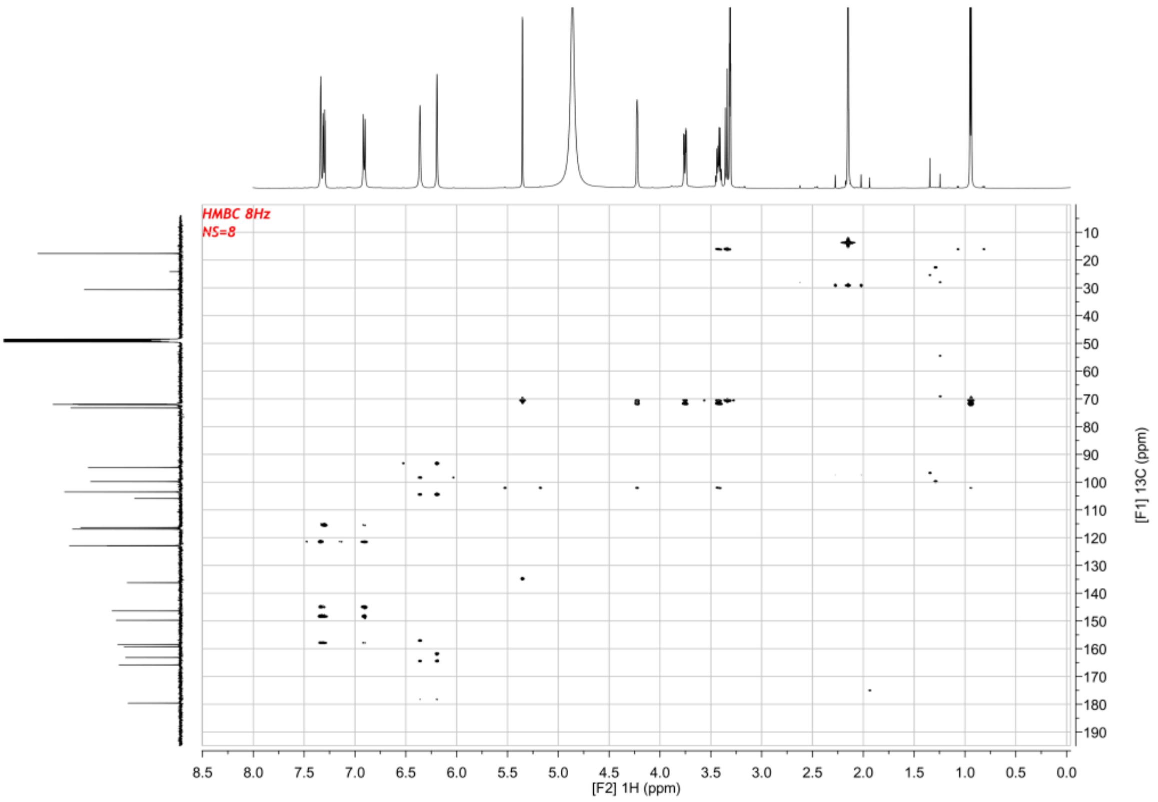

Figure S171. 1D TOCSY and 1D ROESY NMR subspectra of H-1$_{(3\text{-}O\text{-}\alpha\text{-Rha})}$ and H-6$_{(3\text{-}O\text{-}\alpha\text{-Rha})}$ in compound **16** (methanol-d_4, 500.18 MHz).

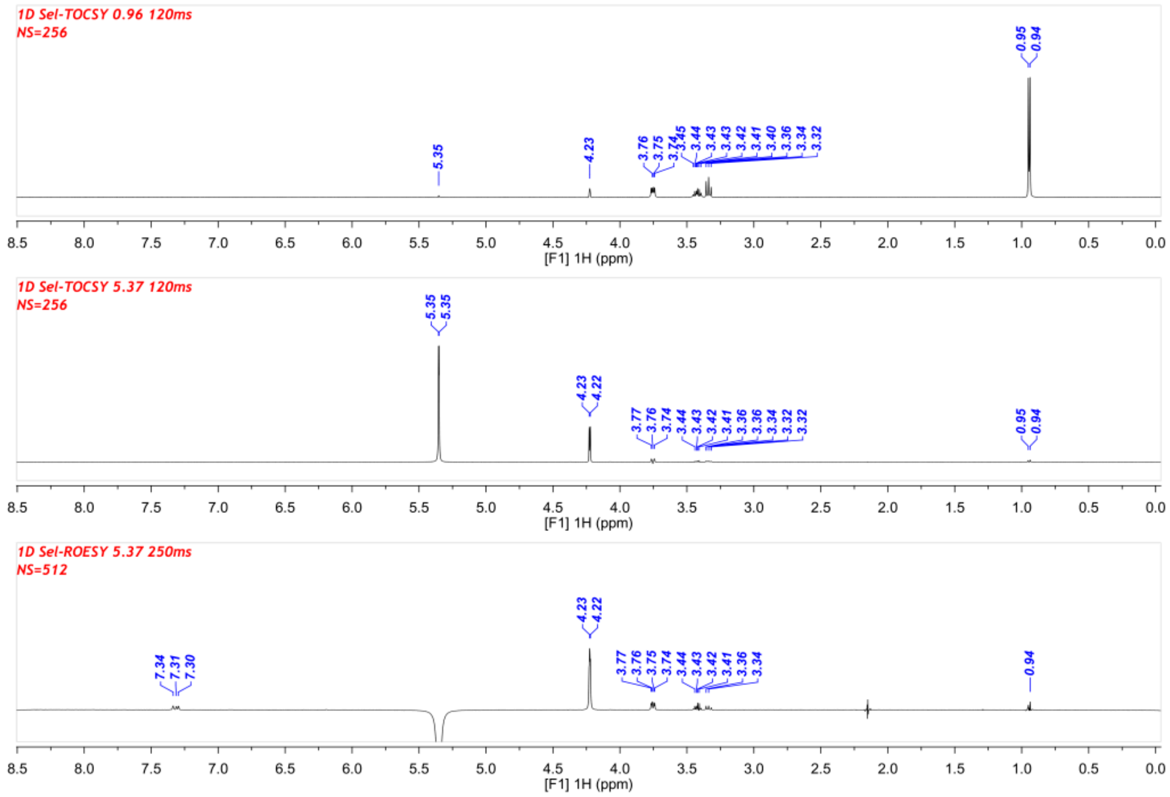

Compound 17

Figure S172. 1D ^{1}H-NMR spectrum of compound **17** (methanol-d_4, 500.18 MHz).

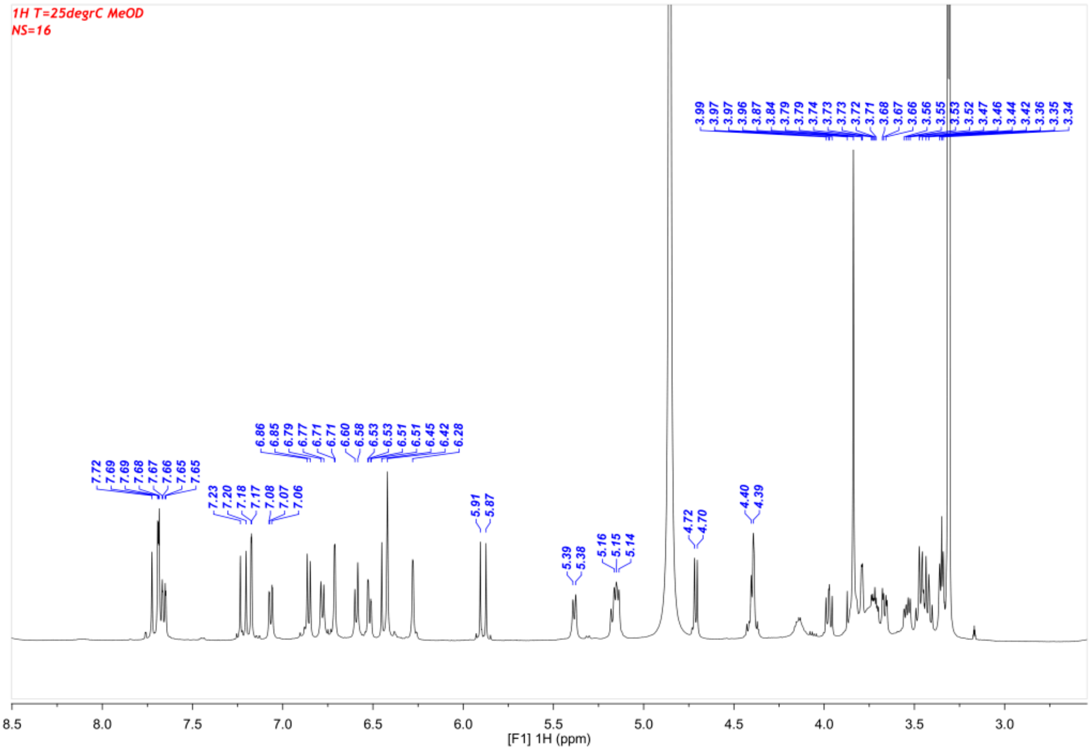

Figure S173. 1D ^{13}C-NMR spectra (1H decoupled and DEPT-135) of compound **17** (methanol-*d*4, 125.77 MHz).

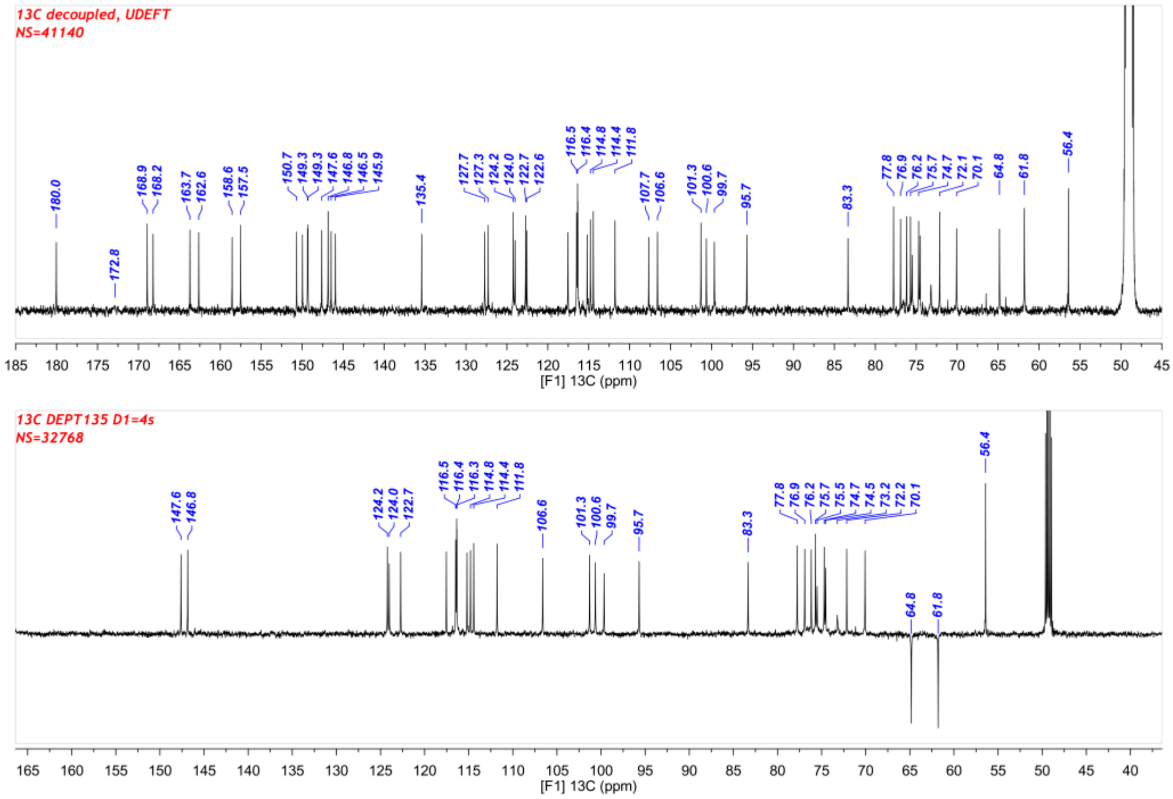

Figure S174. 2D *g*-COSY NMR spectrum of compound **17** (methanol-*d*4, 500.18 MHz).

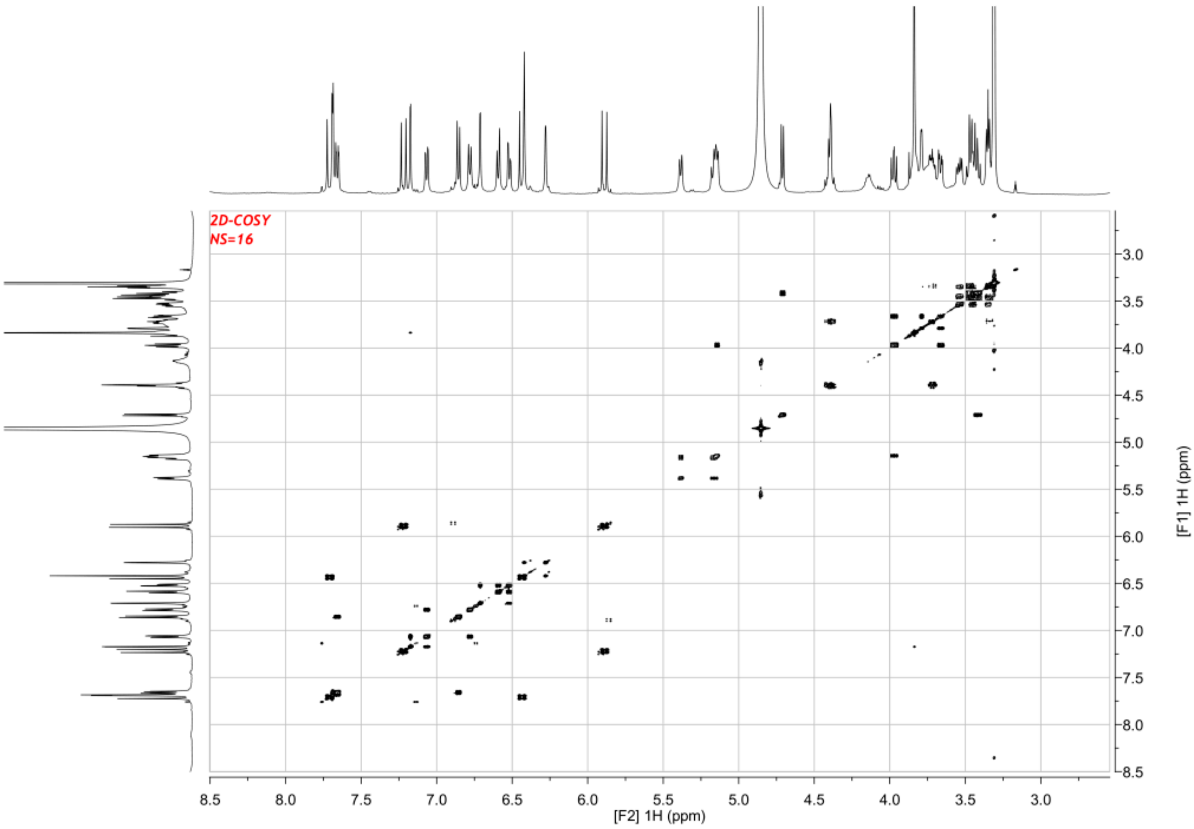

Figure S175. 2D TOCSY NMR spectrum of compound **17** (methanol-*d*4, 500.18 MHz).

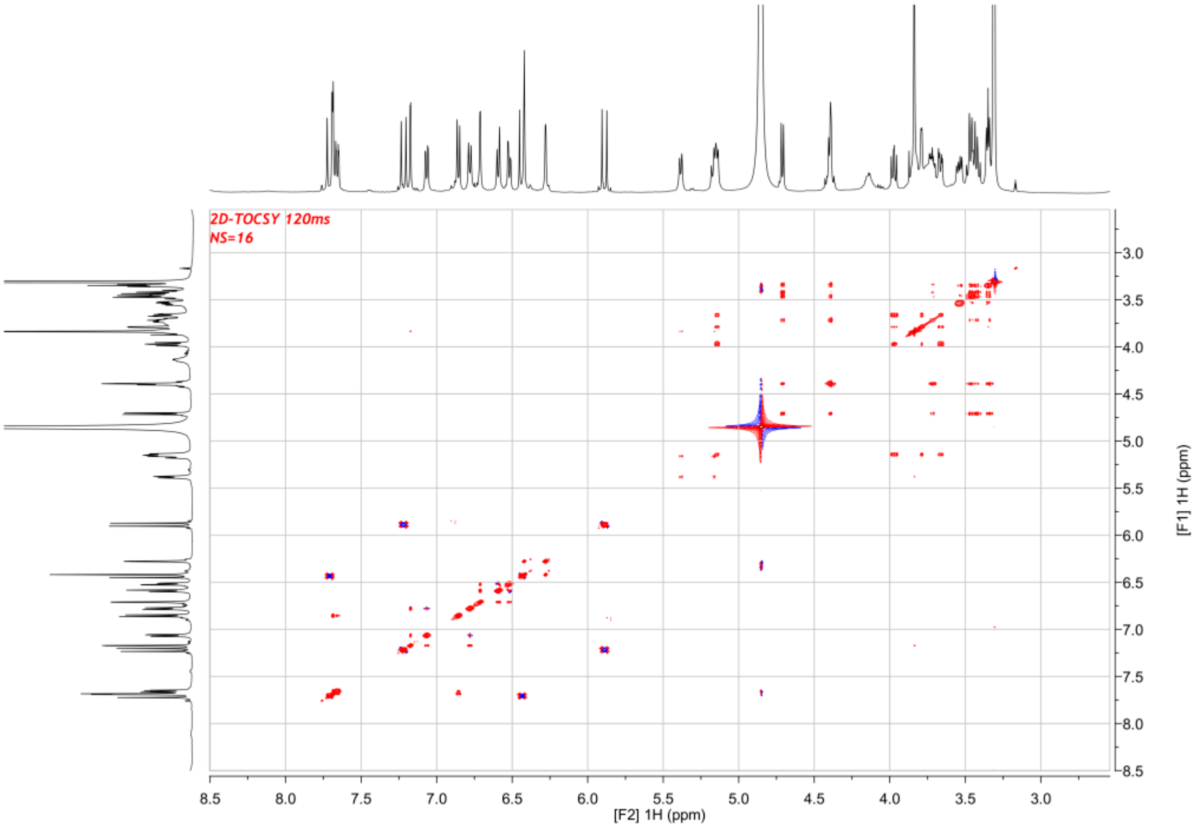

Figure S176. 2D ROESY NMR spectrum of compound **17** (methanol-*d*4, 500.18 MHz).

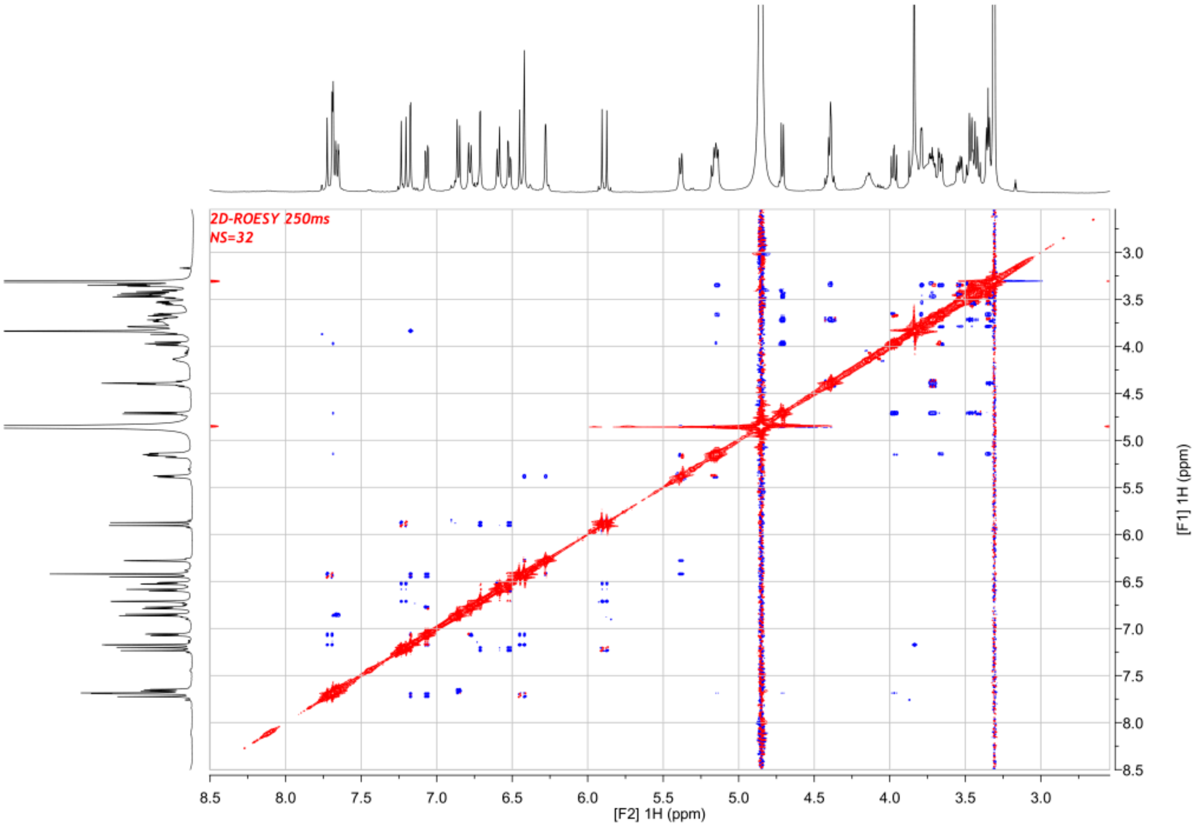

Figure S177. 2D *g*-HSQC-NMR spectrum of compound **17** (methanol-*d*4, 500.18 MHz, 125.77 MHz).

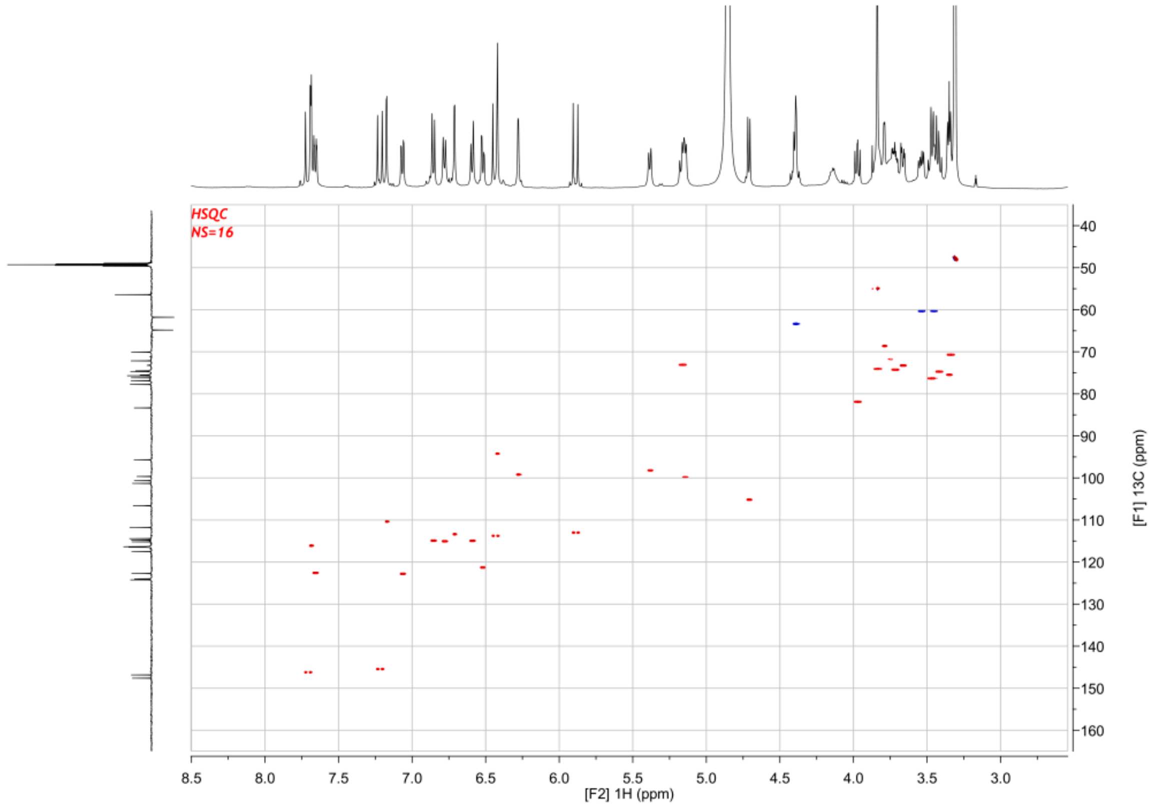

Figure S178. 2D *g*-HSQC-TOCSY NMR spectrum of compound **17** (methanol-*d*4, 500.18 MHz, 125.77 MHz).

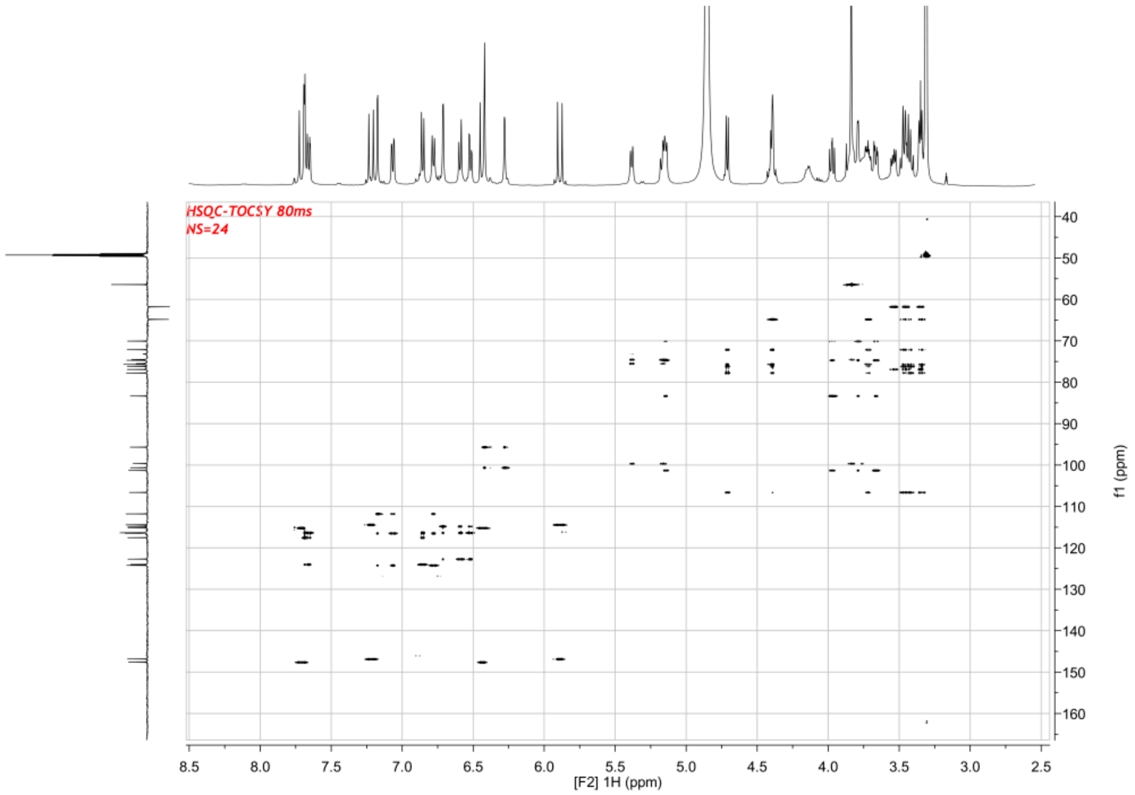

Figure S179. 2D *g*-HMBC-NMR spectrum of compound **17** (methanol-d_4, 500.18 MHz, 125.77 MHz).

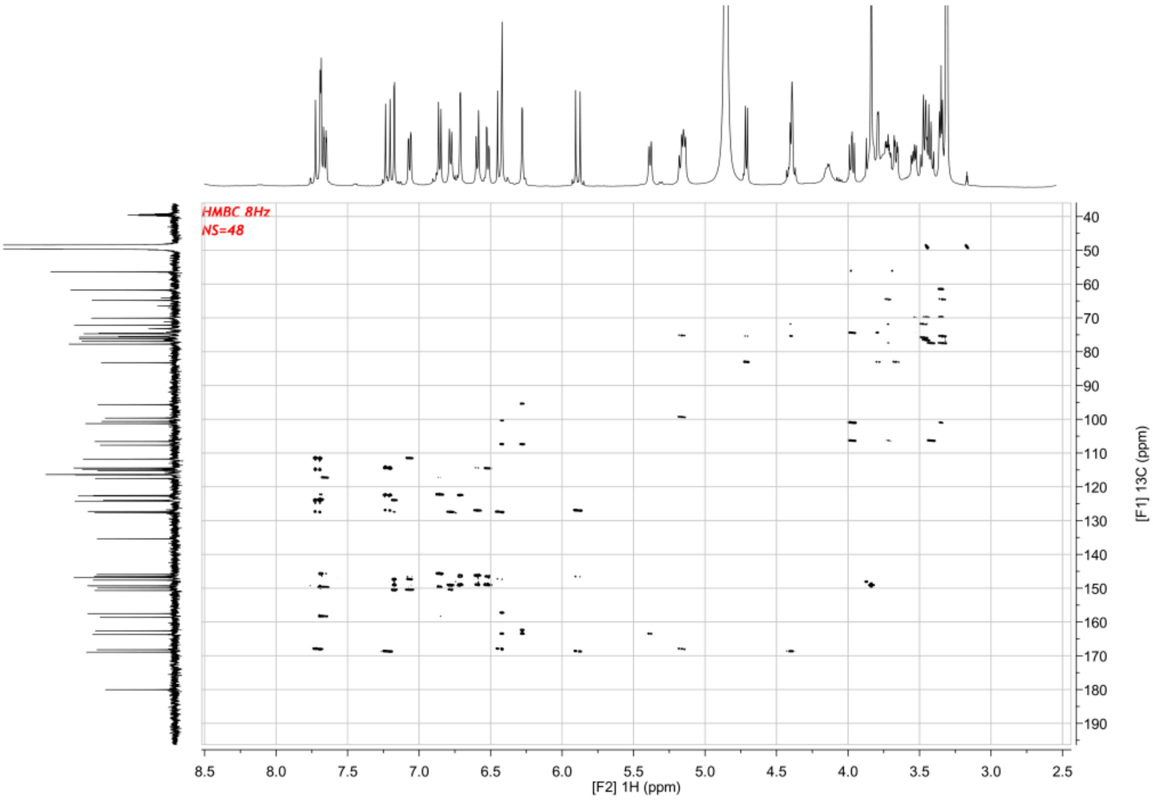

Figure S180. 1D TOCSY and 1D ROESY NMR subspectra of H-1(7-*O*-β-GlcA) in compound **17** (methanol-d_4, 500.18 MHz).

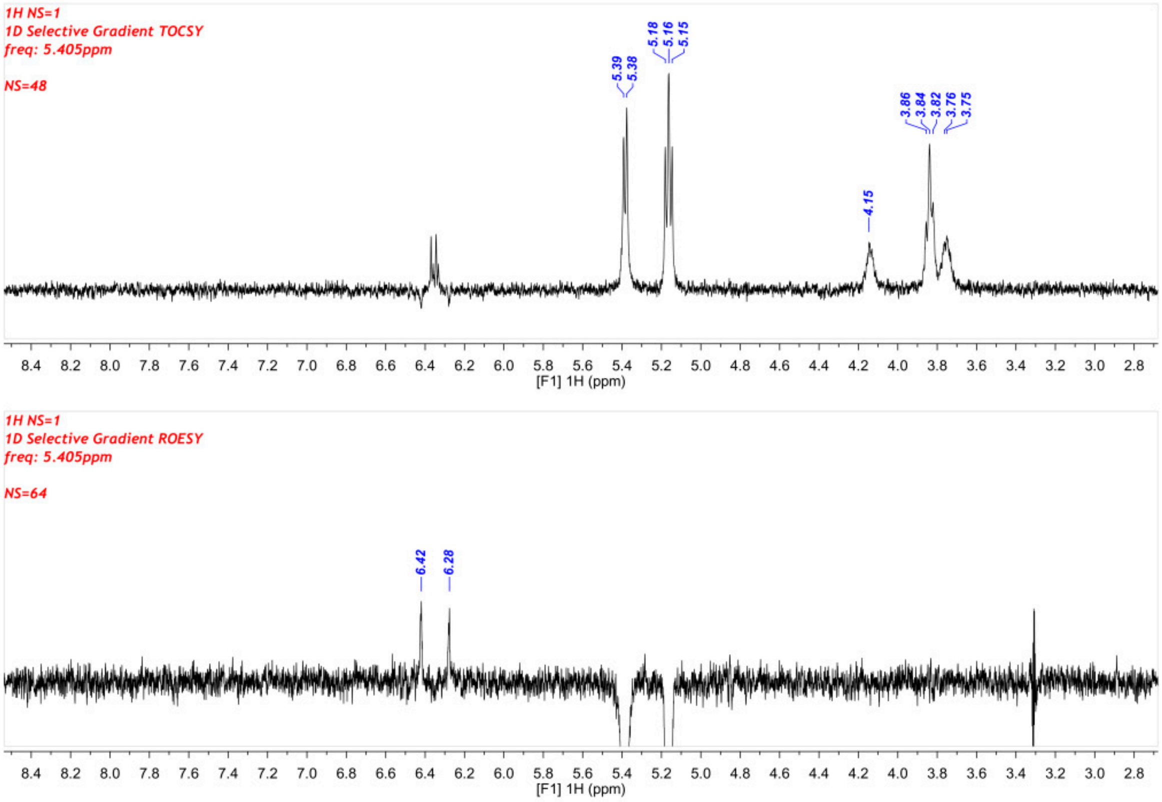

Figure S181. 1D TOCSY subspectrum of H-2$_{(3\text{-}O\text{-}\beta\text{-Gal})}$ in compound **17** (methanol-d_4, 500.18 MHz).

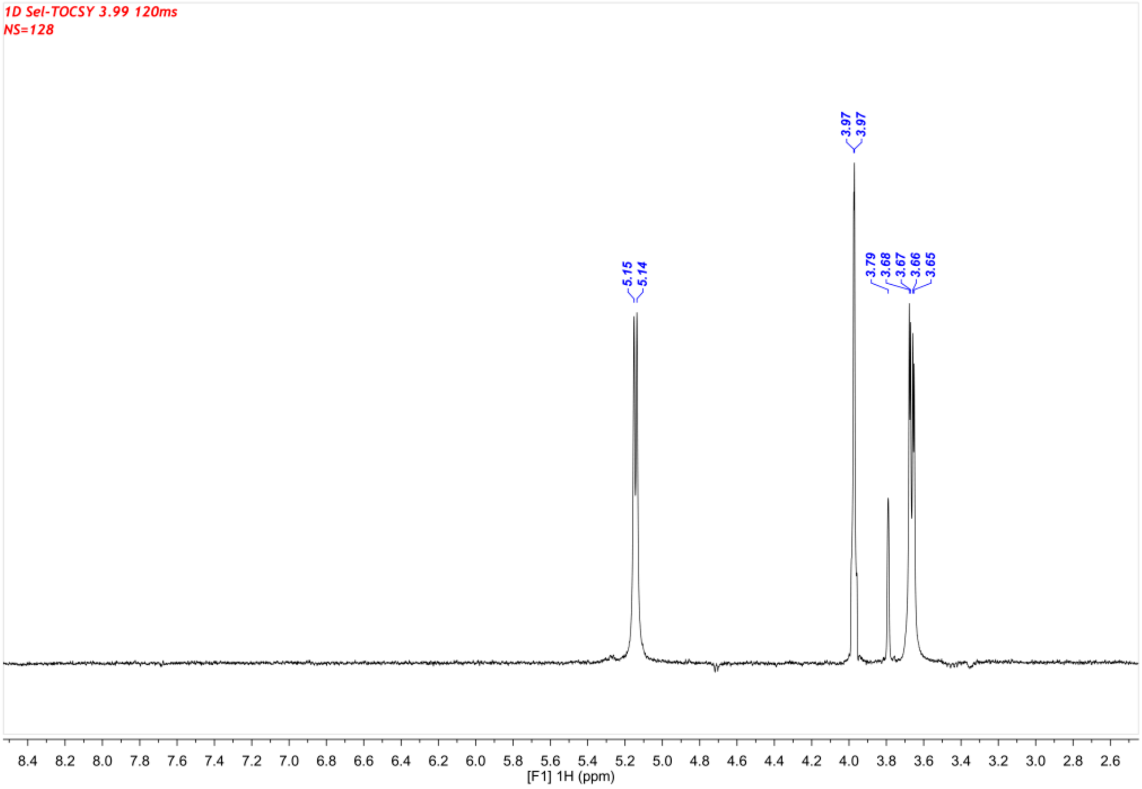

Figure S182. 1D TOCSY and 1D ROESY NMR subspectra of H-1$_{(2^{Gal}\text{-}O\text{-}\beta\text{-Glc})}$ in compound **17** (methanol-d_4, 500.18 MHz).

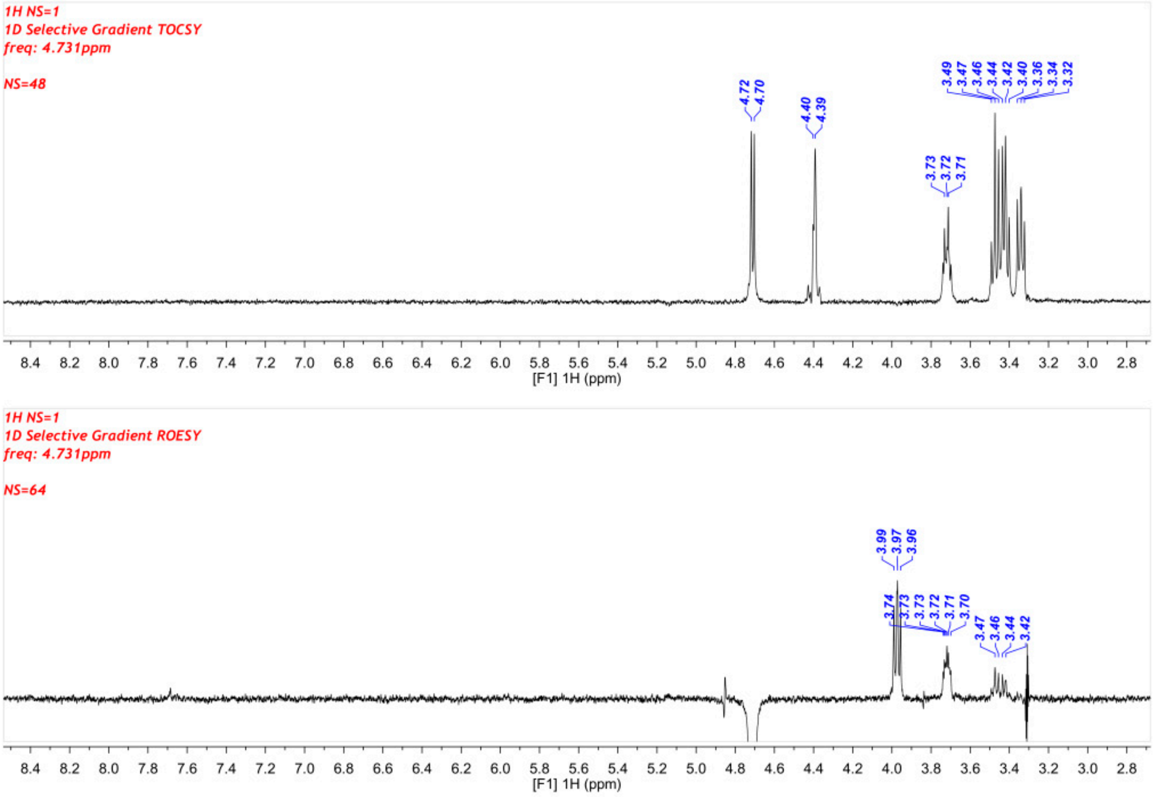

Compound 18

Figure S183. 1D ^{1}H-NMR spectrum of compound **18** (methanol-*d*4, 500.18 MHz).

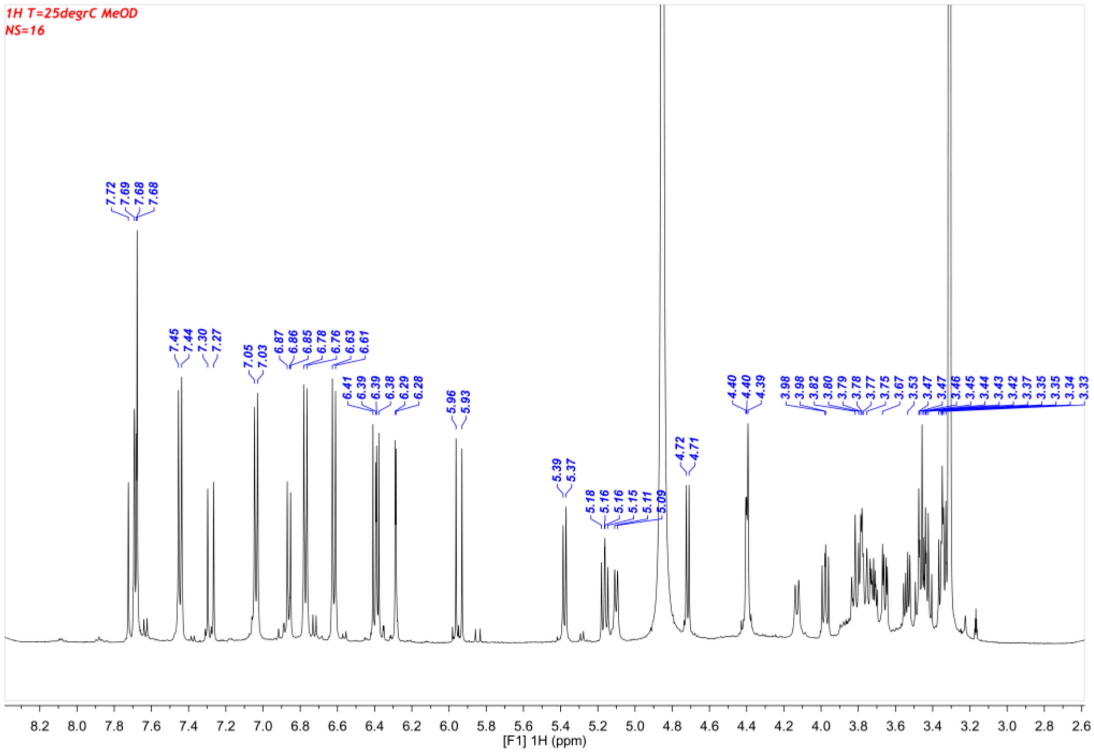

Figure S184. 1D ^{13}C-NMR spectra (1H decoupled and DEPT-135) of compound **18** (methanol-*d*4, 125.77 MHz).

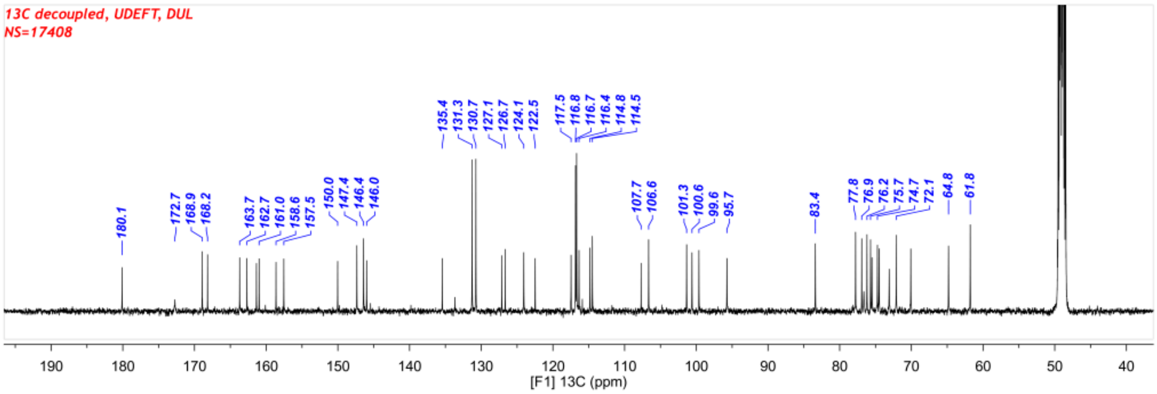

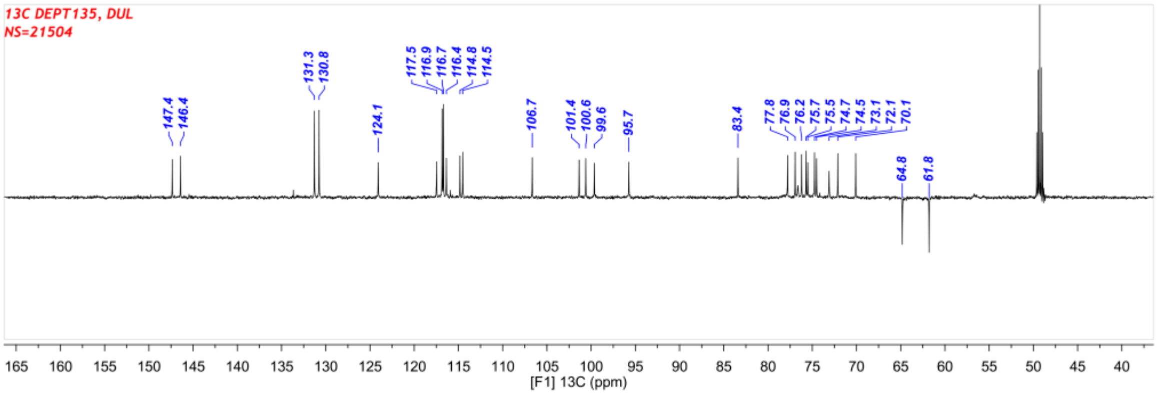

Figure S185. 2D *g*-COSY NMR spectrum of compound **18** (methanol-d_4, 500.18 MHz).

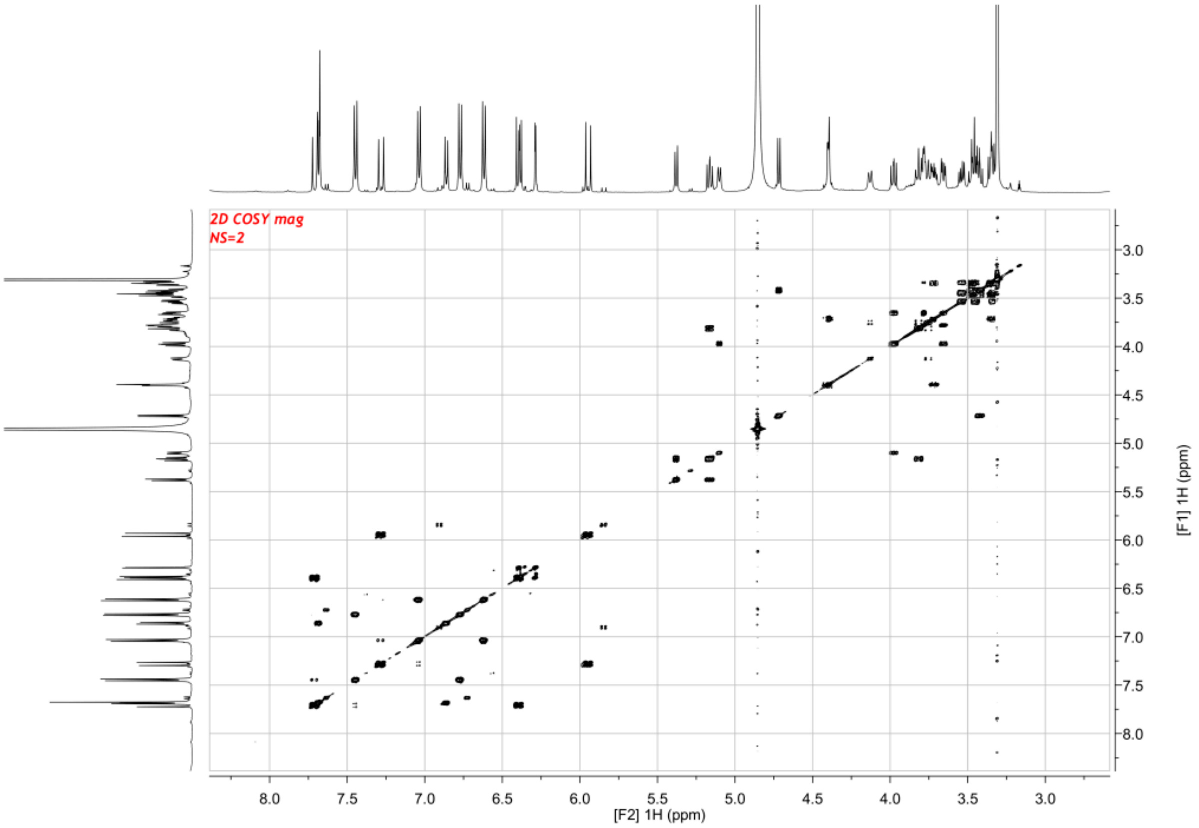

Figure S186. 2D TOCSY NMR spectrum of compound **18** (methanol-d_4, 500.18 MHz).

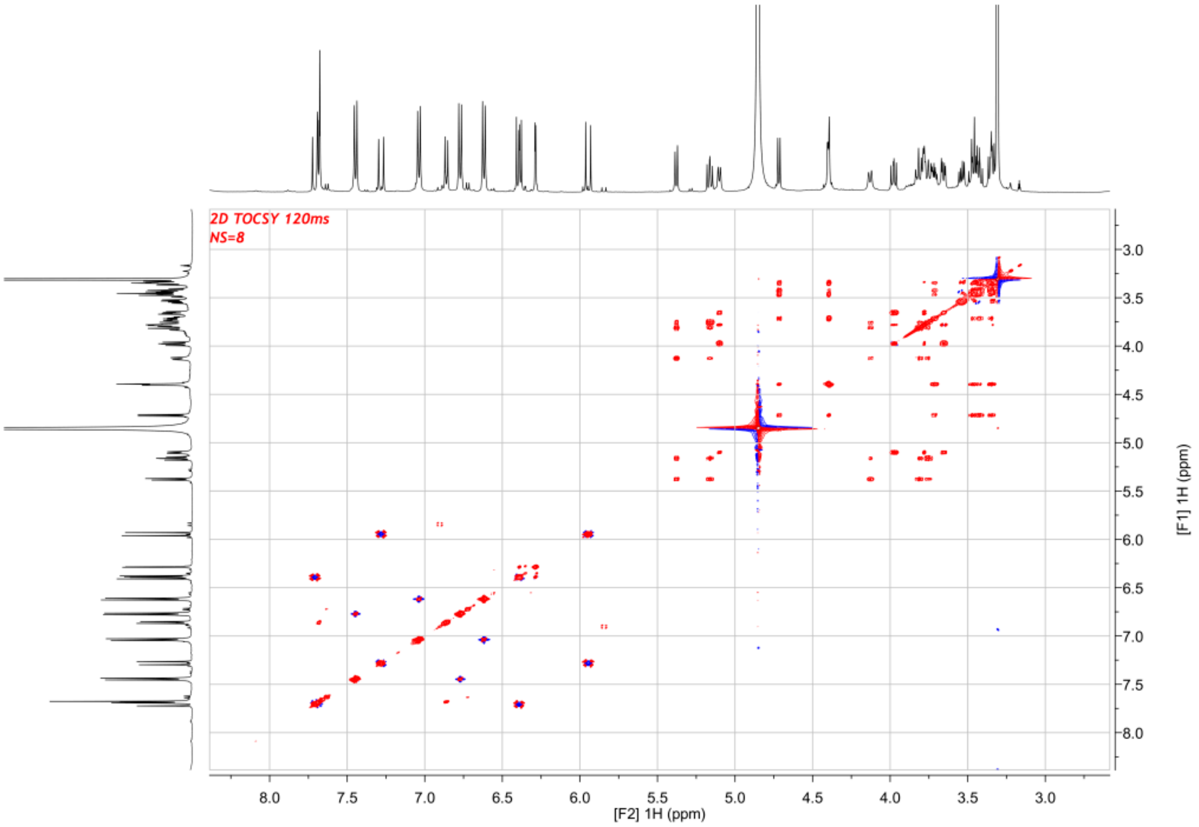

Figure S187. 2D ROESY NMR spectrum of compound **18** (methanol-d_4, 500.18 MHz).

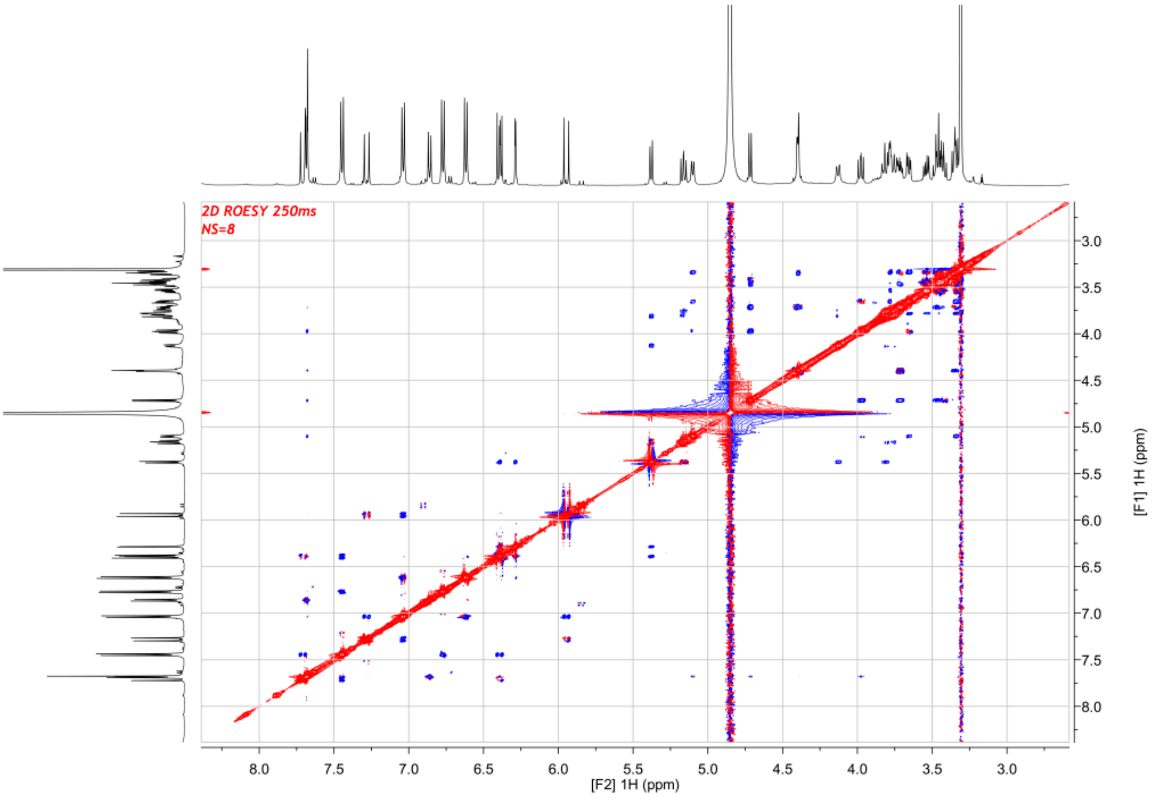

Figure S188. 2D *g*-HSQC-NMR spectrum of compound **18** (methanol-d_4, 500.18 MHz, 125.77 MHz).

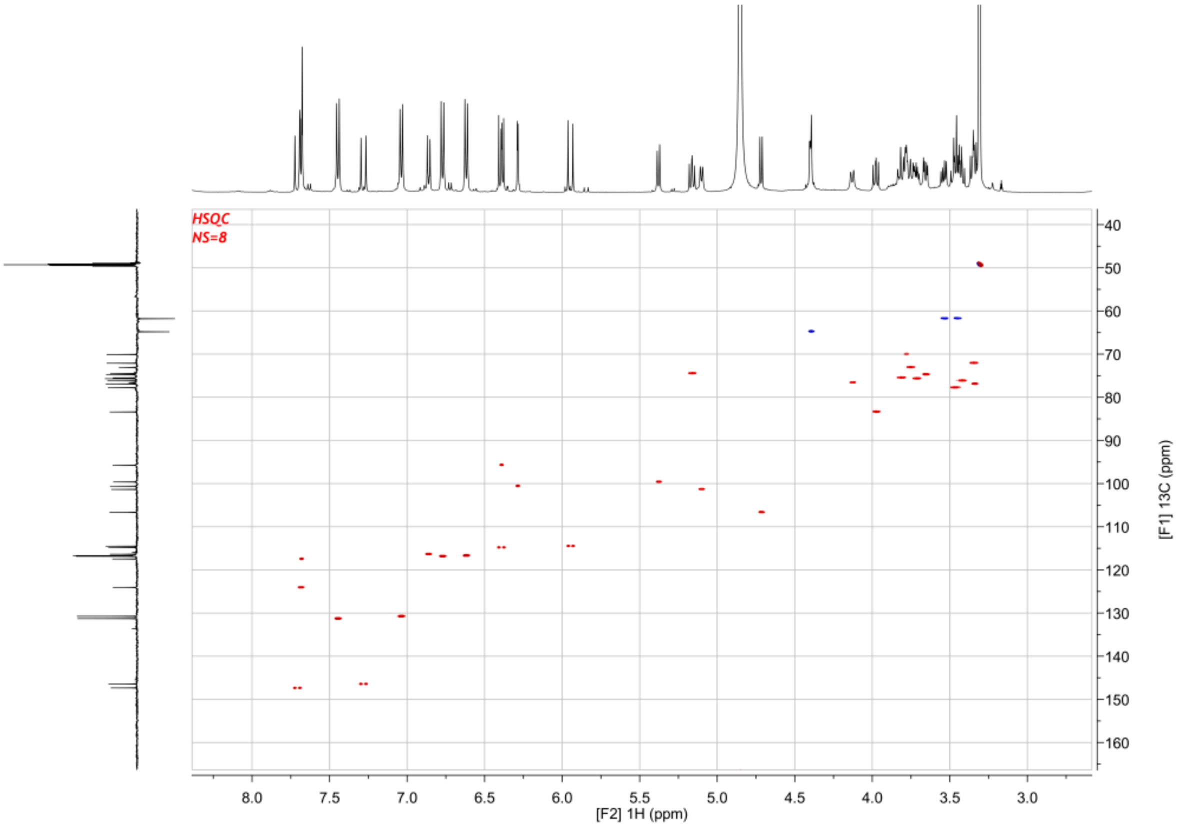

Figure S189. 2D *g*-HSQC-TOCSY NMR spectrum of compound **18** (methanol-d_4, 500.18 MHz, 125.77 MHz).

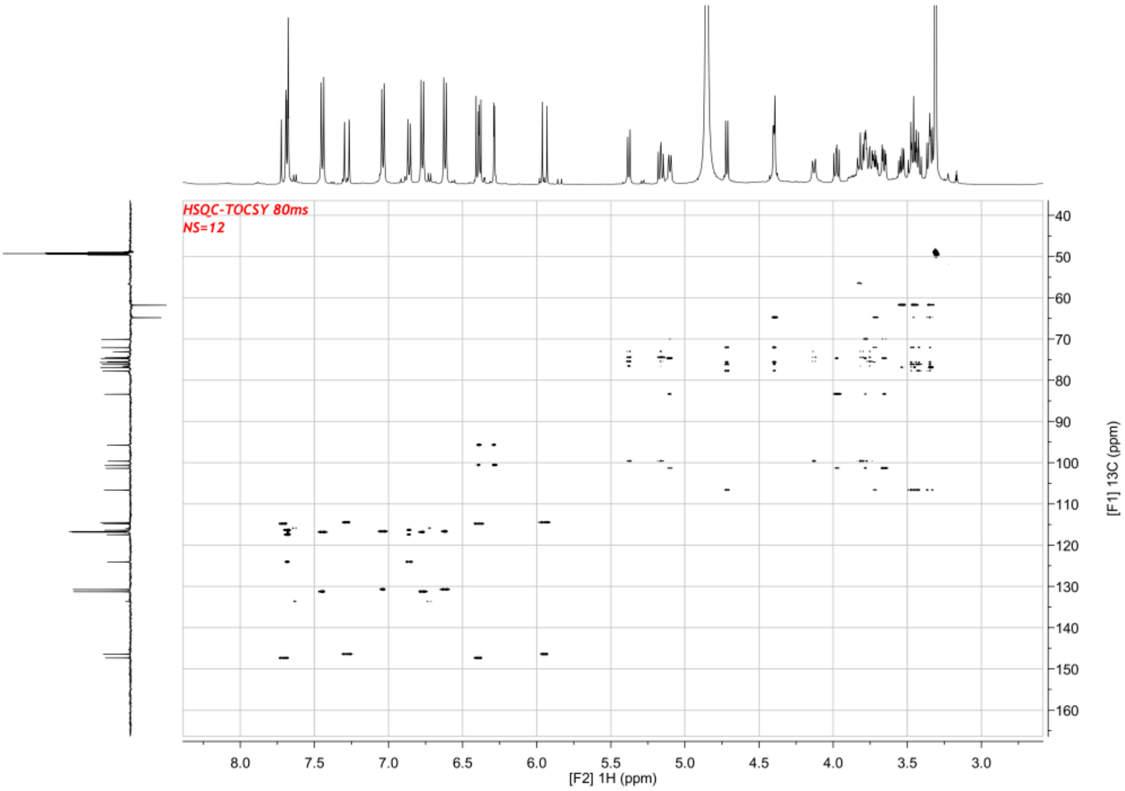

Figure S190. 2D *g*-HMBC-NMR spectrum of compound **18** (methanol-d_4, 500.18 MHz, 125.77 MHz).

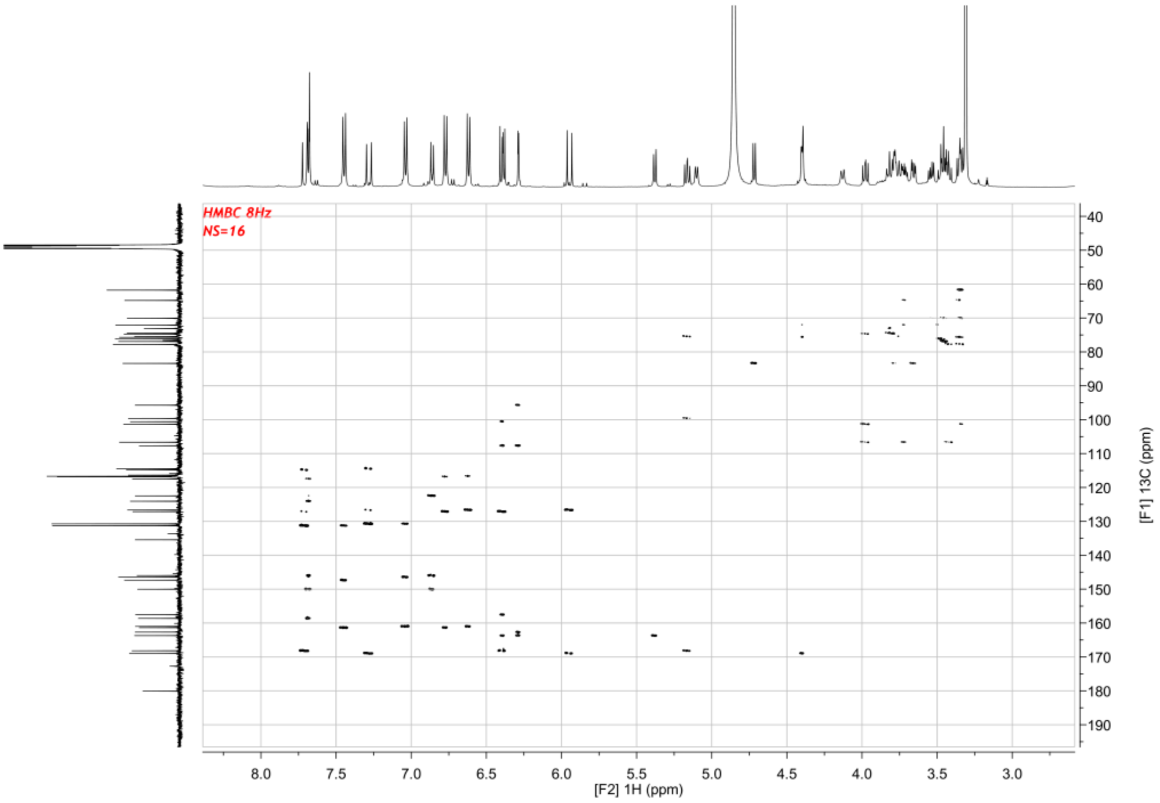

Figure S191. 1D TOCSY and 1D ROESY NMR subspectra of H-1(7-*O*-β-GlcA) in compound **18** (methanol-*d*4, 500.18 MHz).

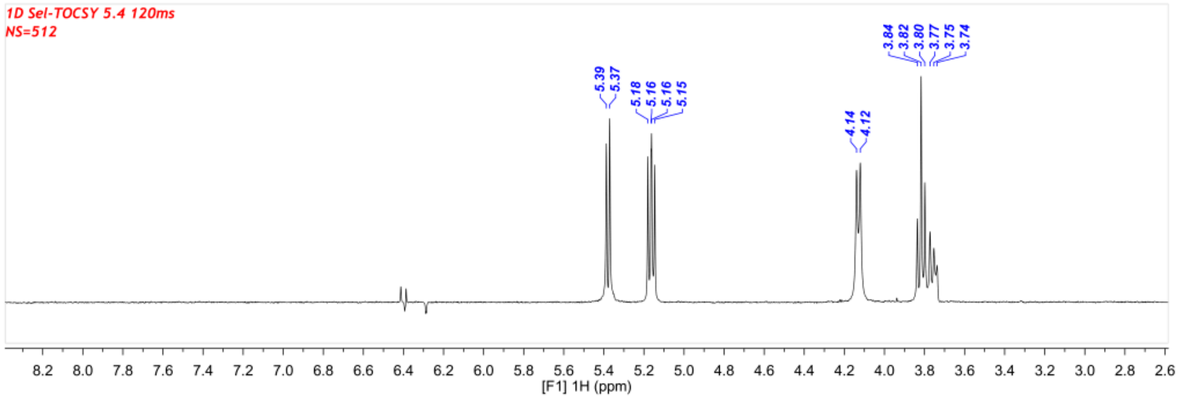

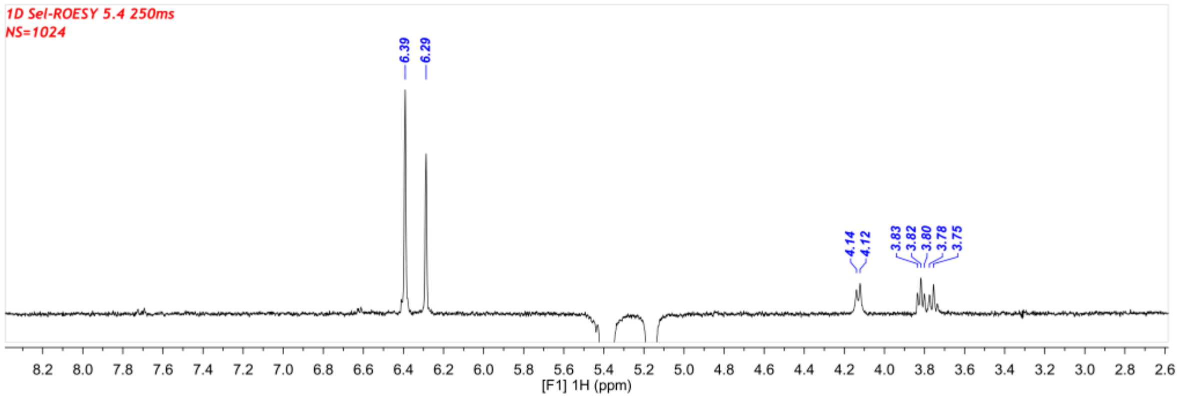

Figure S192. 1D TOCSY and 1D ROESY NMR subspectra of H-1(3-*O*-β-Gal) in compound **18** (methanol-*d*4, 500.18 MHz).

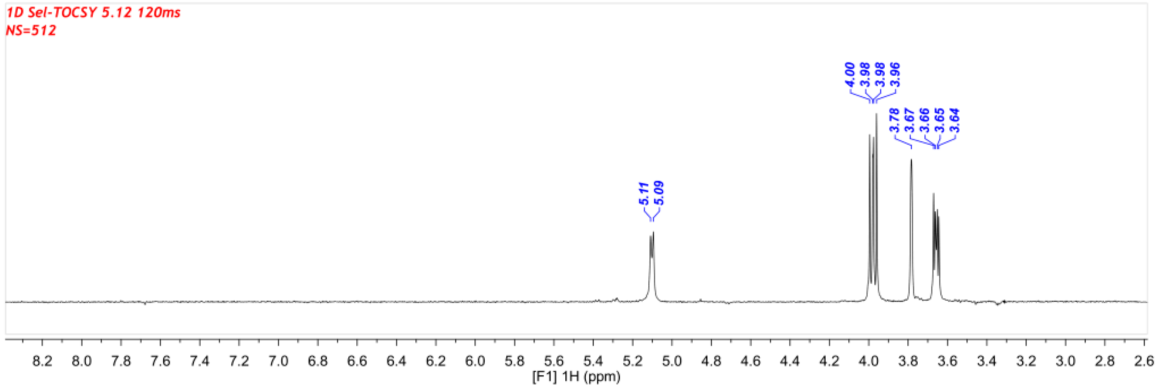

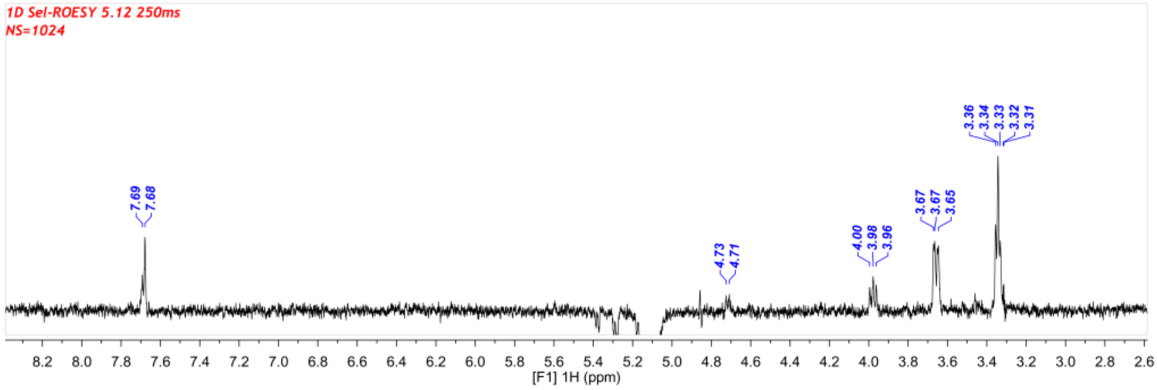

Figure S193. 1D TOCSY and 1D ROESY NMR subspectra of H-1(2^{Gal}-*O*-β-Glc) in compound **18** (methanol-d_4, 500.18 MHz).

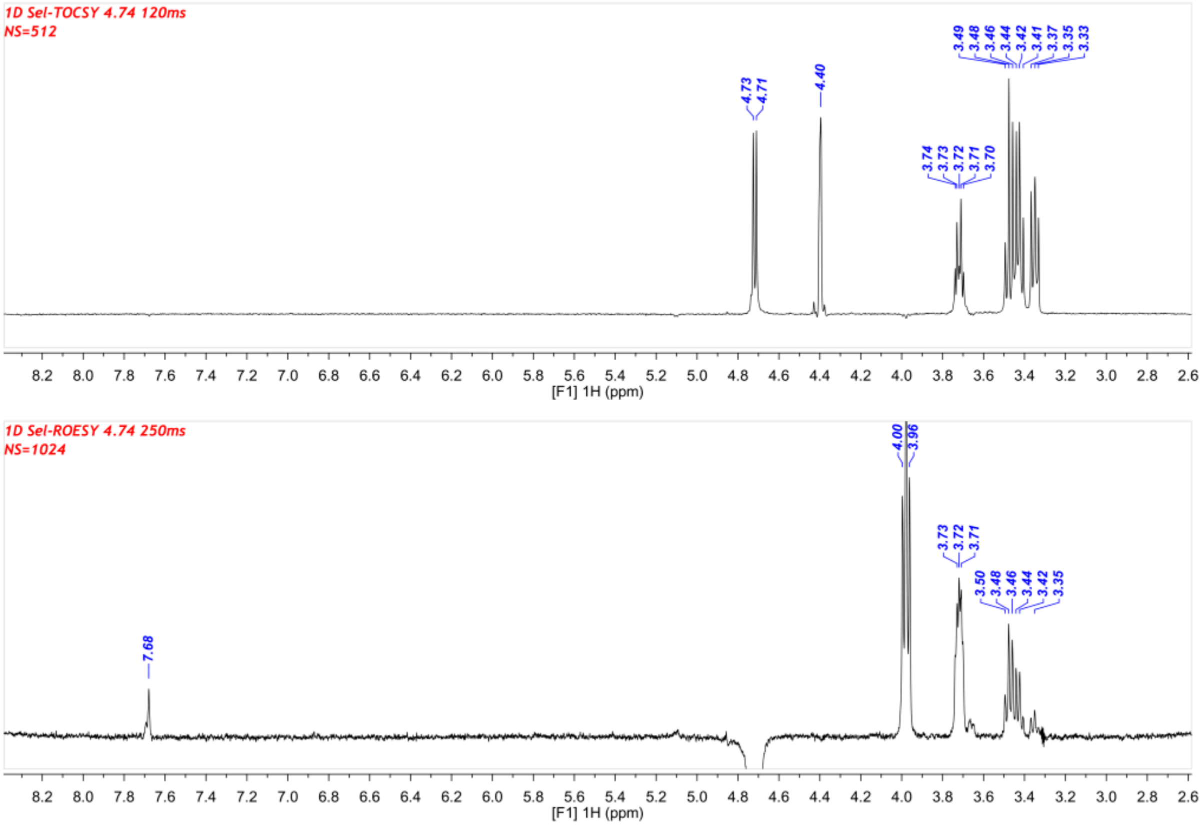

Figure S194. UPLC-MS chromatograms of phenolic acid reference standards.

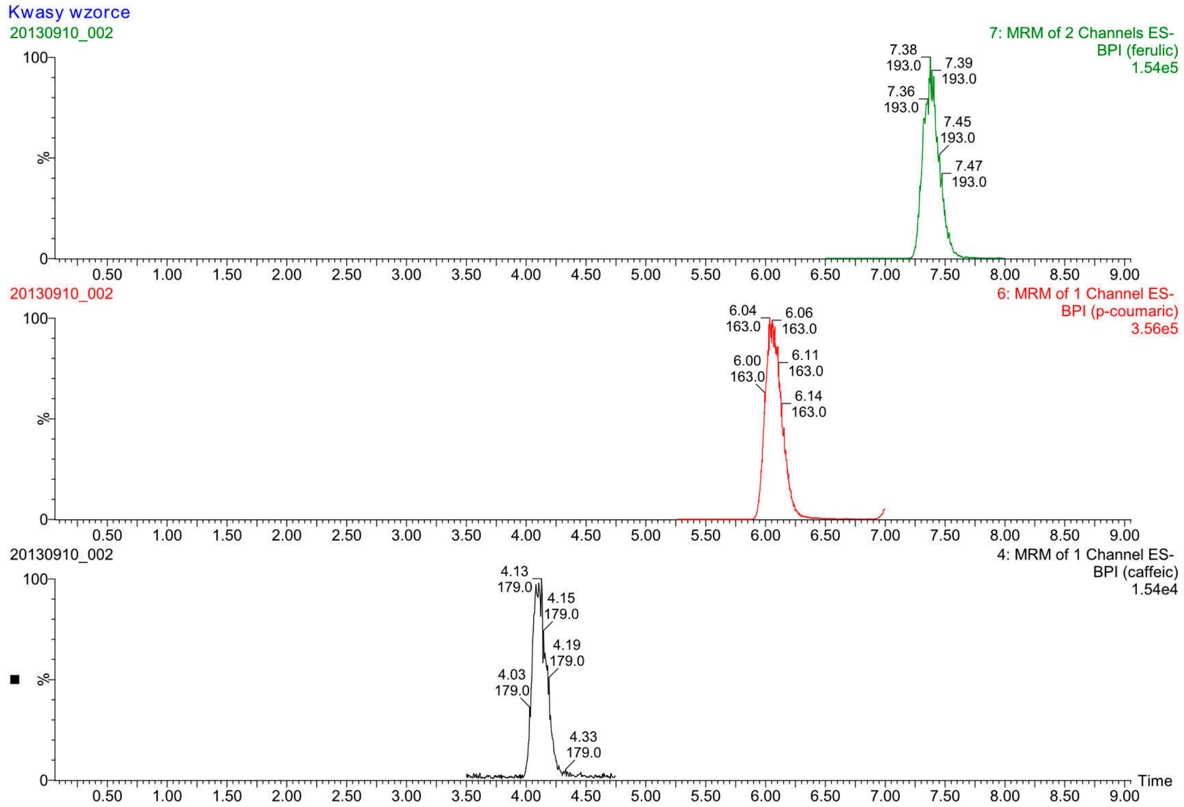

Figure S195. UPLC-MS chromatograms of phenolic acids released during alkaline hydrolysis of the compound **4**.

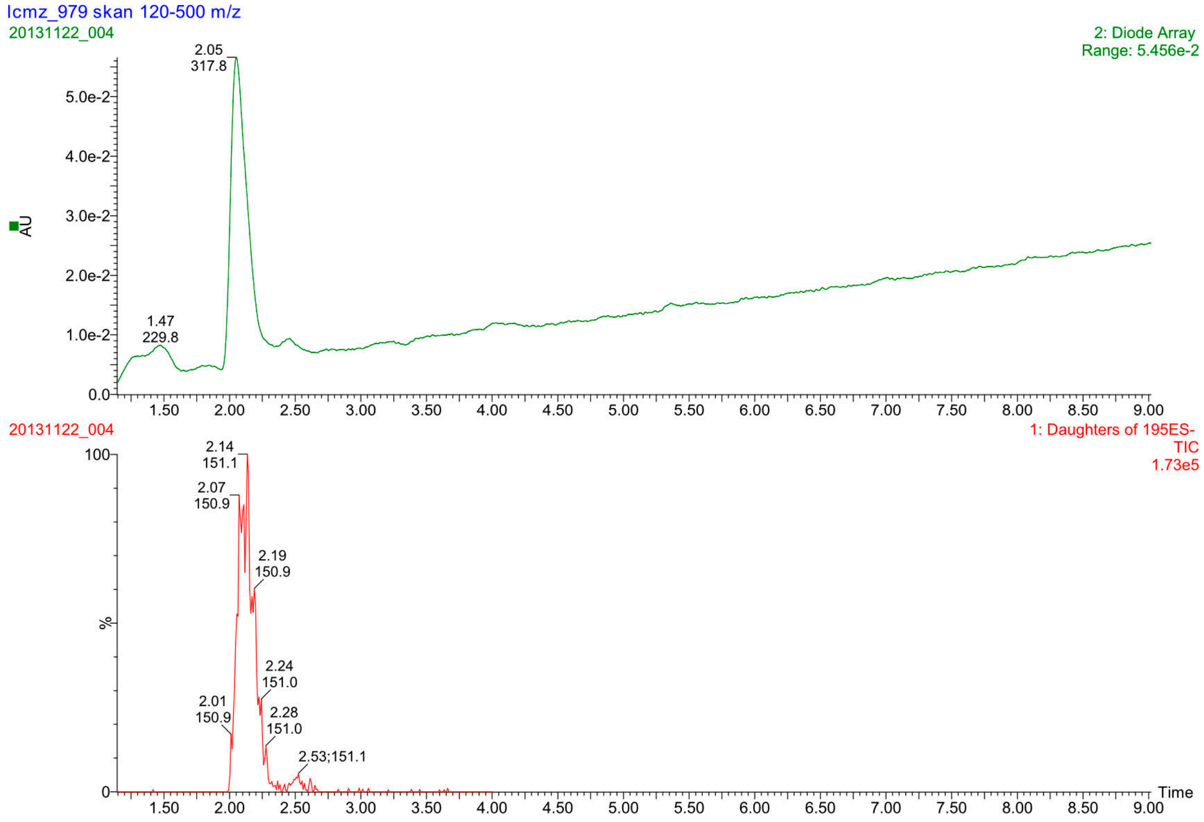

Figure S196. The UV spectrum of the putative 3,4,5-trihydroxycinnamic acid.

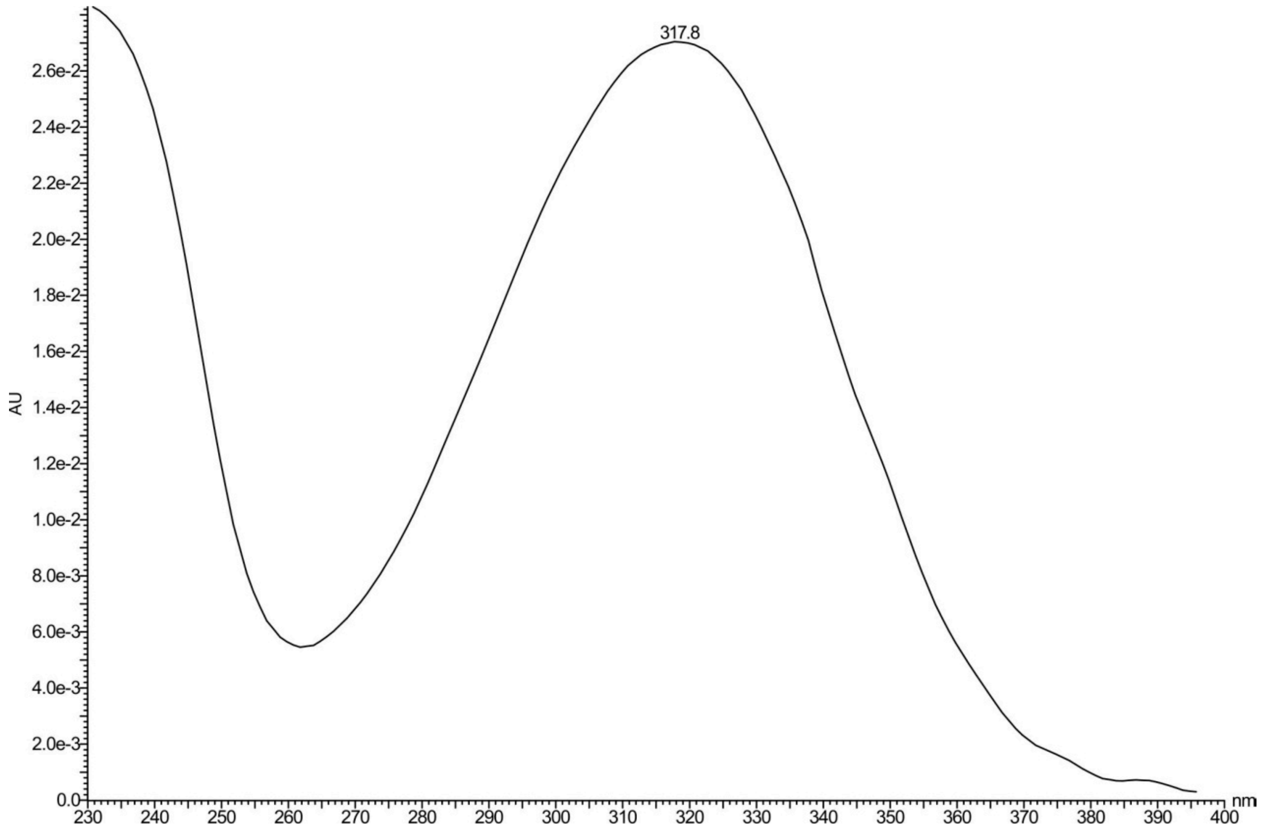

Figure S197. UPLC-MS chromatograms of phenolic acids released during alkaline hydrolysis of the compound **5**.

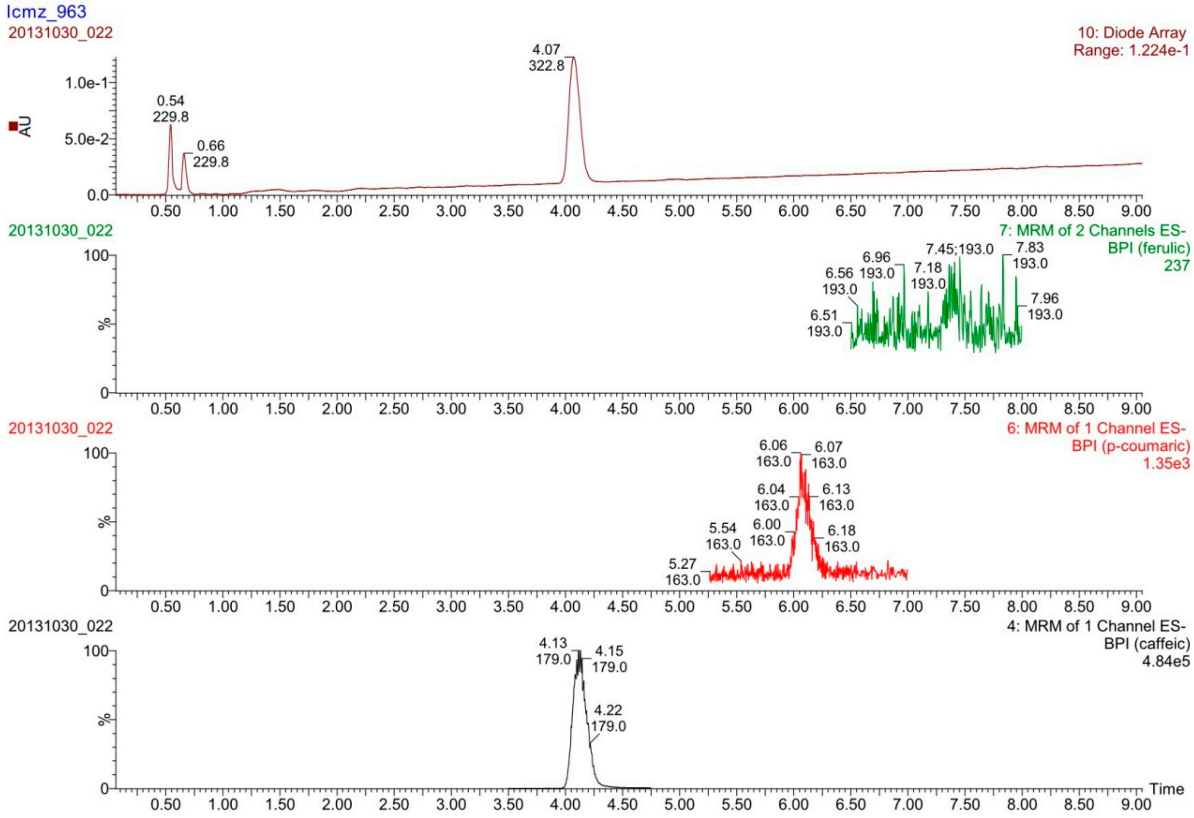

Figure S198. UPLC-MS chromatograms of phenolic acids released during alkaline hydrolysis of the compound **6**.

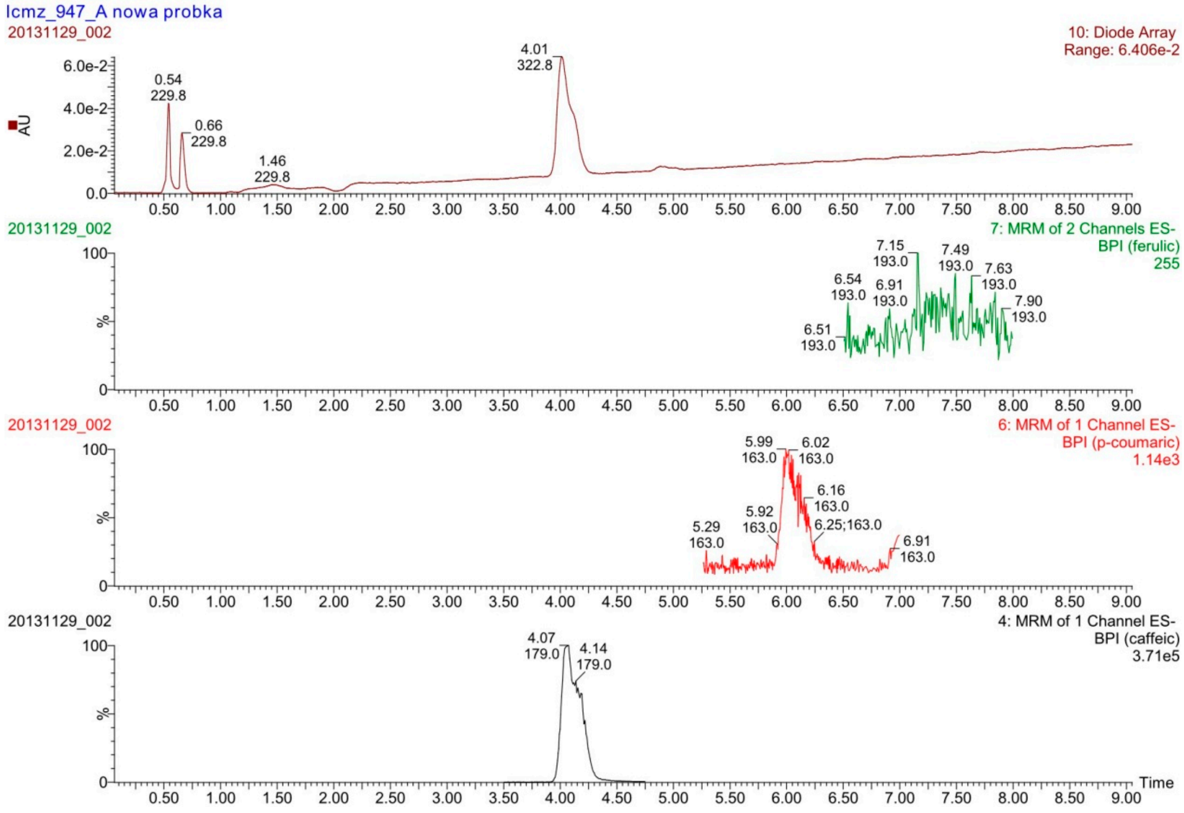

Figure S199. UPLC-MS chromatograms of phenolic acids released during alkaline hydrolysis of the compound **7**.

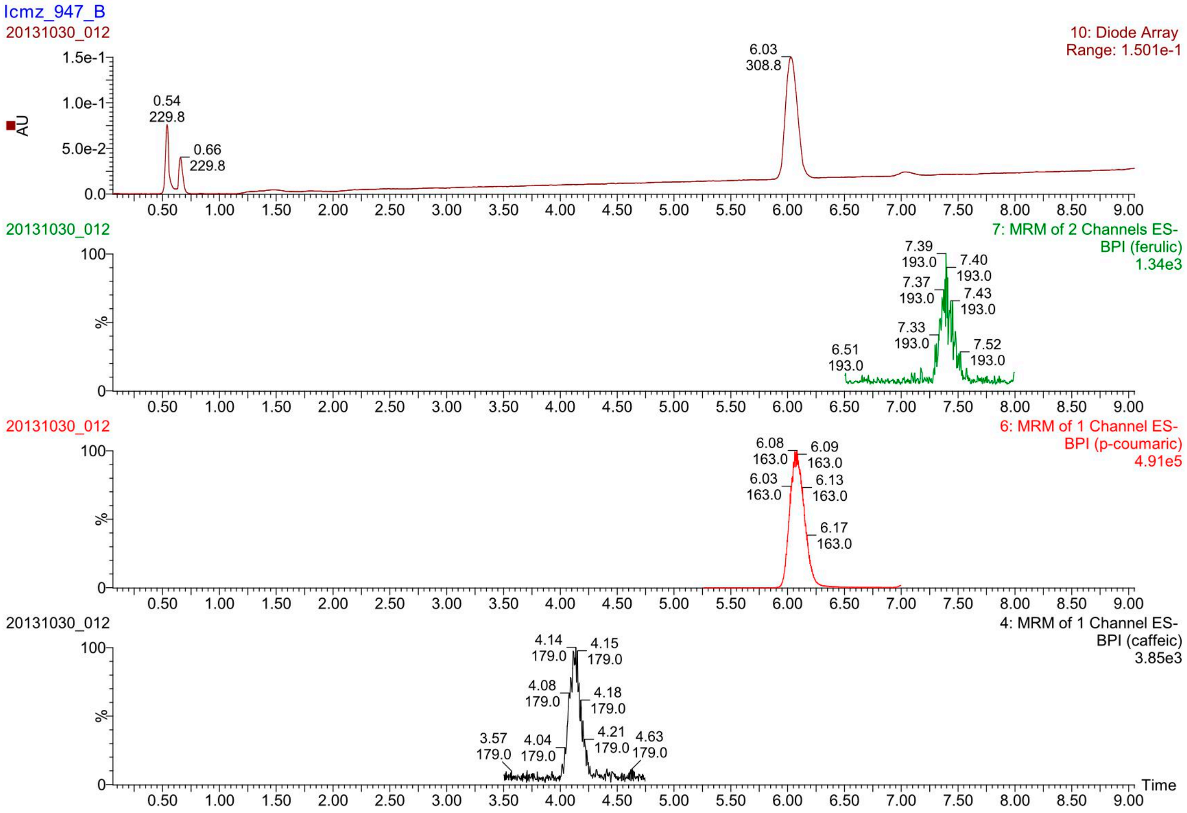

Figure S200. UPLC-MS chromatograms of phenolic acids released during alkaline hydrolysis of the compound **8**.

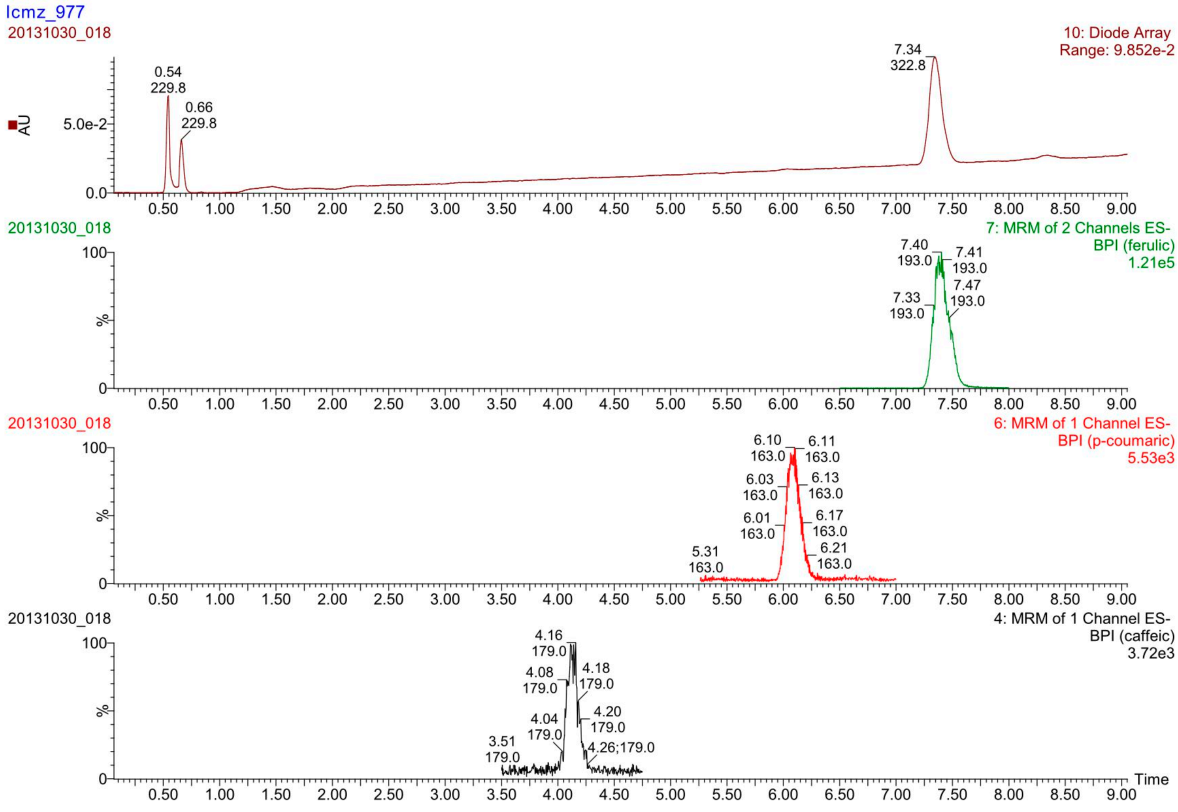

Figure S201. UPLC-MS chromatograms of phenolic acids released during alkaline hydrolysis of the compound **9**.

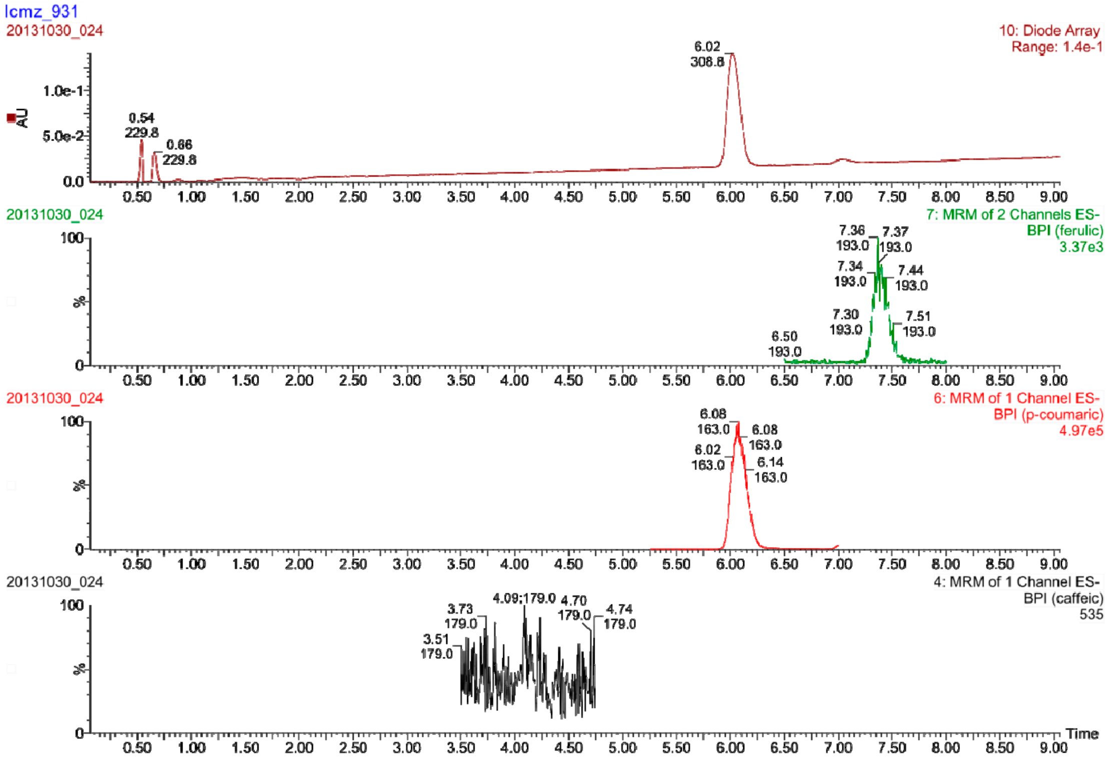

Figure S202. UPLC-MS chromatograms of phenolic acids released during alkaline hydrolysis of the compound **10**.

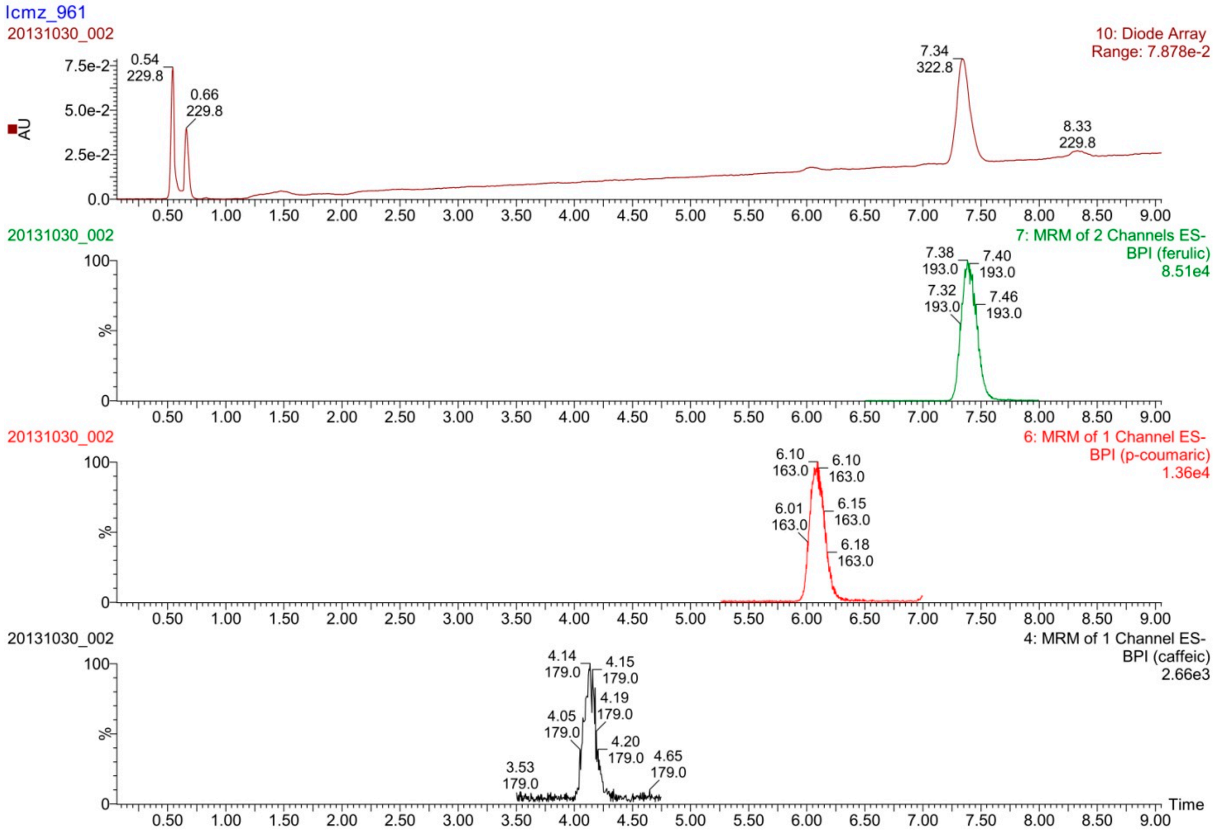

Figure S203. UPLC-MS chromatograms of phenolic acids released during alkaline hydrolysis of the compound **11**.

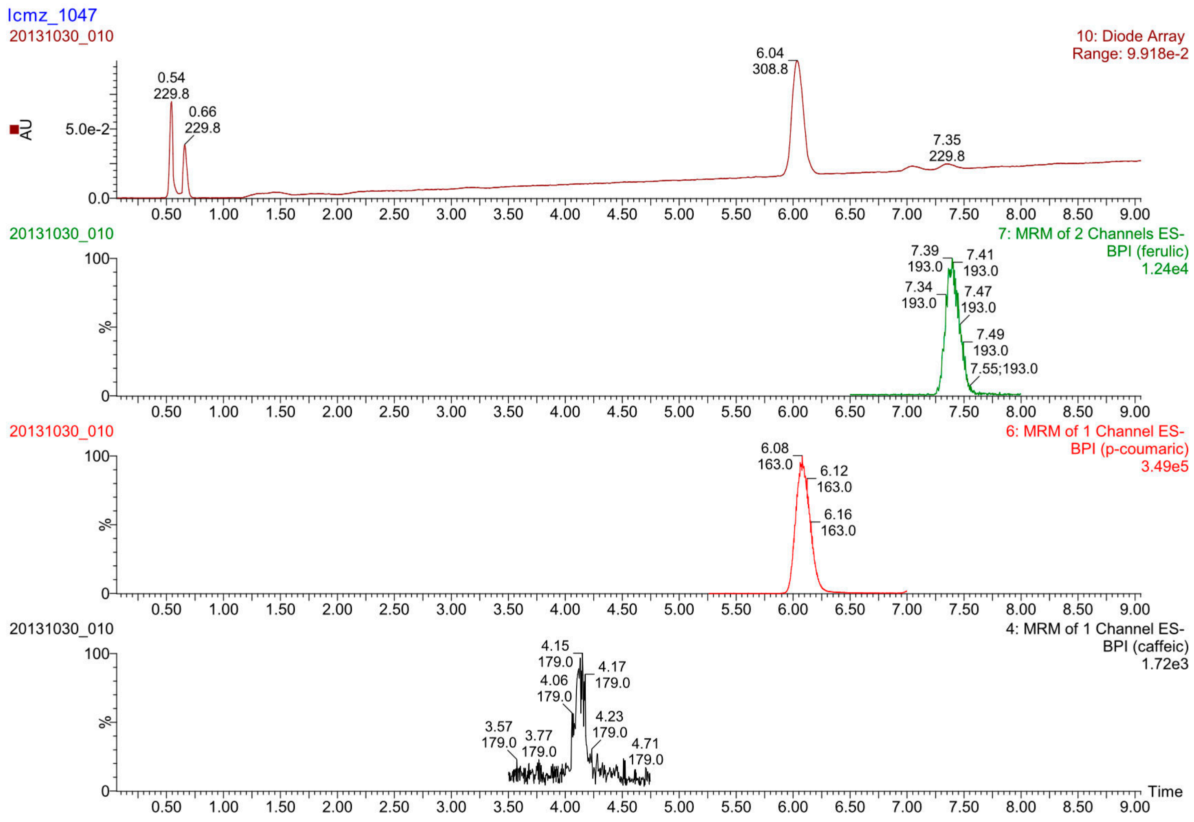

Figure S204. UPLC-MS chromatograms of phenolic acids released during alkaline hydrolysis of the compound **12**.

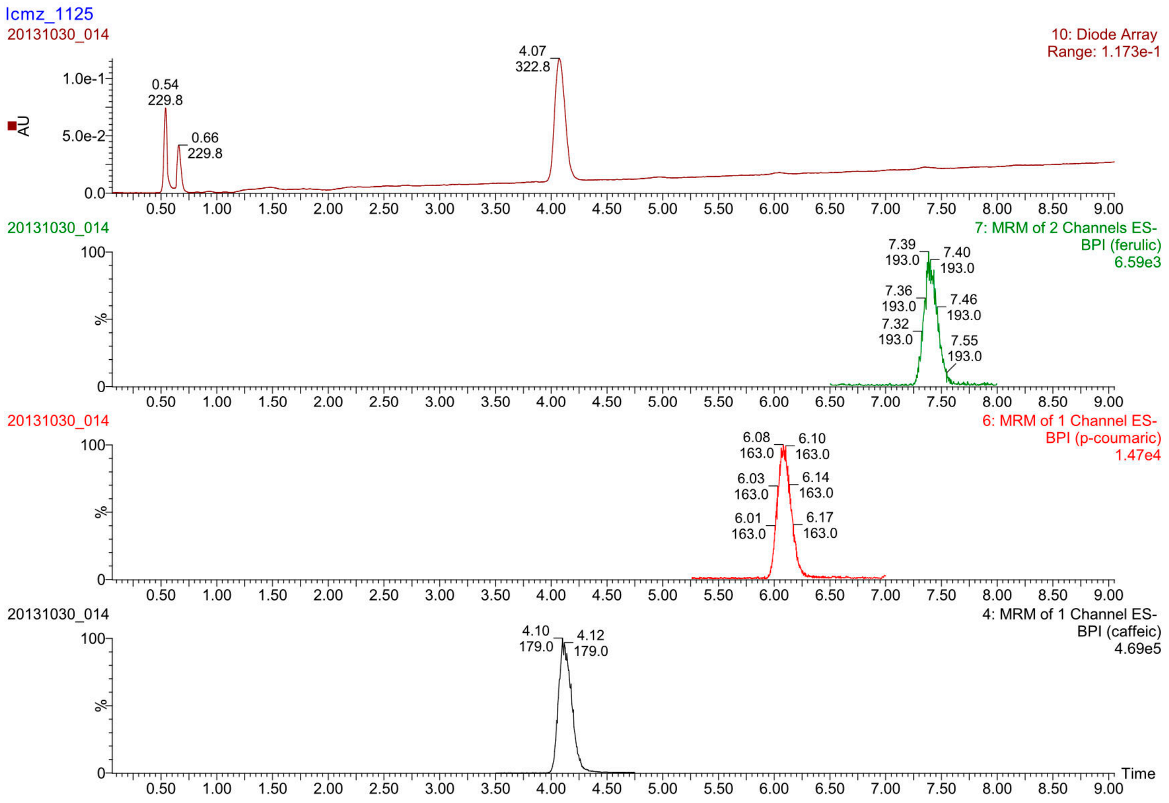

Figure S205. UPLC-MS chromatograms of phenolic acids released during alkaline hydrolysis of the compound **13**.

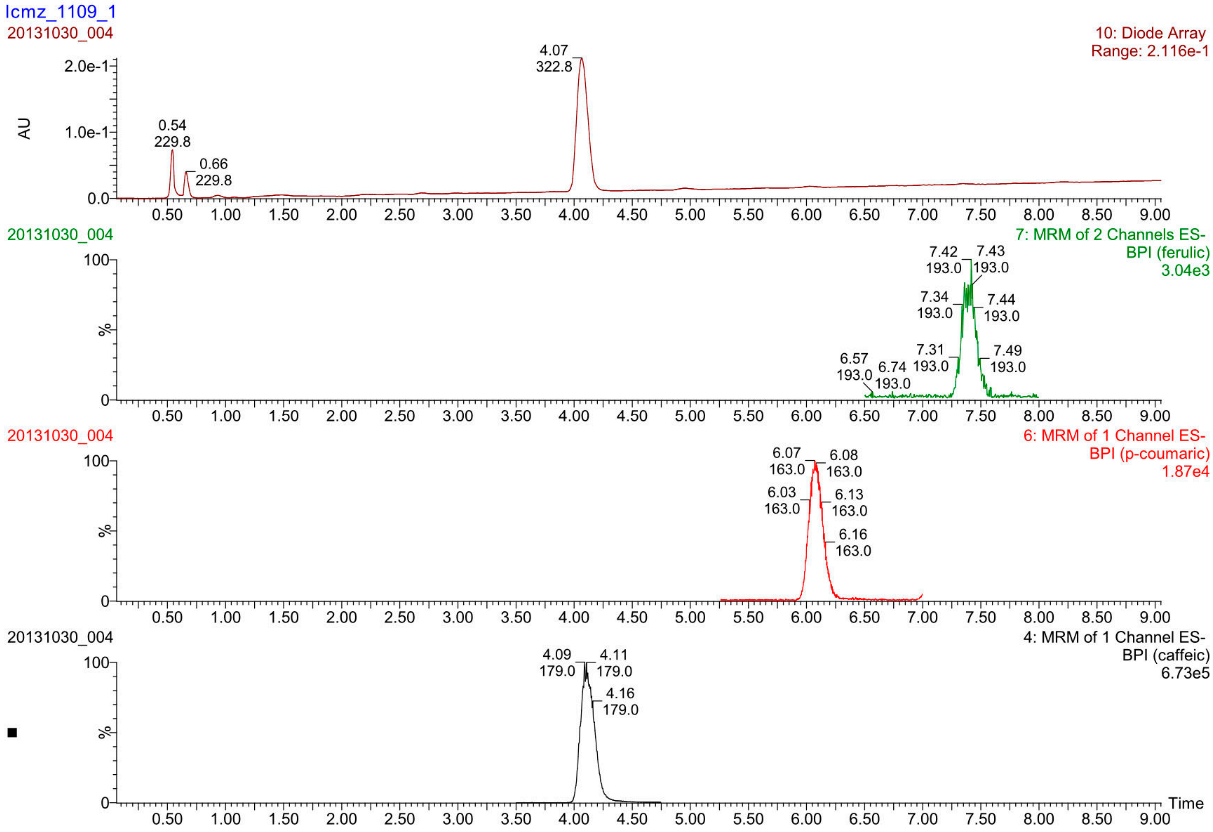

Figure S206. UPLC-MS chromatograms of phenolic acids released during alkaline hydrolysis of the compound **14**.

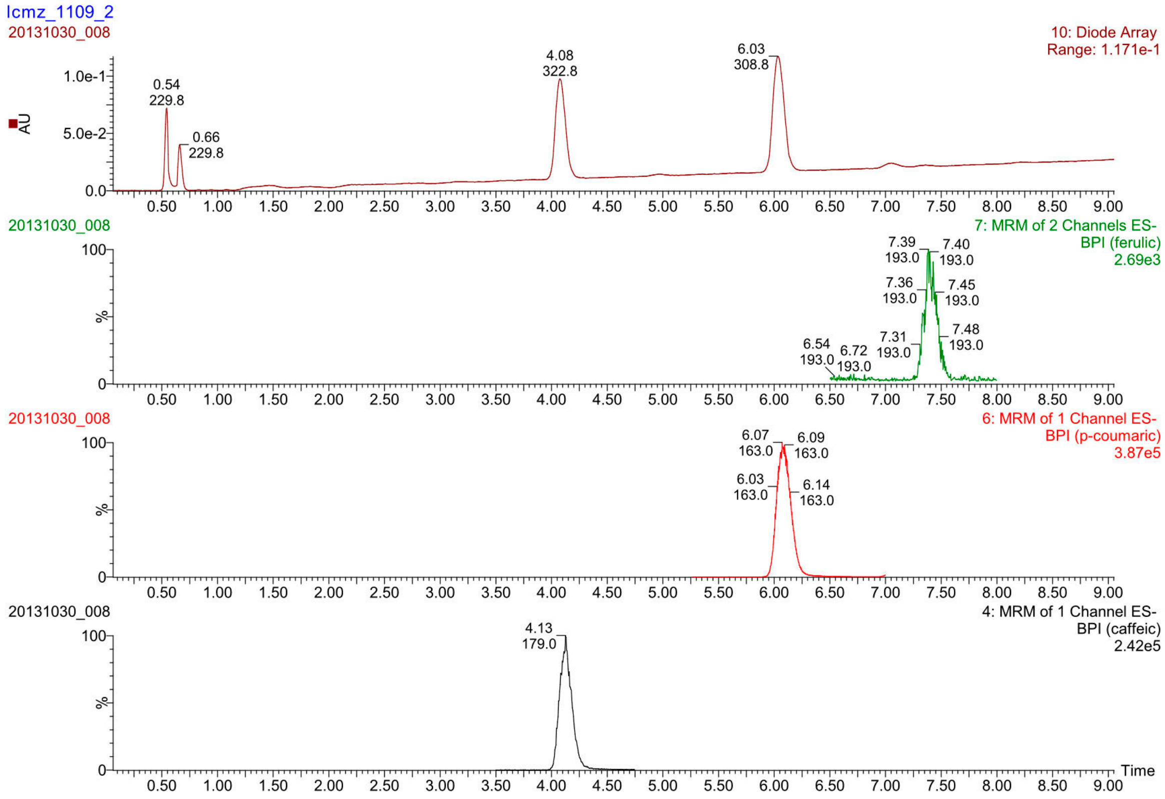

Figure S207. UPLC-MS chromatograms of phenolic acids released during alkaline hydrolysis of the compound **15**.

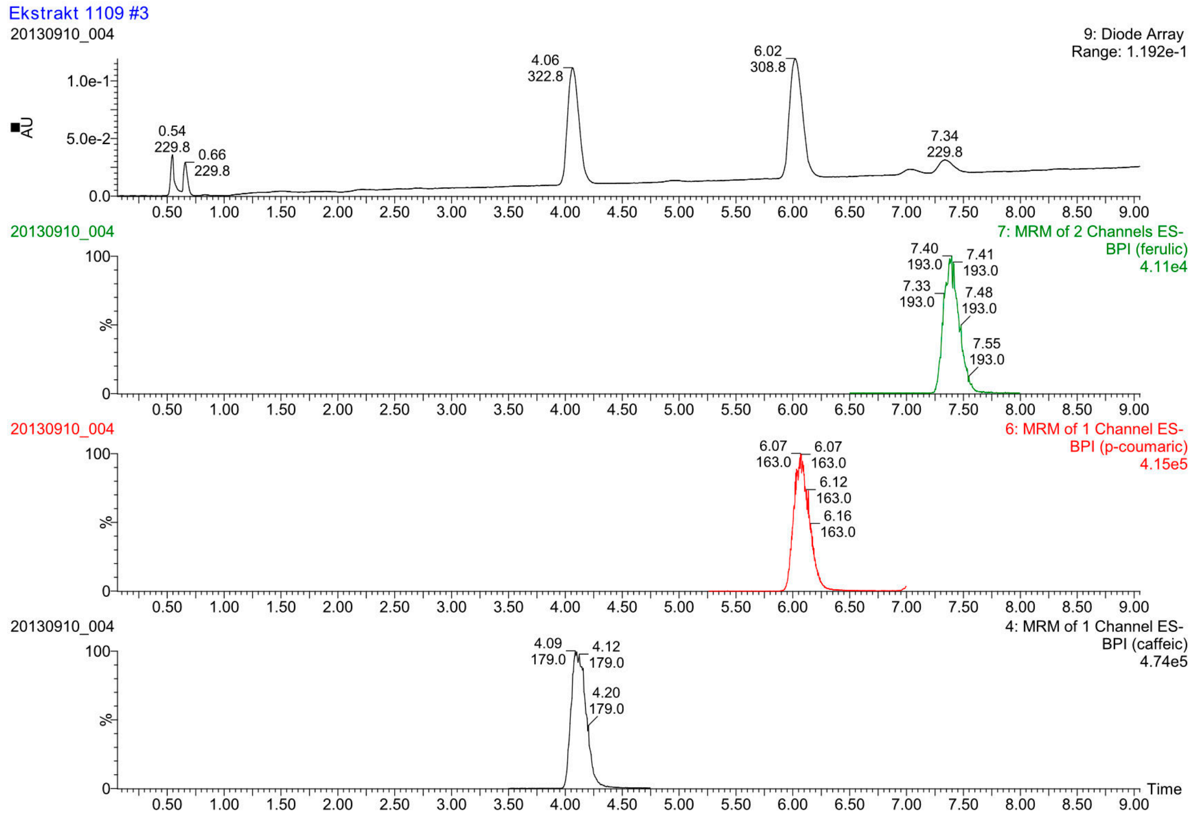

Figure S208. UPLC-MS chromatograms of phenolic acids released during alkaline hydrolysis of the compound **17**.

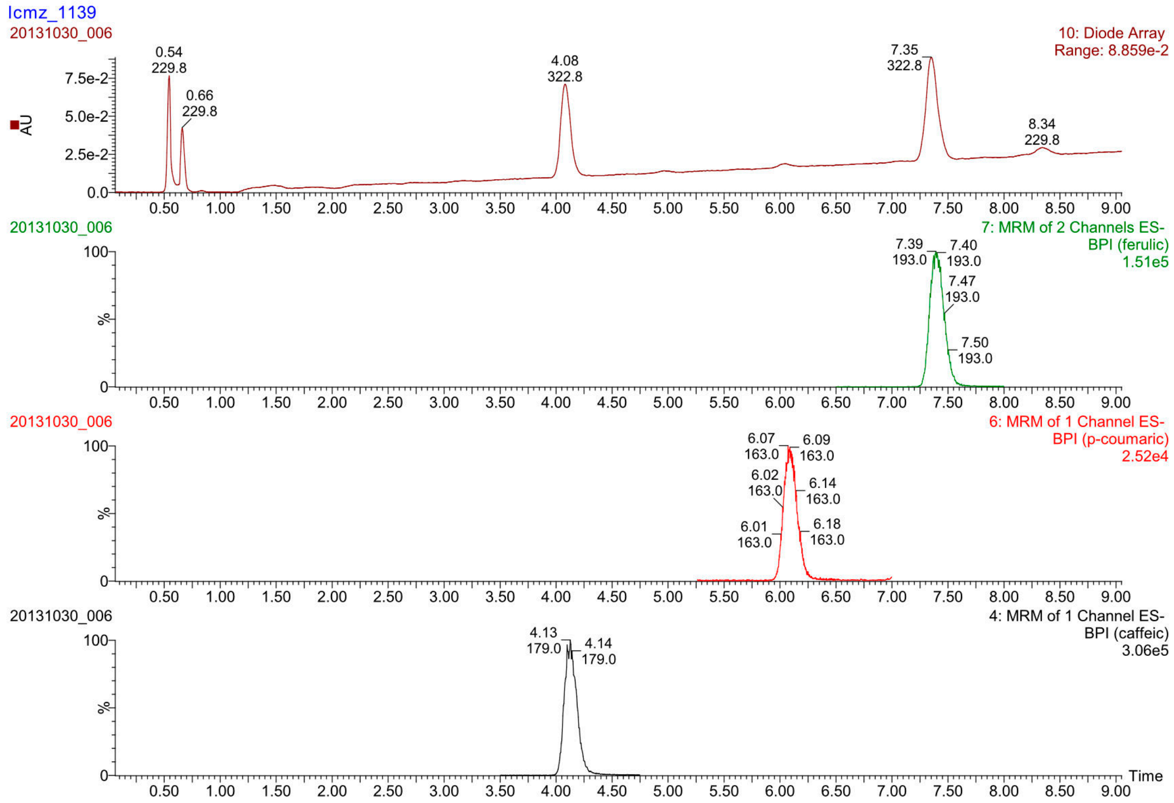

Figure S209. UPLC-MS chromatograms of phenolic acids released during alkaline hydrolysis of the compound **18**.

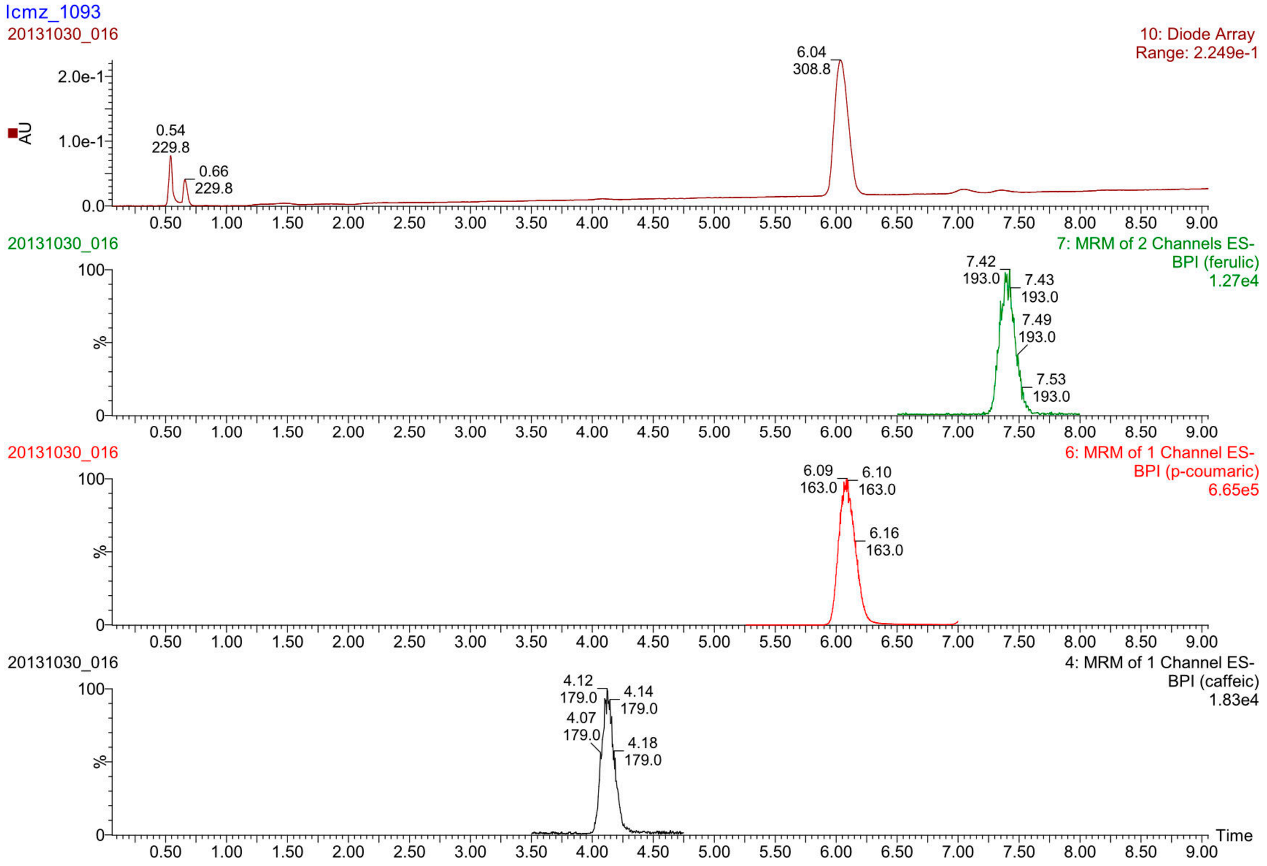

Figure S210. UPLC-MS chromatograms of the sugar standards.

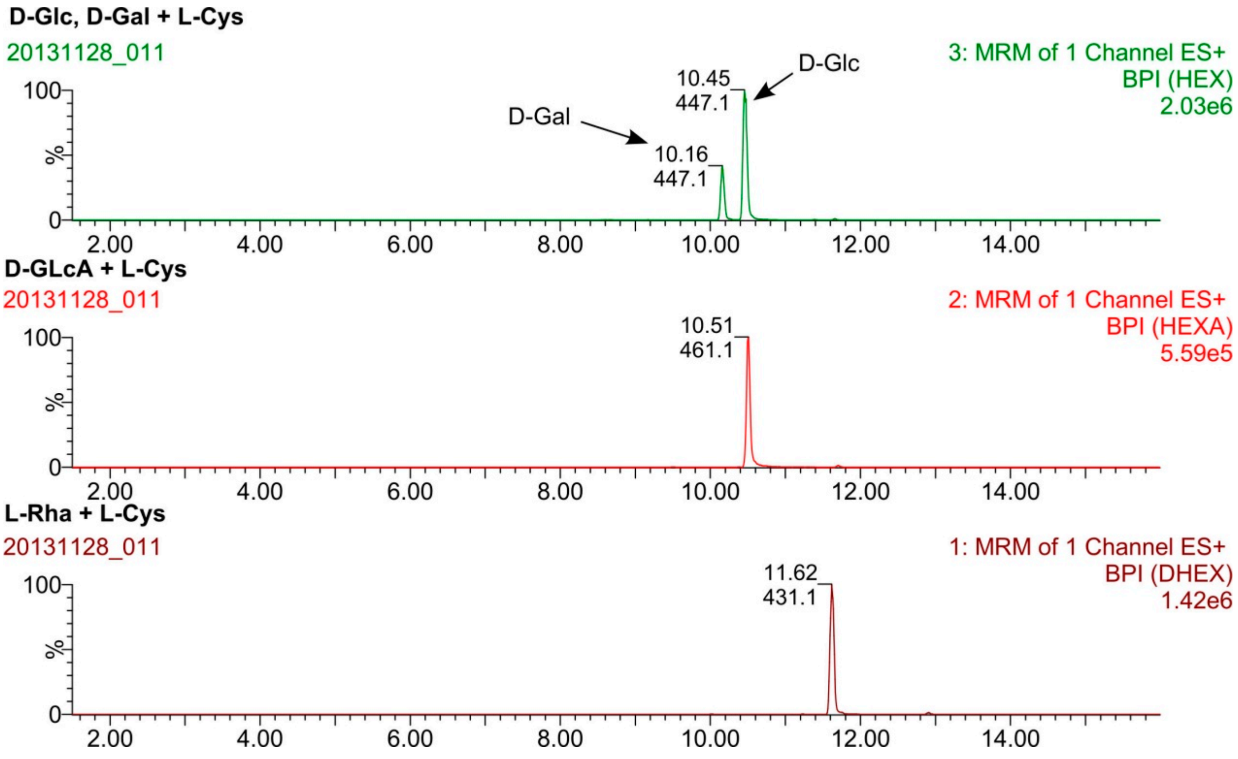

Figure S211. UPLC-MS chromatograms of sugars released during the acid hydrolysis of the compound **1**.

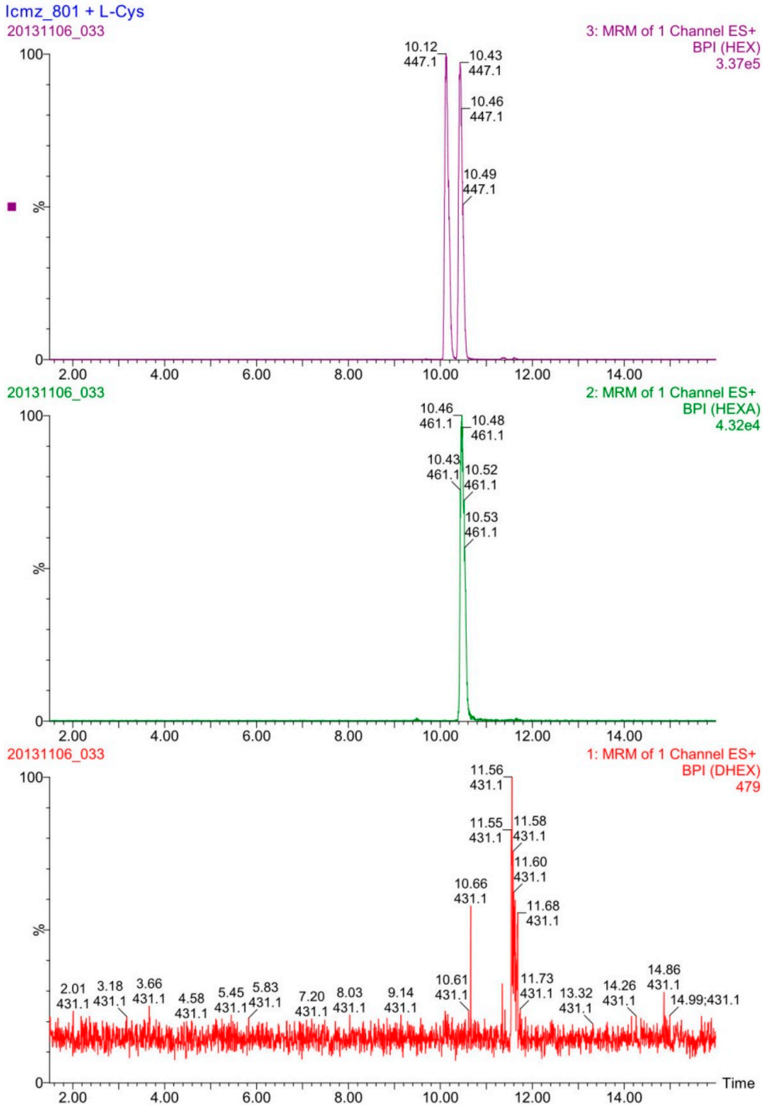

It can be noticed that heights of peaks of D-glucuronic acid, obtained for the samples of sugars released from the flavonoids **1**, **3**, **4–10**, **12–15**, **17**, and **18,** were distinctly lower than those of D-glucose and D-galactose. This may be explained by the fact that 7-*O*-glucuronides of flavonols are much less susceptible to acid hydrolysis than their 7-*O*-glucosides, and especially 7-*O*-rhamnosides (Mabry, T.J.; Markham, K.R.; Thomas, M.B. The aglycone and sugar analysis of flavonoid glycosides. In *The Systematic Identification of Flavonoids*; Springer: Berlin/Heidelberg, Germany, **1970**; pp. 23–32).

Figure S212. UPLC-MS chromatograms of sugars released during the acid hydrolysis of the compound **3**.

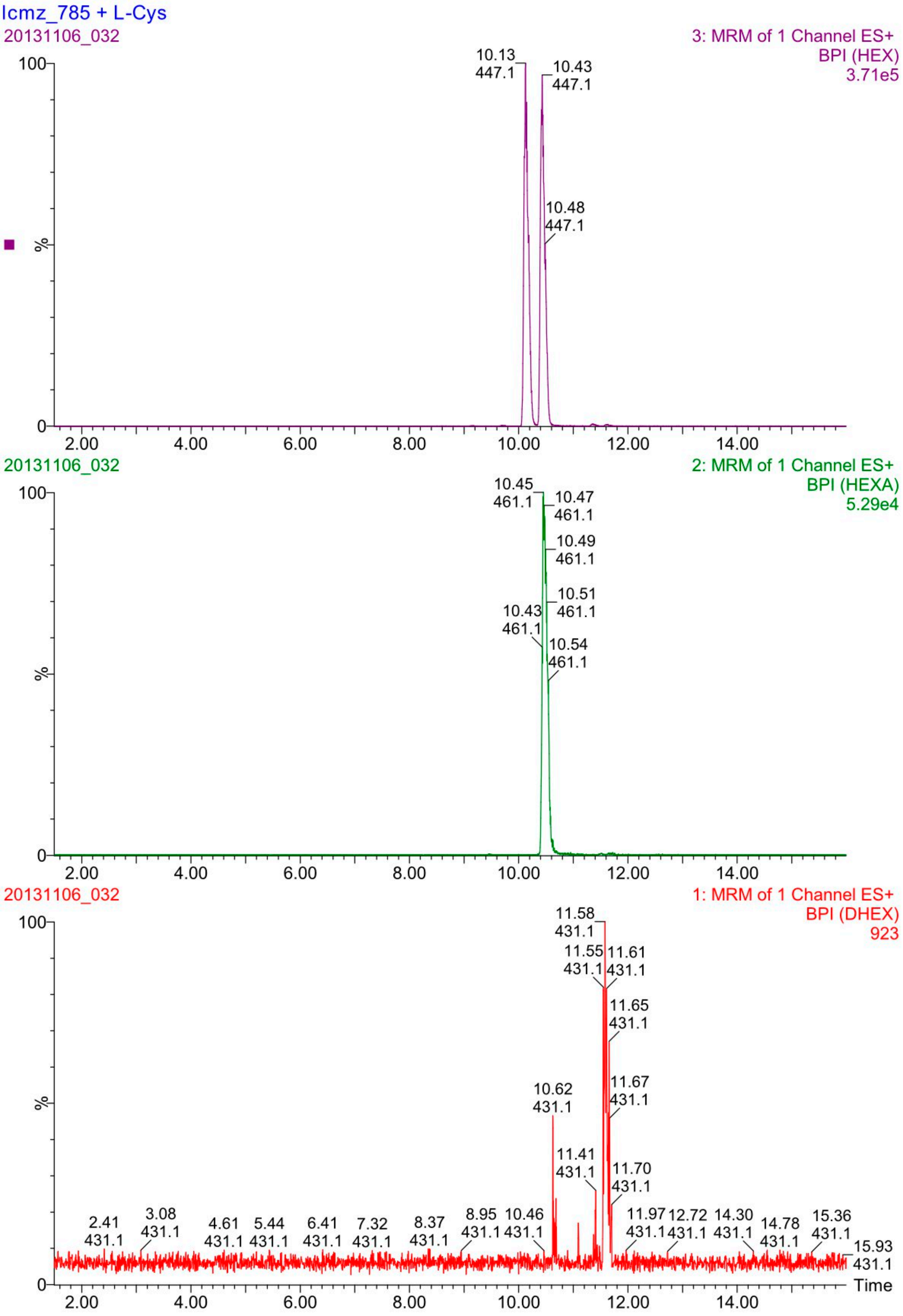

Figure S213. UPLC-MS chromatograms of sugars released during the acid hydrolysis of the compound **4**.

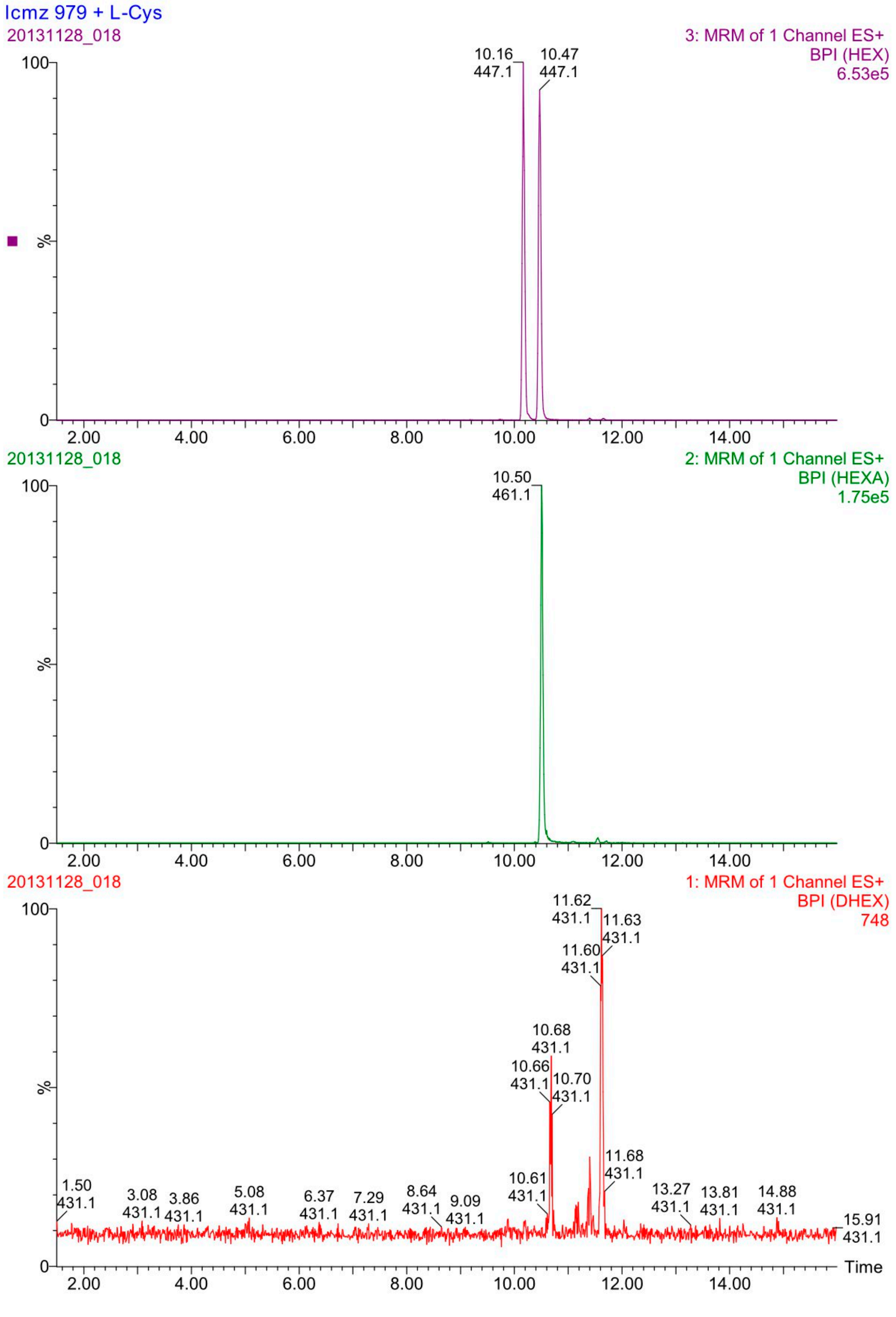

Figure S214. UPLC-MS chromatograms of sugars released during the acid hydrolysis of the compound **5**.

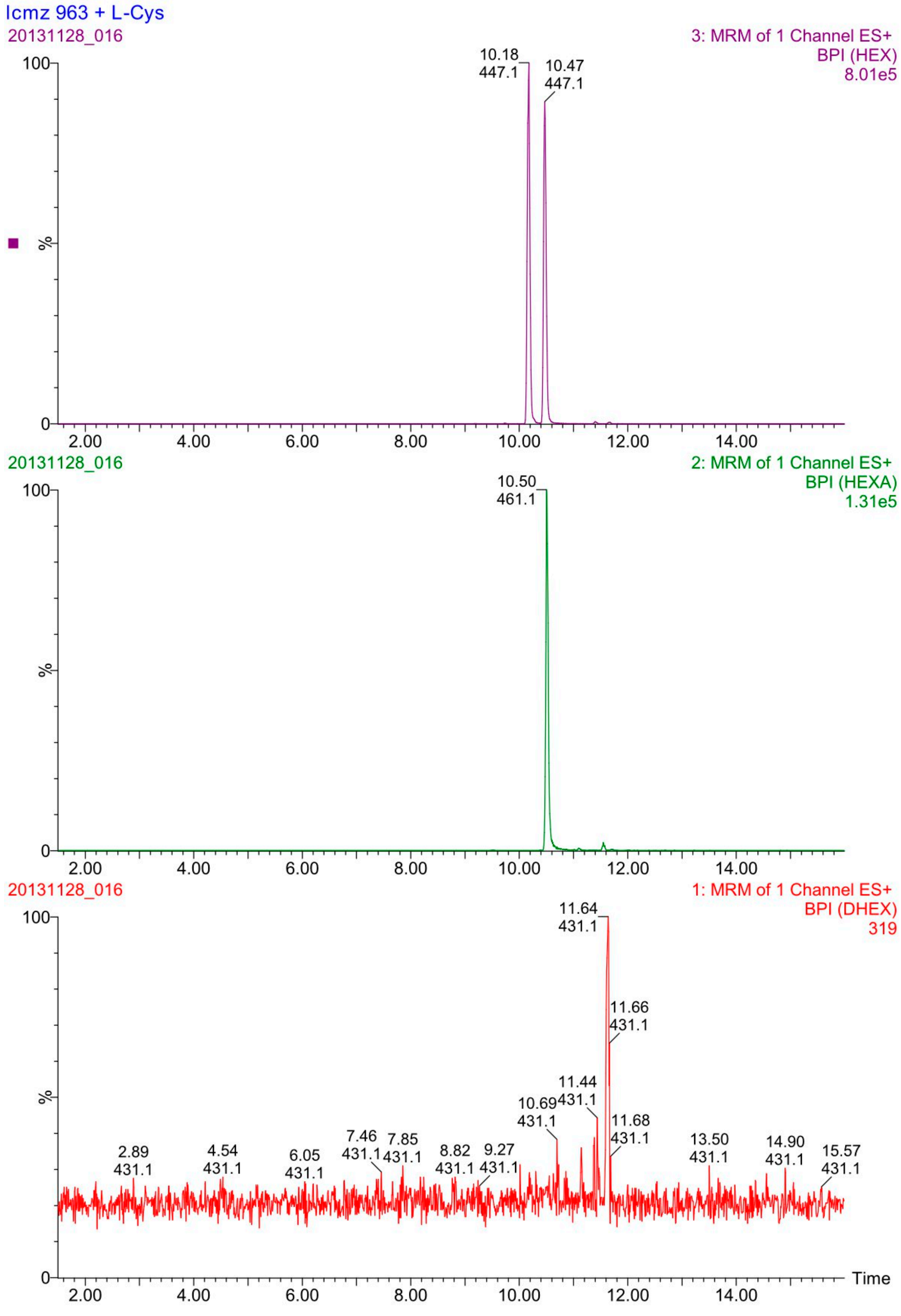

Figure S215. UPLC-MS chromatograms of sugars released during the acid hydrolysis of the compound **6**.

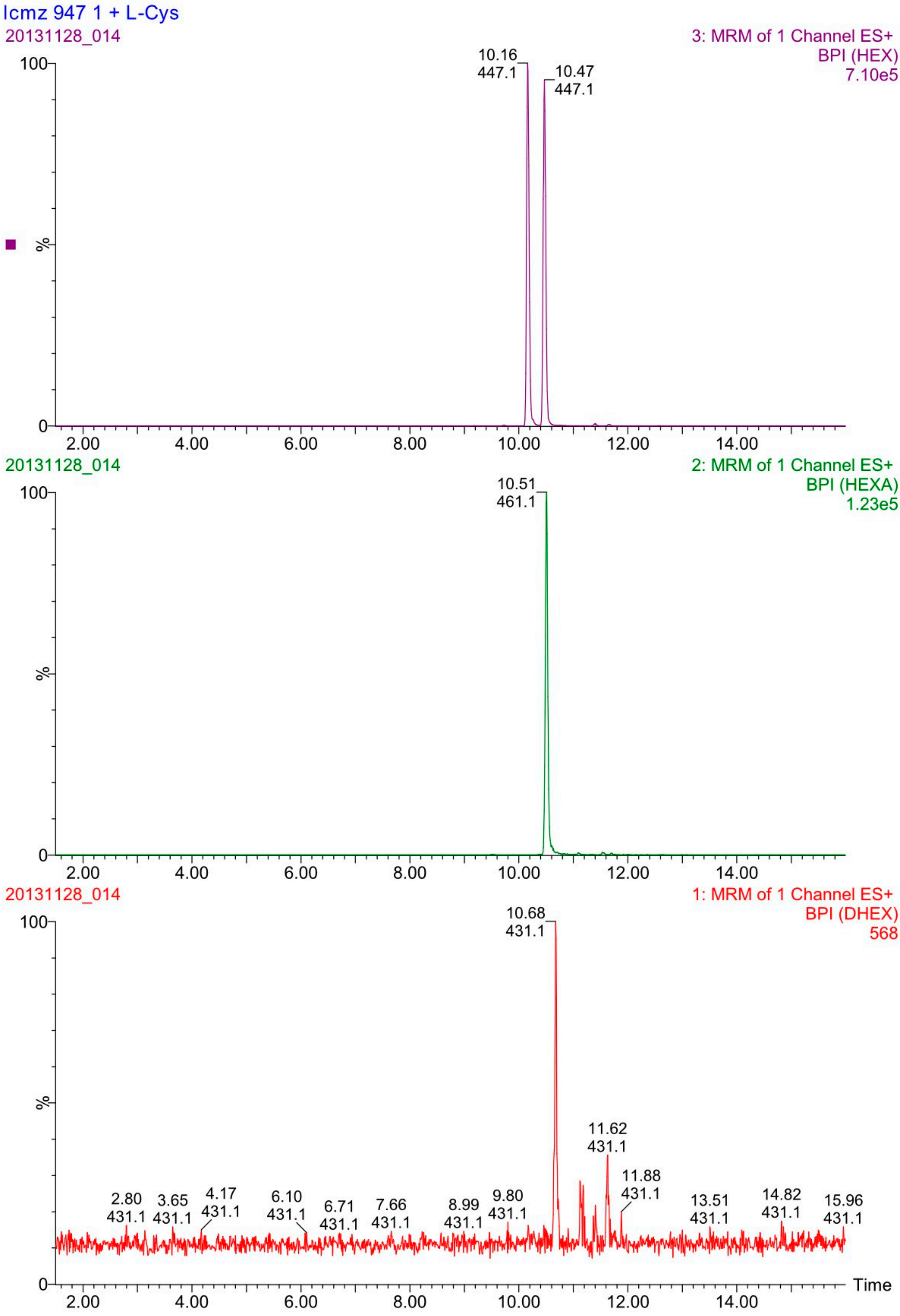

Figure S216. UPLC-MS chromatograms of sugars released during the acid hydrolysis of the compound **7**.

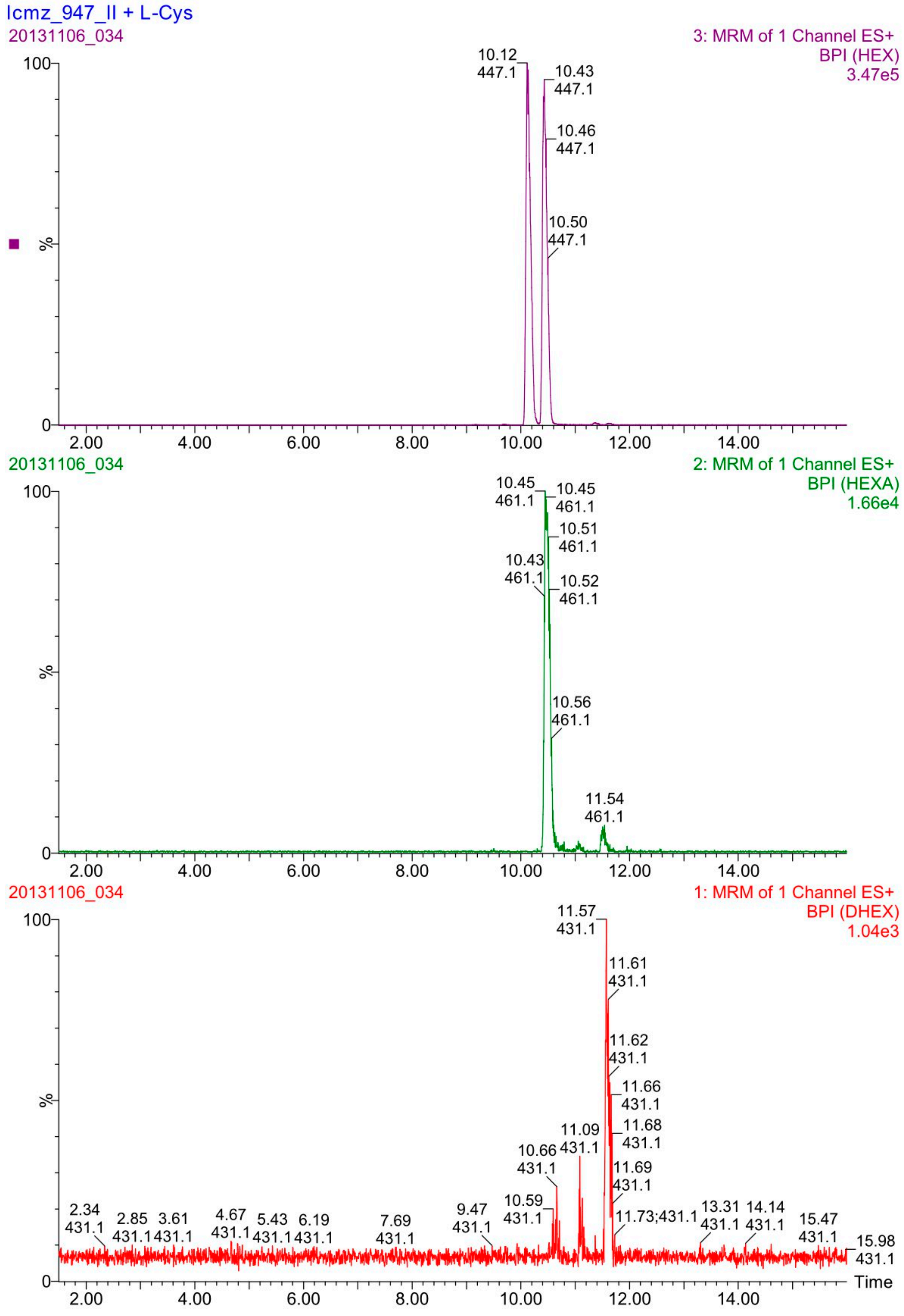

Figure S217. UPLC-MS chromatograms of sugars released during the acid hydrolysis of the compound **8**.

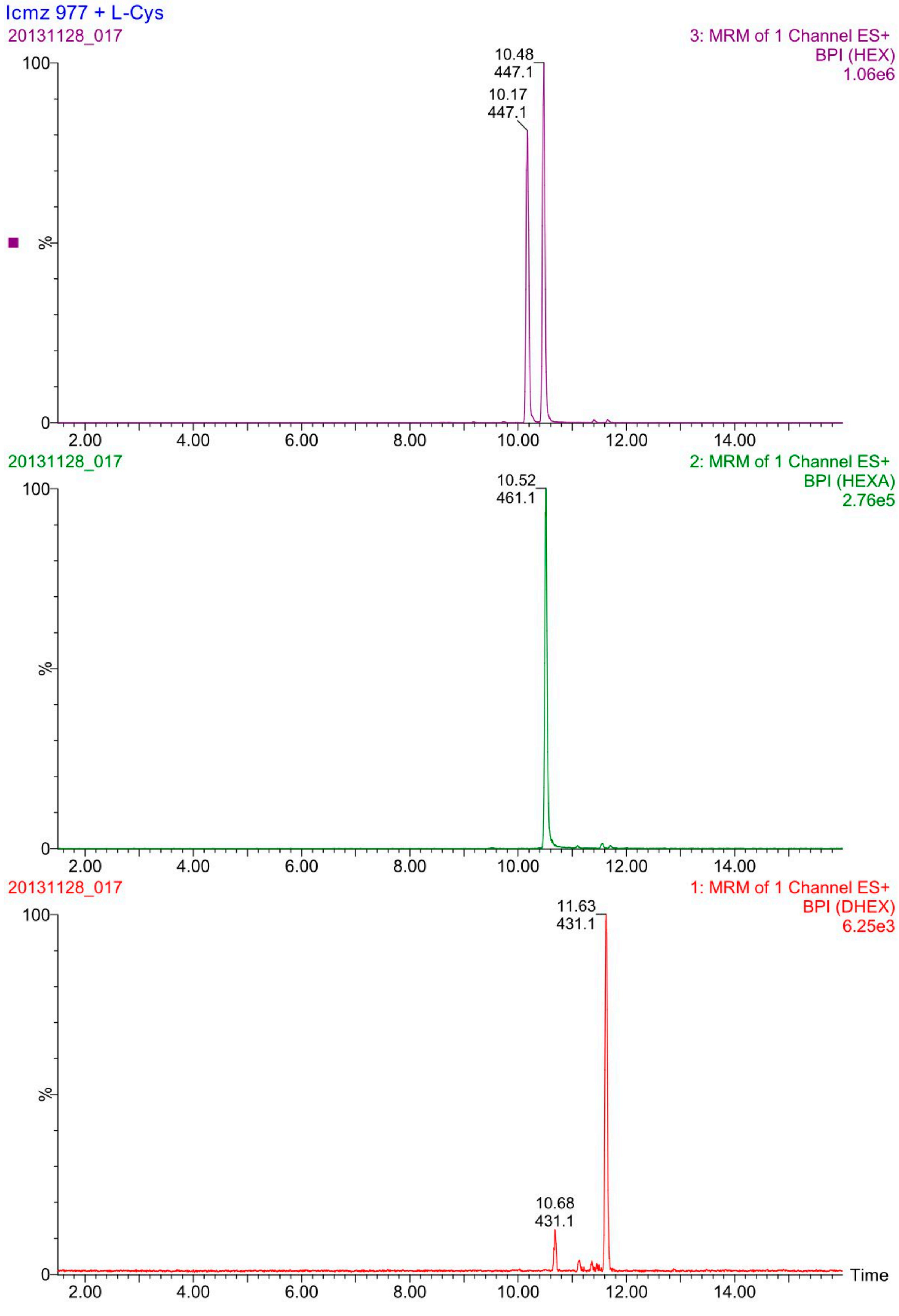

Figure S218. UPLC-MS chromatograms of sugars released during the acid hydrolysis of the compound **9**.

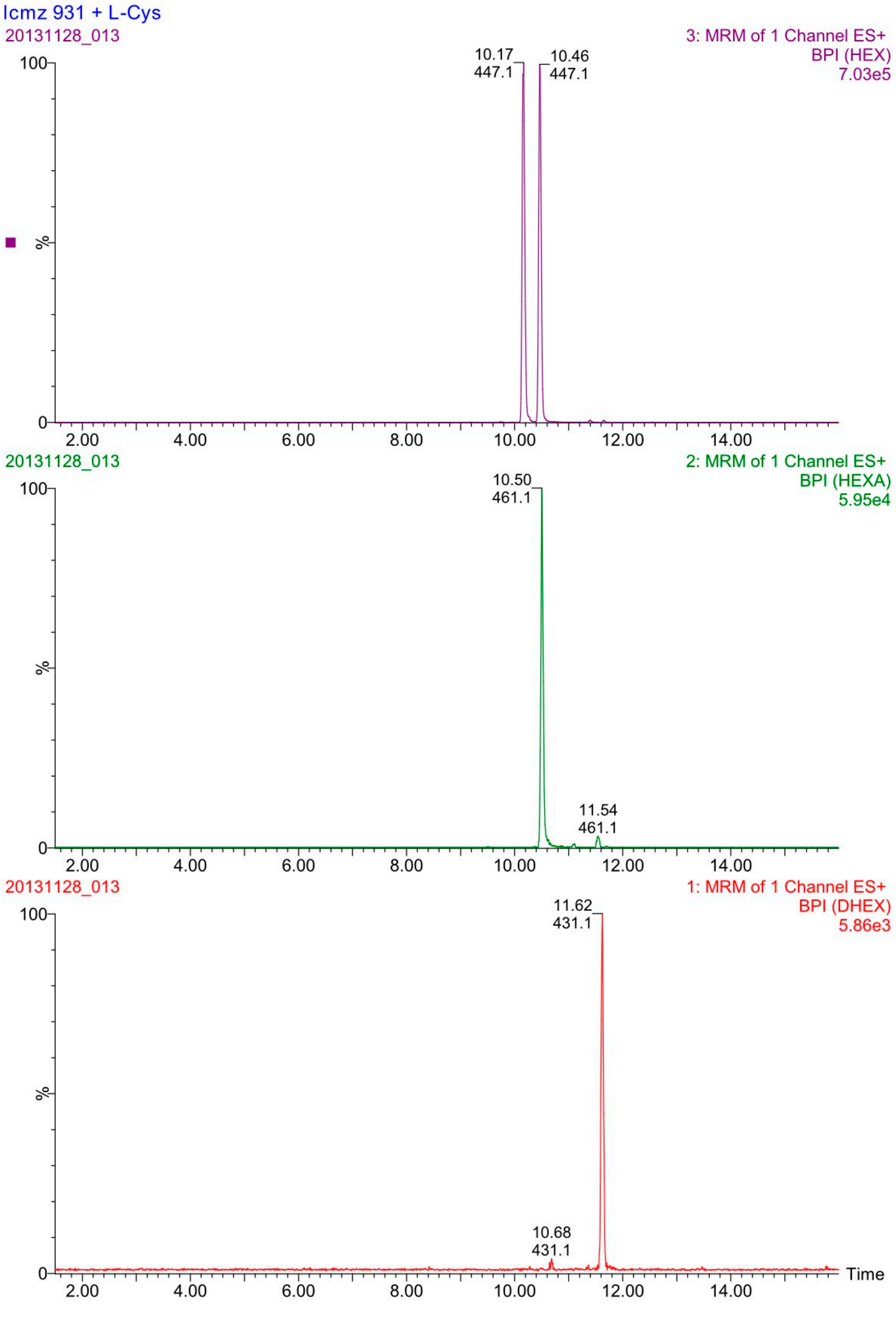

Figure S219. UPLC-MS chromatograms of sugars released during the acid hydrolysis of the compound **10.**

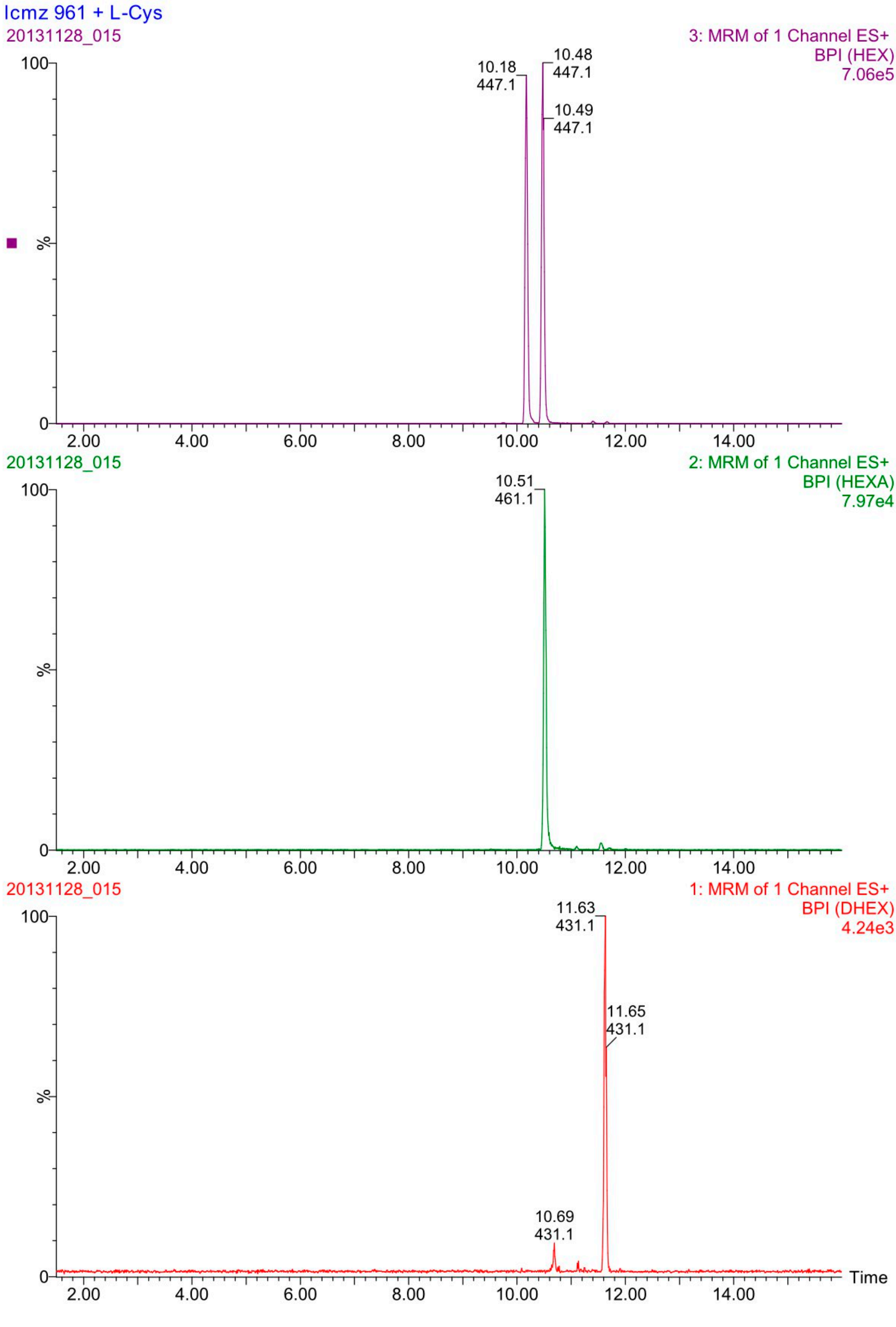

Figure S220. UPLC-MS chromatograms of sugars released during the acid hydrolysis of the compound **11**.

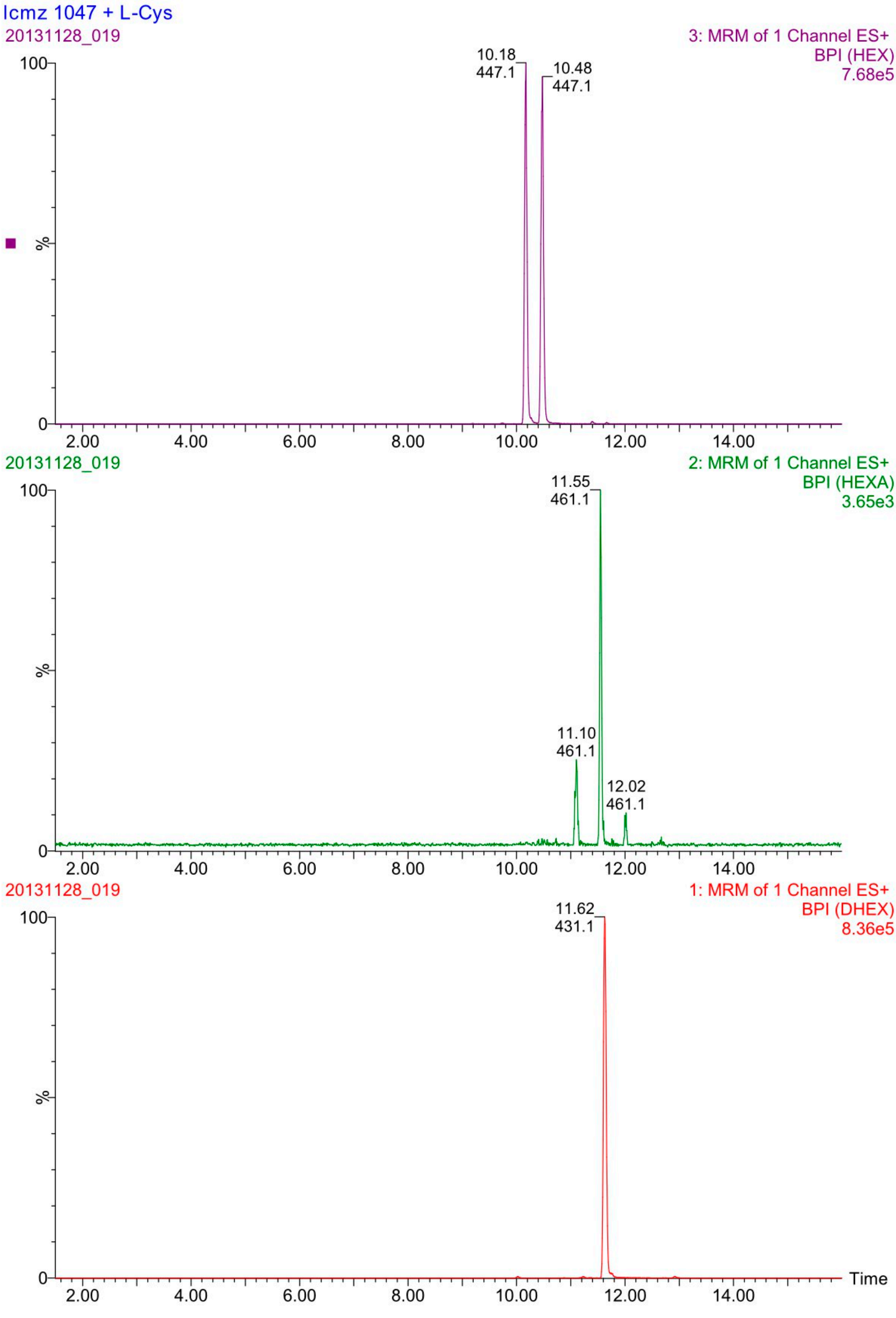

Figure S221. UPLC-MS chromatograms of sugars released during the acid hydrolysis of the compound **12**.

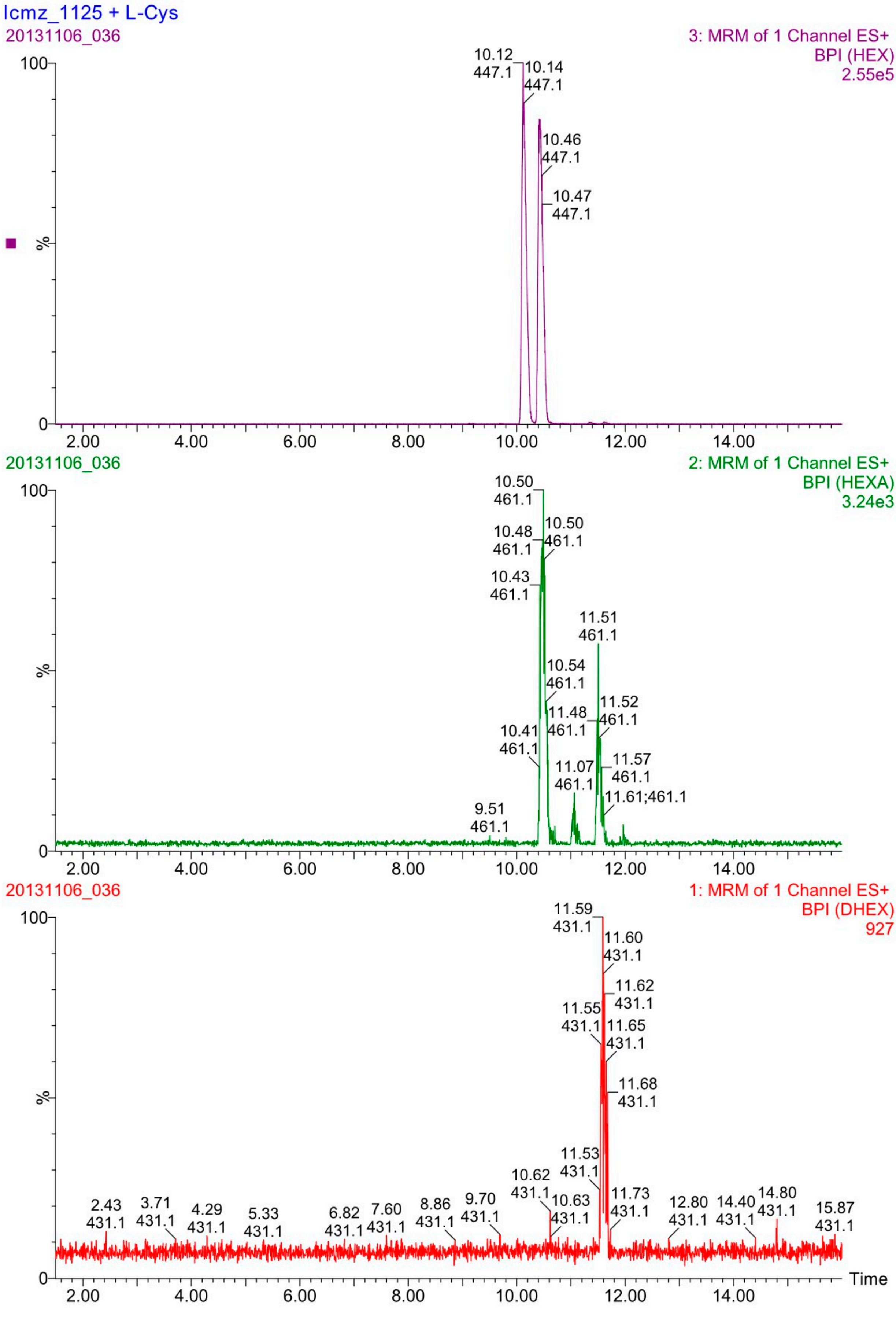

Figure S222. UPLC-MS chromatograms of sugars released during the acid hydrolysis of the compound **13**.

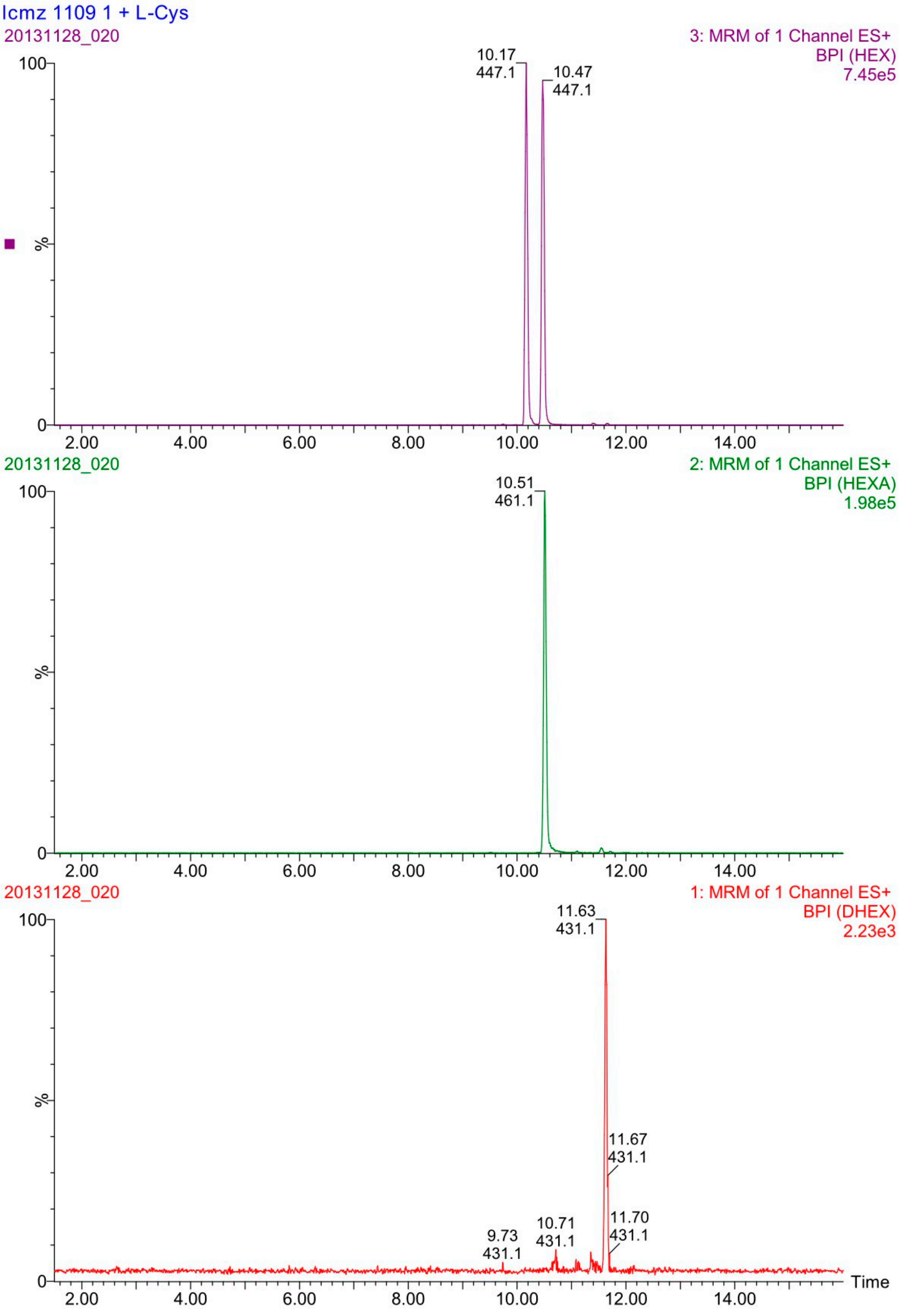

Figure S223. UPLC-MS chromatograms of sugars released during the acid hydrolysis of the compound **14**.

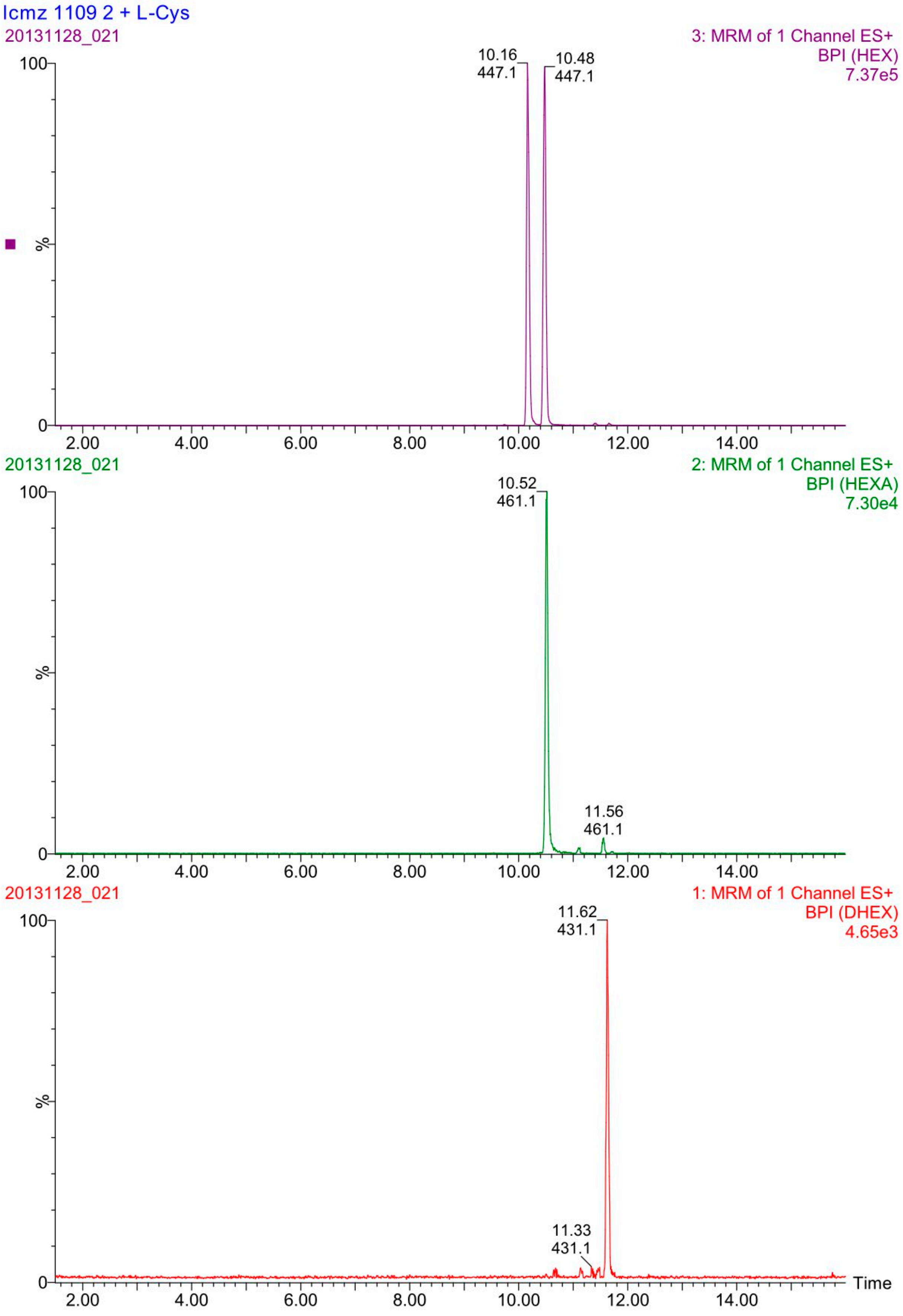

Figure S224. UPLC-MS chromatograms of sugars released during the acid hydrolysis of the compound **15**.

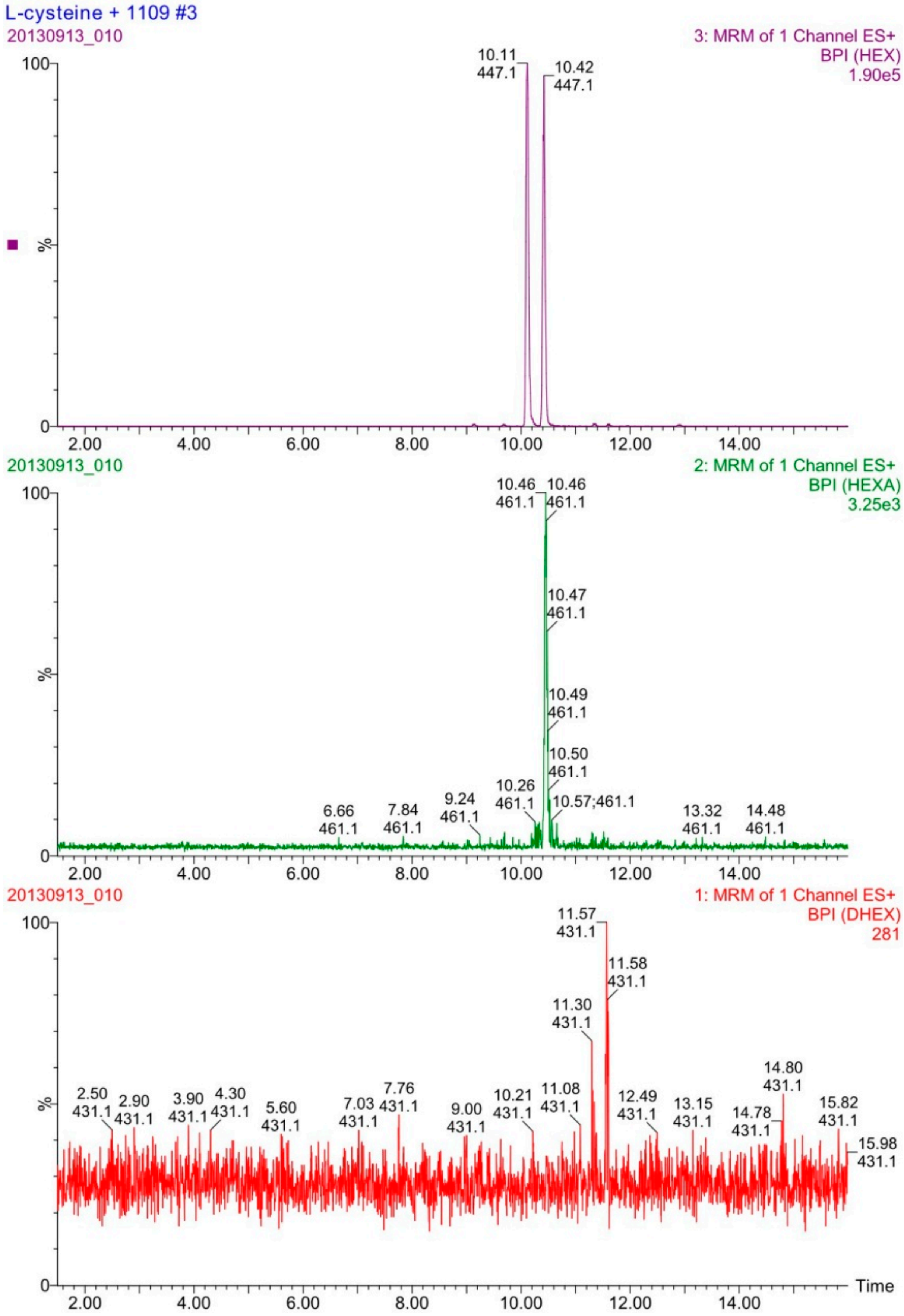

Figure S225. UPLC-MS chromatograms of sugars released during the acid hydrolysis of the compound **17**.

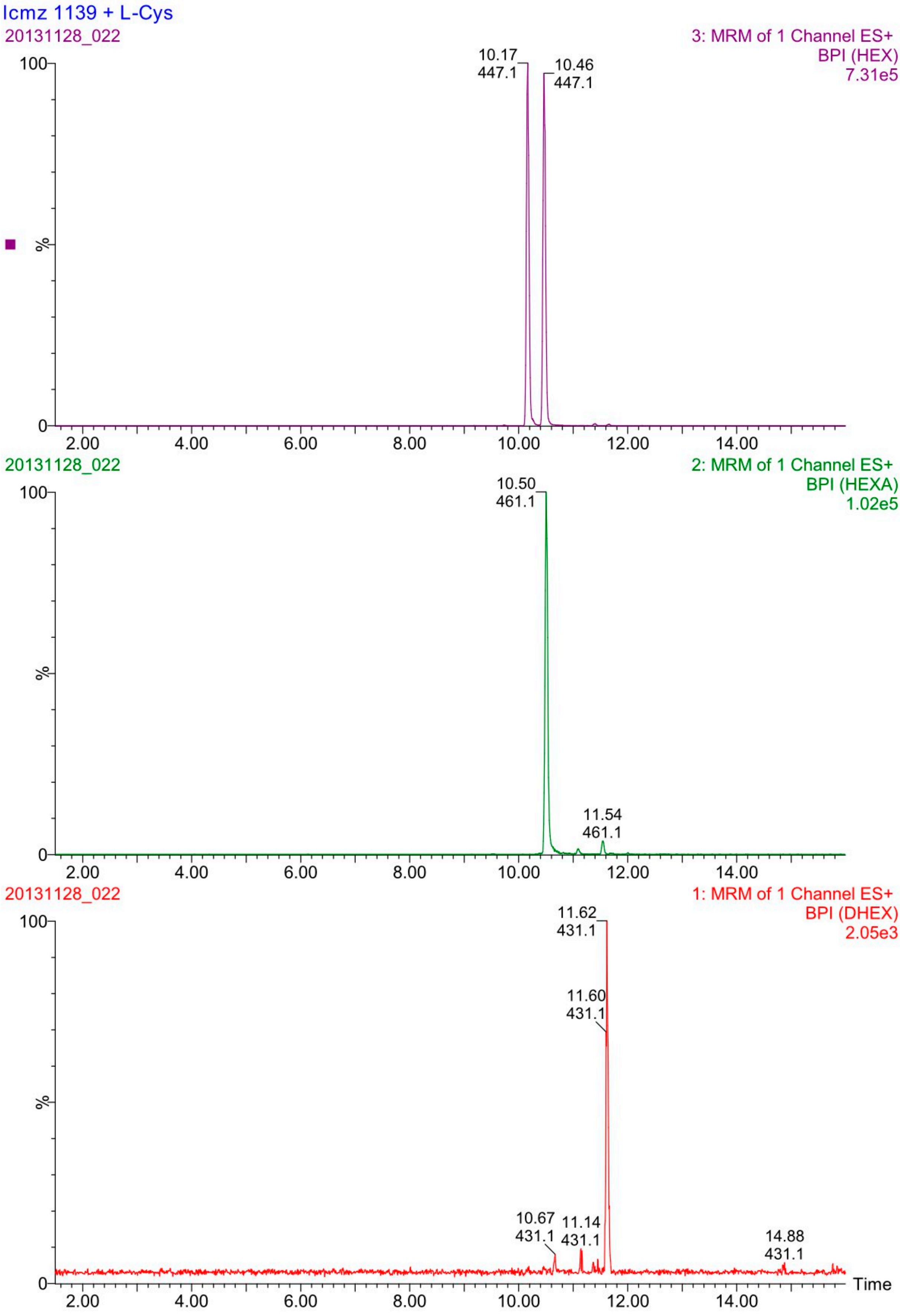

Figure S226. UPLC-MS chromatograms of sugars released during the acid hydrolysis of the compound **18.**

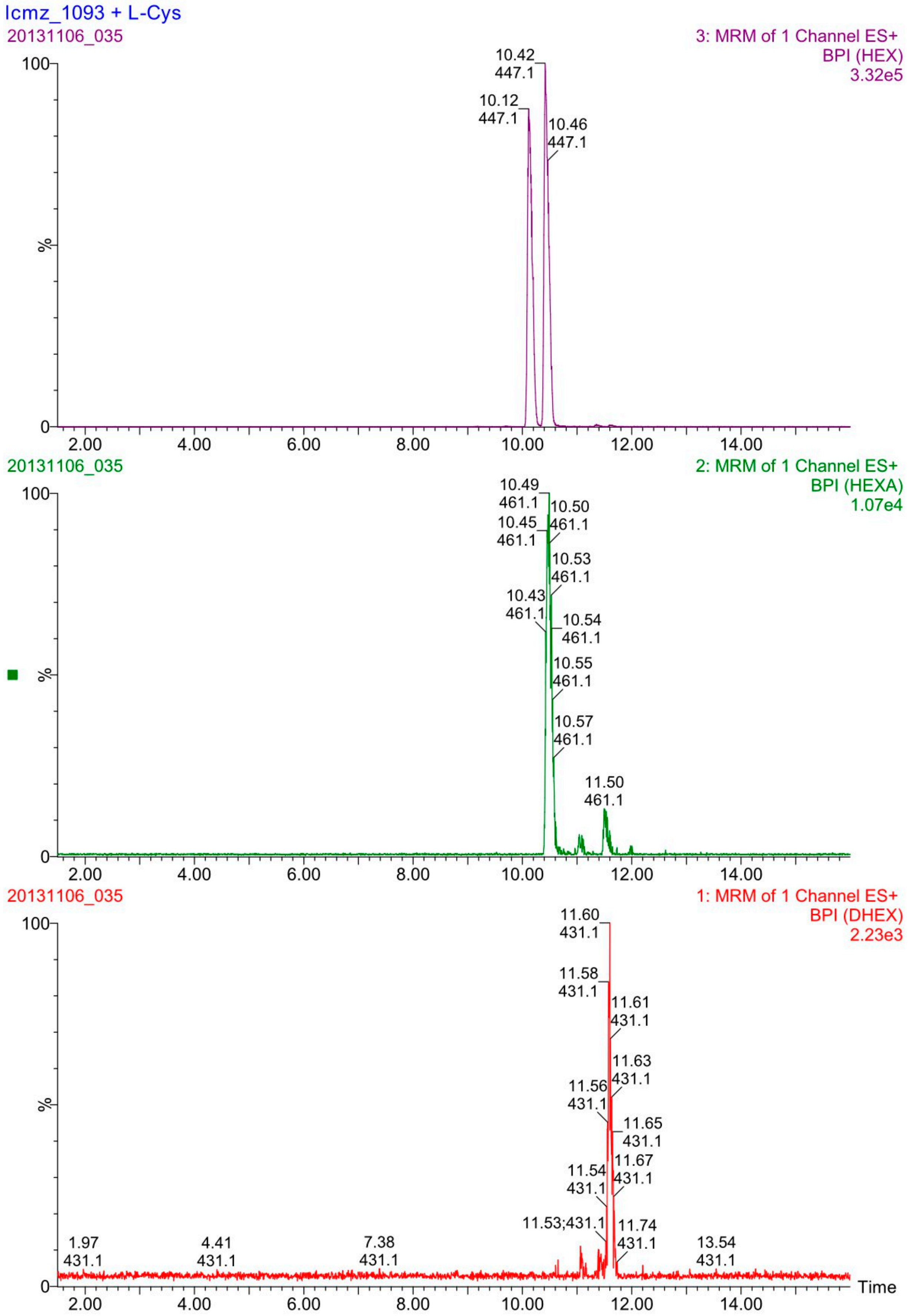

Figure S227. UPLC-MS chromatograms of the methanol extract of lentil seeds (var. Tina), and the UV spectrum of the dominant phenolic compound.

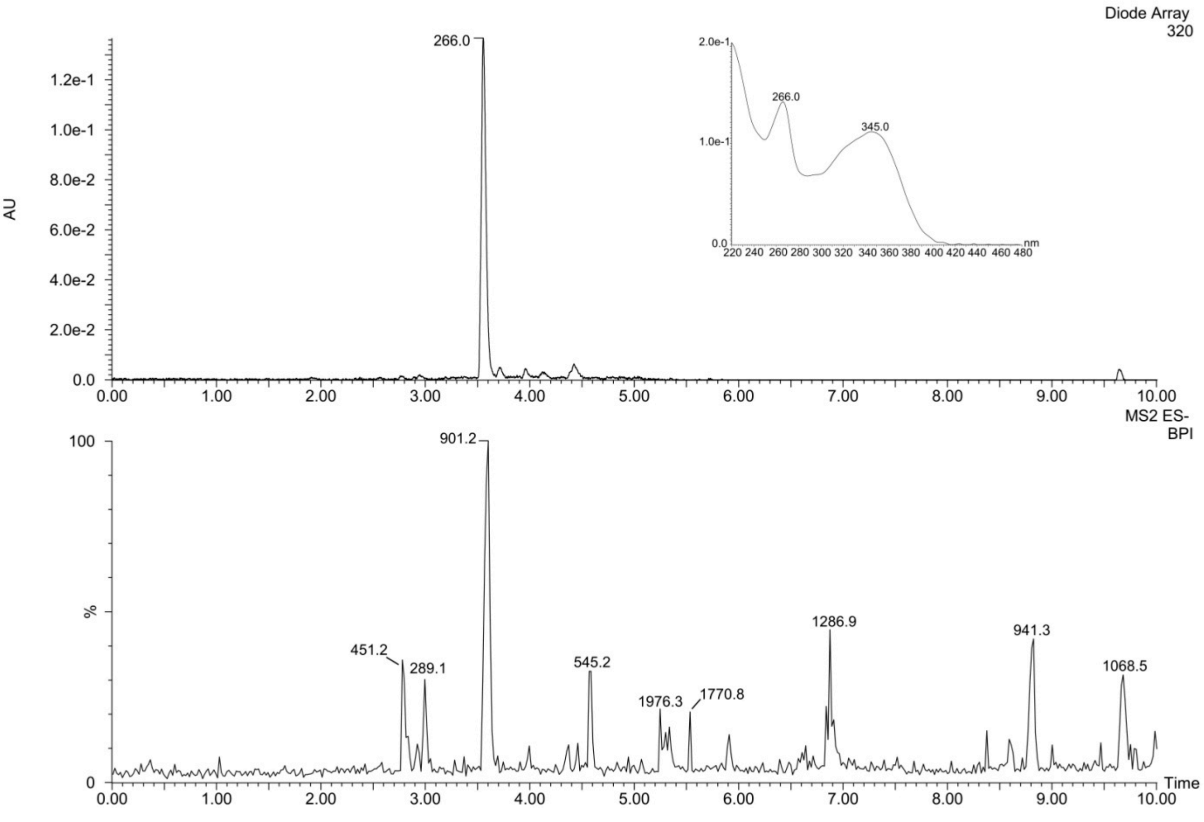

Supplement: Supplementary File 1 [file molecules-19-18152-s001.pdf]
